# The New World whirligig beetles of the genus *Dineutus* Macleay, 1825 (Coleoptera, Gyrinidae, Gyrininae, Dineutini)

**DOI:** 10.3897/zookeys.476.8630

**Published:** 2015-01-23

**Authors:** Grey T. Gustafson, Kelly B. Miller

**Affiliations:** 1 Department of Biology and Museum of Southwestern Biology, University of New Mexico, Albuquerque, NM 87131, USA

**Keywords:** Taxonomy, identification, key, morphology, aquatic arthropods

## Abstract

All New World members of the whirligig beetle genus *Dineutus* Macleay, 1825 are treated. The New World *Dineutus* are found to be composed of 18 species and 6 subspecies: one species, *Dineutus mexicanus* Ochs, 1925, **stat. n.** is elevated from subspecies to species rank, and the subspecies *Dineutus carolinus mutchleri* Ochs, 1925, **syn. n.** is synonymized here with the typical form. Lectotypes are designated for *Dineutus discolor* Aubé, 1838, *Dineutes metallicus* Aubé, 1838, *Dineutus solitarius* Aubé, 1838, *Dineutes analis* Régimbart, 1883, and *Gyrinus longimanus* Olivier, 1795. Each taxonomic unit is provided with a taxonomic history, type locality, diagnosis, distribution, habitat information, and a discussion section. The aedeagus and male mesotarsal claws are illustrated, and dorsal and ventral habitus images of both sexes, for each species and subspecies are provided. General distribution maps are provided for all taxonimc units. A key to the genera of New World Gyrinidae, as well as all the New World *Dineutus* species is provided. General *Dineutus* anatomy as well as a clarification of homology and anatomical terms is included.

## Abstract (es)

Las especies del Neuvo Mundo de género *Dineutus* Macleay, 1825 son revisadas. En el Neuvo Mundo *Dineutus* se compone de 18 especies y seis subespecies, la subespecie *Dineutus mexicanus* Ochs, 1925, **stat. n.** se eleva al rango de especie, y la subespecie *Dineutus carolinus mutchleri* Ochs, 1925, **syn. n.** es sinonimizada con la forma típica. Lectotípos son designados por *Dineutus discolor* Aubé, 1838, *Dineutes metallicus* Aubé, 1838, *Dineutus solitarius* Aubé, 1838, *Dineutes analis* Régimbart, 1883, y *Gyrinus longimanus* Olivier, 1795. Cada especie es acompañada de una diagnosis, historia taxonómica, tipo de hábitat que ocupa y una breve discusión. El edeago y uña metatarsal del macho son ilustrados, así como imágenes del habitus dorsal y ventral de ambos sexos. Se presentan mapas de distribución por especie, así como una clave para las especies del Neuvo Mundo de género *Dineutus* y los géneros de Gyrinidae del Neuvo Mundo. Características anatómicas de *Dineutus* y una clarificación de la homología de términos anatómicos son incluidos.

## Introduction

The genus *Dineutus* Macleay, 1825 is represented in the New World by 18 species and 6 subspecies. Members of *Dineutus* are among the largest of all New World whirligig beetles found in North and Central America, where they are common elements of aquatic environments including ponds, lakes, rivers, and streams. The genus is known from tropical regions around the world, but is notably absent from South America (Miller and Bergsten 2012). There have been several previous treatments of New World *Dineutus*, most of which were regional: Leech (1948) treated California; Ochs (1949), Central America; Young (1954) and Epler (1996; 2010), Florida; Gordon and Post (1965), North Dakota; Sanderson (1982) for North and South Carolina; Ferkinhoff and Gunderson (1983) for Minnesota and its adjacent states and Canadian provinces; Hilsenhoff (1990) for Wisconsin; and Ciegler et al. (2003) for South Carolina. The largest published treatments of the genus *Dineutus* for North America were only for the region north of Mexico by LeConte (1868) and Roberts (1895). Since these works there has been no comprehensive treatment of the genus published and readily available for all of the New World. Wood (1962) did treat all *Dineutus* of the western hemisphere, however, his master’s thesis was never published and portions heavily relied on previously published work for treatment of the more uncommon species. The main purpose of this project is to treat, for the first time, the entire *Dineutus* fauna of the New World, including all known species and subspecies, and the elevation of one subspecies to species status.

Classically, the genus *Dineutus* in the New World was divided into two subgenera: *Cyclinus* Kirby, 1837 and *Dineutus* *sensu stricto* by Hatch (1925b, 1930) and Ochs (1926a; 1949). This division was primarily based on the overall body form, with *Cyclinus* comprising the smaller, more elongate and narrowly oval species, with the large and broadly oval species placed in *Dineutus* *s. str.* The lack of concrete characters and seeming continuum of body sizes has been an issue discussed in the past by Hatch (1925b), and the difficulty in separating these subgenera convincingly from the African subgenus *Protodineutus* Ochs, 1926b was noted by Brinck (1955), without any resolution. Guignot (1950) divided the North American species among four different subgenera, placing most of the species within *Cyclinus*, moving most of the large and oval species to the subgenus *Protodineutus*, except *Dineutus longimanus* (Olivier, 1795) which he placed in the subgenus *Rhombodineutus* Ochs, 1926b (along with New Guinean species and a Madagascan species) and *Dineutus truncatus* (Sharp, 1873) which he maintained in *Dineutus* *s. str.* Brinck (1955), unsatisfied with Guignot’s (1950) classification, instead relegated all North American species to the subgenus *Cyclinus* without any diagnosis aside from their distribution in North America. To confound matters even more, Miller and Bergsten (2012) recently found that the North American *Dineutus* species are indeed monophyletic. The phylogenetics of the genus *Dineutus*, as well as the rest of the dineutine genera and subgenera, will be addressed in a future paper, potentially providing better evidence regarding the monophyly and potential synapomorphies for the North American *Dineutus*. Until that can be done, we recommend discontinued use of the subgenus name Dineutus (Cyclinus) for the New World species until its monophyly and diagnostic combination can be investigated more thoroughly.

This work is meant to allow for the accurate identification of all the New World species and subspecies of *Dineutus*. It is our hope that this work will help improve identification of material within public and private collections and help facilitate research on stream ecology, and aspects of these charismatic beetles’ behavior and biology. This work greatly improves upon previous identification materials by introducing a novel external character for use in identification (the male mesotarsal claws), providing high-quality habitus photos for each taxon, detailed aedeagal illustrations, distributions maps, and in-depth differential diagnoses.

The vast majority of published research on, or utilising, New World *Dineutus* species has focused on aspects of their behavior, especially those associated with their water-surface aggregrations. Many of the common *Dineutus* of North America are known to form giant multispecies and sometimes multigeneric (Realzola et al. 2007) aggregrates of hundreds to thousands of individuals, dubbed rafts by Heinrich and Vogt (1980) (reviewed for all Gyrinidae by Jäch et al. 2010). It is well established that these mass aggregations operate as a selfish herd (Hamilton 1971) for predation avoidance (Romey 1995; Romey and LaBuda 2010; Romey and Rossman 1995; Romey and Wallace 2007; Romey et al. 2008; Vulinec and Miller 1989; Watt and Chapman 1998). Several studies have looked at contact behavior of individuals within these aggregates (Freilich 1986; Knight et al. 1996; Vulinec 1987), others at composition of aggregates, daily movements, and changes in aggregate formations and indiviuals’ positions (Fairn Evan et al. 2009; Fitzgerald 1987; Freilich 1989; Heinrich and Vogt 1980; Realzola et al. 2007; Romey 1995; Romey and Galbraith 2008; Romey and Wallace 2007). Studies on population dynamics (Nürnberger 1996; Nürnberger and Harrison 1995) and evidence for competition between populations of several broadly sympatric species (Istock 1966; 1967) have also been performed. The amazing speed at which gyrinids swim, yet maintaining maneuverability (Fish and Nicastro 2003) and avoidance of collisions within aggregates (Romey et al. 2014) has been of interest more recently. This last aspect is especially interesting as it relates to whirligig beetles’ unique antennae’s ability to detect surface waves in order to avoid objects (Romey et al. 2014; Tucker 1969). Aspects of gyrinid defensive secretion have also interested researchers, as they produce a unique chemical cocktail, dubbed gyrinidal, which prevents predation (Eisner and Aneshansley 2000; Fitzgerald 1986; Meinwald et al. 1972). Studies on the vision centers of whirligig’s have also begun (Lin and Strausfeld 2012; 2013). Nearly all of the above studies have used some of the most common North American species: *Dineutus assimilis* (Kirby, 1837), *Dineutus hornii* Roberts, 1895, *Dineutus nigrior* Roberts, 1895, and *Dineutus discolor* Aubé, 1838, making it clear *Dineutus* species present excellent candidates for model study systems for a variety of areas of study.

Despite how common and abundant most species of New World *Dineutus* are, studies on their life history, and juvenile stages are shockingly missing. The general lack of life history knowledge for all North America water beetles was pointed out as early as the 1920’s by Wilson (1923), who provided an excellent general review for most families. There exists no formal study on the complete life history for any species. At most there have been anecdotal accounts of life history scattered about in the literature, or those collected from laboratory reared specimens as part of studies focused on other aspects of the species’ biology. Hatch (1925a) described briefly the life history of several common North American species, with much of the data being summarized from other sources. Smith (1926) also added some life history information on *Dineutus assimilis* in his note on behavior, but this was mostly anecdotal. Istock (1966) briefly describes some life history elements for *Dineutus assimilis*, *Dineutus hornii*, and *Dineutus nigrior*, including adult and larval durations for *Dineutus nigrior* and *Dineutus hornii*, later (1967) adding anecdotal pupal information for the later two species, but this was not the focus of Istock’s studies. The juvenile stages have similarly never been formally studied and described for any species, with the exception of *Dineutus assimilis*. Wilson (1923) provided a formal description for all life stages of *Dineutus assimilis*, including illustrations, and given the date of this work it is quite exceptional. Despite Wilson’s (1923) formal description, a modern one is still in order, as there was no incorporation of chaetotaxy. The only other work on the larval form at the species level is that of Hatch (1927), who provided a key to the first instar of four common species. Images of the larvae of *Dineutus assimilis*, *Dineutus hornii*, and *Dineutus nigrior* are provided in Istock (1967), without information as to which instar they belong, as again this was not the focus of the paper. Keys to the larvae of North America gyrinid genera have been provided, with the exception of *Spanglerogyrus* Folkerts, 1979, for which larvae remain unknown (Sanderson 1982; White and Roughley 2008). Hopefully accurate identification of species will facilitate much needed research on life history and juvenile stages in New World *Dineutus*.

## Material and methods

A total of 1674 specimens were examined for this study. Specimens were loaned in order to have each species represented for study, as the goal of this paper is to make identification possible, and to summarize available information on the New World species. This study is not meant to be a revision of the genus in North America, thus no attempt was made to obtain all available material. Material was loaned until all known species were represented for study, thus material was not loaned from some of the larger collections like the Smithsonian Institution (United States National Museum, Washington, D.C., U.S.A), as it was not necessary in order to have all species accounted for in this study. The second author has visited the Smithsonian Institution and confirmed loaning of their large quantity of material, would likely only add more locality data, at the risk of damaging specimens.

For each species a taxonomic history is provided. This list is a partial chresonomy *sensu* Dubois (2000) meant to track origins of incorrect subsequent spellings, synonyms, and changes in taxonomic rank or movement for each name. The list is not a complete logonomy or chresonomy in that every single use of the name is not tracked, but instead the list provides efficient taxonomic information for the history of the name. The family-group name classification follows Gustafson and Miller (2013).

Measurements were taking using a Cen-Tech 4 inch Digital Caliper (ITEM 47256).

Total body lengths were measured from the anterolateral margin of the clypeus to the apex of the elytral apices. These areas were chosen for the boundaries of lengths since they are more fixed than other possible boundaries. For example, the labrum may be depressed thereby making it a poor choice as an anterior boundary, and the abdomen may be more or less protruding making it an unsuitable posterior boundary. For each species and subspecies an attempt was made to measure the largest and smallest specimens available for each sex.

Specimens for dissections and imaging were relaxed by placing them in lightly boiling water.

The aedeagus was then dissected from relaxed males and placed in warm 10% KOH for about 5 minutes. Following removal from KOH the aedeagus was placed in vinegar to neutralize the base and washed in water. After dissection and/or imaging aedeagi were placed in microvials attached to the pin with the original specimens.

Images of non-aedeagal structures were drawn using a camera lucida attached to a Zeiss Discovery V8 stereo microscope. For aedeagi, drawings were made using Adobe Illustrator by tracing and modifying light photography images subsequently modified in Adobe Photoshop.

Dorsal, ventral habitus as well as aedeagal images were taken using a Visionary Digital BK+ light imaging system (www.visionarydigital.com, R. Larimer).

Handwriting on type labels was identified using Horn et al. (1990).

Specimens examined were loaned or examined from the following depositories:

AMNH American Museum of Natural History, New York, New York, U.S.A. Material from this collection was not loaned, it was examined online via http://research.amnh.org/iz/types_db/, label data was provided by S. Lodhi.

EMEC Essig Museum of Entomology, University of California, Berkeley, California, U.S.A. (P. Oboyski)

FSCA Florida State Collection of Arthropods, Gainesville, Florida, U.S.A. (M. Thomas)

GTGC Grey T. Gustafson personal collection.

IEXA Instituto de Ecologia, A.C. Xalapa, Veracruz, Mexico (R. Arce-Pérez)

KSEM Division of Entomology, University of Kansas Natural History Museum, Lawrence, Kansas, U.S.A. (A.E.Z. Short)

MCZ Museum of Comparative Zoology, Harvard University, Cambridge, Massachusettes, U.S.A. Material from this collection was not loaned, it was examined online via http://insects.oeb.harvard.edu/mcz/

MNHN Musée National d’Histoire Naturelle, Paris, France (A. Mantilleri)

MSBA Museum of Southwestern Biology Arthropod Division, University of New Mexico, Albuquerque, New Mexico, U.S.A. (K.B. Miller)

MTEC Montana Entomology Collection, Montana State University, Bozeman, Montana, U.S.A. (M. Ivie)

NMPC National Museum, Prague, Czech Republic (M. Fikáček)

UCRC University of California Entomology Research Museum, Department of Entomology, Riverside, California, U.S.A. (D. Yanega)

WIBF West Indian Beetle Fauna Project, Montana Entomology Collection, Bozeman, Montana, U.S.A. (M. Ivie)

ZMHB Museum für Naturkunde der Humboldt Universitat zu Berlin, Berlin, Germany (M. Uhlig)

### On the homology of the gyrinid anatomy and terminology

In many groups of aquatic beetles morphological structures, especially the legs, have been rotated, and maintaining appropriate homology relative to other groups of beetles is critical (Miller and Nilsson 2003). Within the Gyrinidae the current position of all the legs are different from the ancestral condition within Adephaga. Gyrinidae have their prothoracic legs rotated ca. 90° anteriorly in normal anatomical repose, whereas the meso- and metathoracic legs have been rotated ca. 90° posteriorly, similar to the condition in Dytiscidae (Miller and Nilsson 2003). Therefore, the surfaces homologous with other beetles have changed such that what appears to be the “ventral” and “dorsal” surfaces are actually the anterior and posterior surfaces in the meso- and metathoracic legs (Miller and Nilsson 2003). Similarly the “true” ventral and dorsal surfaces are now located “posteriorly” and “anteriorly” in the meso- and metathoracic legs. Both situations are reversed in the prothoracic legs, which have been rotated in the opposite direction relative to the meso- and metathoracic legs. Previous gyrinid workers (e.g. Brinck 1955) have not used terminology reflecting the homologous surfaces, but we here follow Miller and Nilsson’s (2003) suggestion to use terminology more carefully reflecting this.

**Figure 1.**
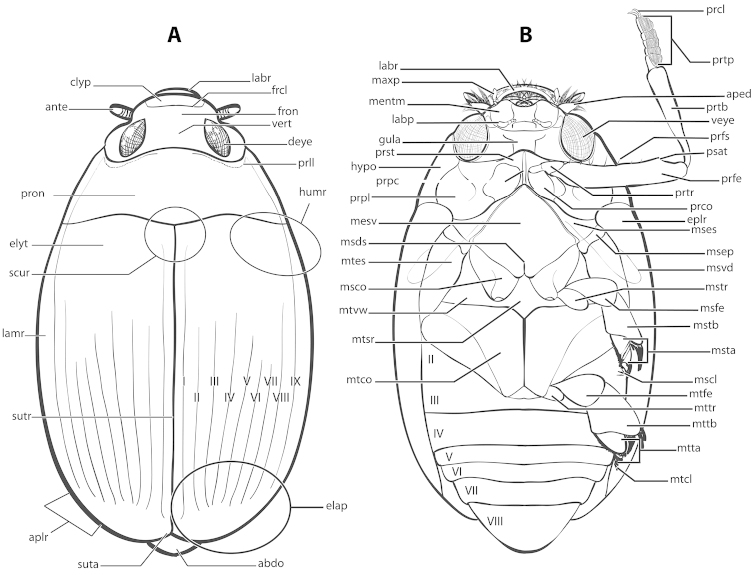
Generalized anatomy of *Dineutus* adult ♂ **A** dorsal. clyp: clypeus, ante: antenna, pron: pronotum, elyt: elytron, scur: scutellar region, lamr: lateral marginal depression of elytron, sutr: elytral suture, aplr: apicolateral angle of elytral apex, suta: sutural angle of elytral apex, abdo: abdomen, elap: elytral apex, I–IX: elytral striae, humr: humeral region of elytron, prll: pronotal transverse impressed line, deye: dorsal eye, vert: vertex, fron: frons, frcl: frontoclypeal suture, labr: labrum **B** ventral. labr: labrum, maxp: maxillary palp, mentm: mentum, labp: labial palp, gula: gula, prst: prosternum, hypo: hypomeron, prpc: prosternal process, prpl: propleuron, mesv: mesoventrite, msds: mesothoracic discrimen, metes: metanepisternum, msco: mesocoxa, mtvw: metaventral wing, mtsr: metaventrite, mtco: metacoxa, II–VIII: abdominal sternites, metcl: metatarsal claw, mtta: metatarsus, mttb: metatibia, mttr: metatrochanter, mtfe: metafemur, mscl: mesotarsal claw, msta: mesotarsus, mstb: mesotibia, msfe: mesofemur, mstr: mesotrochanter, msvd: mesoventrite depression for receiving prothoracic leg, msep: mesepimeron, mses: mesanepisternum, eplr: elytral epipleuron, prco: procoxa, prtr: protrochanter, prfe: profemur, psat: profemoral sub-apicoventral tooth, prfs: ventral profemoral setae, veye: ventral eye, prtb: protibia, aped: antennal pedicel, prtp: setose pad of protarsus, prcl: protarsal claw.

**Figure 2.**
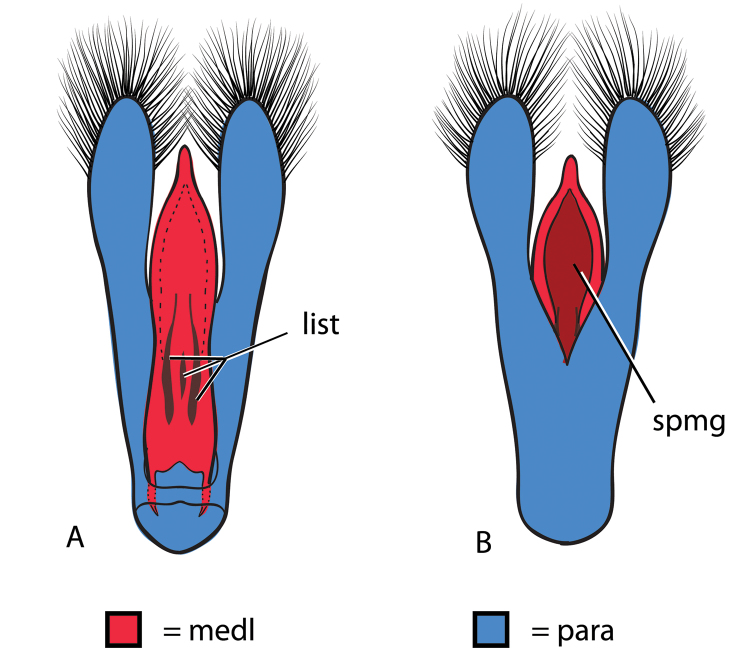
Generalized anatomy of *Dineutus* aedeagus. **A** dorsal view medl: median lobe, list: longitudinal lists of the median lobe, para: parameres **B** ventral view spmg: sperm-groove.

Other terminology referring to gyrinid morphology largely follows that of Lawrence et al. (2011), Brinck (1955) and Roberts (1895). A detailed illustration of *Dineutus* morphology and terminology used here is provided in Figs 1 and 2. There are seven visible adominal ventrites in *Dineutus*, however, the roman numerals given in Fig. 1B refer to the true homologous abdominal sternite count, with the first sternite being hidden by the metacoxae (Lawrence et al. 2011). While it appears that there are actually only six visible ventrites, the first visible ventrite is actually two fused sternites (a suture is still visible in some taxa such as species of *Enhydrus* (Brinck 1955; 1978; Miller and Bergsten 2012)).

### Structures of taxonomic importance

The purpose of this paper is to allow for the identification of all the species of *Dineutus* within the New World. A key to the genera of Gyrinidae within the New World as well as to all the species of *Dineutus* is provided. The key to species works for both males and females. The identification of *Dineutus* can be done without many difficulties using only external characters. However, several characteristics of the genus make identification somewhat difficult. First, males and females tend to be dimorphic with the females of several species appearing very similar (e.g. *Dineutus assimilis*, *Dineutus nigrior*, and *Dineutus hornii*). Whereas gyrinids are often collected in large rafts or aggregrates, these aggregates often contain multiple species or even multiple genera (Realzola et al. 2007). Therefore, each specimen may need to be identified carefully to avoid assuming all belong to the same species. Furthermore, many species are separated by very minor external characters, but differ drastically in genitalia. Therefore the dissection of genitalia is often required for reliable identification and may require dissection of multiple specimens from a series to ensure all species within the aggregation are identified correctly. For this reason, for each species, a dorsal and ventral habitus are included of both sexes, as well as illustrations of the aedeagus and the male mesotarsal claws.

**Mesotarsal claws (**Fig. 1B **mscl):** The mesotarsal claws of New World *Dineutus* are sexually dimorphic, a feature that has been overlooked in previous treatments of the group. Males have the mesotarsal claws modified in several ways causing them to differ significantly from the metatarsal claws, whereas the mesotarsal claws of females are more similar in form to the metatarsal claws in terms of size and shape. The mesotarsal claws of females do differ from the metatarsal claws, however, not to the degree of the male’s. The modifications to the male mesotarsal claws include increased size (Fig. 32C), the presence of a denticle on the ventral margin of one or both claws in some species (Fig. 36C), and variations in the curvature of the ventral margin of the claw, from straight (Fig. 17C) to strongly curved (Fig. 21C). The mesotarsal claws are useful as an external diagnostic character to distinguish between several externally similar species such as *Dineutus assimilis* (Fig. 9C) and *Dineutus nigrior* (Fig. 32C).

**Protibial shape (**Fig. 1B **prtb):** The overall shape of the male protibia has been historically used as a diagnostic character (Roberts 1895; Wood 1962; and Ochs 1949) and is used here, though it is not always diagnostic. This is another sexually dimorphic character. Males typically have a variety of protibial shapes, whereas females most often have a more generalized club-shaped protibia. There are three protibial shapes seen in New World *Dineutus*: club-shaped, wedge-shaped, and subsinuate. Club-shaped is the most general of the protibial shapes, and is characterized by more or less similar protibial width throughout the entire protibial length (Fig. 44C, D), or the protibia is only weakly expanded distally (Fig. 14C, D). This shape occurs in most of the Central American and Mexican species, as well as in the female of the majority of species. The wedge shape is represented by a more or less even expansion distally and is exemplified by males *Dineutus assimilis* (Fig. 8C), *Dineutus nigrior* (Fig. 31C), and *Dineutus hornii* (Fig. 18C). The subsinuate shape is represented by the protibia with the medial margin more or less abruptly expanded distally with the proximal half of the protibia narrow and parallel sided. Species exemplifying this modification are *Dineutus productus* (Fig. 33C) and *Dineutus serrulatus serrulatus* (Fig. 40C).

**Profemoral dentation and the sub-apicoventral profemoral tooth (**Fig. 1B **psat):** The profemora of New World *Dineutus* possess a very distinct, large denticle located sub-apically on the posterior margin of the ventral surface (Fig. 1B psat) (Roberts 1895). The presence or absence of this tooth is diagnostic (Roberts 1895). Additional features of the tooth, such as relative size, are also useful to distinguish between species that are otherwise externally very similar, for example *Dineutus emarginatus* (Fig. 16D) and *Dineutus carolinus* (Fig. 10D). This tooth can be present on a short carina in some species like *Dineutus carolinus* (Fig. 10D) and may sometimes be reduced, with only the carina apparent.

This character is another case of sexual dimorphism, present only in males. An exception to this may be *Dineutus amazonicus* Hatch, 1930, which is characterized by the presence of a profemoral sub-apicoventral tooth in females. For more discussion on the enigmatic *Dineutus amazonicus* see the discussion section for that species.

There are other variants of the dentation of the ventral profemora of North American *Dineutus*. Some species have a second more basal profemoral denticle (i. e. *Dineutus longimanus*), but for the purpose of this study only the sub-apicoventral denticle, referred to through the rest of this paper as the profemoral sub-apicoventral tooth, was used for diagnostic purposes, as the development of the more basal denticles varied noticeably within species. One species, *Dineutus productus*, has a unique dentation consisting of a series of denticles along the femoral margin with each denticle associated with a setigerous puncture, and the sub-apicoventral tooth distinct.

**Figure 3.**
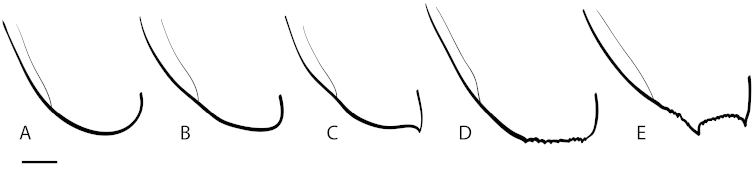
Elytral apex types of North American *Dineutus*. **A** regularly rounded, without apicolateral sinuation **B** flatly rounded/sub-truncate, with apicolateral sinuation **C** sutural angle produced, with apicolateral sinuation **D** truncate, with serrationb **E** spinose, with serration.

**Elytral apices (**Fig. 1A **elap;** Fig. 3**):** The elytral apices are highly diagnostic for North American *Dineutus* (Fig. 3) (Roberts 1895). The elytral apices have two general features of diagnostic importance, the presence or absence of apical serration and/or irregularities, and overall shape. Serration consists of tiny conical cuticular extensions located at the apices of the elytra, usually near the sutural angle and not present laterally beyond the apicolateral angle (Fig. 3E). This serration in some cases is very fine, composed of distinct points, as in *Dineutus truncatus*, or is reduced to irregularities (bumps, weak cones, or projections resulting in an irregularly non-smooth edge), as in *Dineutus mexicanus* or *Dineutus productus*, with the character varying both within and among populations. Some species never have serrations and/or irregularities present.

The general shape of the elytral apices is also diagnostic of groups of species. In some species, the elytral apices are simple and evenly rounded (Fig. 3A) characteristic of *Dineutus carolinus* (Fig. 10A, C) and *Dineutus emarginatus* (Fig. 19A, C). Another form of rounded elytral apices is present, where the apex is much more flatly rounded, as opposed to regularly evenly rounded, which is referred to here as flatly rounded (Fig. 3B). This flatly rounded apex, sometimes has a near truncate appearance, for which it is also referred to as subtruncate. Species exemplifying the flatly rounded/subtruncate elytral apices are *Dineutus sublineatus* (Fig. 44A), *Dineutus discolor* (Fig. 14A), and *Dineutus serrulatus serrulatus* (Fig. 40A) The most common modification of the elytra is to have the sutural angle produced into a broad triangular point (Fig. 3C) as in *Dineutus productus* (Fig. 33A) and *Dineutus nigrior* (Fig. 31C). This production can be variable from nearly totally reduced to well evident. When variable, examination of other diagnostic character states may be required for identification. The more modified elytral apices are truncate (Fig. 3D) or spinose (Fig. 3E). Truncation is only truly present in *Dineutus truncatus* (Fig. 47A, C) and *Dineutus mexicanus* (Fig. 28A, C) and spinosity is present in *Dineutus longimanus* (Fig. 20A, C). Several of these conditions are accompanied by serration as described above.

**Venter coloration:** The coloration of the ventral surface is another important diagnostic character (Roberts 1895). Most New World species of *Dineutus* have the ventral surface dark, usually dark reddish brown to dark brownish black in coloration. However, several species have the venter lightly colored from yellowish orange to red. Light colored species tend to be lotic and associated with streams. These include *Dineutus robertsi* (Fig. 35B, D), *Dineutus discolor* (Fig. 14B, D), *Dineutus angustus* (Fig. 6B, D), and *Dineutus serrulatus serrulatus* (Fig. 40B, D). Only *Dineutus serrulatus analis* appears to be variable in venter coloration (Fig. 39), at times lightly colored red, while others being dark reddish black.

**Antennal shape (**Fig. 1A **ante):** The shape of the antennae is useful for identification with overall thickness and appearance of the ultimate antennomere useful for distinguishing very similar species like *Dineutus hornii* and *Dineutus assimilis*, especially when comparing female specimens. But in general antennal shape alone is not diagnostic.

**Aedeagus (**Fig. 2**):** For this study the male aedeagus was considered to be an important indicator of species boundaries. The male aedeagus is by far the best diagnostic feature for species identification of North American *Dineutus*. Most species have clear and easily distinguishable characters associated with the aedeagus, including the shape of the median lobe, the shape of the sperm-groove, and the shape of parameres. The aedeagus is best viewed in the dorsal aspect (Fig. 2A) for most diagnostic features, such as acumination of the apex of the median lobe, whereas the ventral aspect (Fig. 2B) is used much less frequently for comparison between species’ sperm-grooves (Fig. 2B spmg). The lateral view can be quite informative for determination of species where the apex is dorsally curved (e.g. *Dineutus nigrior* Fig. 32D). The aedeagus should not be viewed nor kept dried as the shape of the median lobe becomes distorted, and should be maintained in a microvial with glycerin and viewed in water. Dissection and examination of the male genitalia is the best means for identification of species.

**Setigerous profemoral punctures (**Fig. 1B **prfs):** In the past, the number of setigerous profemoral punctures present on the anterior surface of the prothoracic legs has been used as a diagnostic character for separating species (Roberts 1895; Ochs 1924, 1929; Young 1954). However, as has been previously found (Wood 1962), these characters are not useful diagnostically. Species vary in the number of setigerous punctures, especially in those that have a large degree of size variation, with larger specimens tending to have an increased number relative to smaller specimens. Further, the setae are often lost and the punctures themselves can be difficult to see in dirty specimens. For these reasons, the number of setigerous profemoral punctures is not used here. There is also a row on the posterior surface of the profemora, but, similarly, these were found to be unreliable as diagnostic features.

**Dorsal coloration:** The dorsal coloration of North American *Dineutus* varies from olive green in color, to metallic bronze, but most species are black to dark green. Although there are some differences in dorsal coloration, the diagnostic utility of this feature is not great. In North American *Dineutus* examined, there occur specimens of both sexes that are much more darkly colored, usually appearing black, even in species normally olive-green in dorsal color. These “melanized” forms occur across the species and, as such, there are specimens in all species that are not diagnosable using color. This melanization occurs naturally (noticeable even in the field during collection) and is not a result of preservation. For this reason, dorsal coloration is described but is not used as a comprehensively diagnostic feature.

## Taxonomy

### Key to the adults of the genera of Gyrinidae within the New World

**Table:** 

1	Dorsal eyes widely divided from ventral eyes; mesothoracic and metathoracic legs broad and highly anteroposteriorly flattened; abdominal sternite VIII not medially deeply emarginate	**2**
–	Dorsal eyes narrowly divided from ventral eyes; mesothoracic and metathoracic legs narrow, not broad; abdominal sternite VIII medially deeply emarginate. Southeastern United States	***Spanglerogyrus albiventris* Folkerts, 1979**
2	Scutellar shield hidden with elytra closed	**3**
–	Scutellar shield visible with elytra closed	**4**
3	Size larger (normally > 9 mm). Elytra and pronotum without pubescent margins; abdominal sternite VIII broadly rounded, not conical, glabrous throughout. Southeastern Canada, United States primarily east and south of the Rocky Mountains, through Mexico and Central America to western Panama, absent from South America	***Dineutus* Macleay, 1825**
–	Size smaller (normally < 9 mm). Pronotum and elytra with pubescent margins; abdominal sternite VIII conical, sternites VII and VIII with line of long setae medially. Southern half of United States, Mexico, Central and South America	***Gyretes* Brullé, 1835**
4	Size larger (12–15 mm). Pronotum without medial transverse depression; elytra with 9 striae present as distinctive longitudinal lines, dorsally olive green in color. Known in Central America only from Panama and Costa Rica, primarily South American	***Enhydrus* Laporte, 1834**
–	Size smaller (rarely > 9 mm, usually < 12 mm). Pronotum with medial transverse depression; elytra with up to 11 striae present most often as linear series of punctures, dorsally commonly black in color. Found throughout most of North and South America	***Gyrinus* Geoffroy, 1862**

### Key to the adults of the New World species of *Dineutus*

**Table:** 

1	Protarsi expanded, posteriorly covered with adhesive suction-cup setae; mesotarsal claws and metatarsal claws dissimilar in shape and/or size, mesotarsal claws modified and/or larger: males (Fig. 1B prtp)	**2**
–	Protarsi not expanded, narrow and parallel sided, posteriorly not covered with adhesive suction-cup setae (Fig. 4B); mesotarsal and metatarsal claws similar in shape, mesotarsal claws not noticeably larger: females	**19**
2	Profemora without sub-apicoventral tooth of any size (Fig. 8D); elytral apices not spinose	**3**
–	Profemora with sub-apicoventral tooth of various sizes and shapes (Fig. 16D); elytral apices various	**7**
3 (2)	Elytral apices regularly (Fig. 3A) and or flatly rounded/sub-truncate (Fig. 3B), sutural angle never produced; mesotarsal claws similar in size with ventral margins either shallowly curved or with denticle	**5**
–	Elytral apices modified, having the sutural angle produced into a more or less developed point (Fig. 3C), rarely rounded or flatly rounded/sub-truncate and without point: if rounded and/or flatly rounded and without point, then protibiae wedge-shaped (Fig. 8C), not club-shaped (Fig. 12C), epipleuron of elytra similar in color as adjacent thorax and abdomen, never lightly colored orange or yellow, and metallic sheen often present. No apical serration present, at most some irregularities or wrinkles; mesotarsal claws either noticeably asymmetrical or with claws similar in size but with the ventral margins straight; not with claws similar in size with ventral margins shallowly curved, nor with a denticle	**4**
4 (3)	Size larger: 11.1–11.7 mm; body outline more broadly oval (Fig. 31C); elytral apices modified with sutural angles produced into a point, lateral sinuation feeble or absent, apical serration absent; protibiae wedge-shaped, with distolateral margin expanded laterally at apex; mesotarsal claws (Fig. 32C) noticeably asymmetrical with anterior claw larger than posterior claw, ventral margins shallowly curved; venter (Fig. 31D) darkly colored, sometimes with metallic luster, epipleuron similarly colored as thorax and abdomen with metallic luster. Aedeagus (Fig. 32A, B, D) with median lobe narrowed in apical 1/4 with shortly rounded apex, not acuminate, in lateral view, apex of median lobe strongly curved dorsally. Southeastern Canada and eastern half of United States	***Dineutus nigrior***
–	Size smaller: 9.9–11.1 mm; body outline more elongate and narrowly oval (Fig. 8C); elytral apices modified with sutural angle more or less produced into a point, lateral sinuation feeble or absent, apical serration absent some irregularities may be present; protibiae wedge-shaped, distolateral margin straight for entirety, not expanded on the distal lateral apex; mesotarsal claws (Fig. 9C) not noticeably asymmetrical, anterior claw not much larger than posterior claw, ventral margin straight, not curved; venter (Fig. 8D) often with metallic luster, epipleuron similarly colored as thorax and abdomen. Aedeagus (Fig. 9A, B, D) with median lobe acuminate in apical 1/5, apex narrowly and shortly rounded, in lateral view not strongly curved dorsally. Very common and widespread from southeastern Canada throughout much of the United States and possibly northern Mexico	***Dineutus assimilis***
5 (3)	Body form broadly oval, generally larger in size: 12.1–15.5 mm, protibiae club-shaped. Elytra with a metallic luster being either entirely bronzy or with a bronzy lateral stripe; mesotarsal claws more similarly sized as metatarsal claws, ventral margins with a denticle	**6**
–	Body form elongate oval (Fig. 18C), attenuated anteriorly; medium size: 9.9–10.9 mm. Protibiae wedge-shaped, not club-shaped; Elytra without a bronzy lateral stripe ending before elytral apex; venter darkly colored (Fig. 18D) usually dark reddish, rarely with a metallic luster, if metallic luster present then it is weakly developed, epipleuron lighter in coloration, usually yellowish orange (Fig. 18D); mesotarsal claws (Fig. 19C) similar in size and noticeably larger than metatarsal claws, ventral margin shallowly curved, not straight, and without denticle. Aedeagus (Fig. 19A, B, D) with median lobe in dorsal view mostly parallel sided, evenly narrowed in apical 1/3, apex strongly narrowed, in lateral view apex of median lobe weakly curved dorsally; parameres in dorsal view laterally expanded in apical 1/4, nearly evenly rounded apically. Southeast Canada and eastern half of the United States	***Dineutus hornii***
6 (5)	Venter usually dark reddish-brown (Fig. 12D), rarely light reddish orange with yellow mesothoracic leg and metathoracic legs; Antennal flagellum thicker and less parallel sided, ultimate segment much more rounded, less elongate and much less pointed apically; anterior mesotarsal claw (Fig. 13C) without strong denticle; elytra (Fig. 12C) with lateral bronzy stripe disappearing apically, elytral striae weakly developed; last lateral elytral interval without punctures, rarely with lateral punctures indistinctly present. Aedeagus (Fig. 13 A, B, D) with medial lobe in dorsal view with apicomedial papilla, in ventral view sperm-groove parallel sided, in lateral view median lobe curved, apically narrow, parameres weakly rounded laterally in apical 1/3. Eastern United States, southwest to Texas. Widespread and commonly collected	***Dineutus ciliatus***
–	Venter light in coloration (Fig. 12D): yellowish, not dark reddish-brown; epipleuron with lateral margin darkened; Antennal flagellum narrower and more parallel sided, ultimate segment more elongate and pointed apically; anterior mesotarsal claw (Fig. 36C) with strong denticle; elytra (Fig. 35C) without distinct lateral bronzy stripe disappearing apically, instead nearly entirely bronzy; elytral striae faint, but fairly distinct, last lateral stria with punctures distinguishable. Aedeagus (Fig. 36A, B, D) with median lobe without apicomedial papilla, more flatly rounded apically, sperm-groove more triangular, in lateral view median lobe thick and flat, parameres more strongly arced in apical 1/3. Appalachian mountains of northeastern Georgia, and southwestern North and South Carolina. Uncommonly collected Appalachian endemic	***Dineutus robertsi***
7 (2)	Elytral apices regularly rounded (Fig. 3A), sometimes flatly rounded/subtruncate (Fig. 3B), but without modifications and not truly truncate (Fig. 3D). If venter red in coloration then flatly rounded apices always accompanied by serration and/or irregularities (if flatly rounded without apical serration and/or irregularities and reddish venter go to couplet 12)	**8**
–	Elytral apices variously modified, either with true truncation (Fig. 3D), spinosity (Fig. 3E), and/or sutural angles produced to a more or less developed point (Fig. 3C)	**12**
8 (7)	Size smaller: 8–11.7 mm. Elytra with elytral striae faint, not well developed, most evident medially on elytral disc, not all striae easily visible	**9**
–	Size larger: 12.3–15.5 mm. Elytra (Fig. 44C) with elytral striae very well developed all 9 mostly and easily visible. Dorsally usually olive green in coloration, ventrally very dark reddish brown to black. Elytral apices regularly to flatly rounded/subtruncate without serrations and or irregularities; protibiae club-shaped; sub-apicoventral tooth usually very large and acute. Aedeagus (Fig. 45A, B, D) with median lobe in dorsal view nearly as long as parameres, narrow, becoming attenuated apically, apex very narrowly rounded, parameres in dorsal view weakly expanded laterally at apical 1/3, apex obliquely angled. Primarily in Mexico and Central America, just reaching the extreme southwestern United States	***Dineutus sublineatus***
9 (8)	Elytral apices with serration and/or irregularities present apically, elytral apices either regularly (Fig. 3A) or flatly rounded (Fig. 3B), apicolateral sinuation either present and shallow, or absent; mesotarsal claws with ventral margins shallowly curved (Fig. 11F) or with a weak denticle (Fig. 38F)	**10**
–	Elytral apices without serration or irregularities apically, no apicolateral sinuation present, apices regularly rounded (Fig. 3A); mesotarsal claws with ventral margins straight (Fig. 17C) or anterior claw mostly straight with a slight ventral expansion at about half its length (Fig. 43C)	**11**
10 (9)	Size generally larger: 9.9–11.7 mm; Elytra (Fig. 40C) with apicolateral sinuation present, lateral marginal depression broad when present; profemoral tooth (Fig. 40D) often well developed, sometimes small and weakly developed (Fig. 39E); anterior mesotarsal claw (Fig. 38F) short with ventral margin possessing a denticle; venter usually red in color, sometimes very dark red almost black. Aedeagus (Fig. 41A, B, D) with median lobe in dorsal view as long as parameres, highly parallel sided, narrowed in apical 1/4, apex flatly to regularly rounded, parameres in apical 1/3 weakly laterally expanded, apically obliquely flatly rounded to truncate. Eastern United States	***Dineutus serrulatus***
–	Size generally smaller: 9.1–10.9 mm; Elytra (Fig. 10C) with apicolateral sinuation usually absent, feeble if at all present; Profemoral tooth small (Fig. 10D), not well developed, often atop a small ridge; distinct marginal depression present on elytra, most evident in humeral angle, which is greatly expanded posteriad; mesotarsal claws (Fig. 11F) with ventral margin shallowly rounded, lacking denticle; venter (Fig. 10D) darkly colored, usually black to very dark brown, not red. Aedeagus (Fig. 11) with median lobe in dorsal view nearly as long as parameres, not parallel sided, being widest basally and regularly narrowed apically, becoming more narrowed in apical 1/3, some populations more much more noticeably narrowed, apex very shortly rounded; parameres parallel sided, not laterally expanded, broadly rounded apically, not flatly rounded to truncate. Eastern United States in to the Caribbean	***Dineutus carolinus***
11 (9)	Profemoral sub-apicoventral tooth small (Fig. 42D); body form (Fig. 42C) more regularly oval; pronotum with lateral edge obtusely rounded posteriorly to anteriorly; elytral apices broadly rounded; size: 9.2–10.4 mm. Aedeagus (Fig. 43A, B, D) with median lobe just shorter than parameres, narrow and mostly parallel sided with apical margin angled towards acumination, acumination strongly pointed with lateral margins narrowed to apex, apex narrowly rounded and sharp; parameres without medial constriction. California, Texas, Mexico, and through Central America	***Dineutus solitarius***
–	Profemoral sub-apicoventral tooth large and triangular (Fig. 16D); body form (Fig. 16C) more elongate oval; pronotum with lateral edge more straightly angled posteriorly to anteriorly; elytral apices more narrowly rounded; size: 8.6–11 mm. Aedeagus (Fig. 17A, B, D) with median lobe shorter than parameres, apically rounded towards acumination, not parallel sided, acumination strongly pointed with lateral margins fairly parallel sided towards apex, not narrowed, apex shortly rounded, not sharp; parameres with medial constriction giving lateral margins a sinuous shape. Eastern United States	***Dineutus emarginatus***
12 (7)	Elytral apices spinose (Fig. 3E): each elytron produced apically into two points, one sutural and the other parasutural, serration present apically. Caribbean endemic	***Dineutus longimanus***
–	Elytral apices not spinose; either truncate (Fig. 3D) or with the sutural angle produced to a more or less pronounced point (Fig. 3C)	**13**
13 (12)	Elytral apices truncate (Fig. 3D); Size large 13.5–17.5 mm. Dorsally olive green in coloration, ventrally dark reddish brown to black. Mexican and Central American species	**18**
–	Elytral apices not truncate, elytra with sutural angle produced to a point (Fig. 3C). Size under 13 mm. Dorsally and ventrally various in coloration, some species with lighter colored venters	**14**
14 (13)	Body form regularly oval (Fig. 4C), not attenuated anteriorly; dorsally often olive green in coloration for entirety; Elytra without lateral marginal depression, at most weakly apparent, striae indistinct, lateral margins with strong reticulation giving a bronzy appearance, apices with the sutural angle produced into a more or less developed point; serrations and irregularities present apically; profemora (Fig. 4D) with sub-apicoventral tooth small and weakly developed. Aedeagus (Fig. 5A, B, D) with median lobe in dorsal view shorter than parameres, nearly parallel sided, narrowed in apical 1/6, parameres weakly expanded laterally in apical 1/3, medially weakly curved, flatly rounded apically. Caribbean endemic	***Dineutus americanus***
–	Body form attenuated anteriorly (Fig. 14C), elongate oval; not often dorsally olive green, more often very darkly colored black to bronzy, if olive green than other coloration often present: Elytral apices with or without serrations and irregularities present apically. Continental North American species	**15**
15 (14)	Irregularities and/or serration present apically on elytra; venter either dark or lightly colored; sub-apicoventral tooth variable, may be accompanied by denticles	**16**
–	Irregularities and/or serration absent apically on elytra; venter always lightly colored red to light orange red; profemoral sub-apicoventral tooth small, never accompanied by denticles	**17**
16 (15)	Small in size: 9.5–9.6 mm. Profemoral sub-apicoventral tooth small and accompanied by a row of denticles associated with each setigerous puncture of the posterior face of the profemora, proceeding proximad; mesotarsal claws (Fig. 34C) elongate, with ventral margin straight, not accompanied by denticle; elytral apices (Fig. 33C) with sutural angle produced to a point; apices near suture deflexed often accompanied by a swelling of the first interval of the elytra just posterior to the deflexion; apices near suture with irregularities or bumps, not fine or noticeable serration; apicolateral sinuation weakly developed; venter (Fig. 33B) darkly colored: usually black to very dark brown, often bronzy with a metallic luster, final abdominal segment sometimes lightly colored. Aedeagus (Fig. 34A, B, D) with median lobe in dorsal view noticeably shorter than parameres, weakly constricted just apicad to middle, acuminate in apical ca. 1/5, apex of acumination flatly rounded; parameres sinuate laterally after basal 1/3, in apical 1/3 laterally expanded, apically strongly rounded. Restricted to middle United States from Illinois, Kansas, to Texas and south to Nuevo Leon, Mexico. Uncommonly collected species	***Dineutus productus***
–	Larger in size: 9.9–11.7 mm. Profemora with sub-apicoventral tooth not accompanied by a row of denticles associated with each setigerous puncture of the posterior face of profemora proceeding proximad; mesotarsal claws (Fig. 38F) short, ventral margin strongly curved, accompanied by a denticle; elytral apices (Fig. 37C) often with noticeable serration present near suture; apicolateral sinuation noticeable; venter (Fig. 37D) usually red to very dark red, rarely appearing entirely black (if black extremities of venter appearing dark red). Aedeagus (Fig. 38) with median lobe in dorsal view as long as parameres, highly parallel sided, not strongly acuminate apically, but narrowed in apical 1/4, apex flatly to regularly rounded, parameres in apical 1/3 weakly laterally expanded, apically obliquely flatly rounded to truncate, not strongly rounded. Eastern United States. Widespread and fairly common species	***Dineutus serrulatus***
17 (15)	Smaller in size: 9.4–10.8 mm. Body form narrow and parallel sided (Fig. 6C); dorso-ventrally convex. Aedeagus (Fig. 7A, B, D) with median lobe in dorsal view shorter than parameres, nearly parallel sided, narrowed in apical 1/6; parameres weakly expanded laterally in apical 1/3, medially weakly curved, flatly rounded apically. Northern Florida and southern Georgia and Alabama. Restricted in range and less commonly collected species	***Dineutus angustus***
–	Larger in size: 10.9–12.1 mm. Body form elongate oval, attenuated anteriorly (Fig. 14C); typically dorso-ventrally depressed posterior to the scutellum. Aedeagus (Fig. 15A, B, D) with median lobe in dorsal view weakly constricted medially, weakly narrowed in apical 1/3, apex obtusely rounded; parameres narrow, parallel sided, not weakly curved, apically very flatly rounded. Southeastern Canada, Eastern United states, possibly in to Northeastern Mexico. Widespread and common species	***Dineutus discolor***
18 (13)	Elytral apices finely serrulate; protarsi (Fig. 30C) with ultimate protarsomere ca. ×2 as long as wide; mesotarsal claws (Fig. 48C) with ventral margin not expanded into weak denticle. Aedeagus (Fig. 48A, B, D) with median lobe nearly evenly tapered towards apex, not acuminate. Central America: Nicaragua to Panama	***Dineutus truncatus***
–	Elytral apices not finely serrulate, apices with weak serrulation and or irregularities; ultimate protarsomeres less than ×2 as long as wide (Fig. 30A); mesotarsal claw (Fig. 29C) with ventral margins expanded basally into weak denticle; Aedeagus (Fig. 29A, B, D) with median lobe apically acuminate. Mexico into Central America as far as Honduras and El Salvador	***Dineutus mexicanus* stat. n.**
19 (1)	Profemora without sub apicoventral tooth	**20**
–	Profemora with sub apicoventral tooth; elytral apices regularly rounded with weak apicolateral sinuation, serrations and or irregularities absent; venter darkly colored. Species known only from a single female specimen from Sevier Co., Arkansas, U.S.A.	***Dineutus amazonicus***
20 (19)	Elytral apices (Fig. 3E) with two pronounced spines with serrations and irregularities present apically; venter often lightly colored, but also dark reddish brown. Caribbean endemic	***Dineutus longimanus***
–	Elytral apices without spinosity, variously modified	**21**
21 (20)	Elytral apices truncate (Fig. 3D), with serrations and/or irregularities present; Size large: 13.3 –15.9 mm. Dorsally olive green, ventrally very dark reddish brown to black in color; abdominal sternite VII’s posterior boundary without a medial rounded posteriad expansion. Mexican and Central American group of species	**22**
–	Elytral apices not truncate, either regularly (Fig. 3A) or flatly rounded/subtruncate (Fig. 3B), or with the sutural angles produced (Fig. 3C)	**23**
22 (21)	Elytral apices with fine serration present, lateral corner of truncation broadly angled, apicolateral margin not sinuate, elytral striae primarily indistinct, visible upon close examination, dense microreticulation covering entirety of elytra and pronotum, producing a polished metal feel, elytra often with violet iridescence; ulimate protarsus ca. 2× as long as wide. Nicaragua to western Panama	***Dineutus truncatus***
–	Elytral apices with serration reduced to small pointed bumps and/or irregularities, lateral corner of truncation distinctly angled, apicolateral margin often faintly sinuate, elytral striae fairly distinct, microreticulation less dense, without violet iridescence; ulimate protarsomere less than ca. 2× as long as wide. Mexico to Honduras and El Savlador	***Dineutus mexicanus* stat. n.**
23 (21)	Elytral apices regularly (Fig. 3A) or flatly rounded/subtruncate (Fig. 3B), without the suture angle produced to point	**24**
–	Elytral apices with the sutural angle more or less strongly produced to a point (Fig. 3C)	**30**
24 (23)	Elytral apices without serrations and or irregularities present apically	**25**
–	Elytral apices with serrations and or irregularities present apically	**29**
25 (24)	Elytra (Fig. 44A) with all 9 striae easily visible, elytral apices regularly to flatly rounded; abdominal sternite VII with the posterior margin with a medial round posteriad expansion (Fig. 44B); regularly elongate oval, large in body size (12.6–14 mm); dorsally olive green in color, ventrally very dark reddish brown to black. Southwestern United States to Honduras	***Dineutus sublineatus***
–	Elytra without all 9 elytral striae easily visible, primarily visible medially on disc, most often indistinct laterally, abdominal sternite VII without a medial round posteriad expansion on the posterior margin	**26**
26 (25)	Larger species, size: 11.5–15.1 mm, typically brown to metallic in color dorsally, body form broadly oval. Species found in the eastern half of the United States	**27**
–	Smaller species, size: under 11 mm, typically olive green to black in dorsal coloration	**28**
27 (26)	Larger species: 13.6–15.1 mm; Venter (Fig. 35B) light in coloration: yellowish; epipleuron with lateral margin darkened; antennal club narrower and more parallel sided, ultimate segment more elongate and pointed apically; elytra (Fig. 35A) without distinct lateral bronzy stripe disappearing apically, instead elytra nearly entirely bronzy; elytral striae faint, but fairly distinct, last lateral striae with punctures distinguishable. Appalachian mountains of northeastern Georgia, and southwestern North and South Carolina. Uncommonly collected Appalachian endemic	***Dineutus robertsi***
–	Smaller species: 11.5–14.6 mm; Venter (Fig. 12B) darker in coloration: reddish brown; antennal club thicker and less parallel sided, ultimate segment much more rounded, less elongate and and much less pointed apically; elytra (Fig. 12A) with lateral bronzy stripe disappearing apically, elytral striae weakly developed; last lateral elytral interval without punctures, rarely with lateral punctures indistinctly present. Eastern United States, southwest to Texas. Fairly widespread and commonly collected	***Dineutus ciliatus***
28 (26)	Body form (Fig. 42A) much more broadly and regularly oval, pronotum with lateral edge obtusely rounded posteriorly to anteriorly, elytral apices much more broadly rounded. Size: 9.1–10.2 mm. Dorsally always olive green. Primarily a Mexican and Central American species, only reaching the most southwestern United States	***Dineutus solitarius***
–	Body form (Fig. 16A) more elongate oval, pronotum with lateral edge more straightly angled posteriorly to apically, elytral apices relatively more narrowly rounded. Size: 8.9–10.1 mm. Frequently very darkly colored near black sometimes olive green in color. Primarily an eastern United States species, extending as far west and south as central Texas	***Dineutus emarginatus***
29 (24)	Elongate oval in body form, attenuated anteriorly (Fig. 40A); elytral apices with strong apicolateral sinuation present, flatly rounded; lateral marginal depression of elytra broad and shallow. Dorsally polished black to bronzy in coloration, with fine microrecticulation present, striae often faint, only evident medially on elytral disc, ventrally often red in coloration, sometimes and infrequently very dark red almost black. Size: 9.6–11.5 mm. Eastern United States and possibly in to northeastern Mexico	***Dineutus serrulatus***
–	More regularly elongate oval body form, less attenuated anteriorly (Fig. 10A); Elytral apices with apicolateral sinuation much more shallow, apices much more roundly angled, not flatly rounded; lateral marginal depression of elytra steep and narrow, most evident at the humeral angle, broadened posteriad. Dorsally more often olive green in coloration, microreticulation of elytra less strongly impressed, elytral striae more frequently distinguishable, even laterally, more often darker brown in coloration. Size: 8.73– 10.60 mm. Eastern United States, possibly in to northeastern Mexico, also found in the Caribbean	***Dineutus carolinus***
30 (23)	Elytral apices without serrations and or irregularities present apically, at most cracks and wrinkles uncommonly present	**31**
–	Elytral apices with serrations and or irregularities present apically and normally. Serrations present as small cuticular cones, irregularities as irregular bumps and peaks	**35**
31 (30)	Venter lighter in color (Fig. 14B): reddish to light yellowish orange. Not dark reddish brown to black and without a metallic hue. Elytral apices with weak to no posterolateral sinuation	**32**
–	Venter darker in color (Fig. 31B): reddish brown to black, often with a metallic hue, only the epipleura of elytra may be lighter in color. Elytral apices with strong posterolateral sinuation present	**33**
32 (31)	Body form (Fig. 6A) narrow and parallel sided; dorso-ventrally convex. Size: 9.8–10.2 mm. Elytral apices without posterolateral sinuation, more regularly and evenly rounded throughout. Northern Florida and southern Georgia and Alabama. Restricted in range and less commonly collected species	***Dineutus angustus***
–	Body form (Fig. 14A) elongate oval, attenuated antteriorly; typically dorso-ventrally depressed posterior to the scutellar area. Size: 10.6–12.8 mm. Elytral apices often with a weak posterolateral sinuation present. Southeastern Canada, Eastern United states, possibly in to Northeastern Mexico. Widespread and fairly common species	***Dineutus discolor***
33 (31)	Epipleura of elytra (Fig. 18B) noticeably lighter in coloration than adjacent thorax and abdomen: yellow to orange in color; elytral apices (Fig. 18A) often steeply angled towards the sutural production; antennae broad with ultimate antennomere rounded; protibiae with distal lateral margin flatly rounded, not prominant. Somewhat smaller in size: 10.3–11.3 mm. Southeastern Canada and the eastern United States	***Dineutus hornii***
–	Epipleura of elytra not noticeably lighter in coloration, sometimes more red in coloration but similar in color to adjacent thorax and abdomen (Fig. 8B). Epipleuron and venter often dark reddish brown to black, and accompanied by a metallic luster; ultimate antennomere more angled; elytral apices more regularly rounded or flatly rounded towards sutural production, not steeply angled	**34**
34 (33)	Larger and noticeably more broader and robust in body form (Fig. 31A); Size: 11.6–11.7 mm. Elytra (Fig. 31A) with striae much more prominent apicomedially on elytral disc, apicolateral sinuation creating a much more prominent and distinct plica; protibiae (Fig. 31B) with distolateral margin prominent. Southeastern Canada and eastern United States	***Dineutus nigrior***
–	Smaller and more narrowly elongate oval in body form (Fig. 8A); Size: 10–11.3 mm. Elytra (Fig. 8A) with the striae more evenly distinct across elytral disc, not prominent apicomedially, apicolateral sinuation of elytra not creating as prominent of a plica; protibiae (Fig. 8B) with distolateral margin not prominent, evenly or flatly rounded. Widespread across southern Canada, and most of the United States, possibly in to northern Mexico. Very common and widespread species	***Dineutus assimilis***
35 (30)	Body form (Fig. 4A) regularly elongate oval, dorsally normally olive green in color, never entirely bronzy metallic, small in size: 8.9–9.1 mm. Elytra (Fig. 4A) without lateral marginal depression evident, laterally strong well impressed reticulation producing a metallic bronzy appearance, striae indistinct, elytral apices with a shallow apicolateral sinuation, regularly rounded, with sutural angle weakly produced. Caribbean endemic	***Dineutus americanus***
–	Body form more elongate oval, attenuated anteriorly, dorsally most often metallic or bronzy in color, sometimes black, rarely olive green, if olive green in color other coloration often present. Continental United States species	**36**
36 (35)	Smaller in size: 9.5–9.9 mm; Body form narrower (Fig. 33A) elongate oval, greatly attenuated anteriorly; elytral apices with irregularities present, not fine or obvious serration; apex of elytra strongly produed, very strong apicolateral sinuation present, producing a strong plica. Ventrally (Fig. 33B) dark brown to black, often with a metallic bronzy hue present, never red in color. Uncommon species known mainly from Texas, Kansas, and south in to Nuevo Leon, Mexico	***Dineutus productus***
–	Larger in size: 9.6–11.4 mm; Body form broader (Fig. 37A) elongate oval, somewhat less attenuated anteriorly; elytral apices with serration present, apices typically appearing more flatly or regularly rounded with apicolateral sinuation more shallow. Ventrally (Fig. 37B) usually red in color, rarely very dark reddish black or brown. Widespread and fairly common species, primarily known from the eastern half of the United States	***Dineutus serrulatus***

#### Dineutus amazonicus

Taxon classificationAnimaliaColeopteraGyrinidae

Hatch, 1930

Dineutus (Cyclinus) amazonicus Hatch, 1930: 16.

##### Type locality.

U.S.A., Arkansas, “Sevier Co., Ark. Saline R. about 18 miles east of DeQueen.”

##### Material examined.

None–see comments below in Discussion.

##### Diagnosis.

Male: unknown.

Female: Length 10 mm, elytral apices regularly oval, serration absent, lateral margins weakly sinuate, profemora of female with apicoventral tooth present running 1/5 its length.

##### Distribution.

Known only from the type locality.

##### Habitat.

Unknown

##### Discussion.

*Dineutus amazonicus* was described from a single female specimen collected in Arkansas during the 1930’s. The species, as described by Hatch (1930), is unique among all other North American *Dineutus* in having females with an sub-apicoventral tooth present on the profemora, running approximately 1/5 its length. In *Dineutus* this is a commonly sexually dimorphic character, with the tooth present only in males. Hatch (1930) indicated the elytra of this species are similar to those of *Dineutus emarginatus*, being regularly rounded and lacking serration.

We were unable to locate the type specimen either at the Smithsonian (F. Shockley, pers. comm.) or Oregon State University (C. Marshall, pers. comm.) where Hatch’s collection was deposited. No specimens in the University of Arkansas collection were identified as *Dineutus amazonicus* (J. Barnes, pers. comm.). It appears that the type may be missing, and that no other published records of this species have occurred since its description in 1930. Although Hatch’s (1930) description at first sounds dubious, as the profemoral sub-apicoventral tooth is in all other species unique to males, Hatch was unlikely to mistake a female gyrinid for a male (Hatch 1925b, 1926), and it therefore seems unlikely that he described a merely aberrant form. For this reason we have included the enigmatic *Dineutus amazonicus*, without having seen neither type nor any other specimens of this species.

The first author recently visited the type locality in an attempt to recollect this species, but was unsuccessful. The locality, Saline River, a large mud bottom river, also had present *Dineutus ciliatus* as described by Hatch (1930), but unsettlingly, the only other species collected at this locality was *Dineutus emarginatus*, the species stated to be most similar to that of *Dineutus amazonicus*. Hopefully in the future the type may be rediscovered or this enigmatic species recollected and its relationship with *Dineutus emarginatus* clarified.

#### Dineutus americanus

Taxon classificationAnimaliaColeopteraGyrinidae

(Linnaeus, 1767)

Figures 4 , 5 , 55

Gyrinus americanus Linnaeus 1767: 568, *Dineutes metallicus*Aubé 1838: 781 [synonymized by Schaum 1848], *Dineutes americanus*: Schaum 1848: 337, Dineutus (Cyclinus) metallicus: Ochs 1926a: 137.

##### Type locality.

“America”.

##### Specimens examined.

8

##### Type material examined.

*Gyrinus americanus* Linnaeus, 1767: syntype (1 ♀ card mounted) “2 [white label typed black ink]// americanus [beige lable, handwritten in black ink]//” examined online through the Linnaen Society of London’s Linnean’s Insect Collection.

*Dineutus metallicus* Aubé, 1838: lectotype, here designated (1 ♀ pinned) “MUSEUM PARIS/ CUBA/ M. De LA SAGRA 764-36 [beige label with thin black border, typed black ink]// green circle [underneath is 764/ 36 handwritten in black ink]// *metallicus au* [beige label, handwritten in black ink, handwriting appears to be Aubé’s, author is partially cut off]// PARATYPE [red label, typed black ink]// LECTOTYPE [red label, typed black ink]//” deposited in the MNHN. Paralectotype (1 ♀ pinned missing right mesothoracic leg past the femur and entire right metathoracic leg) same data as previous except without Aubé’s handwriting label and with “PARALECTOTYPE [red label, typed black ink]//” deposited in the MNHN.

##### Material examined.

**BAHAMAS:** **Great Exuma:** Simons Pt., “23.31.50 75.37.30”, 26.i.1980, leg. S.A. Teale (1 ex. KSEM); **Mayaguana Island:** 3.viii.1963, leg. C.M. Murvosh, BLT (1 ex. FSCA). **CUBA:** Holguín river near Biological Station of PN La Mensura, Piloto, 657 m, 11.v.2013, 20.48640N, 75.779134W, leg. A. Deler-Hernández (1 ex. NMPC). **DOMINCAN REPUBLIC:** **La Altagracia:** Nisibón, 3.v.1978, leg. R.E. Woodruff & G.B. Fairchild, (1 ex. FSCA); **Monte Cristi:** 5 km N Villa Elisa, 10–18.v.1985, leg. E. Giesbert (1 ex. FSCA). **PUERTO RICO:** Almirante Rd., “K.B.Y.”, 9.iii.1935, leg. J.G. Needham (1 ex. MSBA). **U.S.A.:** **The Virgin Islands:** St. Thomas, 27.ii.1925, WIBF 011217 (1 ex. WIBF).

##### Diagnosis.

**Figure 4.**
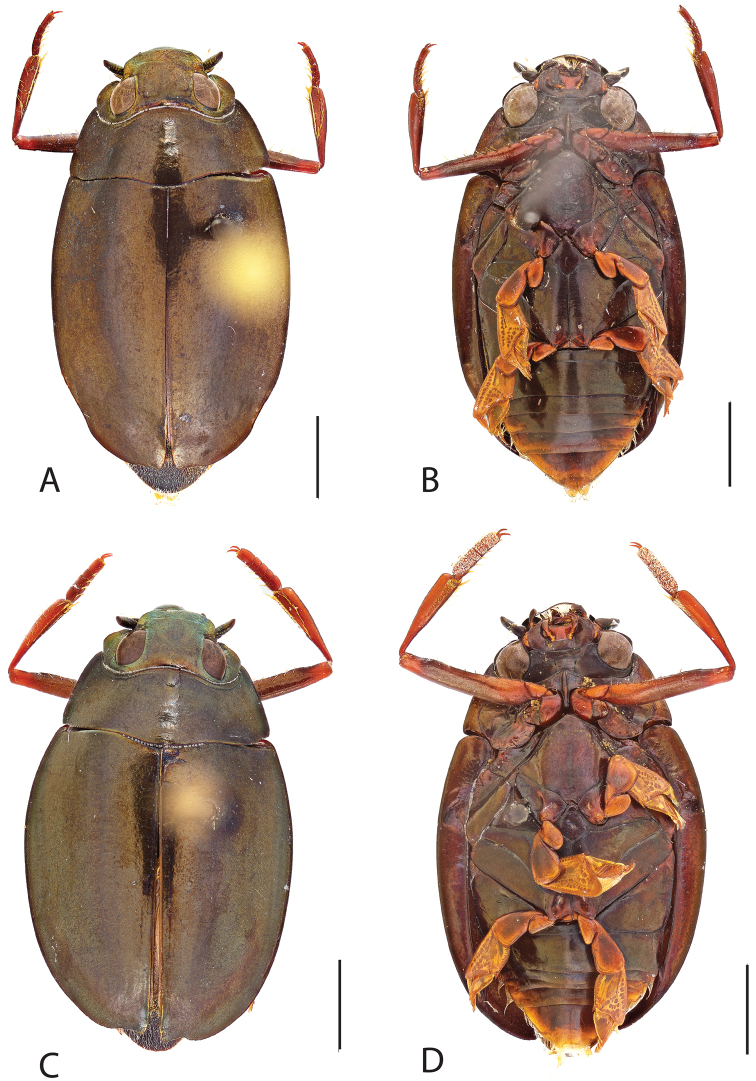
*Dineutus americanus*. **A** ♀ dorsal habitus **B** ♀ ventral habitus **C** ♂ dorsal habitus **D** ♂ ventral habitus. All scale bars ≈ 2 mm.

**Figure 5.**
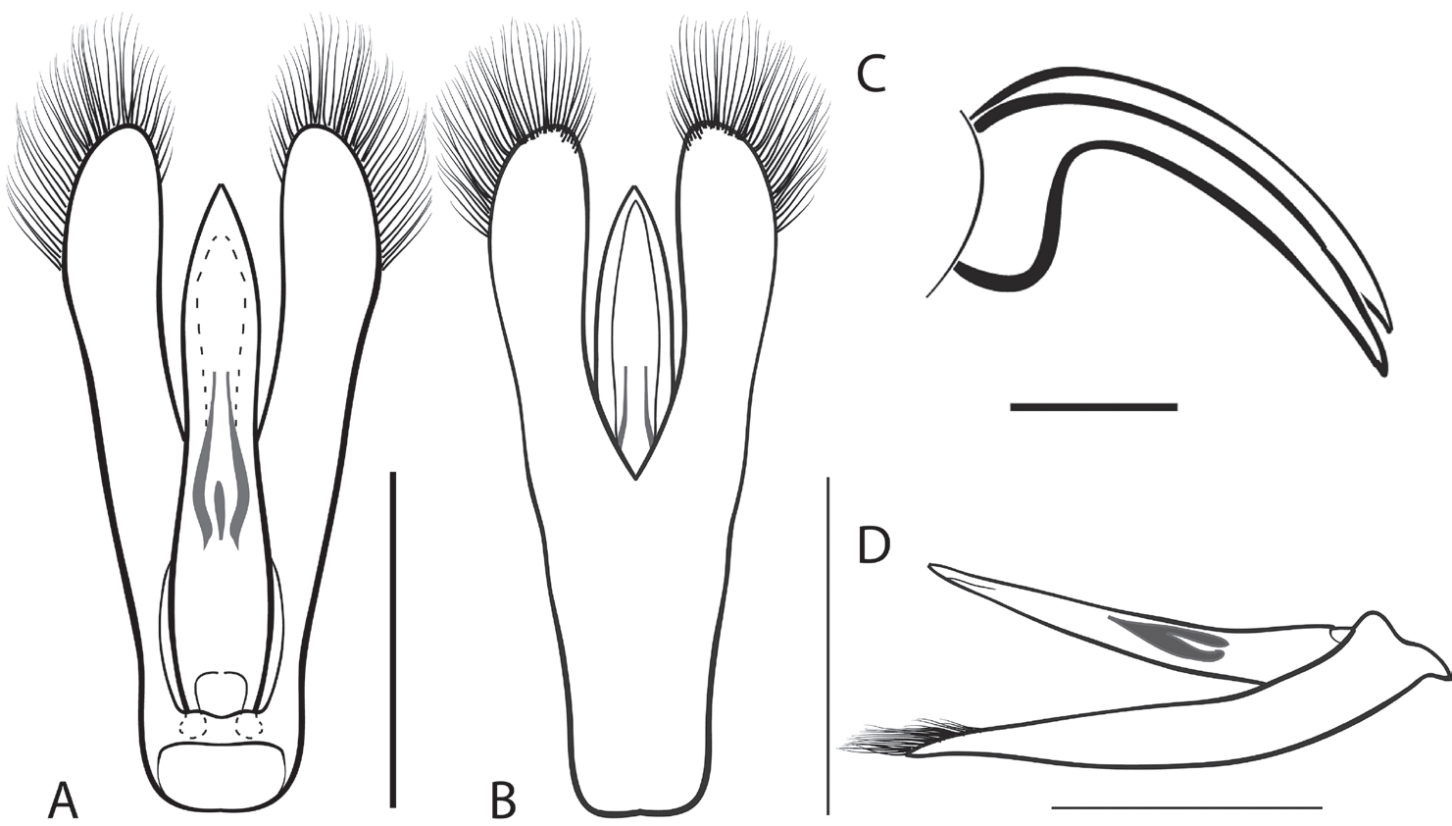
*Dineutus americanus*. **A** aedeagus dorsal view **B** aedeagus ventral view **C** ♂ mesotarsal claws **D** aedeagus lateral view. Scale bar for **C** ≈ 0.10 mm all others ≈ 1 mm.

Male (Fig. 4C–D): Size: 8.7–9.3 mm. Body form elongate oval; elytral apices with sutural angle produced to a point, with serrations and irregularities present apically, elytra with reticulation very strong laterally, medial disc with reticulation sparse or absent, striae often not apparent, lateral marginal depression of elytra not present; profemora with small sub-apicoventral tooth; protibiae club-shaped with apicolateral margin obliquely angled; mesotarsal claws (Fig. 5C) with ventral margin weakly rounded; venter darkly colored, reddish brown to black, weakly metallic, mesothoracic and metathoracic legs usually lighter in coloration, as well as apex of abdomen; Aedeagus (Fig. 5A, B, D) median lobe in dorsal view shorter than parameres, nearly parallel sided, narrowed in apical 1/6; parameres weakly expanded laterally in apical 1/3, medially weakly curved, flatly rounded apically.

Female (Fig. 4A–B): Size: 8.9–9.1 mm. Body form elongate oval; elytral apices with sutural angle produced, with serrations and irregularities present apically, apicolateral sinuation present, elytra with reticulation strongly present laterally, medial disc with reticulation sparse or absent, striae often not present, lateral marginal depression of elytra absent; profemora without sub-apicoventral tooth; protibiae club-shaped with apicolateral angle rounded; venter darkly colored, reddish brown to black, weakly metallic, mesothoracic and metathoracic legs and apex of abdomen usually lighter in coloration.

##### Differential diagnosis.

*Dineutus americanus* is unique among all other species of *Dineutus* in North American in having an elongate oval body form (Fig. 4A, C), elytra without a well defined marginal depression, strong lateral reticulation, with striae mostly absent, the elytral apices with the sutural angle produced to a point with apical serrations and irregularities present, in the male having the profemora with a small sub-apicoventral tooth, protibiae that are club-shaped, and in the form of the male aedeagus (Fig. 5A). This species is also unique in being small in size and endemic to the Caribbean, reaching the Florida Keys (Fig. 55A). The species most similar to *Dineutus americanus* is *Dineutus carolinus*.

Both sexes of *Dineutus americanus* can be distinguished from *Dineutus carolinus* in having the sutural angle of the elytra produced to a point and being generally smaller in size. However, this production can sometimes be highly reduced to only a small point at the sutural angle, especially in males. Both sexes of *Dineutus americanus* can further be separated from *Dineutus carolinus* in lacking the marginal depression of the elytra seen in *Dineutus carolinus*. The absence of the elytral marginal depression is most evident in the humeral region of the elytra, where in *Dineutus carolinus* it is steep and narrow, becoming larger and shallower posteriad. *Dineutus americanus* has the elytra more evenly convex with the lateral portions of the disc with a very strongly impressed reticulation, producing a bronzy green appearance. The elytra also often do not have the striae very apparent due to the strong reticulation, whereas the striae are often faint but evident in *Dineutus carolinus*.

The aedeagus (Fig. 5A) will unambiguously distinguish males of *Dineutus americanus* from *Dineutus carolinus*. In *Dineutus americanus* the median lobe (Fig. 5A) is weakly constricted medially, and only narrowed in the apical 1/6 of its length, whereas that of *Dineutus carolinus* (Fig. 11A) is parallel sided for much of its length, fairly evenly narrowed in the apical 1/3, and is much more narrow overall. The parameres also differ, with those of *Dineutus americanus* more curved along both lateral and medial margins.

Females of *Dineutus americanus* can be distinguished from *Dineutus carolinus* by several of the above described differences, but also the elytra are much more noticeably lacking the marginal depression.

Whereas other small, elongate-oval species may be confused with *Dineutus americanus* (e.g. *Dineutus emarginatus*, *Dineutus solitarius* and possibly *Dineutus assimilis*, since the elytral apices are similar) it should again be noted that *Dineutus americanus* is a Caribbean endemic and its range should limit confusion with all other species except for *Dineutus carolinus* which is also found in the Bahamas (Fig. 53C).

##### Distribution

**(Fig. 55A).** From the Big Pine Key (Florida Keys, U.S.A.) (Wood 1962), through the Caribbean from the Bahamas to Cuba (Blackwelder 1944; Leng and Mutchler 1914a; Peck et al. 1998), Isle de Pinos (Blackwelder 1944), Jamaica (Blackwelder 1994; Leng and Mutchler 1914b), Dominican Republic (Blackwelder 1944), Puerto Rico, St. Thomas, St. John (Blackwelder 1944), Antigua (Blackwelder 1944; Leng and Mutchler 1914a), to Guadeloupe (Blackwelder 1944; Leng and Mutchler 1914a; b; Peck 2011).

##### Habitat.

Lentic species (M. Fikáček pers. com.), also an accidental inhabitant of caves in Cuba (Peck et al. 1998).

##### Discussion.

Many specimens have been misidentified as *Dineutus americanus* due to a long persisting synonymy issue (see the discussion under *Dineutus assimilis*). Mistaken records of *Dineutus americanus* include Ciegler et al. (2003) and Zuellig et al. (2002) for Wyoming and, most likely, the key to larvae provided by Hatch (1927). The true *Dineutus americanus* is only found in the Caribbean and the Florida Keys (Fig. 55A).

##### Type designation.

Aubé (1838) does not give any exact label data, nor does he mention whose collection the types came from. The syntype series was identified by the presence of a disc on the specimen, which was checked with the registrar present at the MNHN to ensure dates were prior to the description by Aubé. The other specimens present either did not provide dates or had dates after Aubé’s (1838) publication. The lectotype (Fig. 51D) here designated was selected as it had a label present with Aubé’s handwriting and identification as “*metallicus*”. We here also confirm that *Dineutes metallicus* is a synonym of *Dineutus americanus* as proposed by Schaum (1848).

#### Dineutus angustus

Taxon classificationAnimaliaColeopteraGyrinidae

LeConte, 1878

Figures 6 , 7 , 52

Dineutes angustus LeConte 1878: 378, [*Dineutes discolor*: Régimbart 1882: 414 proposed synonymy, see discussion], Dineutes discolor var. angustus: Régimbart 1892: 739, Dineutus (Cyclinus) angustus: Hatch 1925b: 447, *Dineutus angustus*: Ciegler et al. 2003: 15.

##### Type locality.

U.S.A., Florida

##### Specimens examined.

15

##### Type material examined.

*Dineutus angustus* LeConte, 1878: syntype (1♀ pinned) “Type/ 6095 [red label, type in typed black ink, 6095 handwritten in black ink]// Fla. [beige label, typed black ink]// *Dineutus angustus*/ Lec. [beige label, handwritten in black ink, handwriting appears to be LeConte’s]//” deposited in the MCZ.

##### Material examined.

**U.S.A.:** **Florida:** Alachua Co., 3.ii.1949, leg. B.W. Cooper (2 ex. FSCA); Columbia Co., O’Leno State Park, 12.ii.1966, leg. F.W. Mead (7 ex. FSCA); Florida: Hillsborough Co., “USF Riverfront”, 21.v.1975, leg. G. Cowden (1 ex. FSCA); Suwannee Co., Branford, 16.vii.1934, leg. J.D. Beamer (4 ex. KSEM).

##### Diagnosis.

**Figure 6.**
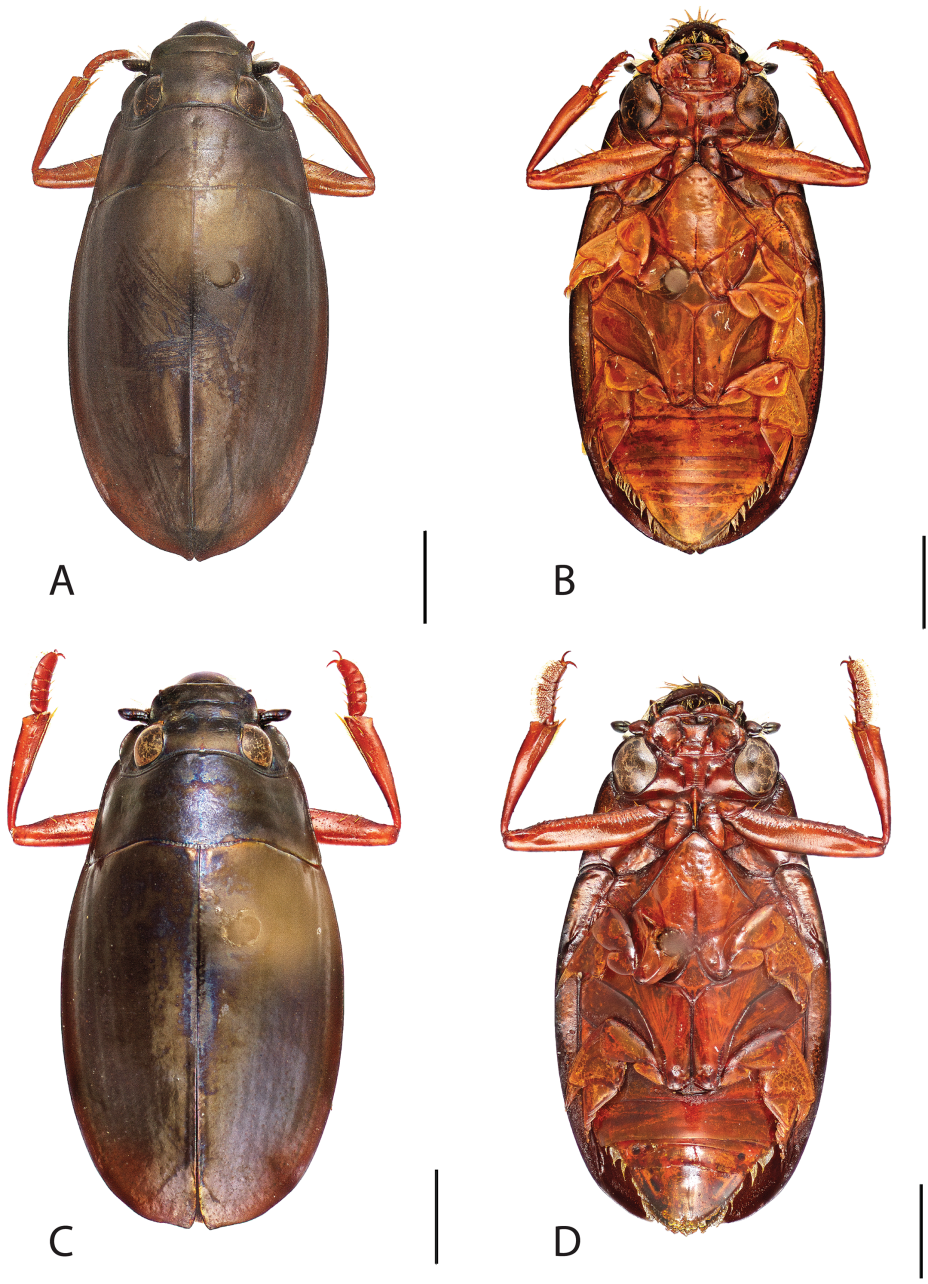
*Dineutus angustus*. **A** ♀ dorsal habitus **B** ♀ ventral habitus **C** ♂ dorsal habitus **D** ♂ ventral habitus. All scale bars ≈ 2 mm.

**Figure 7.**
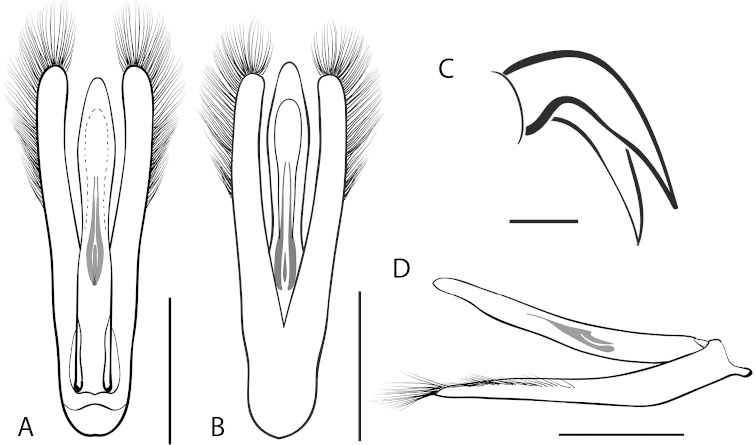
*Dineutus angustus*. **A** aedeagus dorsal view **B** aedeagus ventral view **C** ♂ mesotarsal claws **D** aedeagus lateral view. Scale bar for **C** ≈ 0.10 mm all others ≈ 1 mm.

Male (Fig. 6C–D): Size: 9.4–10.8 mm. Body form very narrowly oval, laterally nearly parallel sided; elytral apices rounded with sutural angle produced into a point, rarely with point reduced and elytra appearing completely rounded, elytral apices without serrations and/or irregularities, elytral striae very faint, most evident medially, elytra laterally with strong reticulation, giving a bronzy appearance, medially replaced by fine microreticulation and fine weakly impressed punctures; profemora with small weakly produced sub-apicoventral tooth; protibia weakly club-shaped; venter lightly colored red to reddish orange; Aedeagus (Fig. 7A, B, D) with median lobe in dorsal view parallel sided basally, weakly constricted medially, apically briefly expanded then narrowed in apical 1/4, apex obtusely rounded, in lateral view median lobe narrowed in apical 1/4, in ventral view sperm-groove parallel sided for near entirety of length, apex broadly rounded, parameres very narrow, shortly constricted in basal 1/5, weakly arced in apical half, apically flatly rounded.

Female (Fig. 6A–B): Size: 9.8–10.2 mm. Body form very narrowly oval, laterally nearly parallel sided; elytral apices roundly angled towards sutural production, with sutural angle produced into a point, apical lateral sinuation often absent, to very weakly developed, elytral apices without serrations and/or irregularities, elytral striae very faint, most evident medially, elytra laterally with strong reticulation, giving a bronzy appearance, medially replaced by fine microreticulation and fine weakly impressed punctures; profemora without sub-apicoventral tooth; protibiae laterally weakly curved, apicolateral margin weakly expanded; venter lightly colored, red to reddish orange.

##### Differential diagnosis.

*Dineutus angustus* is unique among all other North American *Dineutus* in its small size, parallel sided, very narrowly elongate oval body form (Fig. 6A, C), light-colored venter, and somewhat in the shape of the aedeagus (Fig. 7A). This species is most similar to *Dineutus discolor* and it has been debated whether the two are actually distinct species (Régimbart 1892). *Dineutus angustus* of both sexes can be distinguished from *Dineutus discolor* by smaller size, as well as the more parallel-sided and dorsoventrally convex body form. However, the aedeagus of the two species are very similar, with some minor differences. Both aedeagi have the median lobe parallel-sided and very narrow parameres. However, in *Dineutus angustus* the median lobe has a weak constriction medially subtending a slight expansion apically, and a narrowed apical 1/4, whereas the median lobe in *Dineutus discolor* is nearly parallel-sided for most of the length, with only a very weak constriction medially. The parameres of *Dineutus angustus* are more curved in the apical 1/3, and accompanied by a weak constriction in the basal 1/5, in comparison to *Dineutus discolor* which have the parameres much more parallel-sided.

The shape of the apices of the female elytra of *Dineutus angustus* differs from those of *Dineutus discolor*. In *Dineutus angustus* females the apices are broadly angled towards the sutural production and usually lack an apicolateral sinuation. When it is present (rarely), it is only weakly developed. In *Dineutus discolor* females the elytra are regularly rounded to the sutural production, with an apicolateral sinuation present.

##### Distribution

**(Fig. 52D).** U.S.A., Southern Georgia, and Eastern Alabama to northern Florida (Roberts 1895; Wood 1962; Régimbart 1907).

##### Habitat.

Lotic species, seemingly restricted to highly calcareous streams with a basic pH (Young 1954). For more on habitat preference see discussion.

##### Discussion.

Régimbart (1882) originally considered *Dineutus angustus* to be a synonym of *Dineutus discolor*, eventually elevating it to a variation (Régimbart 1892) of *Dineutus discolor* (comparable now to a subspecies), then eventually accepting it as a species distinct from *Dineutus discolor* (Régimbart 1902). We have decided to treat *Dineutus angustus* as a distinct species due to the differences in morphology listed above. The size of the two species in specimens examined for this study only overlap in the extremes, in that the largest specimens of *Dineutus angustus* only just approach the very smallest of *Dineutus discolor*. *Dineutus angustus* was much smaller than any average sized specimen of *Dineutus discolor*, with only a few female specimens of *Dineutus discolor* approaching the size of large *Dineutus angustus* males. Although the aedeagi of both species, which is primarily used here as a delimiter of species boundaries, are very similar, there were some notable differences, even if minor.

Young (1954) noticed that *Dineutus angustus* in Florida appears to be restricted to calcareous streams with a basic pH, while *Dineutus discolor* appears to inhabit streams with a more acidic pH. Young (1954) mentions “intergrade” forms occuring from streams of intermediate pH with both typical forms of *Dineutus discolor* and *Dineutus angustus* present. However, the evidence for the intergrade was in the number of setigerous punctures, which are known to vary among populations, especially with size. Nevertheless, this led Young (1954) to suggest that *Dineutus angustus* may represent the basic stream ecotype of *Dineutus discolor*.

*Dineutus angustus* appears to be very restricted in range so far found only in northern Florida, southeastern Alabama, and southern Georgia (Fig. 52D), records from Virginia are mentioned by Roberts (1895). Similar to Young (1954), we have only seen *Dineutus angustus* from northern Florida, and doubt the other records. Given the extensive range of *Dineutus discolor* and close similarity of that species, we believe it is likely that the two species have been misidentified. *Dineutus angustus* is rarely collected, and not well represented in collections.

#### Dineutus assimilis

Taxon classificationAnimaliaColeopteraGyrinidae

(Kirby, 1837)

Figures 8 , 9 , 52

Cyclinus assimilis Kirby 1837: 78, [*Gyrinus americanus*: Fabiricus 1775: 235 misidentified], [*Cyclous americanus*: Dejean 1833: 58 misidentified], *Dineutes assimilis*: Aubé 1838b: 778, [*Dineutus americanus*: LeConte 1863: 18 misidentified], *Dineutus assimilis*: LeConte 1868: 366, [*Dineutes americanus*: Régimbart 1882: 415 misidentified], *Dineutes assimilis*: Roberts 1895: 285, [*Dineutes americanus*: Régimbart 1907: 138 misidentified], *Dineutes assimilis*: Ahlwarth 1910: 4, [*Dineutes americanus*: Zimmermann 1917: 137 misidentified], *Dineutes assimilis*: Leng and Mutchler 1918: 98, [*Dineutes americanus*: Leng 1920: 82 misidentified], Dineutus (Cyclinus) assimilis: Hatch 1925b: 447 subjective synonym, [Dineutus (Cyclous) americanus: Hatch 1927: 28 misidentified], [*Dineutus americanus*: Leng and Mutchler 1927: 18 misidentified], *Dineutus assimilis*: Ochs 1927b: 36, [*Dineutus americanus*: Leonard 1928: 263 misidentified], *Dineutus assimilis*: Ochs 1930: 135, Dineutus (Cyclinus) assimilis: Hatch 1930: 18, *Dineutus assimilis*: Gordon and Post 1965: 27, [*Dineutus americanus*: Ciegler et al. 2003: 14 misidentified].

##### Type locality.

“Lat. 54°”.

##### Specimens examined.

273

##### Type material.

Not examined. Having examined the type for *Dineutus americanus*, the most confused name with *Dineutus assimilis*, it seems that the identity of *Dineutus assimilis* is secure, so no attempt to loan the type was made.

##### Material examined.

**CANADA:** **Ontario:** Kent Co., Tilbury, vi.1960, leg. K. Stephan (1 ex. FSCA). **U.S.A.:** **Alabama:** Perry Co., Boguechitto Creek,19.vi.1962, leg. F.N. Young, #2047 (1 ex. FSCA); Monroe Co., 10km W Bowles, 31°33.094’N, 86°59.956'W, 11.v.2006, leg. K.B. Miller, KBM1105063 (1 ex. MSBA); **Arkansas:** Benton Co., State Fish Hatchery, 12.iv.1974 (1 ex. FSCA); Conway Co., I-40 Rest Area, 181, 9mi W of State Line, 11.v.1983, leg. L.R. Davis Jr., (1 ex. FSCA); **Florida:** Alachua Co., Gainesville, 17.vi.1947 (1 ex. FSCA); 2 mi NW Gainesville, 20.iv.1974, leg. J.B. Heppner, blacklight (1 ex. FSCA); pond nr. River Styx, 1.viii.1975, leg. J.B. Heppner (1 ex. FSCA); Newman’s Lake, 25.vii.1975, leg. J.B. Heppner (7 ex. FSCA); Columbia Co., O’Leno State Park, 12.ii.1966, leg. F.W. Mead (1 ex. FSCA); Highlands Co., Archbold Biol. Sta., 24.iv.1976, leg. L.L. Lampert Jr. (1 ex. FSCA); Hillsborough Co., 6 mi N Tampa, 3.vi.1978, leg. R. Milton (1 ex. FSCA); Leon Co., SR 373 1 mi SW SR 371, Tallahassee, 16.x.1976 (1 ex. FSCA); Liberty Co., Torreya State Park, 15.v.1970, leg. H. Greenbaum, blacklight/sheet (1 ex. FSCA); Torreya State Park, 25.v.1980, leg. J. Watts, attr. To U.V. (1 ex. FSCA); **Georgia:** Rabun Co., lake in BRM St. Pk., 2.vii.1982, leg. F.N. Young, #2965 (3 ex. FSCA); **Indiana:** Brown Co., nr Crooked Creek, 1.x.1977, leg. F.N. Young (1 ex. FSCA); Crawford Co., Grantsburg, 18.vii.1965, leg. D. Eckert, Blacklight trap (3 ex. FSCA); Ford Co., New Albany, 5.vii.1966, leg. C.E. White, Blacklight trap (2 ex. FSCA); Imperanon Co., 18.viii.1987, leg. N.M. Downie (1 ex. FSCA); Johnson Co., Peoga, 6.v.1966, leg. E. White (7 ex. FSCA); Knox Co., White River, nr Kinora, 12.ix.1964, leg. F.N. Young, #2168 (1 ex. FSCA); Slough along White River, at Edwardsport, 15.iv.1960, leg. F.N. Young, #1687 (1 ex. FSCA); Marion Co., Indianapolis, 15.vii.1963, leg. E. White (1 ex. FSCA); Camp Belzer, BSA, Indianapolis, 12.vii.1966, leg. C.E. White (2 ex. FSCA); Monroe Co., Bloomington, 6.v.1953, leg. R.M. Laycock (1 ex. FSCA); Bloomington, 2.vi.1991, leg. F.N. Young, BLT (1 ex. FSCA); Posey Co., Hovey Lake, 15.viii.1965, leg. C.E. White, Blacklight trap (3 ex. FSCA); **Iowa:** Boone Co., Ledges State Park, 2.v.1955, leg. M.D. Huffman (3 ex. FSCA); same as previous except: 2.v.1985 (1 ex. FSCA); same as previous except: 1.x.1961, leg. J.J. Dinsmore (3 ex. FSCA); same as previous except: 1.xi.1961 (1 ex. FSCA); Linn Co., Cedar Rapids, “1536” (1 ex. MTEC); Plymouth Co., Le Mars, 12.v.1965, leg. B. Perrill (1 ex. FSCA); Story Co., Ames, 15.iv.1930, leg. H.B. Mills (2 ex. MTEC); same as previous except: 24.iv.1939, leg. C. Haight (3 ex. MTEC); **Kansas:** Douglas Co., Lawrence, 24.ix.1921, leg. C. Brown (2 ex. FSCA); **Maryland:** Kent Co., Chestertown, 13.v.1969, leg. T.E. Rogers (1 ex. FSCA); Montgomery Co., Seneca, 27.v.1951, leg. G.H. Nelson (2 ex. FSCA); Prince George’s Co., College Park, 17.iv.1948, leg. B.K. Dozier, in pond (2 ex. FSCA); College Park, 29.iv.1948, leg. H.L. Dozier (1 ex. FSCA); College Park, 14.x.1948, leg. R. Mansueti (1 ex. FSCA); College Park, 25.iv.1953, leg. G.H. Nelson, to light (1 ex. FSCA); **Massachusetts:** Norfolk Co., Dedham, 11.vi.1920 (1 ex. FSCA); **Minnesota:** Morner Co., nr Grand Meadow, roadside park, 18.viii.1965, leg. R.H. Arnett, in dammed pond (13 ex. FSCA); Morula Co., nr Grand Meadow, 18.viii.1965, leg. R.H. Arnett Jr., roadside park in damned pond (40 ex. FSCA); **Mississippi:** Marshall Co., Byhalia, 10.v.1983, leg. L.R. Davis Jr., 11:15PM at lights (1 ex. FSCA); **Missouri:** Calloway Co., 3 mi W Portland on Rt 94, temp pool, 12.vii.1973, leg. S.O. Swadener, Lot No.730712A (1 ex. FSCA); Carter Co., Van Buren, 22.vi.1955 (2 ex. FSCA); Clay Co., nr Missouri River, E of Birmingham, 2.v.1968, leg. J.R. Heitzman (1 ex. FSCA); Dent Co., Montauk St. Pk., 18.v.1978, leg. S.O. Swadener, Lot No.730518-A (1 ex. FSCA); Douglas Co., Cartwright Tree Farm, 10 mi E of Cabool, walnut, apple, & peach orchards, Deciduous Ozark Forest, open fields & Indian Creek, 14.vii.1991, leg. H.M. Webber, at U.V. light (1 ex. FSCA); Franklin Co., 3.vii.1978, leg. K. Jackson, in lake (1 ex. FSCA); same as previous except: at light (1 ex. FSCA); Green Co., Willard, 6.vii.1929, leg. K. Nime, pond (1 ex. FSCA); Jackson Co., Adair Park, Independence, 3.v.1968, leg. J.R. Heitzman (1 ex. FSCA); Vernon Co., Nevada, 10.v.1964, leg. D&J. McReynolds, (1 ex. FSCA); same as previous except: 8.vi.1964 (1 ex. FSCA); same as previous except: 9.v.1972, leg. J.W. McReynolds (1 ex. FSCA); **Montana:** Wibaux Co., pond 30 mi N Wibaux, 27.vii.1990, leg. D.L. Gustafson (2 ex. MTEC); **Nebraska:** Cherry Co., McKelvie Nat’l For., 30 mi WSW Valentine, 9.vii.1998, leg. A. Ramsdale, at blacklight, at forest margin near sand hills prairie, night (1 ex. MTEC); **New Jersey:** Gloucester Co., Paulsboro, 11.vi.1961, leg. H.L. Dozier (1 ex. FSCA); Ocean Co., Lakehurst, 1.viii.1960, leg. H.L. Dozier (1 ex. FSCA); **New York:** Schuyler Co., Texas Hollow State Wildlife Area, 1.ix.1999, leg. K.B. Miller (5 ex. MSBA); Westchester Co., White Plains, 14.v.1922, leg. E.H.P. Squire, (1 ex. FSCA); same as previous except: 20.v.1923 (1 ex. FSCA); same as previous except: 31.v.1923 (3 ex. FSCA); 10.vi.1923 (4 ex. FSCA); **North Carolina:** Jackson Co., Balsam, 6.v.1965, leg. W. Rosenberg (1 ex. FSCA); Macon Co., Watuaga Valley, 28.viii.1987, leg. F.N. Young, #3247A (1 ex. FSCA); Wake Co., Raleigh, Yates Pond, 12.ix.1970, leg. L.L. Lampert (8 ex. FSCA); **North Dakota:** Ransom Co., McLeod, 25.vii.1960, leg. J. Onsager (1 ex. MTEC); **Ohio:** Delaware Co., 4.x.1958, leg. E.I. Hazard (2 ex. FSCA) Franklin Co., Columbus, 20.v.1984, leg. M.A. Ivie (1 ex. MTEC); same as previous except: 1.viii.1985, leg. R.S. Miller (9 ex. MTEC); same as previous except: 7.viii.1985 (18 ex. MTEC); Columbus, Mere Pond, 23.viii.1985, leg. R.S. Miller (32 ex. MTEC); Lucas Co., nr West Toledo, Schwamberger Prarie, 10.vi.1984, leg. J.B. Stribling, UV light (1 ex. MTEC); Williams Co., Mudlake, 18.vii.1984, leg. J.A. Shuey (1 ex. MTEC); Muskingum Co., Zanesville, 2.v.1920, leg. A.E. Miller (3 ex. FSCA); Ross Co., Chillicothe, 1.viii.1992, leg. A.E. Miller (3 ex. FSCA); **Oklahoma:** Catoosa, 26.iv.1938, leg. E.K. Waering (1 ex. FSCA); same as previous except: 26.iv.1939 (1 ex. FSCA); same as previous except: 27.iii.1939 (1 ex. FSCA); Comanche Co., Ft. Still, 20.vi.1974, leg. T.E. Rogers (2 ex. FSCA); Latimer Co., 5 mi W Red Oak, v.1980, leg. K.H. Stephan (1 ex. FSCA); Latimer Co., 5 mi W Red Oak, ix.1980, leg. K.H. Stephan (10 ex. FSCA); Payne Co., Stillwater, 14.vii.1976 (1 ex. FSCA); nr Lake Carl Blackwell, 16.viii.1976 (1 ex. FSCA); **South Carolina:** Greenville Co., Greenville, 9.ix.1954, leg. H.L. Dozier (1 ex. FSCA); Newberry Co., 12.v.1968, leg. L.L. Lampert (1 ex. FSCA); Newberry Co., Jalapa, 24.iv.1973, leg. L.L. Lampert, light (1 ex. FSCA); **Tennessee:** Cuba, 25.v.1964, leg. K. Stephan (1 ex. FSCA); Obion Co., Reelfoot Lake S. P., 1/2 mi SE of Samburg, 4.vii.1983, leg. C.P. Withrow (2 ex. MTEC); **Texas:** Crosby Co., White River Lake, SW Area, 24.ix.1997, leg. Wappes & Huether (1 ex. FSCA); Runnels Co., Miles, 28.vi.1939, leg. H. Wilee Jr. (1 ex. FSCA); Waxahachie Co., Atlanta, 26.v.1964, leg. K. Stephan (1 ex. FSCA); **Virginia:** Rockingham Co., Craney Island, 7.x.1984, leg. C.L. Staines Jr. (1 ex. FSCA); **West Virginia:** Pocahontas Co., Cranberry Glades, 1213-1219 m, 27.vi.1967, leg. H.V. Weems, blacklight (1 ex. FSCA); **Wisconsin:** Dane Co., 18.v.1952, leg. D.H. Habeck (1 ex. FSCA).

**No locality information:** “Station.”, 8.v.1901, “Hatch Ex.” (1 ex. MTEC); “E-2” (1 ex. FSCA).

##### Diagnosis.

**Figure 8.**
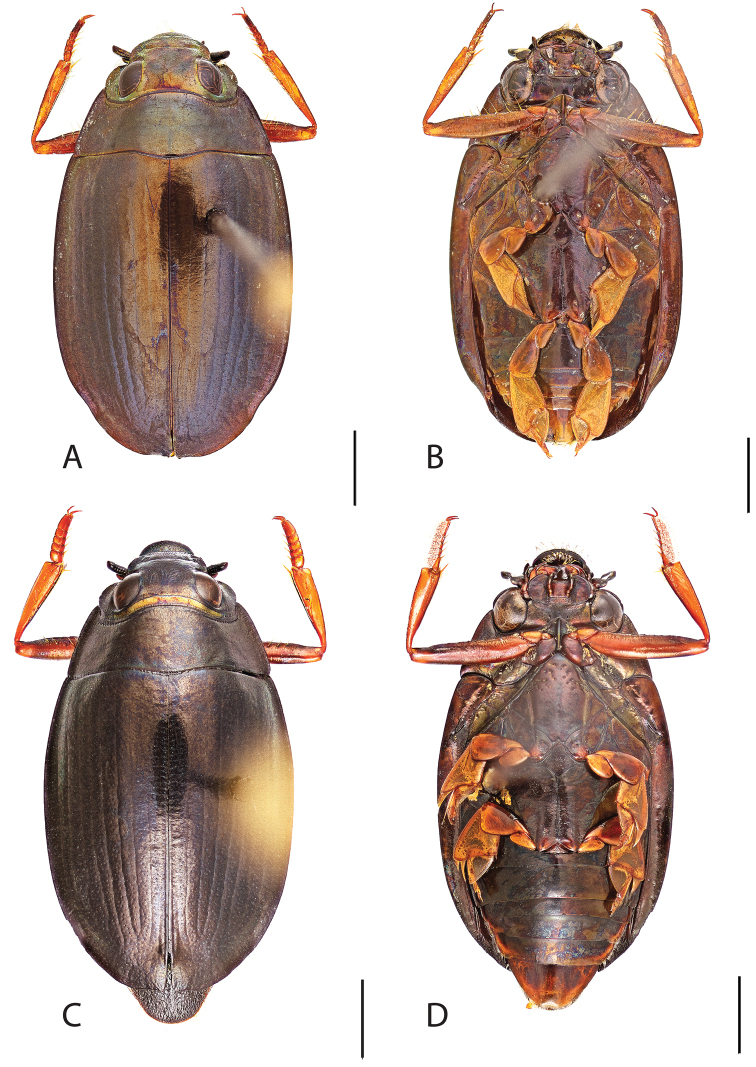
*Dineutus assimilis*. **A** ♀ dorsal habitus **B** ♀ ventral habitus **C** ♂ dorsal habitus **D** ♂ ventral habitus. All scale bars ≈ 2 mm.

**Figure 9.**
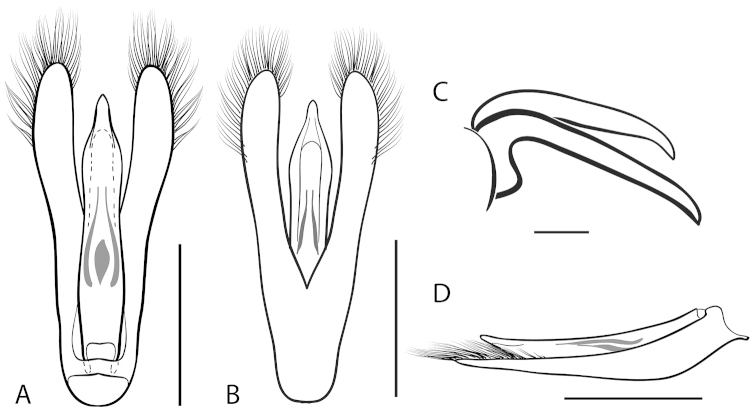
*Dineutus assimilis*. **A** aedeagus dorsal view **B** aedeagus ventral view **C** ♂ mesotarsal claws **D** aedeagus lateral view. Scale bar for **C** ≈ 0.10 mm all others ≈ 1 mm.

Male (Fig. 8C–D): Size: 9.93–11.1 mm. Body form narrowly oval; elytral apices with sutural angle produced into a point, rarely with point reduced and elytra appearing completely rounded, elytral striae faint basally becoming more evident apically and laterally; profemora without sub-apicoventral tooth; protibia wedgeshaped, without distolateral margin produced; mesotarsal claws similar in size, with ventral margin straight; venter darkly colored, reddish brown to black often with a metallic shine present, epipleura similarly colored as thorax and abdomen; Aedeagus (Fig. 9A, B, D) median lobe in dorsal view acuminate in apical 1/5, apex narrowed and shortly rounded, in lateral view apex of median lobe barely curved dorsally, in ventral view sperm-groove parallel sided for near entirety of length, apex flatly rounded, parameres very weakly rounded laterally in apical 1/3.

Female (Fig. 8A–B): Size: 10–11.3 mm. Body form narrowly oval; elytral apices produced and rounded, with sutural angle produced into a point, apicolateral sinuation strong, elytral striae faint basally, becoming more evident apically and laterally; profemora without sub-apicoventral tooth; protibiae laterally weakly curved, distolateral margin not expanded; venter darkly colored, reddish brown to black, often with metallic luster, epipleura similarly colored as thorax and abdomen.

##### Differential diagnosis.

*Dineutus assimilis* is unique among all North American species of *Dineutus* in the narrowly elongate oval body form, both sexes with the elytral apices more or less regularly rounded with the sutural angles produced, males with the profemora without a sub-apicoventral tooth, a wedge shaped protibia, mesotarsal claws that are similar in shape with their ventral margins straight, and the form of the aedeagus. The species most similar to *Dineutus assimilis* are *Dineutus hornii* and *Dineutus nigrior*, especially in the form of the females. Males of *Dineutus assimilis* can be separated from *Dineutus hornii* by the sutural angle of the elytra being regularly produced to a point, although rarely some individuals have the point reduced giving the elytra a regularly rounded look similar to those of *Dineutus hornii*. In such cases, specimens of *Dineutus assimilis* can be distinguished by the elytral epipleura being colored similarly to the adjacent thoracic and anterior abdominal ventrites (Fig. 8D), in contrast with *Dineutus hornii* in which the epipleura is different in color (much lighter) from the rest of the venter (Fig. 18D). The mesotarsal claws can also be used to separate males of *Dineutus assimilis* (Fig. 9C) from *Dineutus hornii* (Fig. 19C). In *Dineutus assimilis* the ventral margins of the claws are straight, whereas in *Dineutus hornii* they are very shallowly curved. *Dineutus assimilis* also have the ultimate antennomere angled, whereas in *Dineutus hornii* the antennal flagellum is short and thick, with the ultimate antennomere rounded. Males of *Dineutus assimilis* can be separated from *Dineutus nigrior* in their smaller size (9.9–11.3 mm), more narrow body form, and, most readily, by the mesotarsal claws which are similar in size with straight ventral margins, whereas in *Dineutus nigrior* the mesotarsal claws are distinctly asymmetrical with the anterior claw larger than the posterior and both having the ventral margins shallowly curved. The most reliable way to distinguish between *Dineutus assimilis*, *Dineutus hornii* and *Dineutus nigrior* is the aedeagus. The median lobe of the aedeagus of *Dineutus assimilis* (Fig. 9A) is acuminate in the apical 1/5. The aedeagi of both *Dineutus hornii* and *Dineutus nigrior* are more elongate and parallel sided with the median lobe apically narrowed but not acuminate.

Females of *Dineutus assimilis* are more difficult to differentiate from *Dineutus hornii* and *Dineutus nigrior*. Females of *Dineutus assimilis* differ from those of *Dineutus hornii* in the epipleura color being similar to the adjacent thoracic anterior abdominal ventrites, whereas in *Dineutus hornii* the epipleura are lighter in color than the surfaces of the thorax and abdomen (Fig. 18B). The elytral apices of the females of *Dineutus assimilis* are more similar to those of *Dineutus nigrior*. In general *Dineutus assimilis* is smaller (10–11.3 mm) than *Dineutus nigrior*, and also more narrowed in body form. The most reliable way to separate *Dineutus assimilis* from *Dineutus nigrior* is the distal lateral margin of the protibiae. In *Dineutus assimilis* the distal lateral margin is not produced laterally (Fig. 8B), but continuously weakly curved, whereas in *Dineutus nigrior* the margin is laterally weakly expanded (Fig. 31B).

##### Distribution

**(Fig. 52A).** Southern Canada from British Columbia to Nova Scotia (Majka 2008; Roughley 1991; Webster and DeMerchant 2012), and east of the Rocky Mountains in the U.S.A. as far south as Texas and Northern Florida (Epler 2010; Ferkinhoff and Gunderson 1983; Folkerts 1978; Gordon and Post 1965; Hilsenhoff 1990; Malcolm 1971; Régimbart 1907; Roberts 1895; Sanderson 1982; Whiteman and Sites 2003; Wood 1962).

##### Habitat.

This is most widespread and commonly encountered species of *Dineutus* in North America, occupying both lotic and lentic habitats (Woods 1962; Hilsenhoff 1990; Whiteman and Sites 2003). The first author has collected this species in a secondary growth deciduous forest, Baldwin Woods, near Baldwin, Kansas, in a large forest pond with a muddy bottom, with plenty of leaf detritus and several fallen emergent trees. Here *Dineutus assimilis* was found in large numbers near the shore, with others often exploring deeper regions of the pond. In New Brunswick, Canada, *Dineutus assimilis* was collected in similar habitats, the margins of ponds with emergent vegetation within forests composed of red oak and red maple (Webster and DeMerchant 2012). Webster and DeMerchant (2012) also found *Dineutus assimilis* in black spruce and tamarack bogs. This species has also been collected in other lentic situations from the Prairie Region of Missouri, where Whiteman and Sites (2003) found specimens associated with the following plant taxa: *Brasneia*, *Ceratophyllum*, *Juncus*, *Lespedeza*, *Ludwigia*, *Polygonum*, *Potamogeton*, *Sagittaria*, *Typha*, Cyperaceae, and Poaceae. This species also comes to ultraviolet light (Webster and DeMerchant 2012).

##### Discussion.

*Dineutus assimilis* is the most commonly encountered species of *Dineutus* in North America, and has one of the largest ranges of any North American species (Fig. 52A), often erroneously listed under the name *Dineutus americanus*, even recently (e.g. Ciegler et al. 2003). As early as 1927, Ochs (1927b) had the types of *Dineutus americanus* examined by an associate in London, and determined unambiguously that *Dineutus americanus* is the Caribbean species, with *Dineutus assimilis* found in mainland North America. We have again confirmed, after examination of the syntype of *Dineutus americanus*, that *Dineutus assimilis* is not *Dineutus americanus* of Linnaeus. It is likely all records of *Dineutus americanus* from areas not in the Caribbean or the Florida Keys actually represent *Dineutus assimilis*. An example of references clearly referring to *Dineutus assimilis* is Zuellig et al. (2002), who recorded “*Dineutus americanus*” from Wyoming. Hatch’s (1927) key to the larvae of “*Dineutus americanus*” most likely refers to *Dineutus assimilis* as well.

The juvenile stages have been formally described and illustrated by Wilson (1923) and sparse life history information is available in Smith (1926) and Istock (1966; 1967).

#### Dineutus carolinus

Taxon classificationAnimaliaColeopteraGyrinidae

LeConte, 1868

**Figure 10.**
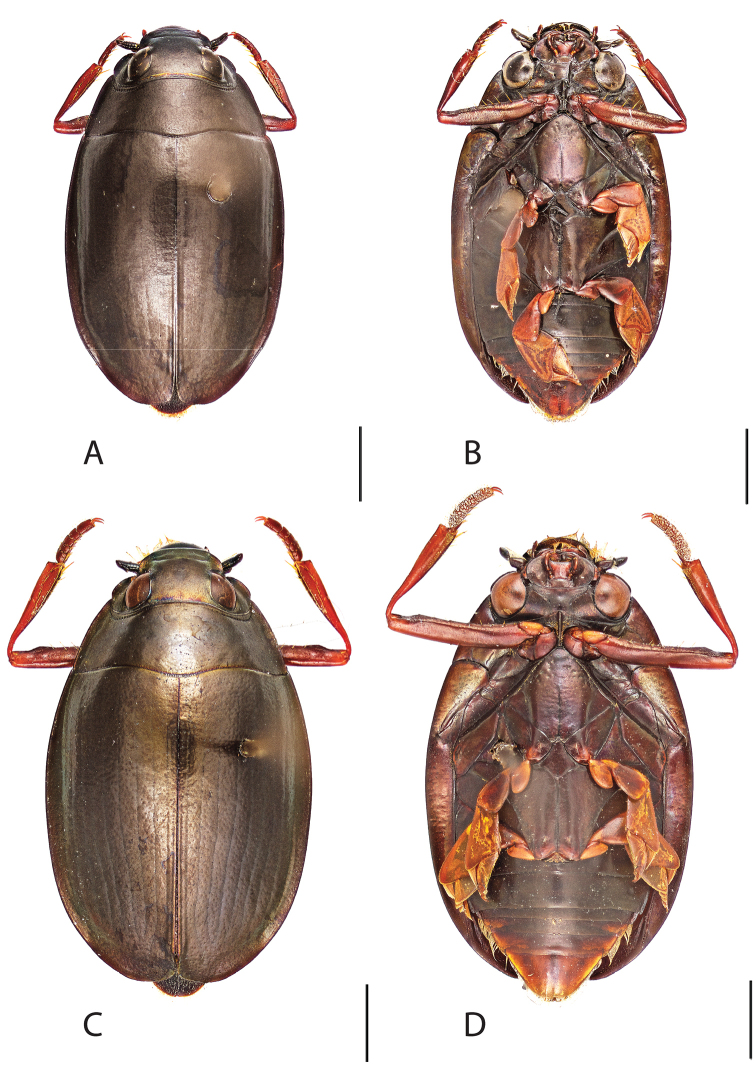
*Dineutus carolinus*. **A** ♀ dorsal habitus **B** ♀ ventral habitus **C** ♂ dorsal habitus **D** ♂ ventral habitus. All scale bars ≈ 2 mm.

**Figure 11.**
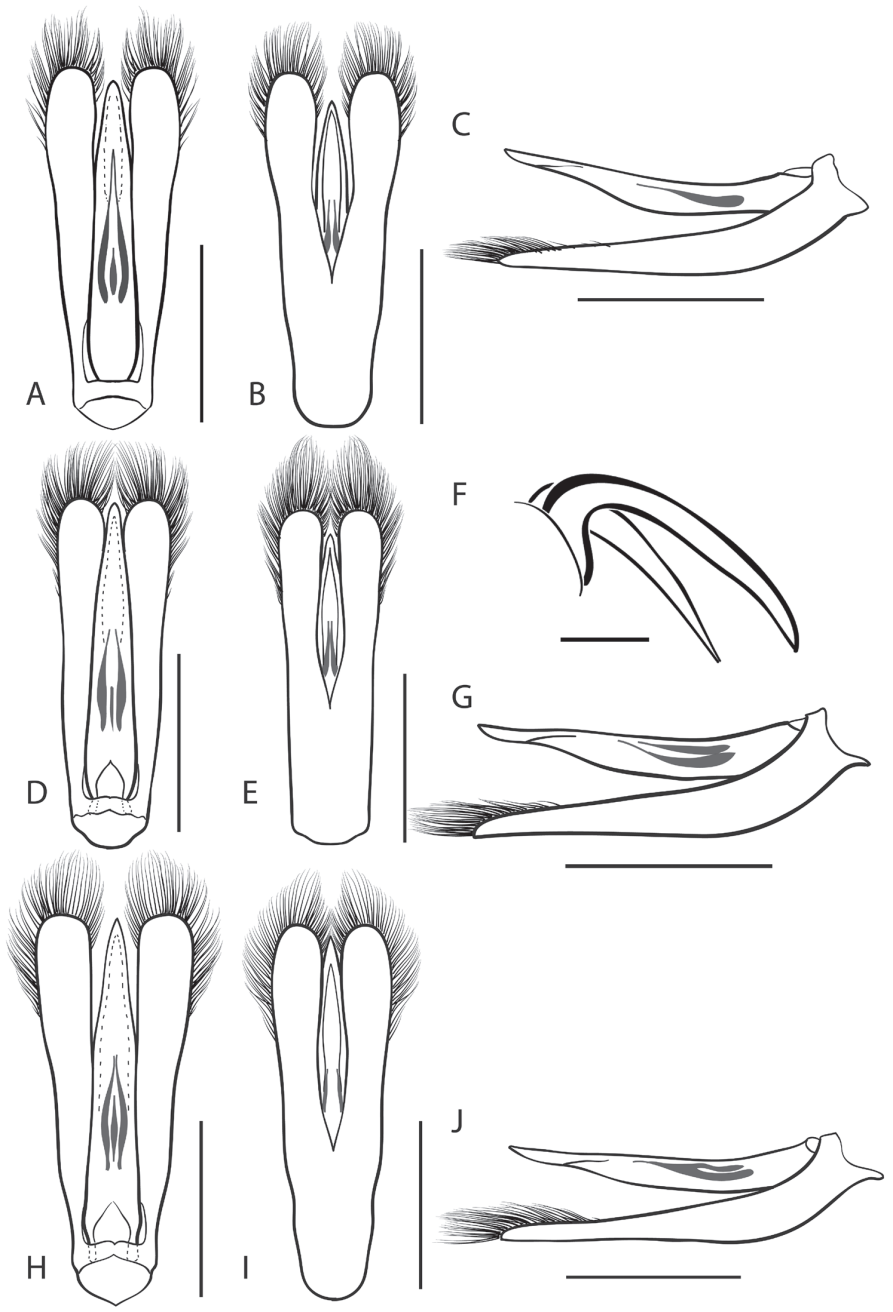
*Dineutus carolinus*. Bahamas specimen aedeagus **A** dorsal view **B** ventral view **C** lateral view; Florida specimen aedeagus **D** dorsal view **E** ventral view **F** ♂ mesotarsal claws **G** aedeagus lateral view, Texas specimen aedeagus **H** dorsal view **I** ventral view **J** lateral view. Scale bar for **F** ≈ 0.10 mm all others ≈ 1 mm.

Figures 10 , 11 , 53

Dineutus carolinus LeConte 1868: 366, Dineutes emarginatus var. carolinus: Régimbart 1882: 418, *Dineutes carolinus*: Roberts 1895: 283, Dineutus (Cyclinus) carolinus mutchleriOchs 1924: 1 **syn. n.**, Dineutus (Cyclinus) emarginatus carolinus: Ochs 1926: 136, Dineutus (Cyclinus) carolinus: Ochs 1929: 125, *Dineutus carolinus*: Ciegler et al. 2003: 15.

##### Type locality.

South Carolina.

##### Specimens examined.

199

##### Type material examined.

*Dineutus carolinus* LeConte, 1868: syntype (♀ pinned) “[orange disc]// Type/ 6093 [orange label Type typed in black ink, 6093 handwritten in black ink]// Dineutus carolinus/ Lec. [white label handwritten in black ink, handwriting appears to be LeConte’s]// LECTOTYPE/ Dineutus carolinus/ Desig. R.P. Withington III/ 1998 [red label, handwritten in black ink]//” deposited in MCZ.

*Dineutus carolinus mutchleri* Ochs, 1924: holotype (♂, pinned) “Nassau, Bahamas, V-VI-1917/Wm. M. Mann Collector//Amer. Mus. Nat. Hist., Dept. Invert. Zool., No.28070/HOLOTYPE/Dineutus carolinus LeC.subsp. mutchleri OchsType ! ♂/Dineutus carolinus LeConte 1868. Det: L. Cook 2005” AMNH type catalogue No. 433.

##### Material examined.

**BAHAMAS:** **Eleuthera Island:** Rainbow Bay, 4.vii.1989, leg. D.B. & R.W. Wiley (1 ex. FSCA); Rainbow Bay, 21-28.iv.1984, leg. J.R. Wiley (6 ex. FSCA); **Grand Bahamas Island:** Freeport, 21.xii.1984, leg. S. Dunkle (4 ex. FSCA); **Great Exuma:** Simons Pt., “23.31.50-75.47.30”, 13.i.1980, leg. S.A. Teale (1 ex. KSEM); same as previous except: 21.i.1980 (1 ex. KSEM); same as previous except: 26.i.1980 (3 ex. KSEM); **New Providence:** 1.viii.1959, leg. J.B. Rearle (1 ex. FSCA); **South Bimini:** 14.vi.1967, leg. B.K. Dozier (2 ex. FSCA). **U.S.A.:** **Arkansas:** Washington Co., Devil’s Den State Park, pond, 6.viii.1975, leg. D. Huggins, SEMC 1054952 (1 ex. KSEM); **Florida:** Alachua Co., 10.ii.1949, leg. S.B. Mansell (5 ex. FSCA); same as previous except: 19.ii.1949, leg. B.W. Cooper (1 ex. FSCA); same as previous except: 8.iv.1949, leg. B.W. Cooper (2 ex. FSCA); same as previous except: 8.iv.1949, leg. E.H. McConkey (7 ex. FSCA); same as previous except: 19.iv.1949, leg. W.L. Jennings (1 ex. FSCA); same as previous except: 17.ii.1950, leg. O.G. Fogle (1 ex. FSCA); same as previous except: 15.iv.1950, leg. E.W. Michelson (1 ex. FSCA); same as previous except: 18.iv.1951, leg. J.E. Brogdan (1 ex. FSCA); same as previous except: x.1960, leg. S. Cabler (1 ex. FSCA); same as previous except: 4.ix.1989, leg. M.L. May (1 ex. FSCA); Gainesville, 20.iii.1987, leg. Willis, ACC.76-77; ACC.79-83; ACC.86; ACC.88 (9 ex. FSCA); Gainesville, 21.iii.1978, leg. L.R. Davis Jr., (2 ex. FSCA); same as previous except: 5.v.1978, leg. M.C. Thomas (1 ex. FSCA); same as previous except: 11.v.1978 (1 ex. FSCA); same as previous except: 2.vi.1978 (1 ex. FSCA); same as previous except: 18.iv.1983, leg. N. Hastettle (1 ex. FSCA); same as previous except: 5.vi.1983, leg. L.R. Davis Jr. (1 ex. FSCA); 5.vi.1959, leg. H.V. Weems Jr., taken at light (1 ex. FSCA); Gainesville, Beville Hts., 5.vii.1980, leg. L.A. Stange, Blacklight trap (1 ex. FSCA); NW Gainesville, 27.iii.1974, leg. J.B. Heppner, at blacklight (7 ex. FSCA); Gainesville, 3517 NW 10th Ave., 1.vi.1993, leg. R.E. Woodruff, Blacklight trap (8 ex. FSCA); 2 mi NW Gainesville, 20.iv.1974, leg. J.B. Heppner, blacklight (4 ex. FSCA); 6 mi SW Gainesville, 4.xi.1974, leg. L.R. Davis Jr., BLT (1 ex. FSCA); same as previous except: 5.xi.1974 (2 ex. FSCA); same as previous except:17.xi.1974 (1 ex. FSCA); same as previous except: 19.xi.1974 (2 ex. FSCA); Bainsville, 24.iii.1983, leg. C. Blare (1 ex. FSCA); Hatchet Creek, 25.vii.1975, leg. J.B. Heppner (3 ex. FSCA); O’Leno State Park, 8.viii.1997, leg. J. Cicero (3 ex. FSCA); Hogtown Creek, 28.vi.1975, leg. J.B. Heppner (1 ex. FSCA); Clay Co., Hibernia, 7.viii.1939, leg. J.D. Beamer (1 ex. KSEM); Collier Co., Copeland, 27.iv.1972, leg. H. Flaschka (1 ex. FSCA); Naples, 27.iv.1984, leg. R.A. Belmont, u.v. blacklight trap (1 ex. FSCA); Naples, 13.v.1984, leg. R.A. Belmont (4 ex. FSCA); Naples, 15.xii.1985, leg. R.S. Miller (1 ex. MTEC); Columbia Co., O’Leno State Park, 12.ii.1966, leg. F.W. Mead (1 ex. FSCA); O’Leno State Park,11.xii.1954, leg. C.N. Patton (1 ex. FSCA); Dade Co., nr Everglades Nat. Prk., fresh water, 7.v.1955, leg. D.K. Caldwell, K13 (8 ex. FSCA); Dade Co., Camp Mahachee, nr. Matheson Hammock, 27.iv.1983, leg. M.C. Thomas & L. Parker, Blacklight trap (2 ex. FSCA); Homestead, 28.v.1958, leg. D.O. Wolfenbarer, Blacklight trap (1 ex. FSCA); 25 m W Miami, 23.vii.1934, leg. P. McKinstry (1 ex. KSEM); 25 m W Miami, 23.vii.1934, leg. M.E. Griffith (1 ex. KSEM); Ross-Castello Hammock, 1.v.1968, leg. R.H. Arnett, Blacklight trap (1 ex. FSCA); Miami Springs,15.vi.1961, leg. C.E. White (1 ex. FSCA); Dixie Co., Horseshoe Beach, 28.vii.1985, leg. P. Van Mierop, pond (1 ex. FSCA); Escambia Co., Pensacola, 17.v.1960, leg. R.E. Woodruff, col. At light (1 ex. FSCA); Gadsden Co., Rocky Comfort Creek, 4 mi S Hwy 268,13.v.1980, leg. G.B. Wibmer, uv light (1 ex. FSCA); Gulf Co., St. Joseph T.H. Stone Memorial State Park, 14.vi.1969, leg. H.V. Weems Jr. (1 ex. FSCA); Henderson Co., Fletcher, 10.vii.1979, leg. L.L. Lampert, U.V. Light (1 ex. FSCA); Hernando Co., Weekiwachee Spring, 3.vi.1954, leg. W.C. Sloan, Sta.4 (2 ex. FSCA); Highlands Co., Archbold Biol. Sta., 7.iv.1975, leg. L.L. Lampert, UVL (1 ex. FSCA); same as previous except: 18.xi.1982, leg. L.L. Lampert Jr., UVL (1 ex. FSCA); same as previous except: 19.iv.1976, leg. L.L. Lampert Jr. (1 ex. FSCA); same as previous except: 23.vi.1988, leg. K.E.M. Galley, at blacklight SE tract (2 ex. FSCA); same as previous except: 19.iii.1968, leg. C.E. White, at blacklight trap (2 ex. FSCA); same as previous except: 10.ii.1993, leg. M.J. Rothschild (1 ex. FSCA); Highlands Co., Highlands Hammock State. Prk., 9-10.viii.1983, leg. K.W. Vick, Blacklight trap (1 ex. FSCA); same as previous except: 11.viii.1983 (1 ex. FSCA); Hillsborough Co., Hillsborough RI St. Pk., 9-10.viii.1983, leg. K.W. Vick, Blacklight trap (5 ex. FSCA); Plant City, 20.vi.1926, leg. C. O. Bare (1 ex. KSEM); Indian River Co., nr. Vero Beach, 12.iv.1983, leg. K. Hibbard (3 ex. FSCA); Lake Co., 26.iv., leg. E.M. Davis, (3 ex. FSCA); Leon Co., Springhill Rd., nr. Airport, 16.x.1980, leg. B. Lenczerski (1 ex. FSCA); Liberty Co., Yellow Creek SE of Telogia, 5.ix.1990, leg. F.N. Young, #3435 (1 ex. FSCA); same as previous except: 7.x.1992, #3503 (1 ex. FSCA); Torreya State Park, 16.v.1970, leg. H. Greenbaum, blacklight/sheet (1 ex. FSCA); Marion Co., 1-75 & Rte. 44, 12.iii.1988, leg. L.R. Davis Jr. & M. L. Benoit, at light (1 ex. FSCA); Village of Rainbow Springs, 3-7.vii.1982, leg. M.C. Thomas (2 ex. FSCA); Ocala, 5.viii.1975, leg. T. Rogers (1 ex. FSCA); Big Pine Key, 15.iii.1947, leg. L.D. Beamer (1 ex. KSEM); Okaloosa Co., 3 mi S. of Holt Log Lake Bridge, 4.x.1966, leg. P.A. Thomas (3 ex. FSCA); Palm Beach Co., 28.xi.1947, leg. McRae (1 ex. FSCA); Palm Beach Co., 3 mi N Bell Grande, 13.xii.1985, leg. R.S. Miller (2 ex. MTEC); Saint Lucie Co., White City,1.iv.1983, leg. K. Hibbard (2 ex. FSCA); U.S.A.: **Georgia:** Okefenokee Swamp, 30.vii.1934, leg. E. Griffith (2 ex. KSEM); same as previous except: 8.iii.1934, leg. P.A. McKinstry (1 ex. KSEM); Decantur Co., 1 mi W Recovery, 18.viii.1953, leg. F.N. Young, #986 (2 ex. FSCA); **Kansas:** Labette Co., Altamont, 5 mi E, Labette Creek, 22.vi.1974, SEMC 1054951 (1 ex. KSEM); **Louisiana:** St. John the Baptist, Edgard, 6.iii.1973, leg. V. Brou (2 ex. FSCA); same as previous except: 9.iii.1973 (1 ex. FSCA); same as previous except: 11.iii.1973 (2 ex. FSCA); same as previous except: 30.iii.1973 (1 ex. FSCA); same as previous except: 14.iv.1973 (2 ex. FSCA); 19.iv.1973 (1 ex. FSCA); same as previous except: 15.vi.1973 (2 ex. FSCA); same as previous except:13.vii.1973 (1 ex. FSCA); East Baton Rouge, Baton Rouge,19.x.1929, leg. H.A.S. (1 ex. MTEC); same as previous except: 31.vii.1961, leg. G.N. Ross (1 ex. FSCA); Madison, Tallulah, 7.vii.1930, leg. H. Mills (1 ex. MTEC); **Maryland:** Worcester Co., Pocomoke City, 22.ix.1984, leg. C.L. Staines Jr. (1 ex. FSCA); **North Carolina:** Carteret Co., Walker Mill Pond, 15.iii.1990, leg. J.B. Sullivan (1 ex. FSCA); Craven Co., North Harlowe, 18.vii.1990, leg. J.B. Sullivan (1 ex. FSCA); Jackson Co., Balsam, 2.v.1965, leg. W. Rosenberg (1 ex. FSCA); **Oklahoma:** Payne Co., nr Lake Carl Blackwell, 16.viii.1976 (1 ex. FSCA); **Texas:** Colorado Co., 3.iv.1922, leg. G. Wiley, “U of X Lot 1108” (3 ex. KSEM); Colorado Co., 18.v.1922, leg. G. Wiley (1 ex. KSEM); Montgomery Co., Woodlands, 2.vi.1979, leg. J.E. Wappes (3 ex. FSCA); same as previous except: 3.v.1980 (1 ex. FSCA); Walker Co., “Strawn”, 7.iii.1952, leg. T. Pyburn, “Green Branch” (1 ex. FSCA); **Virginia:** Middlesex Co., Warner, 13.x.1983, leg. C.L. Staines Jr. (1 ex. FSCA).

##### Diagnosis.

Male (Fig. 10C–D): Size: 9.1–10.9 mm. Body form elongate oval; elytral apices regularly rounded, with serrations and irregularities present apically, elytra with reticulation strong laterally and apically, medial disc with reticulation sparse or absent, striae faintly present, most evident medially on elytral disc, lateral marginal depression of elytra evident, narrow in humeral region, expanded posteriad, usually extending to lateral elytra apex; profemora with small sub-apicoventral tooth atop profemoral carina; protibiae subsinuate, distolateral margin flatly angled and weakly expanded; mesotarsal claws (Fig. 11F) with ventral margin weakly rounded; venter darkly colored, reddish brown to black, mesothoracic and metathoracic legs usually lighter in coloration, as well as apex of abdomen; Aedeagus (Fig. 11A) with median lobe in dorsal view nearly to as long as parameres, widest basally and regularly narrowed apically, more narrowed in apical 1/3, in some individuals much more noticeably narrowed in the apical 1/3, apex very shortly rounded, in lateral view median lobe sinuate ventrally in apical 1/3, parameres parallel-sided, broadly rounded apically.

Female (Fig. 10A–B): Size: 8.7–10.6 mm. Body form elongate oval; elytral apices regularly rounded, with serrations and irregularities present apically, apicolateral sinuation usually present, sometimes very strongly developed, elytra with reticulation strong laterally and apically, medial disc with reticulation sparse or absent, striae faintly present, most evident medially on elytral disc, lateral marginal depression of elytra evident, narrow in humeral region, expanded posteriad, usually extending to lateral elytra apex; profemora without sub-apicoventral tooth; protibiae weakly subsinuate, distolateral margin flatly angled; venter darkly colored, reddish brown to black, mesothoracic and metathoracic legs usually lighter in coloration, as well as apex of abdomen.

##### Differential diagnosis.

*Dineutus carolinus* is unique among North American *Dineutus* in having both sexes elongate oval and the elytra with a distinct lateral marginal depression, the elytral apices regularly rounded with serration and irregularities present, males with the profermoral sub-apicoventral tooth small and often atop a short carina, the male mesotarsal claws with the ventral margin rounded, and the unique shape of the aedeagus. The species most similar to *Dineutus carolinus* are *Dineutus emarginatus*, *Dineutus solitarius*, and *Dineutus americanus*.

Both sexes of *Dineutus carolinus* can be separated from *Dineutus emarginatus* by the elytra apices being more narrowly rounded with serrations and/or irregularities present. The presence of serrations, however, can be variable. In some individuals it is somewhat evident at the sutural margin, but others lack serrations entirely, having only roughened irregularities. The microreticulation of the elytra of *Dineutus carolinus* tends to be much more coarse laterally and the medial disk of the elytra often lacks reticulation, whereas *Dineutus emarginatus* tends to have fine microreticulation covering the entire elytra. Although not as reliable, the ventral coloration differs between the two species. In *Dineutus carolinus* the entire venter tends to be more reddish brown whereas it is regularly black in *Dineutus emarginatus*. The dorsal coloration of the two species is very similar.

Males of *Dineutus carolinus* can fairly easily be separated from *Dineutus emarginatus* by the profemoral sub-apicoventral tooth small atop a profemoral carina, rather than large and triangular. Also, in *Dineutus carolinus* the mesotarsal claws have the ventral margins rounded, rather than straight as in *Dineutus emarginatus*. The aedeagus is the best way to identify *Dineutus carolinus*. The median lobe of *Dineutus carolinus* is regularly narrowed for much its length, until the apical 1/3 where it is more strongly narrowed, but not strongly acuminate as in *Dineutus emarginatus*. Females of *Dineutus carolinus* are more difficult to separate from *Dineutus emarginatus*. The best way to distinguish them aside from the more narrowly rounded elytral apices with serrations and/or irregularities in *Dineutus carolinus* is the presence of an apicolateral sinuation in the elytra. This sinuation is nearly always present and well-developed in *Dineutus carolinus*, although in some females it is sometimes weakly developed or absent. *Dineutus emarginatus* females nearly always have this sinuation absent, but at most only weakly developed.

Members of *Dineutus carolinus* of both sexes can be distinguished from *Dineutus solitarius* by more elongate oval body form, the pronotum more narrow with the lateral margins more narrowly angled basally to apically, and the elytral apices more narrowly rounded apically with serrations and irregularities present. *Dineutus carolinus* of both sexes also have the lateral marginal depression of the elytra present, which is not evident in *Dineutus solitarius*. Males of *Dineutus carolinus* differ from those of *Dineutus solitarius* by the mesotarsal claws with the ventral margin curved, unlike *Dineutus solitarius* that have the ventral margins straight. The aedeagus of *Dineutus carolinus* is tapered but not acuminate, whereas that of *Dineutus solitarius* is acuminate. Females of *Dineutus carolinus* can also be separated from those of *Dineutus solitarius* by the apices of the elytra laterally sinuate, whereas in *Dineutus solitarius* they are usually evenly rounded without an apicolateral sinuation, and if a sinuation is present, it is very weakly developed.

*Dineutus carolinus* can be separated from *Dineutus americanus* by the differences provided under the differential diagnosis for *Dineutus americanus*.

##### Distribution

**(Fig. 53A).** Mainly known from the southeastern half of the United States (Epler 2010; Folkerts 1978; Régimbart 1907; Roberts 1895), as far south as the extreme northeast corner of Mexico (Wood 1962), and east into the Caribbean where it is primarily known from Nassau (Young 1953), the range is here extended to the southeast as far as Great Exuma Island.

##### Habitat.

This species appears to be primarily lentic (Young 1953, 1954). *Dineutus carolinus* occurs in Florida and is characteristic of small upland and flatwoods ponds, only rarely being found in slow streams with ample vegetation (Young 1954). The first author has collected *Dineutus carolinus* in slow moving mud bottomed streams and bayous in southeastern Texas.

##### Discussion.

*Dineutus carolinus* is the only species of *Dineutus* well established across much of continental North America as well as in the western Caribbean where its range overlaps with that of *Dineutus americanus*. The two species are fairly similar.

The Caribbean subspecies *Dineutus carolinus mutchleri* was described by Ochs (1924), from Nassau (Bahamas), who used several characters to separate it from the mainland subspecies including, size and number of setigerous femoral punctures, as well as aedeagal shape. Size shows overlap and is not discrete. Number of setigerous punctures is known to vary among populations, especially with size (pers. obs.; Wood 1962). Ochs (1924) described differences in the aedeagus comparing it to the illustration provided by Roberts (1895), but the aedeagus of specimens examined from the Bahamas are identical to that of the mainland populations. We found the aedeagus of the Bahaman specimens to be nearly identical to those from mainland Florida and elsewhere (Fig. 11). Young (1953) also noticed the similarity and suggested that the drawing by Roberts (1895) was actually from an undescribed form in Texas. Ochs (1929) also mentions having compared the Bahaman specimens of *Dineutus carolinus mutchleri* to specimens of *Dineutus carolinus* from Texas, and that those showed greater differences than the Bahaman form.

Having examined some specimens of *Dineutus carolinus* from Texas (FSCA) there is some variation, but not much from other populations of *Dineutus carolinus*. A single male specimen examined from Texas has minor variation in the aedeagus from other mainland specimens (Fig. 11H), which may explain Roberts (1895) illustration. This specimen has the apical 1/3 of the median lobe more strongly narrowed (Fig. 11H) than other populations of *Dineutus carolinus* (Fig. 11A–E, G) from the mainland. The median lobe of the Texas *Dineutus carolinus* is nearly as long as the parameres, which are very parallel-sided and flatly rounded, similar to other populations of *Dineutus carolinus* (Fig. 11). Although Roberts (1895) illustration shows the strong narrowing, it also indicates the median lobe shorter than the parameres, which is not the case in our specimen. The parameres in Roberts (1895) illustration, however, match well with our specimen. Therefore, it may be that Roberts (1895) drew the aedeagus with the median lobe out of proportion, or, more likely, he drew the median lobe slightly flexed dorsally as happens sometimes during eversion or relaxing of the aedeagus. Externally the male from Texas is very similar to males of other populations. The females of *Dineutus carolinus* from Texas populations vary more so than male specimens from Texas, when compared to populations outside of Texas. The females are much more broad in appearance, having the pronotal and elytral margins more broadly rounded laterally. In lateral view the females are also slightly more dorsoventrally convex than other *Dineutus carolinus* females. The increased convexity of the elytra causes the lateral marginal depression to be more shallowly impressed in comparison to other populations. The microreticulation of the elytra also shows variation being much more well-impressed, covering nearly all of the elytra and the pronotum. The elytral apices of Texas *Dineutus carolinus* females are more broadly rounded and do not have the normal lateral sinuation seen in other populations, but have the apices laterally angled or simply regularly meeting the rounded apices, and the apical serrations and irregularities are highly reduced although, under careful observation, present. All of these variations, however, are well within the typical range of variation within other species of *Dineutus* and it is our judgment that the populations from Texas are not differentiated enough to merit a formal taxonomic name. The specimens examined from Texas were from the southeastern part of the state, in Montgomery County, near The Woodlands, north of Houston (FSCA).

Ochs (1929) also admits that after having examined more *Dineutus carolinus* from Florida and Georgia, that *Dineutus carolinus mutchleri* are much more similar to these populations of *Dineutus carolinus* than the Texas forms which he used as a comparison during description of *Dineutus carolinus mutchleri*. It does appear that the Texas specimens are the most distinctive of *Dineutus carolinus* populations, and the populations from the Caribbean formally named as a subspecies by Ochs (1924) are not, in fact, particularly distinctive. Therefore, based on examined specimens from Texas, southeastern U.S., and the Caribbean, we consider *Dineutus carolinus mutchleri* as a junior subjective synonym of *Dineutus carolinus*.

#### Dineutus ciliatus

Taxon classificationAnimaliaColeopteraGyrinidae

(Forsberg, 1821)

**Figure 12.**
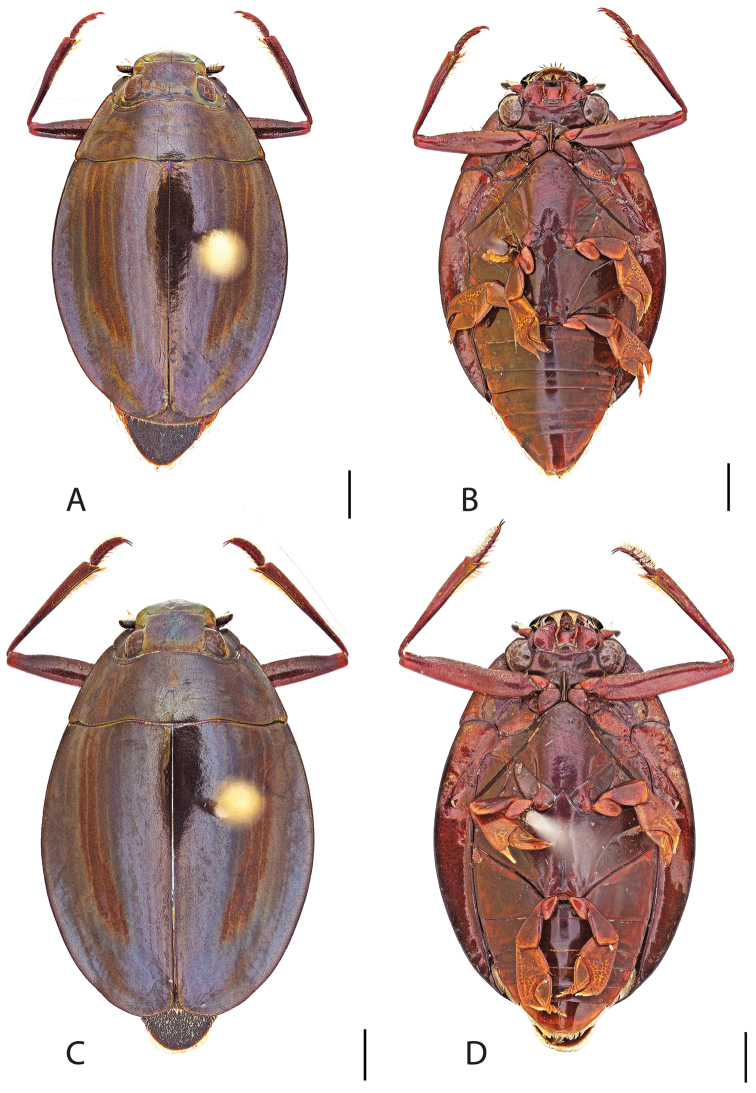
*Dineutus ciliatus*. **A** ♀ dorsal habitus **B** ♀ ventral habitus **C** ♂ dorsal habitus **D** ♂ ventral habitus. All scale bars ≈ 2 mm.

**Figure 13.**
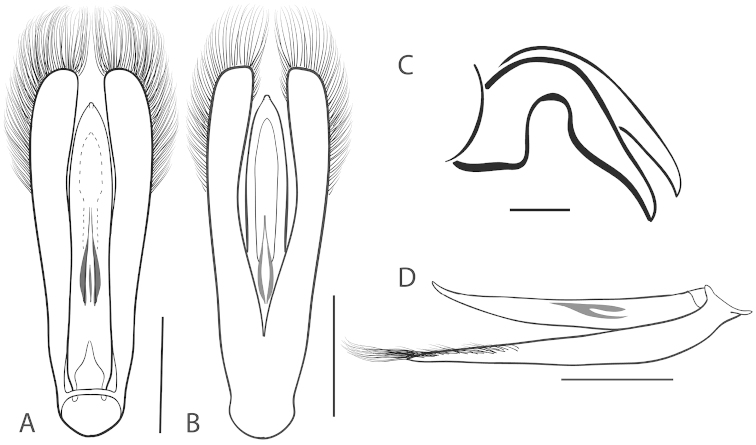
*Dineutus ciliatus*. **A** aedeagus dorsal view **B** aedeagus ventral view **C** ♂ mesotarsal claws **D** aedeagus lateral view. Scale bar for **C** ≈ 0.10 mm all others ≈ 1 mm.

Figures 12 , 13 , 53

Gyrinus ciliatus Forsberg 1821: 312, *Gyrinus vittatus*Germar 1824: 32 [synonymy by Ochs 1925b], *Cyclous vittatus*: Dejean 1833: 58, *Dineutes vittatus*: Brullé 1835: 240, *Cyclous opacus* Melsheimer, 1846: 29 [synonymy by LeConte 1868], *Dineutus vittatus*: LeConte 1868: 366, *Dineutes vittatus*: Régimbart 1882: 411, [*Dineutes hastatus*: Régimbart 1882: 426 misidentified], *Dineutes ciliatus*: Severin 1889: 152, *Dineutes vittatus*: Severin 1889: 155, *Dineutes opacus*: Severin 1889: 154, *Dineutes vittatus*: Régimbart 1892: 739, [*Dineutes hastatus*: Régimbart 1892: 740 misidentified], *Dineutes inflatus* Blackburn 1895: 28 [synonymy by Ochs 1926a], *Dineutus ciliatus*: Ochs 1925b: 174, Dineutus (Dineutus) vittatus: Hatch 1926: 311, Dineutus (Dineutus) ciliatus: Ochs 1926a: 138, *Dineutus vittatus*: Leonard 1928: 262, *Dineutus ciliatus*: Ochs 1930: 135, Dineutus (Dineutus) ciliatus: Hatch 1930: 19, *Dineutus vittatus*: Blackwelder 1944: 81. Dineutus (Dineutus) ciliatus: Ochs 1949: 286, Dineutus (Protodineutus) ciliatus: Guignot 1950: 126, Dineutus (Cyclinus) ciliatus: Brinck 1955: 106, *Dineutus ciliatus*: Ciegler et al. 2003: 15.

##### Type locality.

East Indies, likely in error. The type is labeled, “Ind.” according to Ochs (1949), which Young (1954) suggested could refer to Indiana, though Forsberg’s (1821) indicated “Indies oriental.”

##### Specimens examined.

73

##### Type material.

Not examined. Ochs (1925b) examined Forsberg’s types when establishing his synonymies, therefore the identity of *Dineutus ciliatus* is well established in relation to its most common synonym *Dineutes vittatus*.

##### Material examined.

**U.S.A.:** **Alabama:** U.S.A.: Alabama: Conecuh Co., 13 km E Evergreen on Hwy 31, Old Town Creek, 31°27.037'N, 86°49.81'W, 53 m, 11.v.2006, leg. K.B. Miller, KBM1105061 (6 ex. MSBA); Marion Co., Barnsville, 23.viii.1931, leg. R.H. Beamer (1 ex. KSEM); **Connecticut:** New London Co., New London, 16.v.1931, leg. M. Sanderson (1 ex. KSEM); **Delaware:** New Castle Co., Glasgow, 4.v.1957, leg. L.R. Krusberg (1 ex. FSCA); **Louisiana:** Beaugarl Co., 13.viii.1928, leg. R.H. Beamer Jr. (4 ex. KSEM); **Maryland:** Prince George’s Co., College Park, 4.x.1947, leg. B.K. Dozier (6 ex. FSCA); **Massachusetts:** Hampshire Co., Amherst, 16.vi.1904 (1 ex. MTEC); Norfolk Co., Blue Hills Reservation, v.1929, leg. G.C. Wheeler (1 ex. FSCA); **New Jersey:** Bergen Co., Dumont Woods, 9.iv.1931, leg. C.L. Ragot (1 ex. FSCA); Bergen Co., Woodcliff Lake, 20.v.1934 (3 ex. FSCA); Gloucester Co., 1 mi S Paulsboro, 3.vii.1959, leg. H.L. Dozier (4 ex. FSCA); Ocean Co., Cassville, branch of Tom’s River, vi.1931, leg. Siepmann (4 ex. FSCA); Lakehurst, 6.v.1934, leg. C.L. Ragot (1 ex. FSCA); **New York:** Westchester Co., White Plains, 2.x.1921, leg. E.H.P. Squire (1 ex. FSCA); same as previous except: 23.viii.1922 (2 ex. FSCA); same as previous except: 5.ix.1922 (2 ex. FSCA); 10.vi.1923 (19 ex. FSCA); **North Carolina:** Wake Co., Raleigh, leg. S.P. Whitney (1 ex. FSCA); **Oklahoma:** Larimer Co., 5 mi W Red Oak, 2.vii.1977, leg. K.H. Stephan (7 ex. FSCA); U.S.A.: Oklahoma: Larimer Co., 5 mi W Red Oak, vii.1980, leg. K.H. Stephan (1 ex. FSCA); Murray Co., Arbuckle Mts., nr Davis, 21.vi.1922 (1 ex. FSCA); **Rhode Island:** Kent Co., “Greenwich”, 15.vii.1934, leg. W. Sanderson (3 ex. KSEM); **South Carolina:** Sumter Co., 29.iv.1968, leg. L.L. Lampert, on stream (1 ex. FSCA). **No locality information:** “Station.”, 8.viii.1901, “Hatch Ex.” (1 ex. MTEC).

##### Diagnosis.

Male (Fig. 12C–D): Size: 12.7–14.6 mm. Body form broadly roundly oval; antennal flagellum thick and round, ultimate segment rounded; elytral apices regularly rounded, serration absent, elytra with bronzy lateral stripe disappearing apically, elytral striae weakly developed, 8^th^ elytral stria without punctures present or strongly evident; profemora without sub-apicoventral tooth; protibiae club-shaped; anterior mesotarsal claw (Fig. 13C) with denticle; venter normally dark reddish brown, rarely reddish orange (teneral individuals); Aedeagus (Fig. 13A, B, D) medial lobe in dorsal view with apicomedial papilla, in ventral view sperm-groove parallel sided, in lateral view median lobe curved, apically narrow, parameres weakly rounded laterally in apical 1/3.

Female (Fig. 12A–B): Size: 11.5–14.6 mm. Body form broadly roundly oval; Antennal flagellum thick and round, ultimate segment rounded; elytral apices regularly rounded, serration absent, elytra with bronzy lateral stripe disappearing apically, elytral striae weakly developed, 8^th^ elytral stria without punctures present or strongly evident; profemora without sub-apicoventral tooth; protibiae club-shaped; venter normally dark reddish brown, rarely reddish orange (teneral individuals).

##### Differential diagnosis.

This species is most easily distinguished from other members of North American *Dineutus* by the presence of a bronzy lateral stripe on each elytron, a regularly oval body form, large size, absence of a profemoral sub-apicoventral tooth in the male, and the form of the male aedeagus (Fig. 13A). *Dineutus ciliatus* is most similar to *Dineutus robertsi*, but there are several characters that readily separate the two species. Both sexes of *Dineutus ciliatus* have the venter dark reddish brown in color instead of light yellowish orange (most evident on the epipleura), the antennal flagellum thicker and rounder with the ultimate segment rounded instead of thinner and more parallel sided with an angled ultimate segment, and the 8^th^ elytral stria with punctures absent or indistinct. The venter coloration of teneral individuals is lighter, reddish orange, but this does not closely approach that of *Dineutus robertsi*, which is much more yellow in color. Males of *Dineutus ciliatus* also have the anterior mesotarsal claws with a more weakly developed denticle on their ventral surface, and a smaller general body size than *Dineutus robertsi*. The aedeagus (Fig. 13) of *Dineutus ciliatus* has the median lobe narrow and dorsally curved in lateral view with an apicomedial papilla visible in both dorsal and ventral views. The sperm-groove is much more parallel-sided in *Dineutus ciliatus* than *Dineutus robertsi*. Finally, the parameres of *Dineutus ciliatus* are weakly lateral curved in the apical 1/3 instead of strongly curved as in *Dineutus robertsi*.

##### Distribution

**(Fig. 53B).** Most of the eastern half of the United Sates (Folkerts 1978; Malcolm 1971; Sanderson 1982; Wood 1962; Young 1954).

##### Habitat.

This is a lotic species (Young 1954; Hatch 1927). In Florida *Dineutus ciliatus* is commonly found in small, shaded streams, and when found in larger streams prefers to stay near the stream bank (Young 1954). Hatch (1927) describes one habitat of *Dineutus ciliatus* in Massachusetts as a small sandy bottomed stream, approximately five feet wide and with a depth from six to ten inches, with a flow rate of one foot per second or less. Hatch (1927) also noted that *Dineutus ciliatus* prefers to reside near the bank of the stream. In east Texas this species was only collected in lotic situations at East Texas Primitive Big Thicket (Realzola et al. 2007). The first author has collected *Dineutus ciliatus* from both small pebble bottomed forested streams, to larger mud-bottomed rivers, throughout the southeastern United States, where it is quite commonly encountered.

##### Discussion.

*Dineutus ciliatus* is a common species with a wide range (Fig. 53B) and is frequently collected and represented in collections in large numbers.

#### Dineutus discolor

Taxon classificationAnimaliaColeopteraGyrinidae

Aubé, 1838

**Figure 14.**
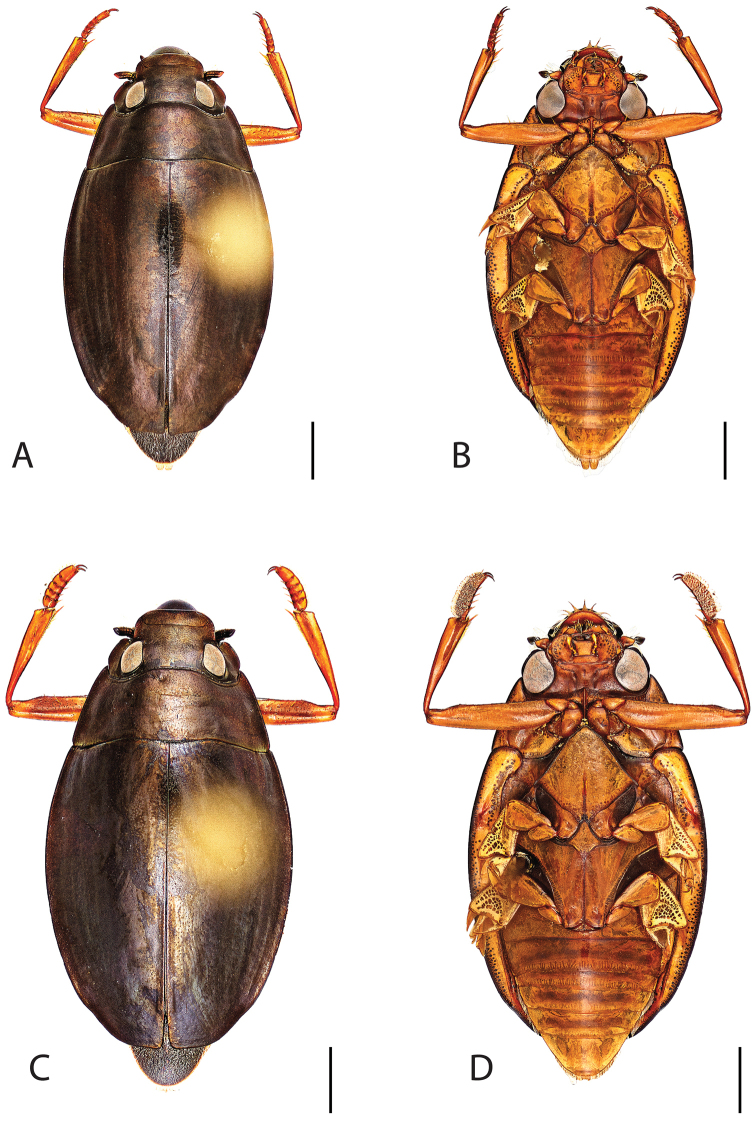
*Dineutus discolor*. **A** ♀ dorsal habitus **B** ♀ ventral habitus **C** ♂ dorsal habitus **D** ♂ ventral habitus. All scale bars ≈ 2 mm.

**Figure 15.**
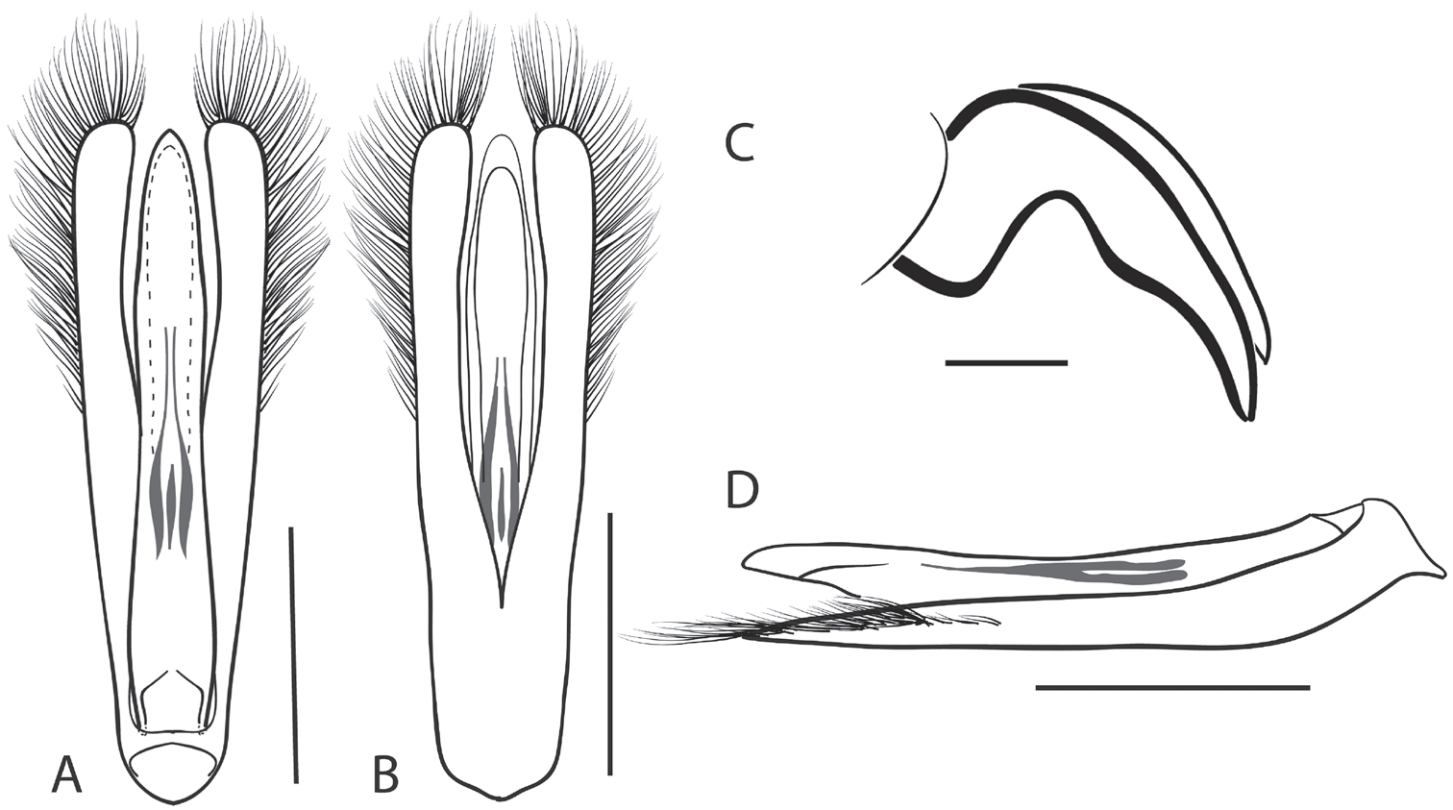
*Dineutus discolor*. **A** aedeagus dorsal view **B** aedeagus ventral view **C** ♂ mesotarsal claws **D** aedeagus lateral view. Scale bar for **C** ≈ 0.10 mm all others ≈ 1 mm.

Figures 14 , 15 , 51 , 52

Dineutes discolor Aubé 1838: 784, *Cyclous labratus*Melsheimer 1846: 9 [synonymy by LeConte 1868], *Dineutus discolor*: LeConte 1863: 18, *Dineutus labratus*: LeConte 1863: 18. *Dineutus discolor*LeConte 1868: 367, Dineutus (Cyclinus) discolor: Hatch 1925b: 448, Dineutus (Cyclous) discolor: Hatch 1927: 27, *Dineutes discolor*: Leonard 1928: 262, Dineutus (Cyclinus) discolor: Hatch 1930: 20, *Dineutus discolor*: Omer-Cooper 1934: 6, Dineutus (Cyclinus) discolor: Brinck 1955: 106, *Dineutus discolor*: Ferkinhoff and Gundersen 1983: 15.

##### Type localty.

The United States of America

##### Specimens examined.

93

##### Type material examined.

*Dineutus discolor* Aubé, 1838: lectotype, here designated (1 ♀ pinned, missing right protarsus and right mesothoracic leg) “MUSEUM PARIS/ AMÉRIQUE SEPT./ AUDOUIN 1833 [beige label, typed black ink]// green disc [underneath is written in ink is 4117/ 33]// TYPE [white label, typed red ink]// LECTOTYPUS/ P. Brinck designavit 1955. [white label, typed black ink]// LECTOTYPE/ Dineutus discolor/ Desig. RP Withington III/ 1998 [red label, handwritten in black ink]// LECTOTYPE [typed black ink]//” deposited in MNHN.

##### Material examined.

**U.S.A.:** **Alabama:** Marion Co., Barnsville, 23.viii.1931, leg. R.H. Beamer (1 ex. KSEM); Monroe Co., 10 km W Bowles, 31°33.094'N, 86°59.956'W, 11.v.2006, leg. K.B. Miller (1 ex. MSBA); **Arkansas:** Washington Co., Lake Sequoyah, 7.x.1992, leg. S. Garner (3 ex. MTEC); **Florida:** Holmes Co., Sandy Creek nr. Ponce de Leon, 11.vi.1978, leg. F.N. Young, #2756 (1 ex. FSCA); Santa Rose Co., Holly Creek at Rd. 260, 6.x.1966, leg. P.A. Thomas, (1 ex. FSCA); **Georgia:** Jackson Co., Allen Creek, S. Gainesville, 20.viii.1981, leg. F.N. Young, #2887 (1 ex. FSCA); **Indiana:** Putnam Co., Deer Creek, Manhattan, 19.viii.1969, leg. D.S. White (3 ex. FSCA); **Maine:** Oxford Co., Paris, 8.vii.1949, leg. C.R. Frost, 2674/ CAF’49 (1 ex. FSCA); York Co., Limington, Saco River, RT.11 at steep falls, 22.vi.1976 (1 ex. FSCA); **Maryland:** Patapsco River, 30.iv.1935, leg. W.L. Jellison (11 ex. MTEC); Prince George’s Co., Riverdale, 10.i.1910 (1 ex. MTEC) Montgomery Co., 2 mi. E. Silver Spring, N.W. Branch, 20.vii.1951, leg. G.H. Nelson (4 ex. FSCA); **Massachusetts:** Hampshire Co., Amherst, 24.vii.1967, leg. A Lavallee, (1 ex. FSCA); Norfolk Co., Dedham, 10.vi.1921, leg. G.C. Wheeler (1 ex. FSCA); **Missouri:** Reynolds Co., Sutton’s Bluff, 9.ix.1978, leg. K. Jackson, in creek (11 ex. FSCA); **New Jersey:** Raritan River Survey I, ACC Station 3B, 2.vii.1957, leg. T. Dolan (2 ex. KSEM); Middlesex Co., Avenel, 24.iv.1926, leg. Siepman (1 ex. KSEM); **New York:** New York, 8.v.1892, leg. E.O. Southwick, E.O. Southwick collection (1 ex. MTEC); Greene Co., East Durham, 26.vii.1971, leg. S.E. Thewke (1 ex. FSCA); Westchester Co., White Plains, 1.vi.1924, leg. E.H.P. Squire, (8 ex. FSCA); same as previous except: 8.vi.1924 (1 ex. FSCA); same as previous except: 10.vi.1923 (1 ex. FSCA); **North Carolina:** Macon Co., small pond in Watauga area n. Franklin, 26.vii.1986, leg. F.N. Young, #3118 (1 ex. FSCA); Moore Co., Mill Creek at Lake View, 7.ii.1966, leg. D.R. Paulson (1 ex. FSCA); Wake Co., 12.ix.1980, leg. R. Hollingsworth (3 ex. FSCA); Wake Co., Raleigh, leg. S.P. Whitney (4 ex. FSCA); same as previous except: 24.ix.1982, leg. R.H. Kenney (1 ex. FSCA); same as previous except: 12.ix.1984, leg. B.S. Bateman (1 ex. FSCA); same as previous except: 11.x.1984, leg. J.L. Williams (1 ex. FSCA); Wake Co., Raleigh, St. Road 1371, SW of Raleigh, 25.vii.1981, leg. S.P. Whitney, in stream (2 ex. FSCA); **Rhode Island:** Washington Co., Carolina, 24.ix.1970, leg. A. Lavallee (6 ex. FSCA); Kent Co., 1.ix.1969, leg. A. Lavallee (5 ex. FSCA); **South Carolina:** Aiken Co., Jackson, 4 mi NW at hwy 125 bridge, Holley Creek, 26.iii.1980, leg. D. Huggins, S. Hamilton, SEMC 1054961 (1 ex. KSEM); **Tennessee:** Cumberland Co., 8 mi S. Crossville, 26.vi.1962, leg. F.N. Young, #1968 (1 ex. FSCA); Maury Co., Colombia (1 ex. MTEC); **Virginia:** Albemarle Co., Charlottesville, 27.xi.1947, leg. H.H. Hobbs (1 ex. FSCA); **West Virginia:** Grant Co., 9 mi. SW Petersburg, 2.ix.1973, leg. J.B. Heppner (7 ex. FSCA).

##### Diagnosis.

Male (Fig. 14C–D): Size: 10.9–12.1 mm. Body form narrowly oval, attenuated anteriorly; elytral apices rounded with sutural angle produced into a point, rarely with point reduced and elytra appearing completely rounded, apicolateral sinuation present, serrations and/or irregularities absent, elytral striae very faint, most evident medially, elytra with fine microreticulation covering entirety, laterally microreticulation often coarser, medially with fine weakly impressed punctures; profemora with small weakly produced sub-apicoventral tooth; protibia weakly club-shaped, approaching subsinuate in large males, with distolateral margin weakly produced; mesotarsal claw as in Fig. 15C; venter lightly colored red to reddish orange; Aedeagus (Fig. 15A, B, D) median lobe in dorsal view parallel sided basally, weakly constricted medially, weakly narrowed in apical 1/3, apex obtusely rounded, in lateral view median lobe narrowed in apical 1/4, in ventral view sperm-groove parallel sided for near entirety of length, apex broadly rounded, parameres narrow, parallel sided, weakly arced basally to apically, apically very flatly rounded.

Female (Fig. 14A–B): Size: 10.6–12.8 mm. Body form narrowly oval, attenuated anteriorly; elytral apices rounded with sutural angle produced into a point, apicolateral sinuation present, serrations and/or irregularities absent, elytral striae very faint, most evident medially, elytra with fine microreticulation covering entirety, laterally microreticulation often coarser, medially with fine weakly impressed punctures; profemora without sub-apicoventral tooth; protibiae laterally weakly curved, distolateral margin weakly expanded; venter lightly colored, red to yellowish orange.

##### Differential diagnosis.

*Dineutus discolor* is unique among all North American species in the elongate oval and attenuate anteriorly body form, the elytra of both sexes with the apices rounded, with the sutural angle produced to a point with an apicolateral sinuation present, without serrations or irregularities present, a lightly colored venter, and males with the profemora with a small apicoventral tooth, and the form of the aedeagus (Fig. 15A). The species most similar to *Dineutus discolor* is *Dineutus angustus*. See the differential diagnosis under *Dineutus angustus* for separation of the two species.

##### Distribution

**(Fig. 52D).** Extreme southeastern Canada from Ontario to Nova Scotia (Majka and Kenner 2009; Roughley 1991; Webster and DeMerchant 2012), and the eastern half of the United States (Epler 2010; Ferkinhoff and Gunderson 1983; Folkerts 1978; Hilsenhoff 1990; Malcolm 1971; Régimbart 1907; Roberts 1895; Sanderson 1982; Wood 1962), south possibly into Mexico: Durango (Ochs 1949).

##### Habitat.

*Dineutus discolor* appears to be strictly lotic, inhabiting streams (Ferkinhoff and Gunderson 1983; Hilsenhoff 1990; Webster and DeMerchant 2012), and it has been suggested that this species prefers clear water (Morrissette 1979). In Quebec *Dineutus discolor* was found in clear running water and in New Brunswick within embayments and along the margins of rivers (Webster and DeMerchant 2012). In Florida, Young (1954) found *Dineutus discolor* to be a typical inhabitant of small streams in the uplands, avoiding the more acidic streams as well as the true flatwood streams within the state. Within streams *Dineutus discolor* swims in the current, moving upstream in quick jerks, allowing individuals to maintain their relative position in the stream among their aggregates, and swim downstream to dive and cling to submerged objects when alarmed (Hatch 1925a). In more swift streams *Dineutus discolor* is often found in eddies behind objects obstructing the current, such as fallen logs, and may be restricted to these habitats (Kolmes 1983a; Vulinec 1987). Stream dwelling *Dineutus* species also frequent areas where the current is slowed such as pools and wide meanders, avoiding torrential areas, riffles, and more swift currents (Folkerts and Donavan 1973). Members of *Dineutus discolor* form rafts in areas of flowing water within the streams, with individuals keeping pace so as to remain in position in the raft, and the raft maintains its relative position within the stream (Brown and Hatch 1929; Hatch 1925a). The first author has collected *Dineutus discolor* in both small pebble-bottomed forested streams as well as large mud-bottomed rivers in the southeastern United States. Both situations had clear water similar to situations described above and individuals collected from larger streams were found in areas with slowed water.

##### Discussion.

*Dineutus discolor* has an extensive range from the northern third of Florida up the Atlantic coast to Canada, and west to Iowa and Minnesota (Fig. 52D). The true extent of the western and southern boundaries of the range of *Dineutus discolor* seems somewhat uncertain. Ochs (1949) lists a single specimen of *Dineutus discolor* in his collection as having the label “Mexico, Durango”.

Kolmes (1983b; 1985) discussed the precopulatory behavior of *Dineutus discolor* describing the interesting “Proleg-up” mate signaling used by females. Kolmes (1983a) also investigated aspects of prey capture within this species.

The larvae of *Dineutus discolor* have been found under stones within streams with adult *Dineutus discolor* at a depth of 20 cm to 60 cm in areas where water is flowing, but not so rapidly as to create breaks in its surface such as rapids (Brown and Hatch 1929). Hatch (1927) included *Dineutus discolor* in a key to gyrinid larvae.

##### Type designation.

While several North American *Dineutus* specimens carry type designations by “RP Withington III”, these were never published thus do not consistute viable nomenclatural acts according to Article 11 of *The Code* (ICZN 1999). Among the material in the MNHN there are 4 other specimens designated by Withington III as paralectotypes, aside from the specimen here designated formally as the lectotype. Brinck also included a lectotype label on this specimen, but similarly, Brinck did not publish a written account of specimens he designated at the MNHN as lectotypes. The specimen selected here as the lectotype (Fig. 51B) has a locality label indicating the specimen is from the U.S. as well as a disc that allows the date and local to be checked in the MNHN’s registrar. Other specimens in the collection (including the unpublished paralectotypes designated by Withington) lack date indications. Therefore only the specimen with the date showing its collection prior to Aubé’s description, and being from the United States (as verified by the registrat at the MNHN) is safe to assume was a part of the original syntype series, and is here formally designated as the lectotype of *Dineutus discolor*. Given the uncertainty of the other specimens suggested to be paralectotypes by Withington III, it is our opinion that these specimens should not be regarded as paralectotypes.

#### Dineutus emarginatus

Taxon classificationAnimaliaColeopteraGyrinidae

(Say, 1825)

**Figure 16.**
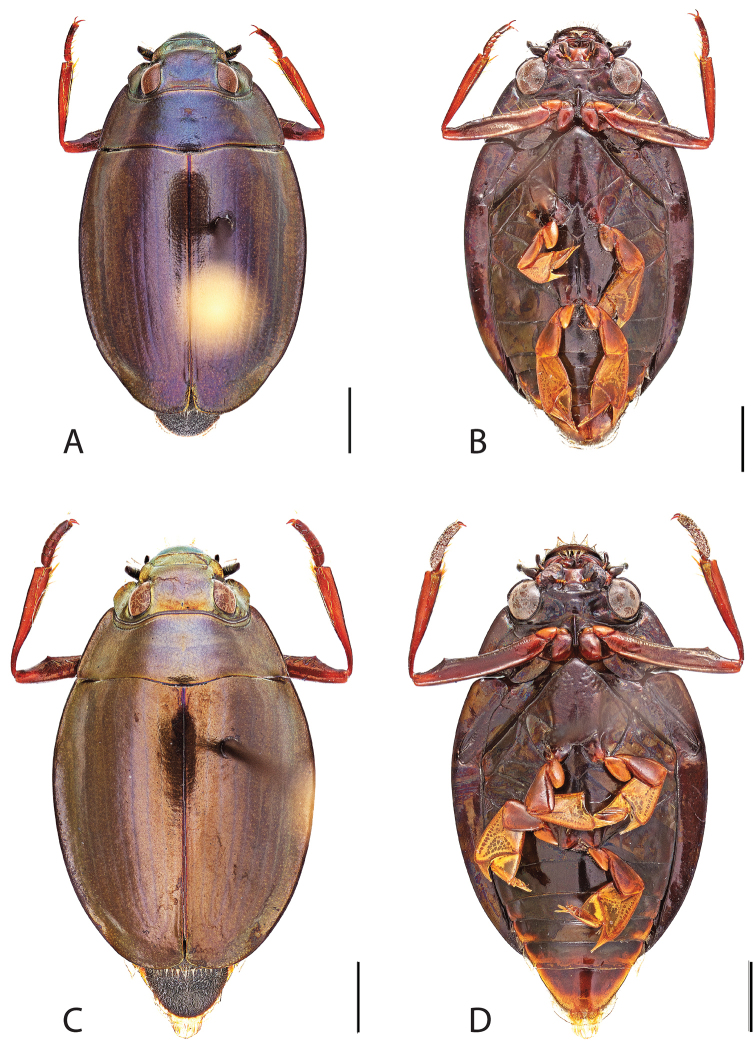
*Dineutus emarginatus*. **A** ♀ dorsal habitus **B** ♀ ventral habitus **C** ♂ dorsal habitus **D** ♂ ventral habitus. All scale bars ≈ 2 mm.

**Figure 17.**
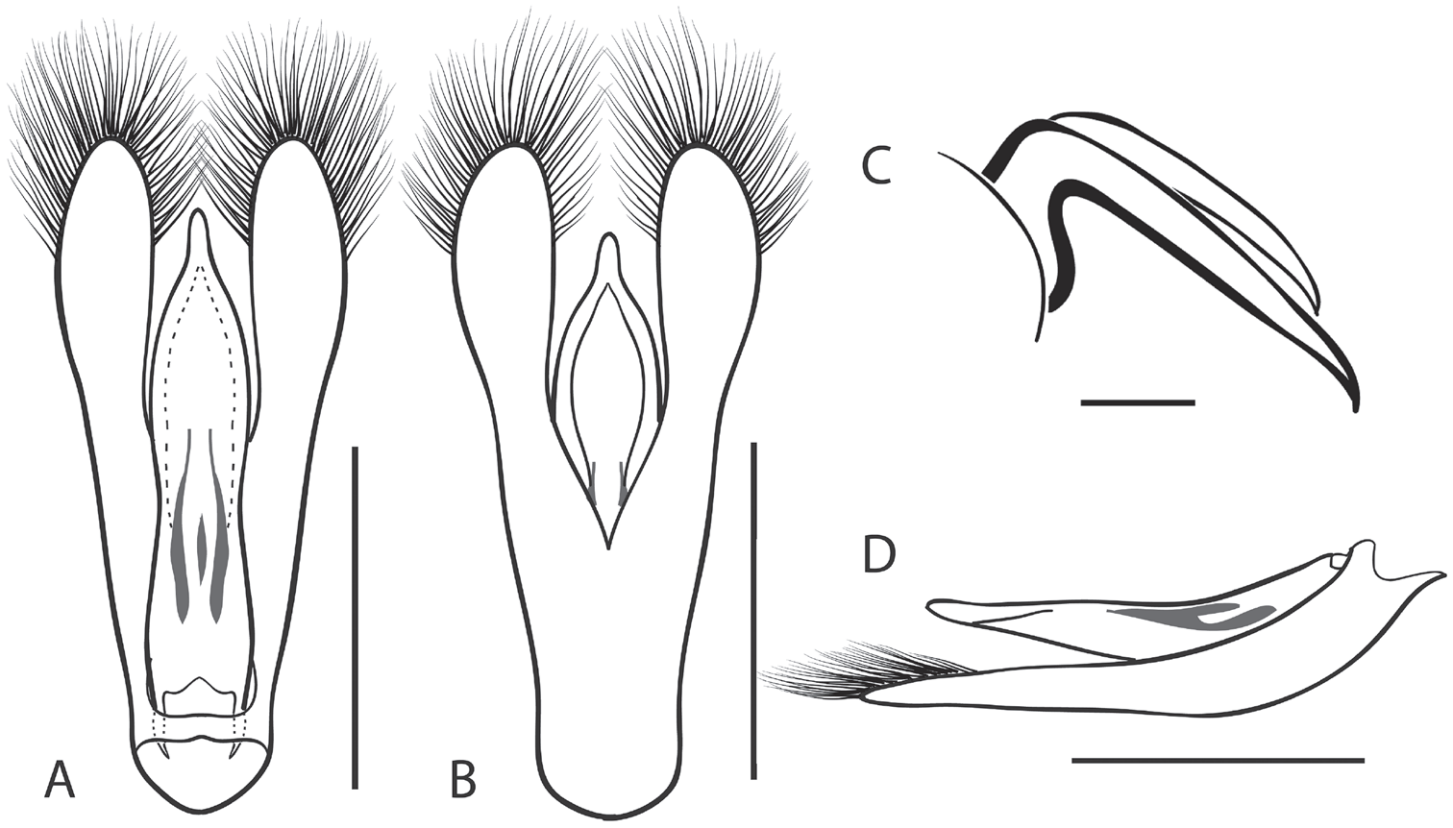
*Dineutus emarginatus*. **A** aedeagus dorsal view **B** aedeagus ventral view **C** ♂ mesotarsal claws **D** aedeagus lateral view. Scale bar for **C** ≈ 0.10 mm all others ≈ 1 mm.

Figures 16 , 17 , 53

Gyrinus emarginatus Say 1825: 108, [*Dineutes americanus*: Aubé 1838b: 777 misidentified], *Dineutus emarginatus*: LeConte 1863: 18, *Dineutes emarginatus*: Régimbart 1882: 366, *Dineutes emarginata* Kellog 1905: 257, Dineutus (Cyclinus) emarginatus: Ochs 1926a: 136, Dineutus (Cyclinus) emarginatus floridensis Ochs, 1929 [synonymy by Cook et al. 2006], *Dineutus emarginatus*: Ciegler et al. 2003: 15.

##### Type locality.

None given.

##### Specimens examined.

75

##### Type material examined.

None examined, none available. The Thomas Say entomology collection is known to have suffered substantial damage with portions of his type collection having been destroyed (Mawdsley 1993). Mawdsley (1993) provided a list of the surviving type specimens, but none of Say’s gyrinid species appear to remain. The MCZ online type database lists the following specimen as being a “Neotype”: (♀, pinned) “340/ ♀/ A. [brown label, handwritten in black ink]// NEOTYPE/ Dineutus emarginatus/ Desig. R.P. Withington III/ 1999 [red label, handwritten in black ink, handwriting unknown]// *Dineutus* ♀/ *emarginatus* (Say)/ Det. 1998 1999/ RP Withington III [white label, typed black ink, except cross through 1998, 1999, and female symbol, handwritten in black ink]// emarginatus 5 [white label, handwritten in black ink, unknown handwriting]// MCZ TYPE/ 34947 [red label, MCZ TYPE typed in black ink, 34947 handwritten in black ink]//.” As with all other R.P. Withington III designations for North America *Dineutus*, they were never published and are invalid, thus this specimen has absolutely no type status. As there is no question as to the identity of *Dineutus emarginatus* Say, no “exceptional need” exists for the designation of a neotype as per Article 75.3 of *The Code* (ICZN 1999), and we refrain from designating a neotype.

##### Material examined.

**U.S.A.:** **Alabama:** Monroe Co., 10km W Bowles, 31°33.094'N, 86°59.956'W, 11.v.2006, leg. K.B. Miller (9 ex. MSBA); **Connecticut:** New London Co., Ledyard, pond, 1.vi.1995, leg. K.B. Miller (2 ex. MSBA); **Florida:** Alachua Co., 2.ii.1949, leg. S.B. Mansell, (1 ex. FSCA); same as previous except: 23.iii.1949, leg. B.W. Cooper (1 ex. FSCA); same as previous except: 8.iv.1949, leg. E.H. McConkey (1 ex. FSCA); same as previous except: 30.iv.1949, leg. O.S. Russell (1 ex. FSCA); same as previous except: 15.iv.1950, leg. J.T. Darlington, (2 ex. FSCA); Alachua Co., Hatchet Creek, 25.vii.1975, leg. J.B. Heppner (4 ex. FSCA); Alachua Co., Gainesville, 6 mi. SW, 12.iii.1975, leg. L.R. Davis Jr., Blacklight trap (2 ex. FSCA); Clay Co., Camp Blanding Training Site, INSECT SURVEY SITE 11, Sand Pine Scrub, 29°55.599'N, 81°59.914'W, 24.ix.1999, leg. M. & M. Minno, Light trap (1 ex. FSCA); Hernando Co., Weekiwachee Spring, 5.iii.1953, leg. W.C. Sloan (1 ex. FSCA); Highlands Co., Archbold Biol. Sta., 7.iv.1975, leg. L.L. Lampert, UVL (2 ex. FSCA); Highlands Co., Highlands Hammock State. Prk., 11.iv.1964, leg. J. Waters (2 ex. FSCA); Collier Co., Immokalee, 23-28.iii.1960, leg. A.F. Wilson, Blacklight trap (1 ex. FSCA); Liberty Co., Yellow Creek SE of Telogia, 7.x.1992, leg. F.N. Young, #3503 (5 ex. FSCA); Walton Co., Eglin AFB., Range Rd. 205, 4.5 mi W. Hwy-331, 16.vi.1995, leg. P.E. Skelley et al., MV & blacklight (1 ex. FSCA); **Georgia:** Okefenokee Swamp, 1.iv.1969, leg. T.E. Rogers (1 ex. FSCA); Bibb Co., Macon, 20.v.1969 (1 ex. FSCA); Lowndes Co., 6.v.1963, leg. E.I. Hazard (1 ex. FSCA); **Louisiana:** West Feliciana, St. Francisville, 17.vi.1955 (1 ex. FSCA); **Maryland:** Charles Co., Allen’s Fresh, 15.viii.1984, leg. C.L. Staines Jr. (1 ex. FSCA); Prince George’s Co., Blue Pond, 29.ix.1949, leg. H.L. Dozier (1 ex. FSCA); Prince George’s Co., Greenbelt, 4.ix.1954, leg. H.L. Dozier, still pond in woods (1 ex. FSCA); **Mississippi:** Hancock Co., Turtleskin Creek, 25.iv.1965, leg. R. Hepburn (2 ex. FSCA); **New Jersey:** Bergen Co., Dumont Woods, 9.iv.1931, leg. C.L. Ragot, (5 ex. FSCA); **New York:** Queens Co., Corona, Long Island, 16.iv.1927, leg. C.L. Ragot (1 ex. FSCA); **North Carolina:** Wake Co., Raleigh, Yates Pond, 12.ix.1970, leg. L.L. Lampert (14 ex. FSCA); **Ohio:** Fairfield Co., Barnebey Center, 31.vii.1978, leg. D. Streett, sweeping net (4 ex. MTEC); **Oklahoma:** Latimer Co., 3.vii.1987, leg. K.E.M. Galley, at black light (2 ex. FSCA); **South Carolina:** Aiken Co., Jackson, 4 mi NW at hwy 125 bridge, Holley Creek, 26.iii.1980, leg. D. Huggins, S. Hamilton, SEMC 1054963 (1 ex. KSEM); **Texas:** Kinney Co., 17m NW Bracktville, 30.x.1997, leg. J.E. Wappes, MV/UV (1 ex. FSCA); La Salle Co., vic. Los Angeles, 11.ix.1993, leg. J.E. Wappes, (1 ex. FSCA); **No locality info:** “Station”, 8.v.1901, “Hatch Ex.”, (1 ex. MTEC).

##### Diagnosis.

Male (Fig. 16C–D): Size: 8.6–11.0 mm. Body form elongate oval; elytral apices regularly broadly rounded, with serrations and irregularities absent apically, elytra with fine reticulation covering its entirety, striae very faintly present, most evident medially on elytral disc, lateral marginal depression of elytra broad, usually extending to lateral elytra apex; profemora with large triangular sub-apicoventral tooth; protibiae subsinuate; mesotarsal claws (Fig. 17C) with ventral margin straight; venter darkly colored, usually black to very dark brown, mesothoracic and metathoracic legs usually lighter in coloration, as well as apex of abdomen; Aedeagus (Fig. 17A, B, D) median lobe in dorsal view shorter than parameres, medially slightly constricted, acuminate in apical 1/5 producing a strong point, apex very shortly rounded, in lateral view median lobe weakly curved dorsally in apical half, ventrally median lobe with large rounded sperm-groove, parameres in dorsal view rounded laterally at apical 1/3, narrowly rounded apically.

Female (Fig. 16A–B): Size: 8.9–10.1 mm. Body form elongate oval; elytral apices regularly broadly rounded, with serrations and irregularities absent apically, apicolateral sinuation usually absent, rarely developed, elytra with fine reticulation covering most of its entirety, striae very faintly present, most evident medially on elytral disc, lateral marginal depression of elytra broad; profemora without sub-apicoventral tooth; protibiae club-shaped; venter darkly colored, usually black to very dark brown, mesothoracic and metathoracic legs usually lighter in coloration, as well as apex of abdomen.

##### Differential diagnosis.

*Dineutus emarginatus* is unique among all other species of North American *Dineutus* in being elongate oval in body shape, having the elytral apices broadly rounded, without serrations and/or irregularities present apically, males with profemora possessing a large triangular sub-apicoventral tooth, and the shape of the aedeagus (Fig. 17A). The species most similar to *Dineutus emarginatus* are *Dineutus carolinus* and *Dineutus solitarius*. In general *Dineutus emarginatus* can be distinguished from *Dineutus solitarius* in being more elongate oval in body form, as opposed to being more regularly oval. This difference is especially evident in the pronotum. *Dineutus emarginatus* has the lateral margin of the pronotum more straightly angled posteriorly to anteriorly, while in *Dineutus solitarius* the pronotal lateral margin is more obtusely angled. The shape of the posterior margin of the pronotum differs between the two species, being more sinuate with the lateral portion of the posterior margin of the pronotum angled towards the posterolateral corners in *Dineutus emarginatus* as compared to the posterior margin being much more straight and less sinuate in *Dineutus solitarius*. *Dineutus emarginatus* in general also has a more evident lateral marginal depression of the elytra compared to *Dineutus solitarius*.

Males of *Dineutus emarginatus* have a much larger profemoral sub-apicoventral profemoral tooth than do those of *Dineutus solitarius*, but the species can be unambiguously separated based on the aedeagus. Both species have the median lobe acuminate, however, the acumination differs between the two species. In *Dineutus emarginatus* the apex of the median lobe is more rounded (Fig. 17A), with the acumination present as a strong point, whose apex is narrowly rounded. The apex of the median lobe of *Dineutus solitarius* (Fig. 43A) is narrowed towards the acumination, with the acumination also evenly narrowed and highly pointed, with the apex of the acumination appearing sharp and narrow. In overall appearance the median lobe of *Dineutus emarginatus* is more broad with a shallow medial constriction giving the basal and apical margins of the aedeagus a more sinuous feel, while in *Dineutus solitarius* the median lobe is more parallel sided and slightly narrowed apically, becoming slightly broadened just before the acumination.

The females of *Dineutus emarginatus* are primarily distinguished from females of *Dineutus solitarius* by the general differences listed for separating the two species.

*Dineutus emarginatus* can be separated from *Dineutus carolinus* by the differences listed in the differential diagnosis for *Dineutus carolinus*.

##### Distribution

**(Fig. 53D).** Most of the eastern half of the United States (Régimbart 1907; Young 1954; Wood 1962; Malcolm 1971; Folkerts 1978; Sanderson 1982).

##### Habitat.

*Dineutus emarginatus* can be found in both lotic and lentic habitats (Young 1954). In Florida *Dineutus emarginatus* is often found at the mouths of slow moving streams or in lakes in regions where wave action is present (Young 1954). This species can also be found in swamp streams, small sand bottomed streams, and canals with running water (Young 1954). The first author has collected this species form a large man-made lake in the Ozarks of Arkansas, as well as in a large slow moving tributary of a mud-bottomed stream (see the discussion under *Dineutus amazonicus*).

##### Discussion.

In the past *Dineutus emarginatus* was divided into two subspecies by Ochs (1929), *Dineutus emarginatus* *s. str.* and *Dineutus emarginatus floridensis* Ochs, 1929. Ochs (1929) based this subspecies on the smaller size of *Dineutus emarginatus floridensis* from that of the typical form and on the difference in the number of setigerous punctures of the profemora, as well as some minor differences in the body form and elytral shape. This subspecies was also stated to be restricted to peninsular Florida (Ochs 1929; Young 1954) with other populations outside of northern and central Florida being a part of the *Dineutus emarginatus* *s. str.* subspecies. Cook et al. (2006) performed a morphometric analysis of several populations of *Dineutus emarginatus* to assess the claims of a significant difference in size, as that being the main basis for the subspecies *Dineutus emarginatus floridensis*. Cook et al. (2006) were unable to discriminate between the two subspecies via a morphometric analysis and suggested the synonymy of *Dineutus emarginatus floridensis*. Ochs (1929) did also mention a difference in the setigerous punctures of the profemora, however as discussed elsewhere, this character is known to be variable.

In a study of gyrinid aggregations at East Texas Primitive Big Thicket by Realza et al. (2007), *Dineutus emarginatus* was most commonly collected with *Dineutus carolinus* and also with *Dineutus serrulatus analis*, but to a lesser extant, but the authors observed that at a given locality both species compositions and proportions were subject to change.

#### Dineutus hornii

Taxon classificationAnimaliaColeopteraGyrinidae

Roberts, 1895

**Figure 18.**
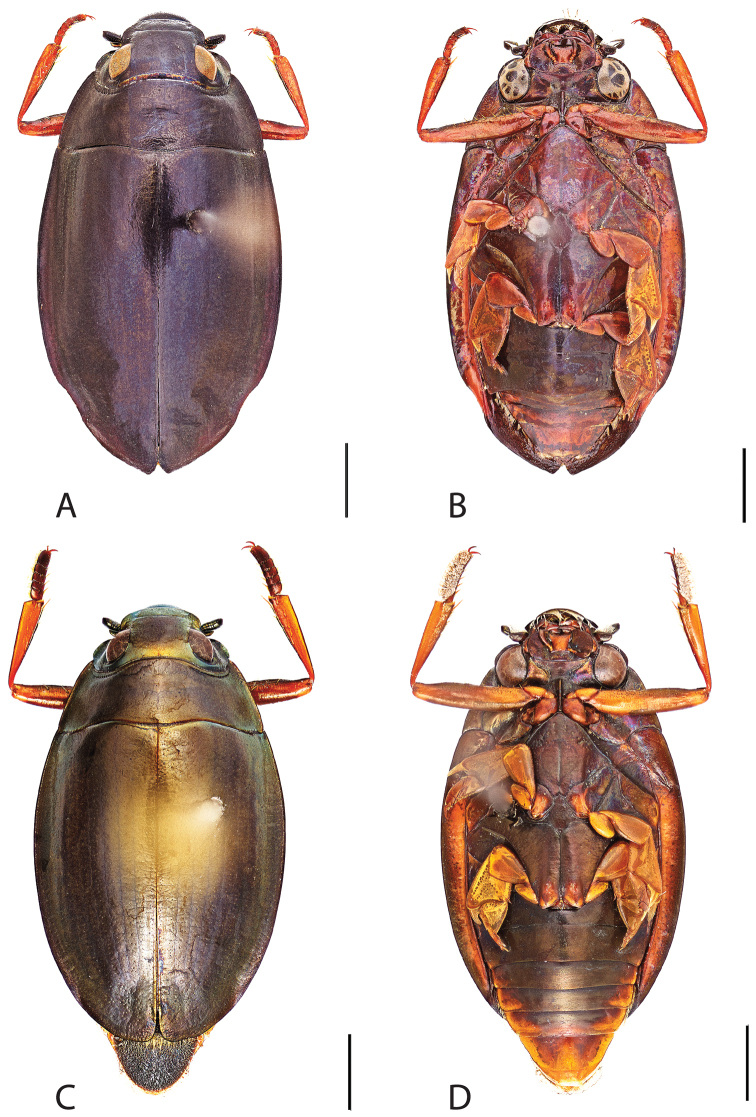
*Dineutus hornii*. **A** ♀ dorsal habitus **B** ♀ ventral habitus **C** ♂ dorsal habitus **D** ♂ ventral habitus. All scale bars ≈ 2 mm.

**Figure 19.**
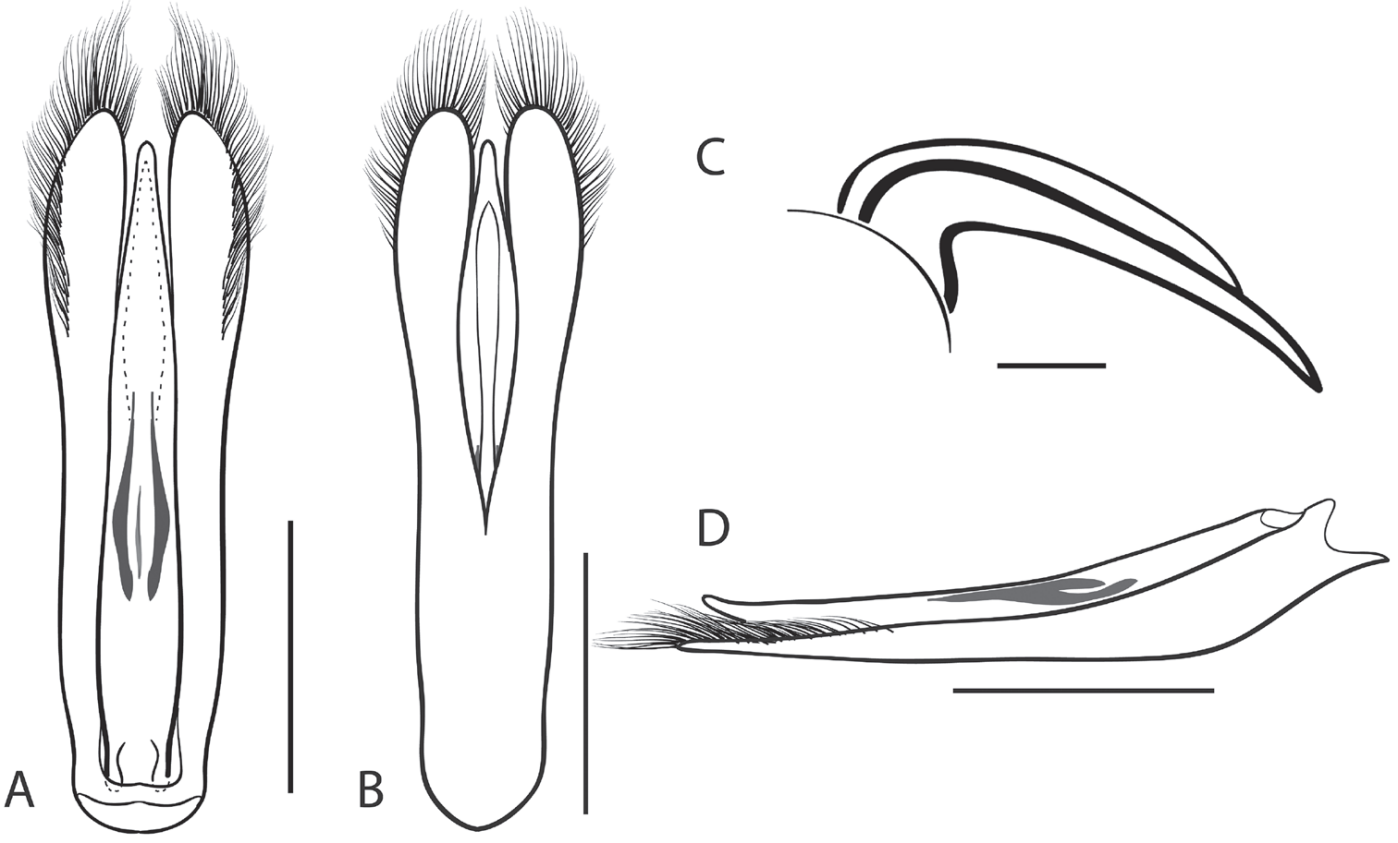
*Dineutus hornii*. **A** aedeagus dorsal view **B** aedeagus ventral view **C** ♂ mesotarsal claws **D** aedeagus lateral view. Scale bar for **C** ≈ 0.10 mm all others ≈ 1 mm.

Figures 18 , 19 , 52

Dineutes hornii Roberts 1895: 282, *Dineutes horni*: Régimbart 1907: 147, Dineutus (Cyclinus) horni: Ochs 1926a: 136, Dineutus (Cyclous) hornii: Hatch 1927: 28, *Dineutes horni*: Leonard 1928: 263, Dineutus (Cyclinus) hornii: Hatch 1930: 20, *Dineutus hornii*: Ferkinhoff and Gunderson 1983: 16.

##### Type localty.

New York.

##### Specimens examined.

45

##### Type material examined.

Syntype (♂ pinned, aedeagus extruded) “N.Y./Acc. 4858/Lectotype/ hornii ♂type # 4 C.H.R./LECTOTYPE Dineutus horni Desig: R.P. Withington III 1998/ Dineutus hornii Roberts 1895 Det. L. Cook 2005” AMNH catalog no. 497.

##### Material examined.

**U.S.A.:** **Iowa:** Boone Co., Ledges State Park, 2.v.1955, leg. M.D. Hoffman (2 ex. FSCA); **Indiana:** Brown Co., nr. Crooked Creek, 1.x.1977, leg. F.N. Young (1 ex. FSCA); Posey Co., Hovey Lake, 17.viii.1965, leg. C.E. White, Blacklight trap (1 ex. FSCA); **Massachusetts:** Middlesex Co., Hopkinton, 9.v.1915 (1 ex. FSCA); **Michigan:** Berrien Co., Harbert Dunes, 17.vii.1956, leg. G.H. Nelson, under washup (1 ex. FSCA); Cheboygan Co., 29.vii.1928, leg. F.G. Batcher, (1 ex. KSEM); same as previous except: 25.vii1931, leg. H.B. Hungerford (1 ex. KSEM); Cheboygan Co., Douglas Lake, 29.vii.1927, leg. H.B. Hungerford (1 ex. KSEM); Cheboygan Co., Douglas Lake, Bessey Cr., 30.vi.1925, leg. H.B. Hungerford (1 ex. KSEM); **New Hampshire:** Carroll Co., “Summer” 1934, leg. N.H. Preble (4 ex. KSEM); **New York:** Broome Co., nr. Binghamton, 10.vii.1997, leg. K.B. Miller (8 ex. MSBA); Schuyler Co., Texas Hollow State Wildlife Area, 1.ix.1999, leg. K.B. Miller (2 ex. MSBA); Tompkins Co., Ringwood, 42°26'33"N, 76°21'47"W, 20.v.2000, leg. K.B. Miller (7 ex. MSBA); Saint Lawrence Co., Black Lake, 27.vii.1941, leg. E.J. Gerberg (1 ex. FSCA); Westchester Co., White Plains, 14.v.1922, leg. E.H.P. Squire (1 ex. FSCA); same as previous except: 30.v.1923 (1 ex. FSCA);

Putnam Co., Carmel, 2.viii.1923, leg. E.H.P. Squire (2 ex. FSCA); **Pennsylvania:** Sussex Co., Peck’s Pond, 41°16'55.4"N, 75°15'18"W, 414 m, 29.v.2007, leg. P.A. Lenhart, swimming in pond (2 ex. MSBA); **South Carolina:** Aiken Co., Jackson, 4 mi NW at hwy 125 bridge, Holley Creek, 25.iii.1980, leg. D. Huggins, S. Hamilton, SEMC 1054964 (1 ex. KSEM); **Wisconsin:** Richland Co., lower WI River, State Wildlife Area, 2 mi W of Lone Rock, 4.x.1997, leg. A. Ramsdale, on surface of lentic pond, near margin, day (6 ex. MTEC).

##### Diagnosis.

Male (Fig. 18C–D): Size: 9.9–10.9 mm. Body form narrowly oval; antennal flagellum short and thick, ultimate segment broad and round; elytral apices rounded, rarely angled toward suture, elytral striae faintly; profemora without sub-apicoventral tooth; protibiae wedge-shaped, with distolateral margin straight; mesotarsal claws (Fig. 19C) similar in size, venter darkly colored reddish brown to black, except epipleura lighter in color, yellow to orange; Aedeagus (Fig. 19A, B, D) median lobe in dorsal view mostly parallel sided, evenly narrowed in apical 1/3, apex strongly narrowed, flatly rounded, in lateral view apex of median lobe weakly curved dorsally; parameres in dorsal view laterally expanded in apical 1/4, nearly evenly rounded apically, in ventral view sperm grove narrow and parallel sided for most its length.

Female (Fig. 18A–B): Size: 10.3–11.3 mm. Body form narrowly oval; antennal flagellum short and thick, ultimate segment broad and round; elytral apices produced, angled towards sutural production, sutural angle produced into a point, apicolateral sinuation present, elytral striae faint basally and laterally, mainly evident apicomedially, becoming more evident apically and laterally; profemora without sub-apicoventral tooth; protibiae laterally weakly curved, distolateral margin weakly expanded; venter darkly colored, reddish brown to black, except epipleura lighter in color, yellow to orange.

##### Differential diagnosis.

*Dineutus hornii* can be distinguished from all other North American species of *Dineutus* in having the epipleura light yellow to orange, while still having a darkly colored venter (as opposed to similarly lightly colored as in *Dineutus discolor*) (Fig. 18B, D), and the males by the form of the aedeagus (Fig. 19A). The species most similar to *Dineutus hornii* are *Dineutus assimilis* and *Dineutus nigrior* especially the female forms. But both sexes differ from the following two species in having the epipleura (Fig. 19B, D) lightly colored yellow to orange relative to their darker venters, as well as having antennal flagella that are short and thick with the ultimate segment appearing broad and round. In both *Dineutus assimilis* and *Dineutus nigrior* the epipleura is similarly darkly colored like the rest of the venter, and in *Dineutus assimilis* often accompanied with a metallic sheen. The ultimate segment of the antennal flagellum in both species differs in being angled as opposed to rounded as in *Dineutus hornii*.

Males of *Dineutus hornii* can be readily distinguished from both *Dineutus assimilis* and *Dineutus nigrior* in having the elytral apices rounded (rarely angled towards the suture) and without the sutural angle produced to a point (Fig. 18C). The aedeagus (Fig. 19A) of *Dineutus hornii* is more similar to *Dineutus nigrior* (Fig. 32A), but can distinguished by the median lobe in dorsal view being more parallel sided, being evenly narrowed in the apical 1/3 (as opposed to apical 1/4), and having the apex of the median lobe flatly rounded at the tip. The median lobe also differs from *Dineutus nigrior* in having the apex weakly curved dorsally in lateral view (Fig. 19D) as opposed to strongly curved in *Dineutus nigrior* (Fig. 32D). The pararmeres of *Dineutus hornii* differ from those of *Dineutus nigrior* in being expanded laterally in the apical 1/4, and evenly rounded apically, and in lateral view being obtusely angled after the basal 1/3 as opposed to strongly angled.

Females of *Dineutus hornii* can be somewhat fairly easily distinguished from those of *Dineutus assimilis* and *Dineutus nigrior* in that the apices of the elytra are angled towards the suture (Fig. 18A) as opposed to being rounded towards the produced sutural angle. This character combined with the epipleural color and the antennal flagellum shape should readily distinguish females of *Dineutus hornii* from both *Dineutus assimilis* and *Dineutus nigrior*.

##### Distribution

**(Fig. 52B).** Extreme southeastern Canada from Saskatchewan to Nova Scotia (Majka 2008; Majka and Kenner 2009; Roughley 1991) and the eastern half of the United States as far south as Texas and northern Florida (Epler 2010; Ferkinhoff and Gunderson 1983; Folkerts 1978; Hilsenhoff 1990; Malcolm 1971; Roberts 1895; Sanderson 1982; Whiteman and Sites 2003; Wood 1962).

##### Habitat.

This species is primarily lentic and occasionally found in streams (Hilsenhoff 1990; Whiteman and Sites 2003). In Canada *Dineutus hornii* inhabits boggy and semi-boggy lakes (Morrissette 1979). In the Missouri Prairie Region, Whiteman and Sites (2003) record this species in ponds with *Brasneia*.

##### Discussion.

The spelling of the specific epithet *hornii* is in some places spelled *horni*, but the discrepancy in spelling was clarified by Majka (2008), finding “*horni*” to be an incorrect subsequent spelling of *hornii*. The unambiguously correct spelling is *Dineutus hornii* (Majka, 2008).

*Dineutus hornii* forms rafts during the daytime consisting of hundreds to thousands of individuals, which may be composed of multiple species (Heinrich and Vogt 1980). At night some individuals disperse to forage, returning to larger rafts of beetles just before dawn, while others do not leave the larger rafts formed during the day (Heinrich and Vogt 1980), unlike the behavior observed by Fitzgerald (1987) for *Dineutus nigrior* where the diurnal rafts of this species appeared to totally disperse by night. Heinrich and Vogt (1980) suggest that *Dineutus hornii* is nocturnal as foraging behavior occurs at night, with the diurnal period spent quiescently in rafts. Brief life history is available in Istock (1966; 1967).

The larva of *Dineutus hornii* was included in a key to gyrinid larvae by Hatch (1927) and the structure of the egg has been describe by Baker and Wai (1987).

#### Dineutus longimanus

Taxon classificationAnimaliaColeopteraGyrinidae

(Olivier, 1791)

##### Differential diagnosis.

This is a very unique *Dineutus* species, easily distinguished from all other New World *Dineutus*, by the elytral apices possessing a spine located between the sutural and apicolateral angles. Serrations and irregularities are also present, and the sutural angle is also produced into a short spine. The venter is usually more lightly colored from reddish to yellow.

##### Distribution and subspecies.

This species is endemic to the Caribbean and represents a bit of a population genetics and Caribbean biogeography problem. The species is currently divided into four subspecies, with each island of the Greater Antilles having its own subpsieces, with the two most unique subspecies being found at opposite ends of the species distribution (Cuba and Puerto Rico). The two subspecies occupying the middle area of the range (Jamaica and the Dominican Republic) are less distinct and seem to form a gradient between the morphologies of the two subspecies at the opposite ends. The aedeagi of three of the subspecies are very similar with only that of *Dineutus longimanus portoricensis* offering some significant differences. For these reasons we have decided to retain the classification proposed by Ochs (1924, 1926a, 1938) in treating the four forms as subspecies. Future genetic work may shed some light on these issues.

*Dineutus longimanus* can be distinguished from all other North American species of *Dineutus* in having the elytral apices spinose with serrations and irregularities present. The subspecies can be separated by the following key:

**Table:** 

1	Body form of both sexes evenly elongate oval (Figs 20A, C; 22A, C); Elytra with apicolateral sinuation not evident, at most weakly developed, apical serrations strongly developed as small thorns; Mesotarsal claws with ventral margin rounded and evenly narrowed apically, without a denticle (Figs 21C; 23C); Metacoxae with numerous shallow punctures extending on to lateral wings of metacoxae; Median lobe of aedeagus more parallel sided, parameres of aedeagus more narrowly rounded (Fig. 21A; 23A). Western Caribbean	**2**
–	Body form of males more laterally expanded after basal half of elytra (Figs 24C; 26C); Elytra with apicolateral sinuation moderately to strongly evident, apical serrations more weakly developed especially apicolaterally; Mesotarsal claws with a denticle (Figs 25C; 27C); Metacoxae with sparse and very shallowly impressed punctures; Median lobe of aedeagus weakly constricted medially, parameres of aedeagus more broadly rounded (Figs 25A; 26A). Eastern Caribbean	**3**
2	Relatively smaller in size: 10.8–12.9 mm. Reticulation of dorsal surface more well impressed, medial disc reticulation composed of small regularly shaped circular sculpticells; Metacoxae with numerous large, shallow punctures present, extending regularly on to the metacoxal wings. Cuba	***Dineutus longimanus cubensis***
–	Relatively larger: 12.1–13.3 mm. Elytral reticulation much more finely impressed, reticulation of medially disc very weakly impressed composed of irregularly shaped sculpticells which are more transversely oriented; Metacoxae with punctures more shallowly impressed, decreased in number the metacoxal wings, only present on the posterior half. Jamaica	***Dineutus longimanus jamaicensis***
3	Dorsally margin of elytra with lateral greenish sheen; metacoxae with punctures more sparse and more shallowly impressed, barely distinguishable; median lobe of aedeagus with narrow dorsal carina at apex, apically less acuminate and more evenly angled apically. Puerto Rico	***Dineutus longimanus portoricensis***
–	Margin of elytral without lateral greenish sheen; metacoxae with punctures relatively apparent, but still sparse and shallowly impressed; median lobe of aedeagus without dorsal carina, more acuminate apically. Dominican Republic, Haiti	***Dineutus longimanus longimanus***

#### Dineutus longimanus cubensis

Taxon classificationAnimaliaColeopteraGyrinidae

Ochs, 1927

**Figure 20.**
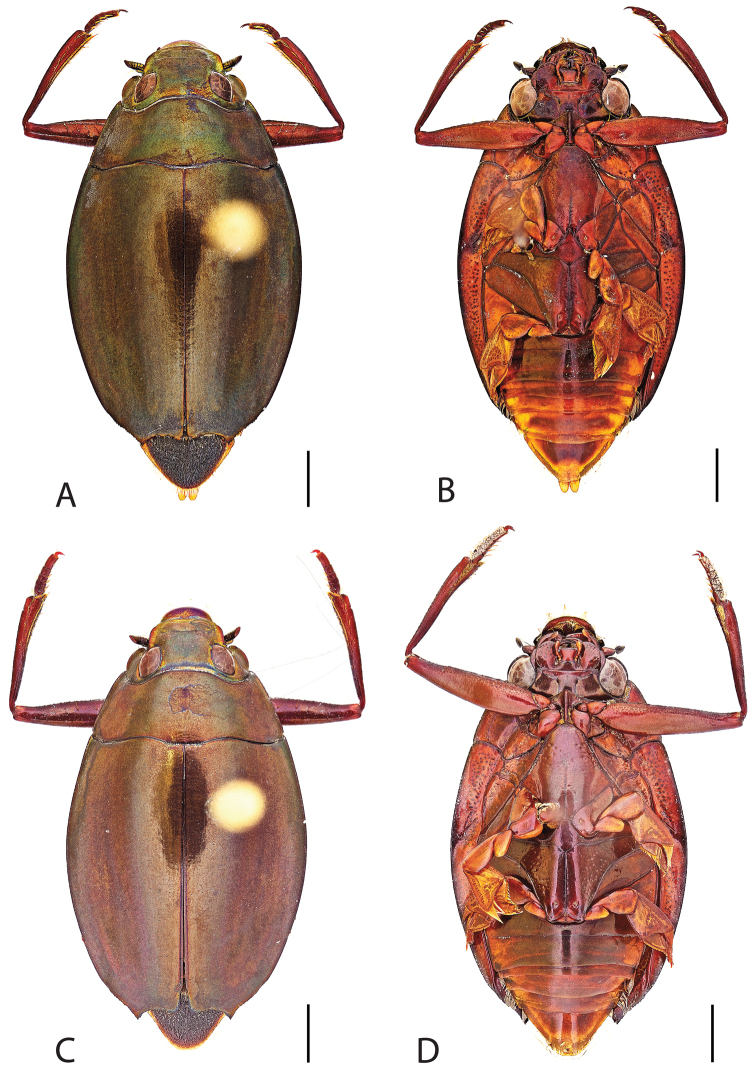
*Dineutus longimanus cubensis*. **A** ♀ dorsal habitus **B** ♀ ventral habitus **C** ♂ dorsal habitus **D** ♂ ventral habitus. All scale bars ≈ 2 mm.

**Figure 21.**
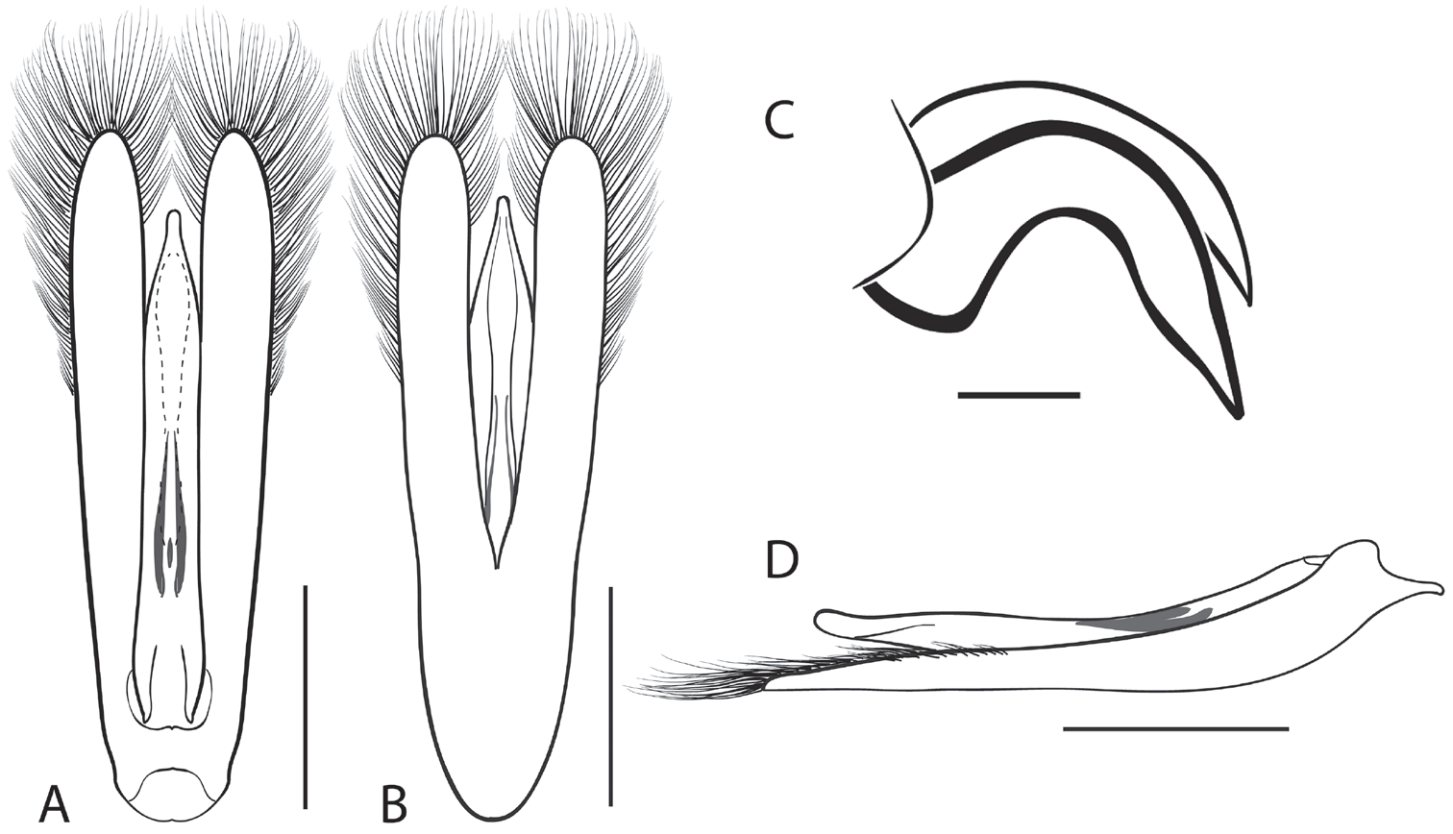
*Dineutus longimanus cubensis*. **A** aedeagus dorsal view **B** aedeagus ventral view **C** ♂ mesotarsal claws **D** aedeagus lateral view. Scale bar for **C** ≈ 0.10 mm all others ≈ 1 mm.

Figures 20 , 21 , 55

Dineutus (Dineutus) longimanus cubensis Ochs 1927a: 192, Dineutus (Rhombodineutus) longimanus: Guignot 1950: 127, Dineutus (Cyclinus) longimanus: Brinck 1955: 106, *Dineutus longimanus cubensis*: Peck et al. 1998: 158.

##### Type locality.

Cuba, Santiago de Cuba.

##### Specimens examined.

17

##### Type material examined.

None examined.

##### Material examined.

**CUBA:** **Holguín:** Sierra de Nipe, 25km S Mayari, Pinares de Mayari, 650 m, 03.vii.1990, leg. M.A. Ivie (7 ex. WIBF); Sierra de Nipe, Rio Piloto, 4.ii.1967, leg. R. Bielawski & A. Riedel (1 ex. WIBF) same as previous except: 590 m, 07.vii.1990, leg. M.A. Ivie (7 ex. WIBF); **Pinar del Rio:** Sierra del Rosario, Rancho Mundito, 16.vi.1959, leg. M.W. Sanderson, C59-29 (1 ex. FSCA); Sierra del Rosario, ca. 15km S CincoPesos Rangel, 420 m, 29.vi.1990, leg. M.A. Ivie, (1 ex. WIBF).

##### Diagnosis.

Male (Fig. 20C–D). Size: 11.4–12.9 mm. Body form regularly elongate oval; elytral apices spinose, with sutural angle produce to a spine, and a second parasutural spine, with thorn-like serrations and irregularities present apically and apicolaterally, apicolateral sinuation mostly absent, elytra with reticulation strong laterally and apically, producing a bronzy appearance, medial disc with reticulation more weakly impressed and composed of smaller cells accompanied by very shallowly impressed punctation, striae mostly effaced by reticulation, if evident at all faintly apparent medially on disc, lateral marginal depression of elytra absent; profemora with very small sub-apicoventral tooth; protibiae club-shaped; mesotarsal claws (Fig. 21C) with ventral margin regularly rounded and evenly narrowed apically; metacoxae with numerous shallow punctures present over most their ventral face; venter lighter in color: reddish brown to reddish orange. Aedeagus (Fig. 21A, B, D) with median lobe in dorsal view shorter than parameres, nearly parallel sided, slightly wider basally and shallowly narrowed apicad, in apical 1/4 shallowly narrowed towards apex, apex regularly rounded, dorsally without narrow carina, ventrally sperm-groove narrow and parallel sided for most its length, apically briefly widened, in lateral view median lobe with dorsal margin shallowly sinuate in apical 1/3, apex broadly rounded; parameres in dorsal view with lateral margins not laterally expanded, parallel sided for most their length, and apically narrowly rounded.

Female (Fig. 20A–B). Size: 10.8–12.4 mm. Body form regularly elongate oval; elytral apices spinose, with sutural angle produce to a spine, and a second parasutural spine, with thorn-like serrations and irregularities present apically and apicolaterally, apicolateral sinuation mostly absent, elytra with reticulation strong laterally and apically, producing a bronzy appearance, medial disc with reticulation more weakly impressed and composed of smaller cells accompanied by very shallowly impressed punctation, striae mostly effaced by reticulation, if evident at all located medially on disc, lateral marginal depression of elytra absent; profemora without sub-apicoventral tooth; protibiae club-shaped; metacoxae with numerous shallow punctures present over most their ventral face; venter lighter in color: reddish brown to reddish orange.

##### Differential diagnosis.

*Dineutus longimanus cubensis* is unique among the other subspecies of *Dineutus longimanus* in being smaller in size (10.8–12.9 mm) and having the metacoxae with numerous shallow punctures present and covering most of their surface. The subspecies most similar to *Dineutus longimanus cubensis* is *Dineutus longimanus jamaicensis* and can primarily be distinguished by the differences in dorsal punctation and the punctures of the metacoxae as provided by the indentification key.

##### Distribution

**(Fig. 55B).** Cuba (Leng and Mutchler 1914a; Ochs 1924, 1926)

##### Habitat.

Lotic, according to Peck et al. (1998) this subspecies occurs in streams through out Cuba and is an accidental cave inhabitant. Cave records for this species include Cueva Jíbara 8, Santiago de Cuba Province and Cueva Caja de Aqua, Saneti Spiritus Province (Peck et al. 1998).

##### Discussion.

Information on the subspecies aside from its taxonomy has been scarce. Given what is currently known it appears that *Dineutus longimanus cubensis* is only known from Cuba.

It is worth noting that the date of the description of *Dineutus longimanus cubensis* is often given as 1926 (Ochs 1929, 1938) as the name was used earlier by Ochs (1926-1927). However, according to Article 12.1 of *The Code* (ICZN 1999) in order for the nomen to be available it must be accompanied by a description or definition and in 1926 the name was simply used with an asterix making it a *nomen nudum* at that time. The description of *Dineutus longimanus cubensis* was not included until the final part of the work published in 1927 on page 192 (Ochs 1927a). Thus the true date for *Dineutus longimanus cubensis* must be 1927 as that is when the nomen satisfied the criteria of *The Code* for availability.

#### Dineutus longimanus jamaicensis

Taxon classificationAnimaliaColeopteraGyrinidae

Ochs, 1938

**Figure 22.**
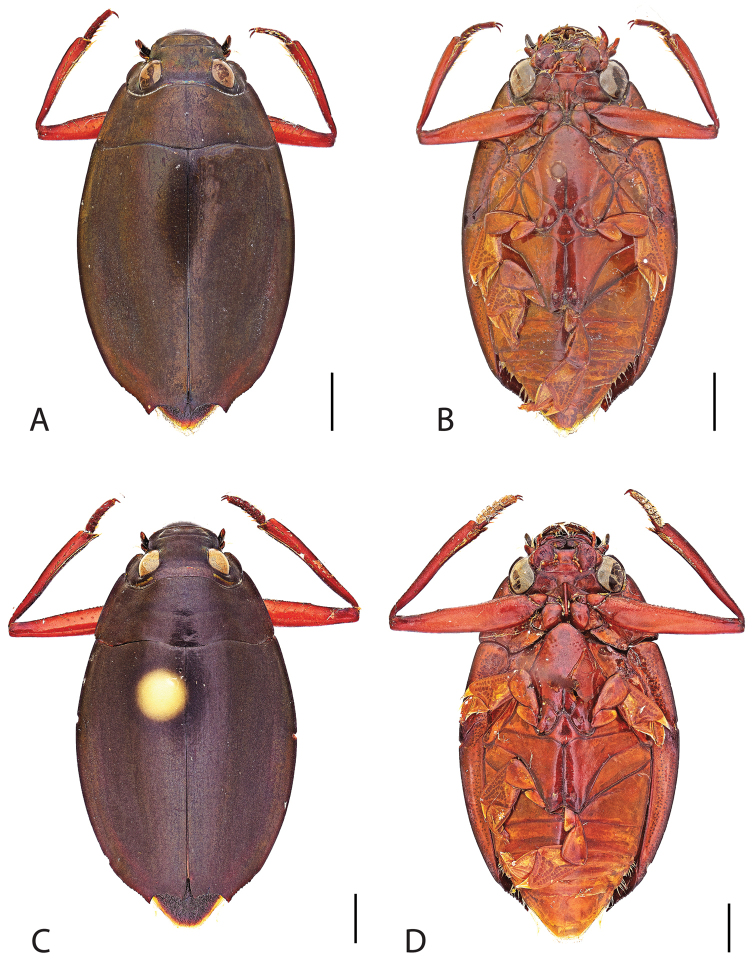
*Dineutus longimanus jamaicensis*. **A** ♀ dorsal habitus **B** ♀ ventral habitus **C** ♂ dorsal habitus **D** ♂ ventral habitus. All scale bars ≈ 2 mm.

**Figure 23.**
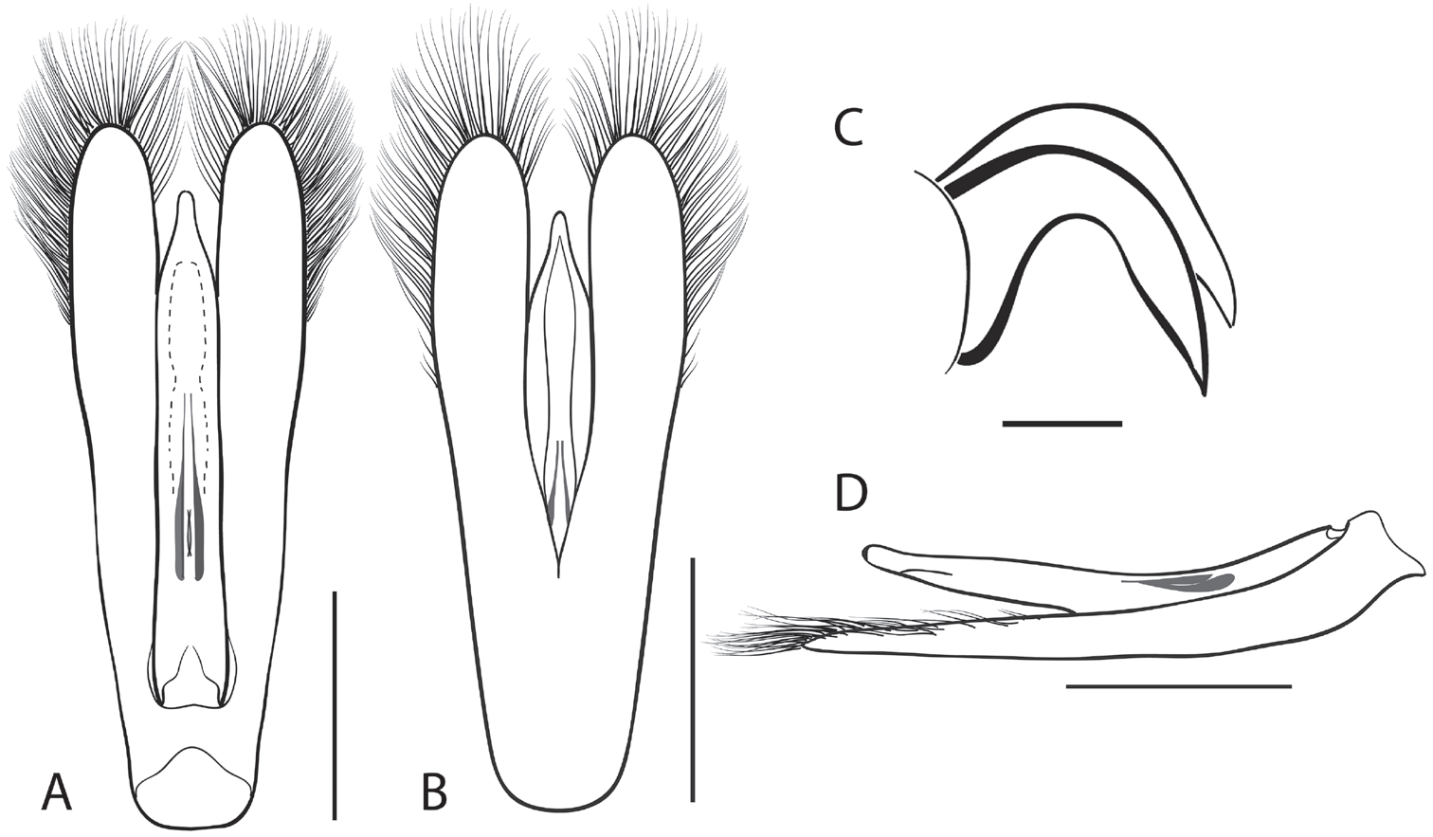
*Dineutus longimanus jamaicensis*. **A** aedeagus dorsal view **B** aedeagus ventral view **C** ♂ mesotarsal claws **D** aedeagus lateral view. Scale bar for **C** ≈ 0.10 mm all others ≈ 1 mm.

Figures 22 , 23 , 55

Dineutus (Dineutus) longimanus jamaicensis Ochs 1938: 88, *Dineutus longimanus jamaicensis*: Blackwelder 1944: 81, Dineutus (Rhombodineutus) longimanus: Guignot 1950: 127, Dineutus (Cyclinus) longimanus: Brinck 1955: 106.

##### Type locality.

Blue Mountains, Jamaica, near 4500 ft. MCZ Type No. 23058.

##### Specimens examined.

4

##### Type material examined.

Holotype (♂, pinned) “Blue Mts./ nr 4500 ft./ Aug. 13-20 [white label, typed black ink]// Jamaica/ 1934/ Darlington [white label, typed black ink]// 23058/ M.C.Z./ HoloType/ jamaicus/ ochs [red label, 23058, Holo, and jamaicus Ochs, handwritten in black ink, rest typed black ink]// Dineutus longimanus ssp. jamaicus/ Ochs/ 1937/ Type! [white label handwritten in black ink, handwriting appears to be Ochs’]// Dineutus longimanus/ jamaicus/ Ochs/ 1937/ type no. 23058 [white label handwritten in black ink and type no. 23058 in pencil, handwriting appears to be Ochs’]//” deposited in the MCZ.

##### Material examined.

**JAMAICA:** **St. Andrew Parish:** Maryland, Mamme River, 1.viii.1985, leg. M. Barrett, in clear stream (3 ex. FSCA).

##### Diagnosis.

Male (Fig. 22C–D). Size: 12.1–13.3 mm. Body form regularly elongate oval; elytral apices spinose, with sutural angle produce to a spine, and a second parasutural spine, with thorn-like serrations and irregularities present apically and apicolaterally, apicolateral sinuation mostly absent, elytra with reticulation strong laterally and apically, producing a bronzy appearance, medial disc with reticulation very weakly impressed and composed of smaller irregularly more transversely shaped cells accompanied by very shallowly impressed punctation, striae mostly effaced by reticulation, if evident at all faintly apparent medially on disc, lateral marginal depression of elytra absent; profemora with very small sub-apicoventral tooth; protibiae club-shaped; mesotarsal claws (Fig. 23C) with ventral margin regularly rounded and evenly narrowed apically; metacoxae with a decent covering of shallow punctures over most their ventral face, laterally only present on posterior half; venter lightly colored: reddish brown to reddish orange. Aedeagus (Figs 23A, B, D) with median lobe in dorsal view shorter than parameres, parallel sided, in apical 1/4 shallowly narrowed towards apex, apex regularly rounded, dorsally without narrow carina, ventrally sperm-groove narrow and parallel sided for most its length, apically briefly widened, in lateral view median lobe with dorsal margin straight, apex broadly rounded; parameres in dorsal view with lateral margins not noticeably laterally expanded, but very weakly sinuate medially, apically narrowly rounded.

Female (Fig. 22A–B). Size: 12.05 mm. Body form regularly elongate oval; elytral apices spinose, with sutural angle produce to a spine, and a second parasutural spine, with thorn-like serrations and irregularities present apically and apicolaterally, apicolateral sinuation mostly absent, elytra with reticulation strong laterally and apically, producing a bronzy appearance, medial disc with reticulation very weakly impressed and composed of smaller irregularly more transversely shaped cells accompanied by very shallowly impressed punctation, striae mostly effaced by reticulation, if evident at all faintly apparent medially on disc, lateral marginal depression of elytra absent; protibiae club-shaped; profemora without sub-apicoventral tooth; metacoxae with a decent covering of shallow punctures over most their ventral face, laterally only present on posterior half; venter lightly colored: reddish brown to reddish orange.

##### Differential diagnosis.

*Dineutus longimanus jamaicensis* is unique among the other subspecies of *Dineutus longimanus* in being larger in size (12.05–13.3 mm) and elongate oval in shape without apicolateral sinuation, and having the metacoxae with numerous shallow punctures covering most of their surface. The subspecies most similar to *Dineutus longimanus jamaicensis* is *Dineutus longimanus cubensis* and can be distinguished by the differences in dorsal punctation and metacoxal punctation provided in the key.

##### Distribution

**(Fig. 55B).** Jamaica (Leng and Mutchler 1914b; Ochs 1938)

##### Habitat.

Specimen label data indicate this is a lotic subspecies, with specimens collected from the Mammee river in St. Andrew, Jamaica (FSCA).

##### Discussion.

Similar to *Dineutus longimanus cubensis* not much is known about *Dineutus longimanus jamaicensis* aside from its taxonomy. It is currently only known from Jamaica.

#### Dineutus longimanus longimanus

Taxon classificationAnimaliaColeopteraGyrinidae

(Olivier, 1795)

**Figure 24.**
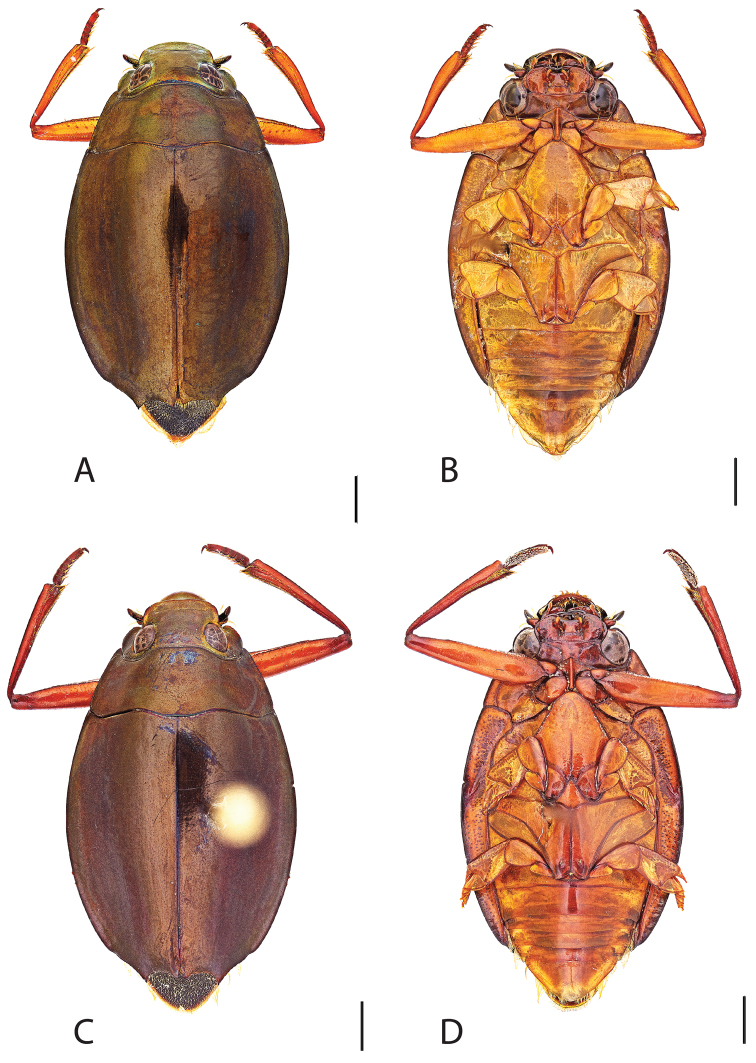
*Dineutus longimanus longimanus*. **A** ♀ dorsal habitus **B** ♀ ventral habitus **C** ♂ dorsal habitus **D** ♂ ventral habitus. All scale bars ≈ 2 mm.

**Figure 25.**
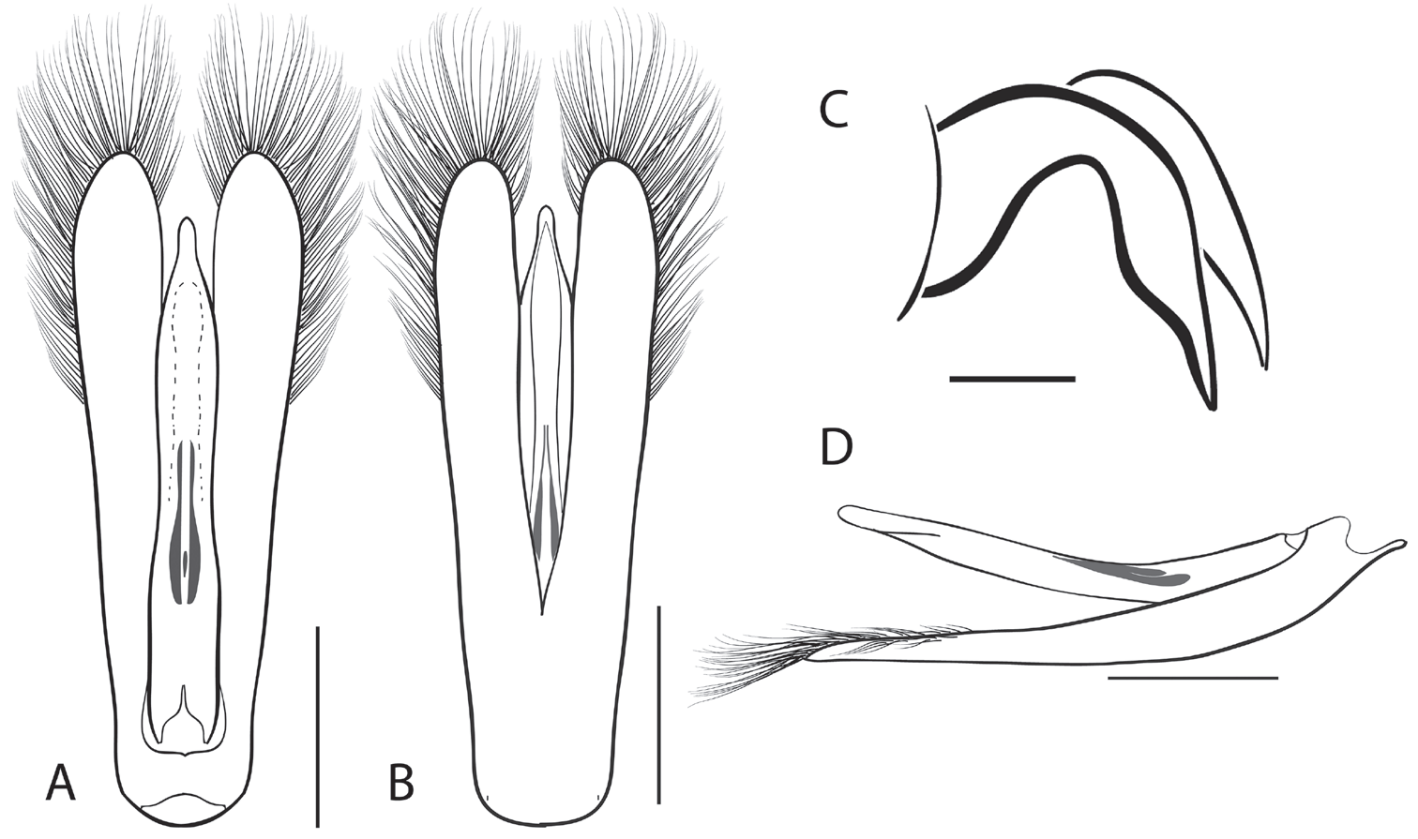
*Dineutus longimanus longimanus*. **A** aedeagus dorsal view **B** aedeagus ventral view **C** ♂ mesotarsal claws **D** aedeagus lateral view. Scale bar for **C** ≈ 0.10 mm all others ≈ 1 mm.

Figures 24 , 25 , 51 , 55

Gyrinus longimanus Olivier 1795: 701, *Gyrinus excisus*Forsberg 1821: 301 [synonymy by Aubé 1838], *Dineutes longimanus*: Aubé 1838a: 408, *Dineutus longimanus*: Ochs 1924: 5. Dineutus (Cyclinus) longimanus: Hatch 1925b: 488, Dineutus (Dineutus) longimanus: Ochs 1926a: 138, *Dineutus longimanus*: Blackwelder 1944: 81, Dineutus (Rhombodineutus) longimanus: Guignot 1950: 127, Dineutus (Cyclinus) longimanus: Brinck 1955: 106.

##### Type locality.

Saint-Domingue (= Hispaniola).

##### Specimens examined.

25

##### Type material examined.

*Gyrinus longimanus* Olivier, 1795: lectotype, here designated: (♂ pinned) “MUSEUM PARIS/ I. St. Domingue/ COLL. BOSC 1828 [beige label, typed black ink, except I. St. Domingue handwritten in black ink, handwriting unknown]// *Gyrinus longimanus*/ *I. St. Domingue Oliv.* [beige label with black border, handwritten in black ink, handwriting appears to be Olivier’s]// TYPE [red label, typed black ink]// LECTOTYPUS/ P. Brinck designavit 1955. [white label, typed black ink]// TYPE [red label, typed black ink]// LECTOTYPE [red label, typed black ink]//” deposited in the MNHN.

##### Material examined.

**DOMINICAN REPUBLIC:** **Pedernales Prov.:** W of Pedernales, on rd to border with Haiti, 18.154° ‘-71.582°, 13.v.2010, leg. G.J. Svenson, sweeping in dry for. & sec. veg. (17 ex. MSBA); N of Pedernales, La Aguita, 18°09.172'N, 71°44.786'W, 188 m, 21.vii.1999, leg. M.A. Ivie, Guerrero, & Dominici (5 ex. WIBF); 1 km N of Banano, Rio Mulitos, 18°09.258'N, 71°45.384'W, 290 m, 17.vi.2005, leg. G. Nearns, (2 ex. FSCA).

##### Diagnosis.

Male (Fig. 24C–D). Size: 12.3–13.9 mm. Body form regularly elongate oval, elytra laterally slightly broadened after basal half; elytral apices spinose, with sutural angle produce to a spine, and a second parasutural spine, with small thorn-like serrations and irregularities present apically but greatly reduced apicolaterally, apicolateral sinuation present and shallow, elytra with reticulation strong laterally and apically, producing a bronzy appearance, medial disc with reticulation more weakly impressed and composed of smaller transversely ovoid cells accompanied by shallowly impressed punctation, striae mostly effaced by reticulation, if evident at all faintly apparent medially on disc, lateral marginal depression of elytra shallow; profemora with very small sub-apicoventral tooth; protibiae club-shaped; mesotarsal claws (Fig. 25C) with ventral margin with a well developed denticle; metacoxae with a sparse covering of very shallow punctures over most their ventral face, laterally only present on posterior margin; venter lightly colored: reddish orange to orangey yellow. Aedeagus (Fig. 25A, B, D) with median lobe in dorsal view shorter than parameres, nearly parallel sided, broader basally and weakly narrowed apicad, in apical 1/4 shallowly narrowed towards apex, apex regularly rounded, dorsally without narrow carina, ventrally sperm-groove narrow and parallel sided, in lateral view median lobe with dorsal margin straight, apex angularly rounded; parameres in dorsal view with lateral margins very weakly laterally expanded in apical 1/2, apically broadly rounded.

Female (Fig. 24A–B). Size: 12.3–13.7 mm. Body form regularly elongate oval; elytral apices spinose, with sutural angle produce to a spine, and a second parasutural spine, with small thorn-like serrations and irregularities present apically but greatly reduced apicolaterally, apicolateral sinuation present and shallow, elytra with reticulation strong laterally and apically, producing a bronzy appearance, medial disc with reticulation more weakly impressed and composed of smaller transversely ovoid cells accompanied by very shallowly impressed punctation, striae mostly effaced by reticulation, if evident at all faintly apparent medially on disc, lateral marginal depression of elytra absent; protibiae club-shaped; profemora without sub-apicoventral tooth; metacoxae with a sparse covering of very shallow punctures over most their ventral face, laterally only present on posterior margin; venter lightly colored: reddish orange to orangey yellow.

##### Differential diagnosis.

*Dineutus longimanus longimanus* is unique among other subspecies of *Dineutus longimanus* in being elongate oval and broadened posteriorly in the male after basal half of elytra, and having the metacoxae with sparse but present punctation. The subspecies most similar to *Dineutus longimanus longimanus* is *Dineutus longimanus portoricensis* but can be distinguished from *Dineutus longimanus portoricensis* in being smaller in size (12.3–13.9 mm) and the aedeagus lacking a dorsal carina.

##### Distribution

**(Fig. 55B).** Haiti, Dominican Republic, (Leng and Mutchler 1914a; Ochs 1938; Wood 1962)

##### Habitat.

Lotic species collected in streams throughout the Dominican Republic (M. Fikáček pers. comm.).

##### Discussion.

Similar to other species not much is known about this species aside from its taxonomy. The extant of range of this and other subspecies of *Dineutus longimanus* may be obscured due to imprecise identification of subspecies.

##### Type designation.

Olivier (1795) in his original description of *Gyrinus longimanus* states it is found in St. Domingue and was described from the cabinet of M. Bosc. A single specimen in the MNHN general collection has a label indicating both the aforementioned locality and being from Bosc’s collection, as well as a label in the handwriting of Olivier with the I.D. of *Gyrinus longimanus*, therefore this specimen (Fig. 51E) is here designated as the lectotype for *Dineutus longimanus longimanus*.

#### Dineutus longimanus portoricensis

Taxon classificationAnimaliaColeopteraGyrinidae

Ochs, 1924

**Figure 26.**
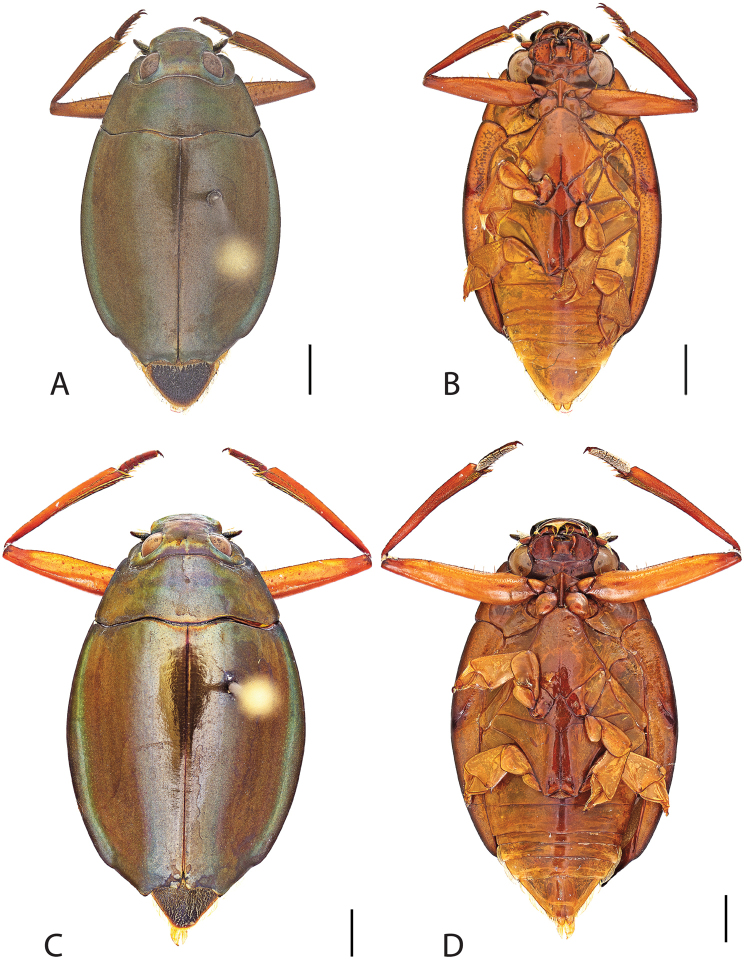
*Dineutus longimanus portoricensis*. **A** ♀ dorsal habitus **B** ♀ ventral habitus **C** ♂ dorsal habitus **D** ♂ ventral habitus. All scale bars ≈ 2 mm.

**Figure 27.**
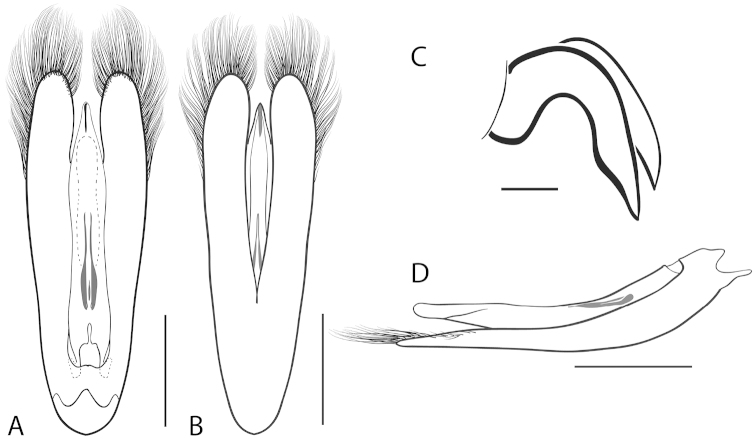
*Dineutus longimanus portoricensis*. **A** aedeagus dorsal view **B** aedeagus ventral view **C** ♂ mesotarsal claws **D** aedeagus lateral view. Scale bar for **C** ≈ 0.10 mm all others ≈ 1 mm.

Figures 26 , 27 , 55

Dineutus longimanus portoricensis Ochs 1924: 5, Dineutus (Cyclinus) longimanus: Hatch 1925b: 448, Dineutus (Dineutus) longimanus portoricensis: Ochs 1926a: 138, *Dineutus longimanus portoricensis*: Blackwelder 1944: 81, Dineutus (Rhombodineutus) longimanus: Guignot 1950: 127, Dineutus (Cyclinus) longimanus: Brinck 1955: 106

##### Type locality.

Puerto Rico, Aibonito.

##### Specimens examined.

27

##### Type material examined.

Holotype (♂, pinned, aedeagus pointed to specimen) “Aibonito, P. R., June 1-3, 1915/Amer.Mus.Nat.Hist., Dept. Invert. Zool. No. 28073/HOLOTYPE/Dineutus longimanus Oliv.subsp. portoricensis Ochs 1924, Typus ♂” AMNH type catalogue No. 434.

##### Material examined.

**PUERTO RICO:** “Hwy-31, Km. 15.4 nr. PasoSecoJct.”, 122 m, 1.viii.1963, leg. P.J. Spangler, (4 ex. WIBF); **Germán:** Río Cain, at PR361, 1 rd. mi. N jct. PR396, N of San Germán, 18°07.062'N, 67°01.518'W, 103 m, 14.v.2009, leg. C.B. Barr, EMEC 654754, 654755, 654757 (3 ex. EMEC); **Lares:** Río Camuy, E off PR134 1.2 rd.mi. N jct. PR111, NE of Lares, 18°18.204'N, 66°49.445'W, 278 m, 16.v.2009, leg. W.D. Shepard, EMEC 654756, 654758, 654759, 654760 (4 ex. EMEC); **Maunabo:** Río Guayanés, at PR181 just S jct. PR182, N Patillas, 18°03.397'N, 65°59.004'W, 422 m, 9.v.2009, leg. C.B. Barr, EMEC 654749-654753 (5 ex. EMEC); **San Patillas:** trib. Río Grande de Patillas at PR184, Bosque Carite Charco Azul Rec. Center, 18°05.460'N, 66°02.150'W, 597 m, 10.v.2009, leg. C.B. Barr, EMEC 654746-654748 (3 ex. EMEC); **Uatado:** trib. Río Caonillas E off PR 612, 2.3 rd. mi. N jct. PR140, NE of Utuado, S Lago dos Bocas, 18°18.177'N, 66°38.708'W, 99 m, 17.v.2009, leg. C.B. Barr, EMEC 654738-654745 (8 ex. EMEC).

##### Diagnosis.

Male (Fig. 26C–D). Size: 12.3–14.5 mm. Body form regularly elongate oval, elytra laterally slightly broadened after basal half; elytral apices spinose, with sutural angle produce to a spine, and a second parasutural spine, with small thorn-like serrations and irregularities present apically but greatly reduced apicolaterally, apicolateral sinuation present and shallow, elytra with reticulation strong laterally and apically, producing a bronzy appearance, medial disc with reticulation more weakly impressed and composed of smaller transversely ovoid cells along with very shallow punctation, striae mostly effaced by reticulation, if evident at all faintly apparent medially on disc, lateral marginal depression of elytra present and accompanied with a green sheen; profemora with very small sub-apicoventral tooth; protibiae club-shaped; mesotarsal claws (Fig. 27C) with ventral margin with a weak denticle; metacoxae with a sparse covering of very shallow punctures that are almost indistinguishable over most their ventral face, laterally only present on posterior margin; venter lightly colored: reddish orange to yellow-orange. Aedeagus (Fig. 27A, B, D) with median lobe in dorsal view just shorter than parameres, broadest basally, becoming narrowed apicad after basal 1/3, then angularly narrowed again in apical 1/3 toward apex, apex narrowly rounded, dorsally with narrow carina, ventrally sperm-groove narrow and parallel sided, in lateral view median lobe with dorsal margin straight, slightly angled ventrally toward apex, apex broadly rounded; parameres in dorsal view with lateral margins not noticeably laterally expanded, apically broadly rounded.

Female (Fig. 26A–B). Size: 11.2–13.4 mm. Body form regularly elongate oval; elytral apices spinose, with sutural angle produce to a spine, and a second parasutural spine, with small thorn-like serrations and irregularities present apically but greatly reduced apicolaterally, apicolateral sinuation present and shallow, elytra with reticulation strong laterally and apically, producing a bronzy appearance, medial disc with reticulation more weakly impressed and composed of smaller transversely ovoid sculpticells along with very shallowly impressed punctation, striae mostly effaced by reticulation, if evident at all faintly apparent medially on disc, lateral marginal depression of elytra present and accompanied with a green sheen; profemora without sub-apicoventral tooth; protibiae club-shaped; metacoxae with a sparse covering of very shallow punctures that are almost indistinguishable over most their ventral face, laterally only present on posterior margin; venter lightly colored: reddish orange to yellow-orange.

##### Differential diagnosis.

*Dineutus longimanus portoricensis* can easily be distinguished from other subspecies of *Dineutus longimanus* in being large in size (11.2–14.5 mm), elytral margin with a lateral green sheen, and most distinctly in the form of the aedeagus. The species most similar to *Dineutus longimanus portoricensis* is *Dineutus longimanus longimanus* but can easily be distinguished from it in being larger in size with the lateral marginal depression having a green sheen, and having the aedeagus with a dorsal carina.

##### Distribution

**(Fig. 55B).** Puerto Rico (Leng and Mutchler 1914b; Ochs 1924).

##### Habitat.

Few specimens included habitat information. One that did, mentions a roadside stream (WIBF). Historical records also indicate this is a lotic subspecies (Ochs 1924).

##### Discussion.

Not much is known about this species aside from its taxonomy, and similar to *Dineutus longimanus longimanus* the true extant of its range is obscured due to imprecise identification of the subspecies.

#### Dineutus mexicanus

Taxon classificationAnimaliaColeopteraGyrinidae

Ochs, 1925 stat. n.

**Figure 28.**
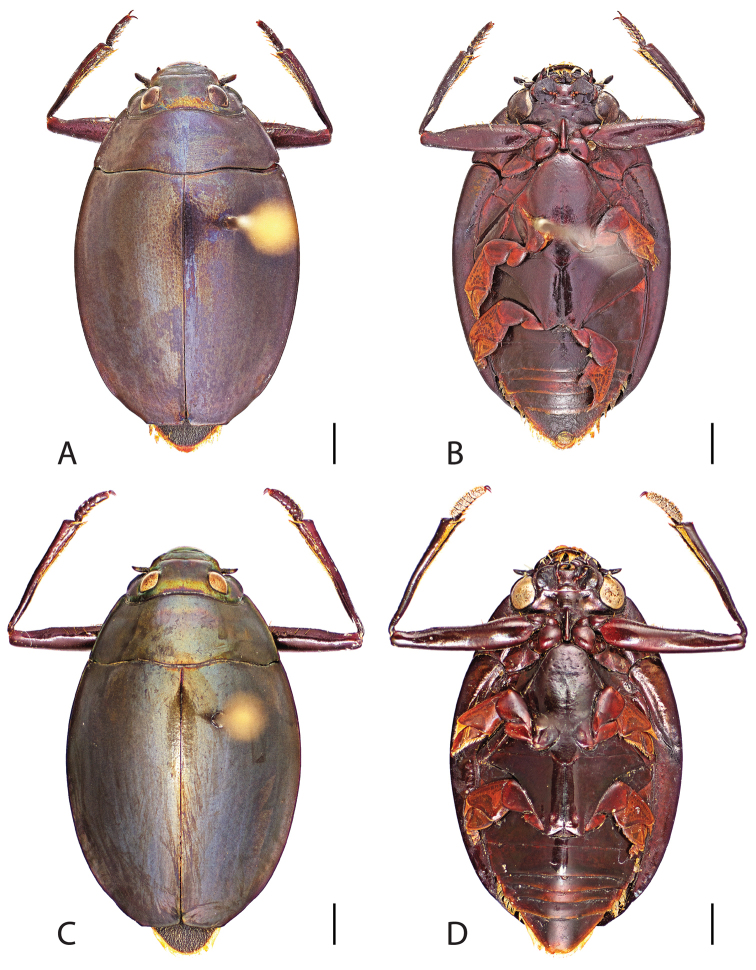
*Dineutus mexicanus*. **A** ♀ dorsal habitus **B** ♀ ventral habitus **C** ♂ dorsal habitus **D** ♂ ventral habitus. All scale bars ≈ 2 mm.

**Figure 29.**
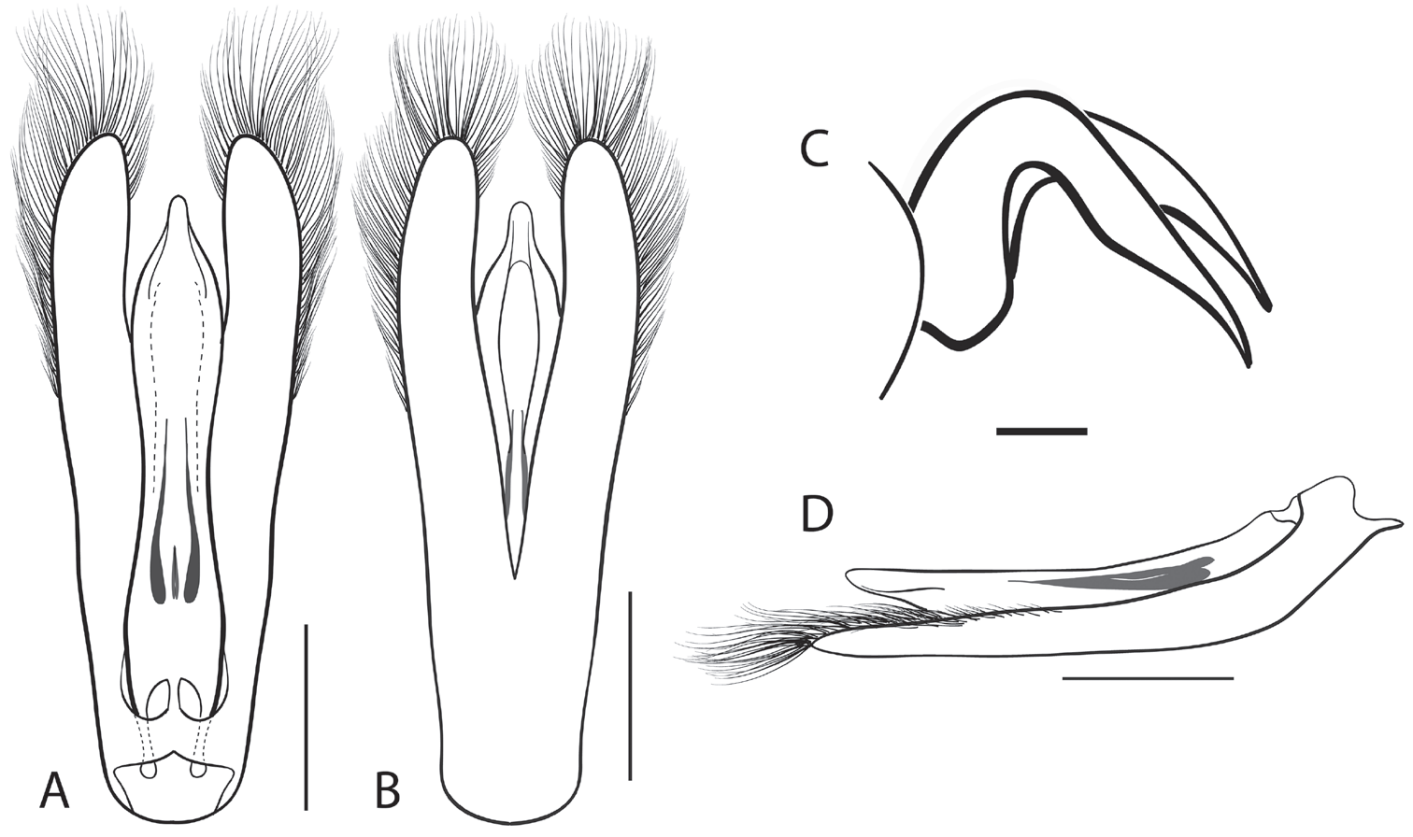
*Dineutus mexicanus*. **A** aedeagus dorsal view **B** aedeagus ventral view **C** ♂ mesotarsal claws **D** aedeagus lateral view. Scale bar for **C** ≈ 0.10 mm all others ≈ 1 mm.

**Figure 30.**
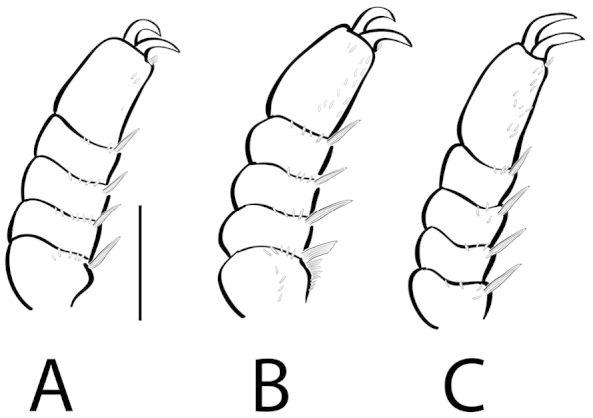
♂ protarsus of **A** *Dineutus mexicanus* **B** *Dineutus* sp. from nr. Tapanatepec, Oaxaca, Mexico **C** *Dineutus truncatus*. Scale bar ≈ 1 mm.

Figures 28 , 29 , 30 , 51 , 54

Dineutus truncatus mexicanus Ochs 1925: 13, Dineutus (Dineutus) truncatus mexicanus: Ochs 1926a: 138, *Dineutus truncatus mexicanus*: Blackwelder 1944: 81, Dineutus (Dineutus) truncatus mexicanus: Ochs 1949: 289, Dineutus (Cyclinus) truncatus mexicanus: Brinck 1955: 106, *Dineutus truncatus mexicanus*: Arce-Pérez and Roughley 1999: 84.

##### Type locality.

Mexico.

##### Specimens examined.

44

##### Type material examined.

Holotype (♂ with aedeagus dissected onto card point): “10393” [white label] // “Mexico Schl.” [green label, handwritten in ink, handwriting unknown] // “Hist.-Coll. (Coleoptera)/ Nr. 10393/ Dineutes spec./ Mexico, Schl./ Zool. Mus. Berlin” [green label] // “Dineutestruncatus/ Sharp/ determ. Ahlwarth” [white label, part handwritten, determination portion typed]// “subspecies/ mexicanus/ Type! Ochs 1924”, [white label, appears to be hand written by Ochs (Horn and Kahle, 1990)]// “subsp. * / mexicanus/ Ochs/ Mexico.” [green label, hand written again by Ochs]// “HOLOTYPUS/ Dineutes truncatus/ ssp. mexicanus Ochs, 1925/ labelled by MNHUB 2012” [red label, typed]// deposited in the ZMHB. Paratypes: (♀ specimen missing left protarsus): “10393” [white label]// “Hits.-Coll. (Coleoptera)/ Nr. 10393/ Dineutes spec./ Mexico, Schl./ Zool. Mus. Berlin” [green label]// “? PARATYPUS/ Dineutes truncatus/ ssp. mexicanus Ochs, 1925/ labelled by MNHUB 2012” [red label]//; (♀ specimen): “4” [white label, handwritten]// “85373” [white label, handwritten]// “Mochtlan/ Guerrero/ Baron” [white label, handwritten]// “Coll./ Harford”, white label “B.C.A. Col. I. 2./ Dineutes/ truncatus,/ Sharp.” [white label, handwritten except for determination]// “Dineutes truncatus/ Sharp/ determ. Ahlwarth” [red label]// “PARATYPUS/ Dineutes truncatus/ ssp. mexicanus Ochs, 1925/ labelled by MNHUB 2012” [red label]//; (♂ specimen): “Mexico/ J.Flohr G.” [white label]// “94618” [white label, handwritten]// “Hist.-Coll. (Coleoptera)/ Nr. 94618/ Dineutes truncatus Sharp/ Mixco, Juquila, Coll. Flohr/ Zool. Mus. Berlin/” [green label]// “truncatus/ Sh” [white label]// “? PARATYPUS/ Dineutes truncatus/ ssp. mexicanus Ochs, 1925/ labelled by MNHUB 2012” [red label]//; (A single right elytron, glued to card, specimen of unknown sex) “94618” [white label, handwritten]// “Mexico/ J.Flohr G.” [white label, handwritten]// “Hist.-Coll. (Coleoptera)/ Nr. 94618/ Dineutes truncatus Sharp/ Mexico, Juquila, Coll. Flohr/ Zool. Mus. Berlin” [green label]// “? PARATYPUS/ Dineutes truncatus/ ssp. mexicanus Ochs, 1925/ labelled by MNHUB 2012” [red label]//; (♂ specimen headless): “Juquila/ 15” [white label, handwritten]//, “94618” [white label, handwritten]// “Mexico, J.Flohr G.” [green label]// “Hist.-Coll. (Coleoptera)/ Nr. 94618/ Dineutes truncatus Sharp/ Mexico, Juguila, Coll. Flohr/ Zool. Mus. Berlin” [green label]// “PARATYPUS/ Dineutes truncatus/ ssp. mexicanus Ochs, 1925/ labelled by MNHUB 2012” [red label]// (6: ZMHB).

##### Material examined.

**EL SALVADOR:** Los Chorros Park, 16.vii.1961 (1 ex. UCRC); **Chalatenango:** San Jose del Sacare, 15.iii.1927, leg. R.A. Stirton (2 ex. KSEM); **Tamanique:** 1000 m, 8.xii.1972, leg. S. & L. Steinhauser (5 ex. FSCA); same as previous except: 3.vii.1972, leg. S. & L. Steinhauser (4 ex. FSCA). **GUATEMALA:** **Zacapa:** Sierra de los Minas, “El Naranjo”, S slope below San Lorenzo Mine, 15.07329 -89.68481, 1600-1700m, 21-24.v.2010, leg. P. Skelley, oak forest at light (1 ex. FSCA). **HONDURAS:** “nr Progreso”, Mico Quemado Mts, 6.xii.1958, leg. J.G. Matthysse, (2 ex. MSBA). **MEXICO:** **Guerrero:** Malinaltepec, Aserradero, 1500m, 2.xi.2000, leg. F. Pacheco, 24-8 (1 ex. IEXA); **México:** Villa Guerrero, Porfirio Díaz, Las Puentes, 14.iv.1990, leg. R. Arce, 7-4 (1 ex. IEXA); **Michoacán:** Chinicuila, Sierra de Coalcomán, La Nuez, Cañada El Colorín, , 15.ix.2003, leg. R. Novelo (2 ex. IEXA); same as previous except:16.ii.2005, leg. Gómex y Novelo, (2 ex. IEXA); **Nayarit:** 7mi N. Tetitlan, 14.vi.1962, leg. D.H. Janzen, EMEC 204752; 204756; 204853; 204856; 204866; 204894 (6 ex. EMEC); 8 mi SE San Blas, 27.vi.1967, leg. A.R. Hardy, (2 ex. UCRC); **Oaxaca:** San Juan Bautista Cuicatlán, Río La Concepción, 2.iv.1989 (4 ex. IEXA); km 84 carr 175 Oaxaca-Tuxtepec, 13.vi.1992, leg. R. Novelo (1 ex. IEXA); San José Independencia, Cerro el Vidrio, 1900 m, 25.v.2004, leg. G. Nogueira, (1 ex. IEXA); **Veracruz:** 6.vii.1965, leg. G.N. Ross, (2 ex. FSCA).

##### Diagnosis.

Male (Fig. 28C–D): Size: 14.5–16.8 mm. Body form regularly oval; elytral apices truncate, lateral corner of truncation distinctly angled, serration reduced to small pointed bumps and/or irregularities, apicolateral margin often faintly sinuate, elytral striae visible anteromedially near suture, disappearing laterally and prior to elytral apices, dense microreticulation covering much of elytra and pronotum; ulimate protarsomere (Fig. 30A) less than ca. 2× as long as wide; protibiae club-shaped; profemora with small acute sub-apicoventral tooth; mesotarsal claw (Fig. 29C) with ventral margin with weak denticle; venter darkly colored, usually black to dark reddish brown, mesothoracic and metathoracic legs usually lighter in coloration, as well as apex of abdomen; Aedeagus (Fig. 29A, B, D) with median lobe in dorsal view weakly constricted medially, being roundly expanded in apical 1/3, acuminate in apical 1/5, with apex broadly rounded, in lateral view, apex of median lobe broadly rounded, ventrally median lobe with parallel sided sperm-groove basally, being shortly expanded and rounded apically, parameres very weakly laterally expanded in apical 1/2.

Female (Fig. 28A–B): Size: 13.9–15.9 mm. Body form regularly elongate oval; elytral apices truncate, lateral corner of truncation distinctly angled, serration reduced to small pointed bumps and/or irregularities, apicolateral margin often faintly sinuate, elytral striae often indistinct, visible upon close examination, dense microreticulation covering entirety of elytra and pronotum, giving elytra and the pronotum a polished metal feel; ulimate protarsomere less than ca. 2× as long as wide; protibiae club-shaped; profemora without sub-apicoventral tooth; venter darkly colored, usually black to dark reddish brown, mesothoracic and metathoracic legs usually lighter in coloration, as well as apex of abdomen.

##### Differential diagnosis.

*Dineutus mexicanus* is unique among all other North American *Dineutus* in having truncate elytra, with the lateral angle distinct, apicolateral sinuation weakly present, blunt serrations and irregularities present apically, males with a small acute profemoral sub-apicoventral tooth, and in the form of the aedeagus (Fig. 29A). The species most similar to *Dineutus mexicanus* is *Dineutus truncatus* (of which it used to be considered a subspecies of). The elytral truncature of *Dineutus mexicanus* differs in form having the apicolateral margin of the eyltra weakly sinuate, the lateral angle distinct, and the striae being more evident than those of *Dineutus truncatus*, especially in the males. There are also differences in body form discussed in the differential diagnosis section of *Dineutus truncatus*.

Males of *Dineutus mexicanus* can further be separated from *Dineutus truncatus* by the form of the protarsi (Fig. 30A), the mesotarsal claw shape, and the form of the aedeagus. See the differential diagnosis under *Dineutus truncatus* for details.

For females see the differential diagnosis section for *Dineutus truncatus*.

##### Distribution

**(Fig. 54D).** From Mexico to El Salvador (Arce-Pérez and Roughley 1999; Ochs 1949).

##### Habitat.

Unknown. Most specimens observed for this study were old, collected during the late 1950’s to the early 1970’s containing only locality data and lacking habitat data. A single specimen from Guatamela was collected in 2010 with the habitat data given as “oak forest at light” (FSCA).

##### Discussion.

The elevation of *Dineutus mexicanus* to full species status was based on the noticeable and significant differences between the aedeagi of *Dineutus truncatus* and *Dineutus mexicanus* as well as the external characters discussed in the differential diagnosis section for *Dineutus truncatus*. These external characters include the protarsi, mesotarsal claws, and the elytral truncature, all found to be reliable indicators of species boundaries in North American *Dineutus*. The holotype (Fig. 51F) has been examined for this study allowing the characters of *Dineutus mexicanus* to be unambiguously known and the species status elevation to be embedded securely in accordance with differences found in the type.

The extent of the range of *Dineutus mexicanus* is currently unclear, due to the unclear identity of historical *Dineutus truncatus* records. For the current study the eastern most record of *Dineutus mexicanus* is from the Mico Quemado mountains near Progresso, Yoro, Hondoras (KSEM). Régimbart (1882) mentions a variety of *Dineutus truncatus* from Honduras, which Ochs (1949) now appears correct in that this record actually refers to *Dineutus mexicanus*. Specimens of *Dineutus mexicanus* for this study were also found in El Salvador, from several localities representing the southern-most confirmed record of *Dineutus mexicanus*. The western-most records for *Dineutus mexicanus* from this study are from Nayarit, Mexico, 8 miles southeast of San Blas. Confirmed specimens of *Dineutus mexicanus* are recorded from numerous records between these east and west localities including Guatemala, and the Mexican states of Veracruz, Oaxaca, and Guerrero. It is unclear how far north the range of *Dineutus mexicanus* extends in Central America and the Isthmus of Tehuanatepec. Based on the limited range data (Fig. 54D), it appears that *Dineutus mexicanus* may be a mountain endemic, following the Sierra de Madre de Chiapas through Chiapas, Mexico, Guatemala, and into El Salvador and Honduras, going northwest to southeast through its range. Therefore, *Dineutus mexicanus* may indeed be absent from the Northern Guatemala, Belize, and the Mexican states of Quintana Roo, Yucatan, Campeche, and Tabasco. In the west *Dineutus mexicanus* records again overlap with mountain ranges, being found in western Oaxaca in the Sierra Madre del Sur, going northwest along the western portion of the Trans-Mexican Volcanic Belt and into the Sierra Madre Occidental, as far north as Nayarit. Ochs (1949) lists a record for Tamaulipas, Mexico near Victoria, possibly for the Sierra Madre Oriental.

#### Dineutus nigrior

Taxon classificationAnimaliaColeopteraGyrinidae

Roberts, 1895

**Figure 31.**
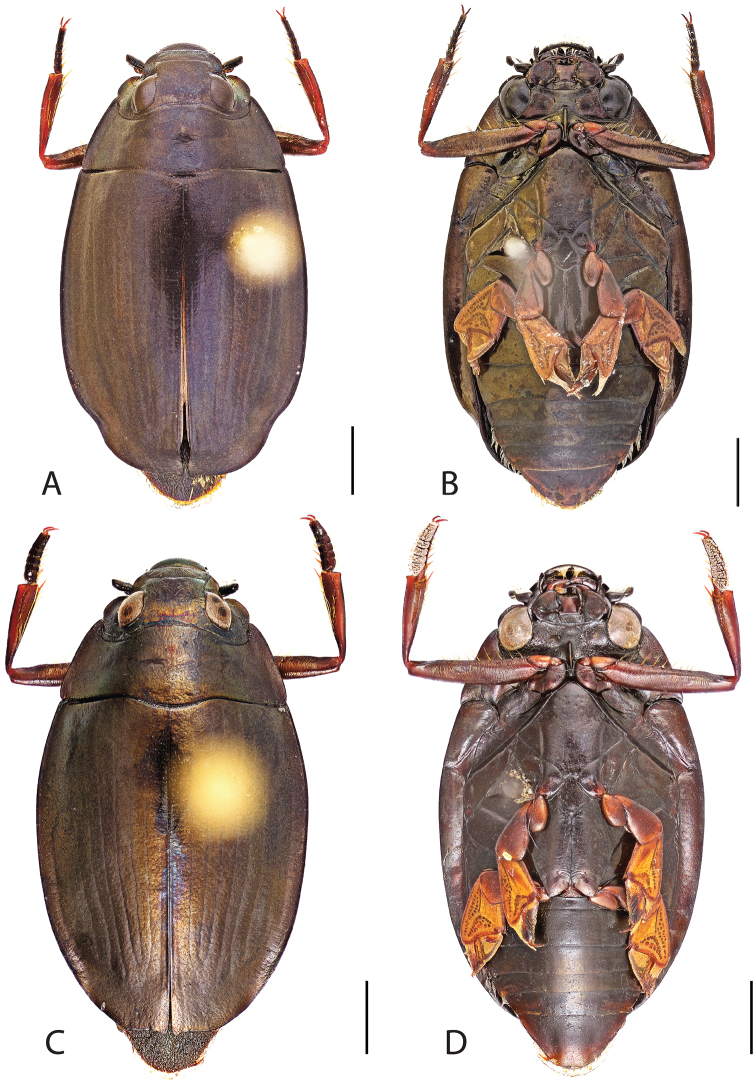
*Dineutus nigrior*. **A** ♀ dorsal habitus **B** ♀ ventral habitus **C** ♂ dorsal habitus **D** ♂ ventral habitus. All scale bars ≈ 2 mm.

**Figure 32.**
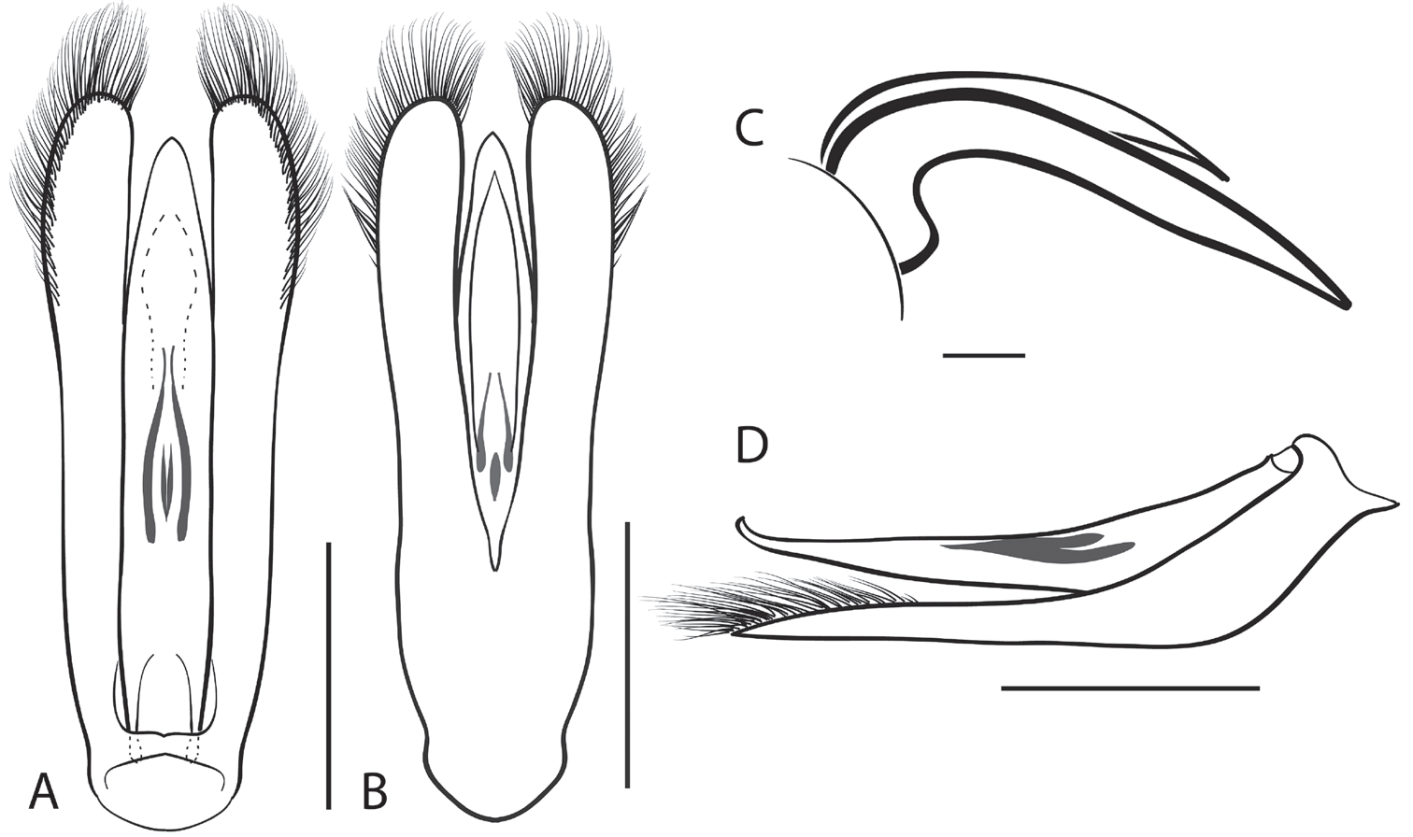
*Dineutus nigrior*. **A** aedeagus dorsal view **B** aedeagus ventral view **C** ♂ mesotarsal claws **D** aedeagus lateral view. Scale bar for **C** ≈ 0.10 mm all others ≈ 1 mm.

Figures 31 , 32 , 52

Dineutes nigrior Roberts 1895: 280, Dineutus (Cyclinus) nigrior: Hatch 1926a: 311, Dineutus (Cyclous) nigrior: Hatch 1927: 28, Dineutus (Cyclinus) nigrior: Hatch 1930: 20. *Dineutes nigrior*: Brimley 1938: 132, Dineutus (Cyclinus) nigrior: Guignot 1950: 126, *Dineutus nigrior*: Ferkinhoff and Gunderson 1983: 19.

##### Type locality.

USA, Vermont.

##### Specimens examined.

**34**

##### Type material examined.

Syntype (♂ pinned, aedeagus extruded) “Bengtn. Co., Vt./Acc.4858/ LECTOTYPE/ nigrior ♂ type # 1 C.H.R./LECTOTYPE Dineutus nigrior Desig: R.P. Withington III 1998/ Dineutus nigrior Roberts 1895 Det : L. Cook 2005” AMNH type catalogue no. 498.

##### Material examined.

**U.S.A.:** **Delaware:** Sussex Co., Milsboro, 13.v.1973, leg. T.E. Rogers (1 ex. FSCA); **Florida:** Alachua Co., Gainesville, 27.vi.1961, leg. R.F. Bussey (1 ex. FSCA); Liberty Co., Torreya State Park, 25.v.1981, leg. J.R. Watts, at U.V. (1 ex. FSCA); **Indiana:** Crawford Co., Grantsburg, 18.vii.1965, leg. D. Eckert, Blacklight trap (1 ex. FSCA); **Maryland:** Prince George’s Co., College Park, 16.vi.1948, leg. B.K. Detler, in pond (1 ex. FSCA); Prince George’s CO., Blue Pond, 29.ix.1949, leg. H.L. Dozier, (1 ex. FSCA); **Massachusetts:** Suffolk Co, Forest Hills, 18.iv.1919 (1 ex. FSCA); **Michigan:** Allegan Co., State Game Area, 17.vii.1986, leg. J.A. Shuey (1 ex. MTEC); Cheboygan Co., Douglas Lake, 30.vii.1927, leg. H.B. Hungerford (1 ex. KSEM); same as previous except: “Mud L”, 31.vii.1923 (1 ex. KSEM); Washtenaw Co., “118 8h”, 24.iv.1921, leg. M.H. Hatch (1 ex. FSCA); **Minnesota:** Clearwater Co., Elk Springs, Itasca State Park, 12.viii.1965, leg. J.S. Nordin, attracted to U.V. (2 ex. FSCA); same as previous except: 7.vii.1965 (1 ex. FSCA); Morner Co., nr. Grand Meadow, roadside park, 18.viii.1965, leg. R.H. Arnett, in dammed pond (2 ex. FSCA); **Missouri:** Wayne Co., 3.2 mi WSW of Patterson Co. Rd. 332, deciduous Ozark forest and old field flora, 4.vii.1988, leg. H.M. Webber, at U.V. light (1 ex. FSCA); **New Jersey:** Cumberland Co., “Dividing Ck. Hansey Creek Rd.”, edge of salt marshes, 30.ix.1989, leg. D. Schloeitzer, at UV light (1 ex. FSCA); **New York:** Schuyler Co., Texas Hollow State Wildlife Area, 1.ix.1999, leg. K.B. Miller (1 ex. MSBA); Westchester Co., White Plains, 5.ix.1922, leg. E.H.P. Squire (1 ex. FSCA); same as previous except: 3.viii.1923 (2 ex. FSCA); same as previous except: 19.v.1923 (1 ex. FSCA); same as previous except: 30.v.1923 (1 ex. FSCA); same as previous except: 10.iii.1923 (1 ex. FSCA); same as previous except: 18.x.1924 (2 ex. FSCA); same as previous except: 12.iv.1925 (1 ex. FSCA); same as previous except: 19.v.1925 (1 ex. FSCA); Westchester Co., Montrose, 4.vii.1932, leg. C.L. Ragot (1 ex. FSCA); **Pennsylvania:** Sullivan Co., Picketts Glen St. Pk., 5.vii.1960, leg. G.W. Byers (2 ex. KSEM); **Virginia:** Giles Co., Mt. Lake Biol. Stat., 30.vi.1968, leg. H. Greenbaum (1 ex. FSCA); **Wisconsin:** Douglas Co., State Hunting Grounds, “T44N.R12W.Sec.11”, 23.vi.1999, leg. A Ramsdale, black light in barrens (1 ex. MTEC).

##### Diagnosis.

Male (Fig. 31C–D): Size: 11.1–11.7 mm. Body form narrowly oval; elytral apices with sutural angle produced into a point, elytral striae faint basally becoming more evident apically and laterally; profemora without a sub-apicoventral tooth; protibiae wedge-shaped, with distolateral margin produced; mesotarsal claws markedly asymmetrical (Fig. 32C), anterior mesotarsal claw larger than posterior claw, venter darkly colored, reddish brown to black with mesothoracic and metathoracic legs lighter in color; Aedeagus (Fig. 32A, B, D) median lobe in dorsal view narrowed in apical 1/4, shortly rounded apically, in lateral view apex of median lobe strongly curved dorsally, parameres strongly curved in lateral view after basal 1/3.

Female (Fig. 31A–B): Size: 11.6–11.7 mm. Body form narrowly oval; elytral apices produced and rounded, with sutural angle produced into a point, apicolateral sinuation strong, elytral striae faint basally, becoming more evident apically and laterally; profemora without sub-apicoventral tooth; protibiae laterally weakly curved, distolateral margin weakly expanded; venter darkly colored, reddish brown to black venter darkly colored, reddish brown to black with mesothoracic and metathoracic legs lighter in color.

##### Differential diagnosis.

This species is unique among all other species of North American *Dineutus* in the extremely large size and assymetrical nature of the male mesotarsal claws (Fig. 32C) and the form of male aedeagus (Fig. 32A, B, D). The species most similar to *Dineutus nigrior* are *Dineutus assimilis* and *Dineutus hornii*, of the two the former most closely resembles *Dineutus nigrior* especially the female members of the species. Females of all three species very closely resemble one another and require careful consideration for identification. Males of the species can be fairly readily distinguished externally, and aedeagal dissections can indisputably separate males of the species.

In general males of *Dineutus nigrior* can be distinguished from members of *Dineutus assimilis* and *Dineutus hornii* by size. *Dineutus nigrior* males are larger (Size: 11.1–11.7 mm) than males of both *Dineutus assimilis* and *Dineutus hornii*. *Dineutus nigrior* males differ from males of *Dineutus hornii* in having the sutural angle of the elytral apices produced into a point, and the venter of *Dineutus nigrior* is much more darkly colored than that of *Dineutus hornii*, with the epipleura being similarly colored as the thoracic ventrites. Males of *Dineutus nigrior* can be distinguished from both *Dineutus assimilis* and *Dineutus hornii* in having the mesotarsal claws markedly asymmetrical (Fig. 32C) with the anterior tarsal claw being larger than the posterior claw, while in both *Dineutus assimilis* (Fig. 9C) and in *Dineutus hornii* (Fig. 19C) the mesotarsal claws are similar in size. The distolateral margin of the protibiae in *Dineutus nigrior* is produced while in *Dineutus assimilis* and *Dineutus hornii* the margin is straight or nearly so. The aedeagus of *Dineutus nigrior* (Fig. 32A) is most similar to *Dineutus hornii* (Fig. 19A) but can be distinguished from *Dineutus hornii* in having the apex of the median lobe strongly curved dorsally in lateral view (Fig. 32D).

The females of *Dineutus nigrior* are also generally larger (Size: 11.6–11.7 mm) than those of *Dineutus assimilis* and *Dineutus hornii*, but unlike the case in the males, females of each of these three species have the sutural angles of the elytra produced into a point. However, the shape of the apices differs between them. In females of *Dineutus nigrior* the apices of the elytra are regularly rounded, and this situation is most different from *Dineutus hornii* where the apices are generally angled towards the sutural production. *Dineutus nigrior* females can be separated from both *Dineutus assimilis* and from *Dineutus hornii* in having the distolateral protibial margin produced, similar to the condition in males, but more weakly expanded.

##### Distribution

**(Fig. 52C).** Extreme southeastern Canada from Manitoba to Nova Scotia (Majka 2008; Majka and Kenner 2009; Roughley 1991) and most of the eastern half of the United States as far south as northern Florida (Epler 2010; Folkerts 1978; Gordon and Post 1965; Hilsenhoff 1990; Malcolm 1971; Régimbart 1907; Roberts 1895; Sanderson 1982; Whiteman and Sites 2003; Wood 1962).

##### Habitat.

Primarily lentic species, found only infrequently in streams (Roberts 1895; Hilsenhoff 1990). In Canada, *Dineutus nigrior* prefers semi-boggy lakes (Morrissette 1979). In the Missouri Prairie Region, Whiteman and Sites (2003) found this species associated with the plant taxa *Brasneia* and *Lespedeza*.

##### Discussion.

Fitzgerald (1987) described the social system of *Dineutus nigrior* in detail and Fairn et al. (2008) studied the effects of parasitism by larval water mites of the genus *Eylais* Latreille, 1796 on adults of *Dineutus nigrior*. Evidence for sexual selection within this species has also been investigated (Fairn et al. 2007a; Fairn et al. 2007b). Brief life history information is available in Istock (1966; 1967).

Key to the larvae of *Dineutus nigrior* provided by Hatch (1927).

#### Dineutus productus

Taxon classificationAnimaliaColeopteraGyrinidae

Roberts, 1895

**Figure 33.**
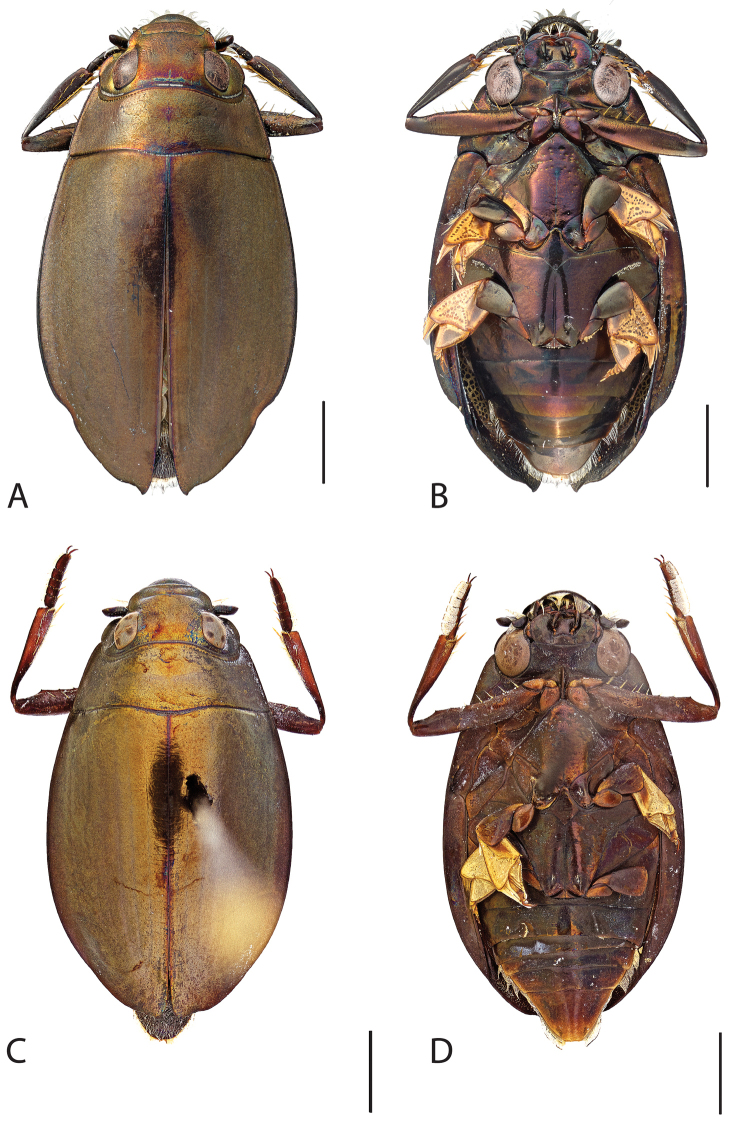
*Dineutus productus*. **A** ♀ dorsal habitus **B** ♀ ventral habitus **C** ♂ dorsal habitus. **D** ♂ ventral habitus All scale bars ≈ 2 mm.

**Figure 34.**
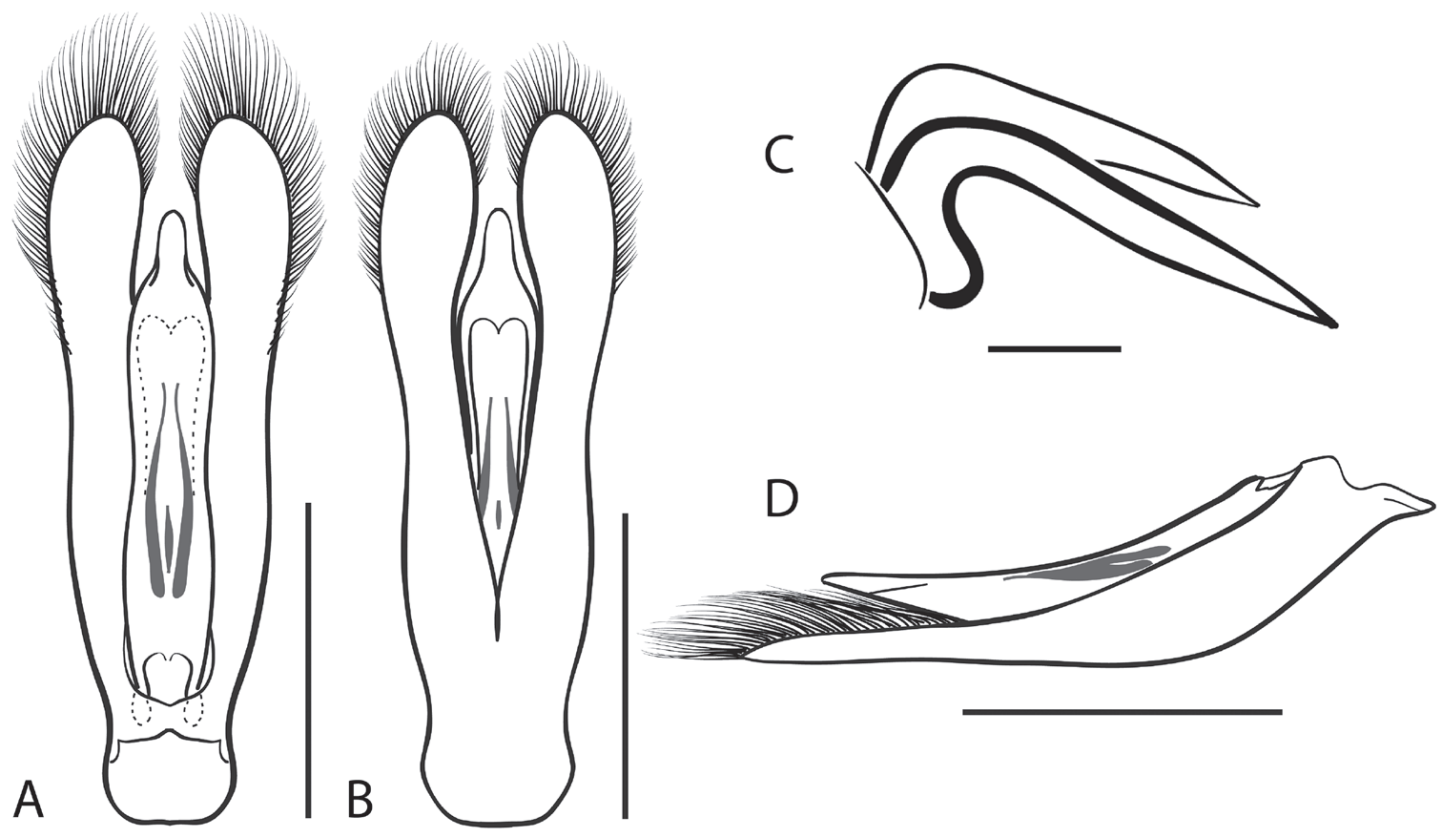
*Dineutus productus*. **A** aedeagus dorsal view **B** aedeagus ventral view **C** ♂ mesotarsal claws **D** aedeagus lateral view. Scale bar for **C** ≈ 0.10 mm all others ≈ 1 mm.

Figures 33 , 34 , 54

Dineutes productus Roberts 1895: 282, Dineutus (Cyclinus) productus: Ochs 1926a: 137.

##### Type locality.

USA, Texas.

##### Specimens examined.

17

##### Type material examined.

Syntype (♂ pinned) “Tex./Acc. 4858/Type productus, ♂type # 2, C.H.R./Dineutus productus Roberts 1895 Det: L. Cook 2005” AMNH type Catologue 496.

##### Material examined.

**U.S.A.:** **Kansas:** Bourbon Co., Ft. Scott, ca. 2 mi NE, Marmaton River, “T25S R25Esec 20 NW 1/4”, 4.viii.1976, leg. S. Hamilton, T. Oldham, SEMC 10549560 (1 ex. KSEM); Elk Co., Elk River at Elk Falls, 37.374416 -96.184123, 278m, 22.vi.2014, leg. C. Maier, C. Faris, S. Baca, G.Gustafson, GTG062214B (5 ex. GTGC), Elk River S. of Longton, 37.36960 -96.078735, 262m, 22.vi.2014, leg. C. Maier, C. Faris, S. Baca, G.Gustafson, GTG062214A (6 ex. GTGC); Greenwood Co., Lapland, 4mi S Fall River, east branch, 13.ix.1974, leg. D. Huggins, SEMC 1054959 (1 ex. KSEM); Lyon Co., Lyon Co.-Greenwood Co. line, Verdigiris River, “T21S R10E sec 36 SE 1/4”, 16.vii.1976, leg. S. Hamilton, SEMC 1054955, SEMC 1054957 (2 ex. KSEM); Morris Co., Council Grove Lake, outflows and groves, 13.vi.1974, leg. T. Edmonds, SEMC 1054958 (1 ex. KSEM); **Texas:** “Sequin”, 26.vi.1938, leg. D.W. Craik (2 ex. KSEM).

##### Diagnosis.

Male (Fig. 33C, D): Size: 9.5–9.6 mm. Body form narrowly elongate oval; elytral apices rounded with sutural angle produced to a point, apicolateral sinuation weakly present, irregularities present apically near sutural production, elytral apices strongly deflexed, reticulation of pronotal and elytral discs strongly impressed, producing a bronzy appearance; elytral striae faintly evident, most evident on the subapicomedial portion of the elytral disc; profemora with a very small sub-apicoventral tooth, accompanied by a series of denticles extending proximad, associated with each posterior setigerous puncture of the profemora; protibiae subsinuate; mesotarsal claws (Fig. 34C) long and of similar size, with ventral margin straight; venter darkly colored, usually black to very dark bronzy-brown, with the tibiae and tarsi of mesothoracic and metathoracic legs usually lighter in coloration, as well as apex of abdomen; Aedeagus (Fig. 34A, B, D) median lobe in dorsal view noticeably shorter than parameres, weakly constricted just apicad to middle, acuminate in apical ca. 1/5, apex of acumination flatly rounded, in lateral view median lobe roundly curved dorsally, in dorsal view parameres sinuate laterally after basal 1/3, in apical 1/3 laterally expanded, apically strongly rounded.

Female (Fig. 33A, B): Size: 9.5–9.9 mm. Body form narrowly elongate oval; elytral apices angled towards very strong sutural production, apicolateral sinuation strongly present and with plica, irregularities present apically near sutural production, elytral apices strongly deflexed, reticulation of pronotal and elytral discs strongly impressed, producing a bronzy appearance; elytral striae faintly evident, most evident on the subapicomedial portion of the elytral disc; profemora without sub-apicoventral tooth; protibiae club shaped, with lateral margin round, weakly expanded apicaly; venter darkly colored, usually black to very dark bronzy-brown, with the tibiae and tarsi of mesothoracic and metathoracic legs usually lighter in coloration, as well as apex of abdomen.

##### Differential diagnosis.

*Dineutus productus* can be distinguished from all other North American *Dineutus* in males having the profemora with a small sub-apicoventral tooth, accompanied by a series of denticles that extend proximad, with each denticle being associated with a posterior setigerous puncture of the profemora, elongate mesotarsal claws of equal size, with straight ventral margins (Fig. 34C), and in the form of the aedeagus (Fig. 34A). The species most similar to *Dineutus productus* is *Dineutus serrulatus analis*. The nominal subspecies of *Dineutus serrulatus serrulatus* should not be confused with *Dineutus productus* in that it has a restricted range in Florida, and its elytral apices would not easy be confused with those of *Dineutus productus*, being regularly rounded, with a strong apicolateral sinuation in both sexes and the dorsum being polished black in appearance. *Dineutus serrulatus analis* on the other hand tends to be variable in the appearance of the elytra (Fig. 37A, C), with some members having the sutural angle produced and the dorsum with strong microreticulation giving the elytra and pronotum a polished bronzed appearance, similar in both respects to *Dineutus productus*.

*Dineutus productus* can generally be separated from *Dineutus serrulatus analis* in having a more elongate narrow body form (Fig. 33A, C), and being smaller in size. *Dineutus productus* is much more narrowly attenuated anteriorly, compared to *Dineutus serrulatus analis*, which is broader in body form and larger in size. The ventral coloration of *Dineutus productus* is most often black to very dark brownish red, and frequently accompanied by a bronzy metallic sheen, while the venter of *Dineutus serrulatus analis* is most often red in color, rarely appearing black and often lacking any metallic sheen. When more darkly colored *Dineutus serrulatus analis* is much more red in hue, compared to when *Dineutus productus* is more lightly colored, having a more brownish hue. A few specimens of *Dineutus serrulatus analis* examined from southeast Kansas had their venters truly black (KSEM) and for these specimens differences between the male secondary sexual characters as well as differences in the elytra allowed for unambiguous identification. The serration of the elytral apices differs between these two species. In *Dineutus productus* the apices of the elytra only have irregularities present, at most having round bumps that resemble highly reduced serration, while in *Dineutus serrulatus analis* the serration is present as distinct and fine points.

The males of *Dineutus productus* can easily be distinguished from those of *Dineutus serrulatus analis* in that the profemoral sub-apicoventral tooth is small and accompanied by a series of denticles that extend proximad, with each denticle being associated with a posterior setigerous puncture of the profemora, in *Dineutus serrulatus analis* there is a profemoral sub-apicoventral tooth as normal, but no accompanying series of denticles. This character appears unique to *Dineutus productus* among all of the North American *Dineutus* species. The mesotarsal claws also differ greatly between these two species. *Dineutus productus* males have the mesotarsal claws elongate with their ventral margins straight (Fig. 34C) and lacking a denticle, while in *Dineutus serrulatus analis* the mesotarsal claws are small, with their ventral margins curved and possessing a denticle (38F). The aedeagus is by far the most definite way to separate the two species. The aedeagus of *Dineutus productus* (Fig. 34A) has the median lobe shorter than the parameres, and strongly acuminate apically, with the parameres being sinuate after the basal 1/3 and laterally expanded in the apical 1/3, with the apices rounded. In *Dineutus serrulatus analis* the median lobe (Fig. 38A, D, H) is as long as the parameres, parallel sided for most its length, having only the apical 1/4 narrowed, with the parameres being parallel sided for most of their length, being only weakly expanded in the apical 1/3 and often obliquely truncate, or obliquely flatly rounded.

Females of *Dineutus productus* (Fig. 33A) can be distinguished from those of *Dineutus serrulatus analis* in having a much greater apicolateral sinuation of the elytra, the sinuation is so deep that a plica is formed, and the elytral apices have a much steeper angle towards the sutural production than in *Dineutus serrulatus analis* females (Fig. 37A), which have a much more shallow apicolateral sinuation and more rounded elytral apices. As mentioned earlier the elytra of *Dineutus productus* have irregularities present apically, but not fine serration, as seen in *Dineutus serrulatus analis*.

##### Distribution

**(Fig. 54A).** Central and south-central United States (Roberts 1895), into northeastern Mexico (Ochs 1949). As this species is so rarely collected, we list here the states and counties where it has been collected and the identification is not in question: **USA:** **Kansas:** Bourbon Co., Elk Co, Greenwood Co., Lyon Co., Morris Co. (this study); **Texas:** Bosque Co. [Clifton] (Ochs 1929), Brazos Co. [College Station] (Wood 1962), Comal Co. (Wood 1962), Dallas Co. [Dallas County](Ochs 1929), Dimmit Co. (Wood 1962), Karnes Co. [Kenedy](Wood 1962), Neuces Co. [Corpus Christi](Wood 1962); **Mexico:** Nuevo Leon: Granja Rodriguez on the borders of the Rio Salado alt. 195m (Ochs 1949).

##### Habitat.

Lotic species, the Kansas Biological Survey field notes (available at the KSEM) indicate the species was collected from slow moving, cool, mud bottomed streams, that were fairly wide. Gustafson et al. (2014) describe the habitat where *Dineutus productus* was recently collected in detail, accompanied by photos of the habitats. The species has also been taken at light traps placed at water level in the vicinity of rapids, on the borders of the Rio Salado, in Nuevo Leon, Mexico (Ochs 1949).

##### Discussion.

*Dineutus productus* appears to be an uncommonly collected species and the reason for this is not exactly clear. Most species found in the United States are uncommon due to a narrow range of endemism (e.g. *Dineutus robertsi* and *Dineutus angustus*), but material examined in this study, as well as records from the literature, suggest the range of this species is not as narrow as the previously mentioned species. *Dineutus productus* was originally described from Texas by Roberts (1895), and most records of this species come from this state (Régimbart 1907; Ochs 1929; Wood 1962). Young (1954) claimed to have specimens from the panhandle of Florida, but Epler (2010) re-examined specimens in the FSCA identified by Young as *Dineutus productus*, finding them to belong to other species. Hatch (1925a) claimed to have collected the species from the Sangamon River near Decatur, Illinois. Ochs (1949) however, questioned the Illinois record as well as Régimbart’s (1907) from the Carolinas, stating all specimens identified as *Dineutus productus* from outside of Texas actually belonged to other species upon his examination. In the same paper Ochs (1949) extended the species range southward in to Neuvo Leon, Mexico.

Material examined for this study from Kansas (KSEM, GTGC), was undoubtedly *Dineutus productus*, confirming that the range of *Dineutus productus* extends at least that far northward. This adds credence to Hatch’s (1925a) record from Illinois. Furthermore, comparing images of the Sangamon River available online to those provided by Gustafson et al. (2014), they appear to be the same habitat. The first author was unable to locate Régimbart’s specimen of *Dineutus productus* within the MNHN, in order to confirm Régimbart’s 1907 record, but *Dineutus productus* was not listed as a species of *Dineutus* found in North and South Carolina by Sanderson (1982). Given this and the inability to locate the specimen of Régimbart’s from the Carolinas in his collection at the MNHN, we do not treat Régimbart’s 1907 record as a part of the species range. Ochs’ diagnosis of *Dineutus productus* (1949) matchs our own, therefore we accept his record for the species from Nuevo Leon, Mexico as representing the southern-most extreme for the range. As mentioned above, given the new Kansas records for the species and the habitat photos of the Sangamon River available online, we accept Hatch’s (1925a) record from Illinois, as the northern-most extreme for the species’ range.

Most works treating *Dineutus productus* only mentioned having examined a few specimens (i.e. small series at most, usually 4 specimens for most studies [Roberts 1895; Régimbart 1907; Ochs 1929, 1949; Young 1954]). Recent collecting revealed that the species could be collected, albeit with much difficulty, which may explain this species rarity in collections (Gustafson et al. 2014). Hopefully accurate information on indentifying this species, as well as modern information of habitat and distribution, will help this species become better known.

#### Dineutus robertsi

Taxon classificationAnimaliaColeopteraGyrinidae

Leng, 1911

**Figure 35.**
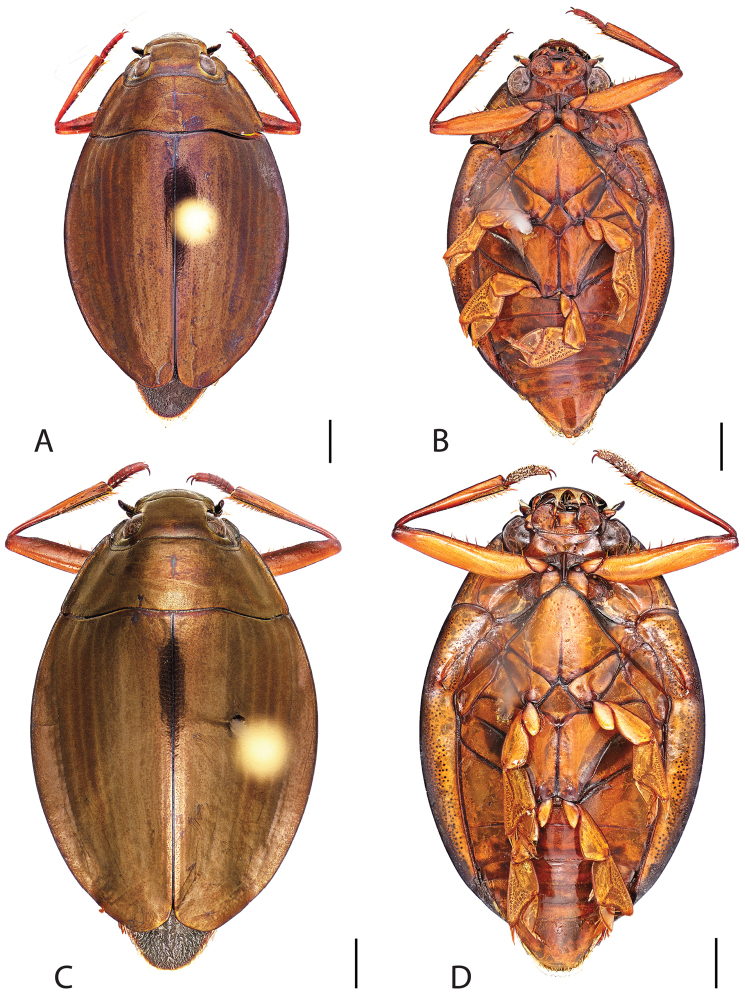
*Dineutus robertsi*. **A** ♀ dorsal habitus **B** ♀ ventral habitus **C** ♂ dorsal habitus **D** ♂ ventral habitus. All scale bars ≈ 2 mm.

**Figure 36.**
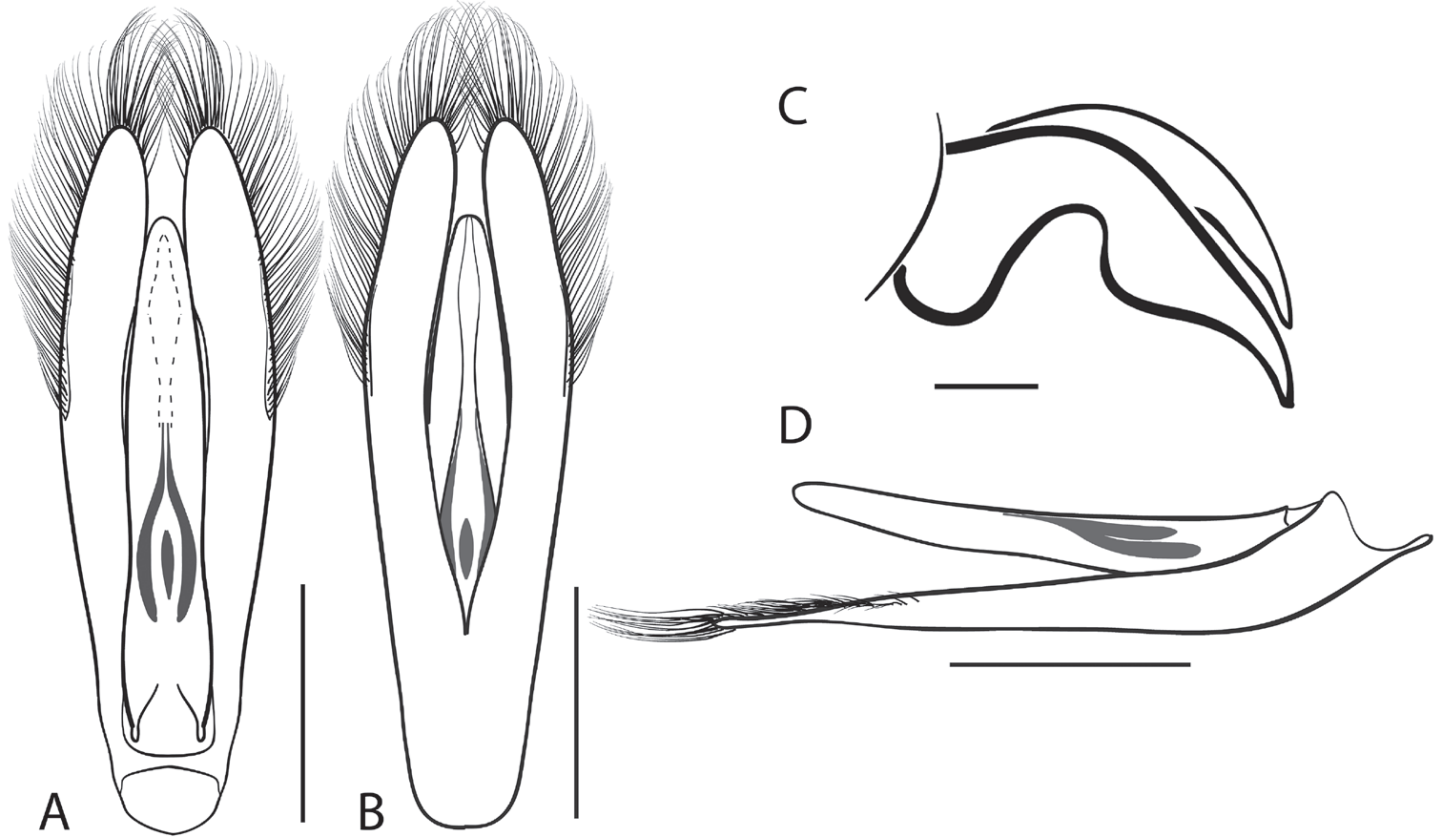
*Dineutus robertsi*. **A** aedeagus dorsal view **B** aedeagus ventral view **C** ♂ mesotarsal claws **D** aedeagus lateral view. Scale bar for **C** ≈ 0.10 mm all others ≈ 1 mm.

Figures 35 , 36 , 53

Dineutes robertsi Leng 1911: 11, Dineutus (Dineutus) robertsi: Ochs 1926: 138, *Dineutus robertsi*: Leech 1938: 61, Dineutus (Protodineutus) robertsi: Guignot 1950: 126, Dineutus (Cyclinus) robertsi: Brinck 1955: 106, *Dineutus robertsi*: Sanderson 1982: 10.30.

##### Type locality.

U.S.A., Georgia: “West Branch War Woman Creek, Rabun Co., Ga., in the mountains, at an elevation of about 2,000 feet.”.

##### Specimens examined.

59

##### Type material examined.

Not examined, but specimens collected from the type locality.

##### Material examined.

**U.S.A.:** **Georgia:** Clayton Co., Warwoman Wldf Mgmt A, Tuckaluge Cr., 34.90155°N 83.30015°W, 533 m, 11.vii.2012, leg. K.B. Miller, KBM11071201 (31 ex. MSBA); Warwoman Cr., 34.89843°N 83.27512°W, 11.vii.2012, leg. K.B. Miller, KBM11071202 (8 ex. MSBA); Cleveland Co., Chattahoochee R., 34.72111°N, 83.74807°W, 12.vii.2012, leg. K.B. Miller, KBM12071202 (11 ex. MSBA); **South Carolina:** Moutain Rest Co., Sumter Ntl. For., 34.85292°N 83.14336°W, 12.vii.2012, leg. K.B. Miller, KBM11071203 (9 ex. MSBA).

##### Diagnosis.

Male (Fig. 35C–D): Size: 12.1–15.5 mm. Body form elongate broadly oval; antennal flagellum narrow and parallel sided, ultimate segment elongate and pointed apically; elytral apices regularly rounded, serration absent, elytra entirely bronzy, elytral striae faint but fairly distinct, 8^th^ elytral stria with large shallow punctures present; profemora without sub-apicoventral tooth; protibiae club-shaped; anterior mesotarsal claw (Fig. 36C) with strong denticle; venter lightly colored yellow to light orange in coloration; Aedeagus (Fig. 36A, B, D) medial lobe in dorsal view flatly rounded apically, in ventral view sperm-groove triangular, narrowed posteriorly and anteriorly, in lateral view median lobe thick and flat, parameres strongly arced in apical 1/3.

Female (Fig. 35A–B): Size: 13.6–15.1 mm. Body form elongate broadly oval; antennal club narrow and parallel sided, ultimate segment elongate and pointed apically; elytral apices regularly rounded, serration absent, elytra entirely bronzy, elytral striae faint but fairly distinct, 8^th^ elytral stria with punctures present; profemora without sub-apicoventral tooth; protibiae club-shaped; venter pale yellow in coloration.

##### Differential diagnosis.

This species is easily distinguished from all other North American species of *Dineutus* by its large size, profemora lacking a sub-apicoventral tooth, entirely bronzy elytra, and broadly oval body shape with a pale yellow venter. Other North American species with pale venters are much more attenuated in body shape (e.g. *Dineutus discolor*, *Dineutus longimanus*).

The species most similar to *Dineutus robertsi* is *Dineutus ciliatus*. *Dineutus robertsi* differs from *Dineutus ciliatus* in having the antennal club narrow and parallel sided, with the ultimate segment elongate and pointed apically, as opposed to having the flagellum being thicker and rounder with the ultimate segment rounded. *Dineutus robertsi* also has the 8^th^ elytral stria with punctures evident laterally as opposed to having them absent or indistinct as in *Dineutus ciliatus*. The males of *Dineutus robertsi* can further be distinguished from *Dineutus ciliatus* in having the anterior mesotarsal claw (Fig. 36C) with a strong denticle. The aedeagus of *Dineutus robertsi* (Fig. 36A) has the median lobe in dorsal view flatly rounded apically with the apex lacking an apicomedial papilla, as compared to *Dineutus ciliatus* (Fig. 13A) in which an apicomedial papilla is present. In ventral view (Fig. 36B), the sperm-groove of the median lob is triangular, narrowed posteriorly and anteriorly, as opposed to that of *Dineutus ciliatus* (13B) which is broader and more parallel sided. In lateral view the median lobe of *Dineutus robertsi* (Fig. 36D) is thick and flat, not dorsally curved as is the case in *Dineutus ciliatus* (Fig. 13D). The parameres also differ in being strongly arced in apical 1/3, those of *Dineutus ciliatus* are weakly arced.

##### Distribution

**(Fig. 53B).** Known from the Appalachian mountains of northeastern Georgia, and southwestern North and South Carolina in the United States (Leng 1911; Sanderson 1982; Wood 1962).

##### Habitat.

Lotic species, frequenting mountainous streams with rocky bottoms. The second author collected many specimens from moderately high gradient, rocky streams in higher elevation mountainous regions in the southern Appalachians. Specimens were common in pools and margins of the streams. They were not found at lower elevations or on larger rivers where they were replaced by *Dineutus discolor* and *Dineutus ciliatus*.

##### Discussion.

*Dineutus robertsi* is highly endemic to the Appalachians of northeastern Georgia and southwestertn North and South Carolina (Fig. 53B). For this reason *Dineutus robertsi* has been infrequently collected and is poorly represented in collections. Once in the range of this species however, it can be regularly collected on mountain streams, and in large numbers. The full extent of the range of *Dineutus robertsi* is still in question. Further sampling of the Appalachians would be greatly beneficial for determining the extent of the range of this highly endemic species.

#### Dineutus serrulatus

Taxon classificationAnimaliaColeopteraGyrinidae

LeConte, 1868

##### Differential diagnosis.

Overall this species can be diagnosed in having elytral apices that are serrulate and with apicolateral sinuation present in both sexes, red colored venters, males with a profemoral sub-apicoventral tooth, and in the form of the male aedeagus. A key to the subspecies is provided and each subspecies is treated individually.

##### Distribution and subspecies.

This species is wide-ranging, highly variable among populations (Fig. 39), and has been divided into two subspecies: *Dineutus serrulatus serrulatus* and *Dineutus serrulatus analis* (Wood 1968). Of these subspecies *Dineutus serrulatus serrulatus* is more consistent in its characters, while those of *Dineutus serrulatus analis* are much more variable causing difficulties in separating the two. *Dineutus serrulatus analis* was originally described as a separate species by Régimbart in 1882, but was relegated to a subspecies of *Dineutus serrulatus* LeConte by Wood (1968). Recent papers have again treated *Dineutes analis* as a separate valid species (e.g. Ciegler et al 2003; Realzola et al. 2007). For this study the authors found no single discrete character that could reliably separate the taxa. Although dorsal coloration can usually be used to separate the two subspecies, populations from northern Florida where the two subspecies meet, show intermediate dorsal coloration, being medially polished black, but laterally bronzy green. Furthermore the aedeagi, of the two subspecies are very similar, with only minor differences that appear variable across the entire range of this species. Another useful character for delimiting similar species, the mesotarsal claws, are also similar showing only minor variation. For these reasons we continue to follow Wood (1968) in treating these two taxa as subspecies.

##### Key to the subspecies of *Dineutus serrulatus*

**Table:** 

1	Body form more broadly oval, especially evident in males (Fig. 40C); male elytra with apices often flatly rounded/subtruncate, rarely with sutural angle produced to a point, apicolateral sinuation strongly present; female elytral apices flatly rounded and commonly with sutural angle produced, apicolateral sinuation less strongly present; dorsal surface with fine reticulation giving the dorsum a more polished smooth appearance, often very darkly colored, black to dark greenish black; profemoral sub-apicoventral tooth of male large and highly acute (Fig. 40D); venter always red in coloration. Distribution: Alabama, Georgia, Florida, and the Carolinas	***Dineutus serrulatus serrulatus***
–	Body form more parallel sided, less broadly oval, even in males (Fig. 40C); males with elytral apices variable from flatly rounded/subtruncate, but often with sutural angle produced, apicolateral sinuation present, but less deeply sinuate; female elytral apices flatly rounded to angled towards sutural production; sutural production commonly present, rarely absent; dorsal surface with reticulation strongly evident and often coarser than nominate form, giving dorsal surface a bronzy appearance, not polished black, often light bronzed to greenish bronzed in color; profemoral sub-apicoventral tooth often smaller than nominate form (Fig. 40D), and more variable; venter often light colored usually red, but northern and western populations with darkly colored venter, dark red to blackish red in coloration. Distribution: From western Alabama north to Indiana, west to southwestern Kansas, south to Texas and possibly Mexico	***Dineutus serrulatus analis***

#### Dineutus serrulatus analis

Taxon classificationAnimaliaColeopteraGyrinidae

Régimbart, 1883

**Figure 37.**
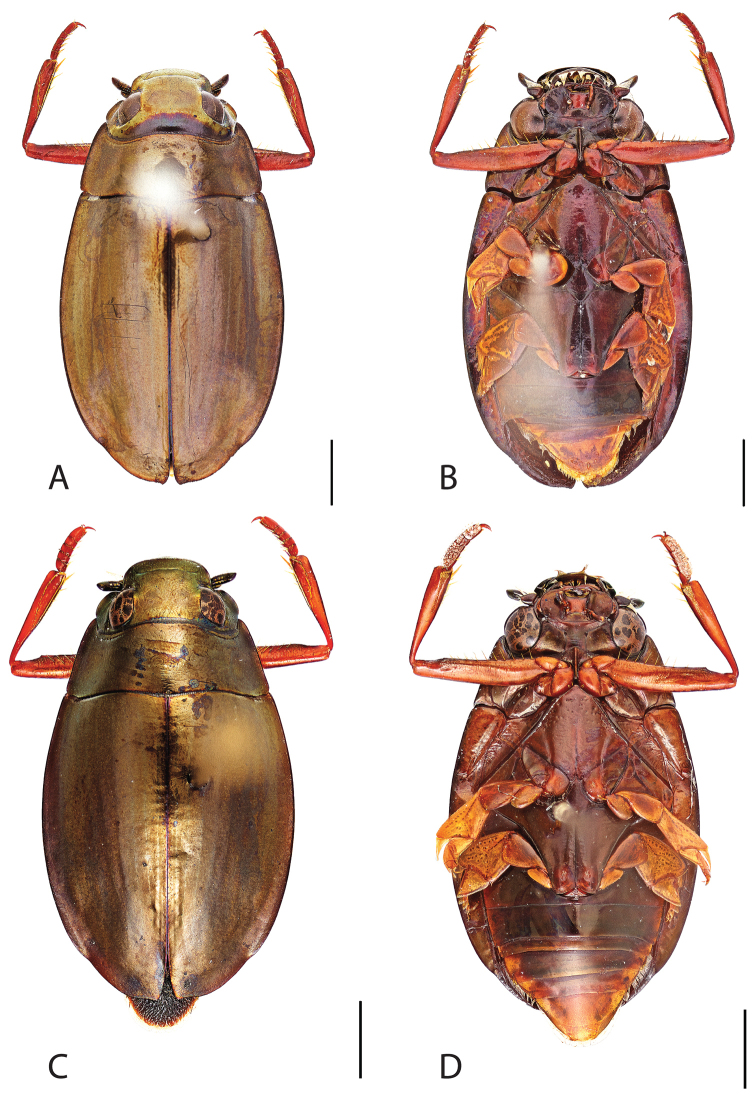
*Dineutus serrulatus analis*. **A** ♀ dorsal habitus **B** ♀ ventral habitus **C** ♂ dorsal habitus **D** ♂ ventral habitus. All scale bars ≈ 2 mm.

**Figure 38.**
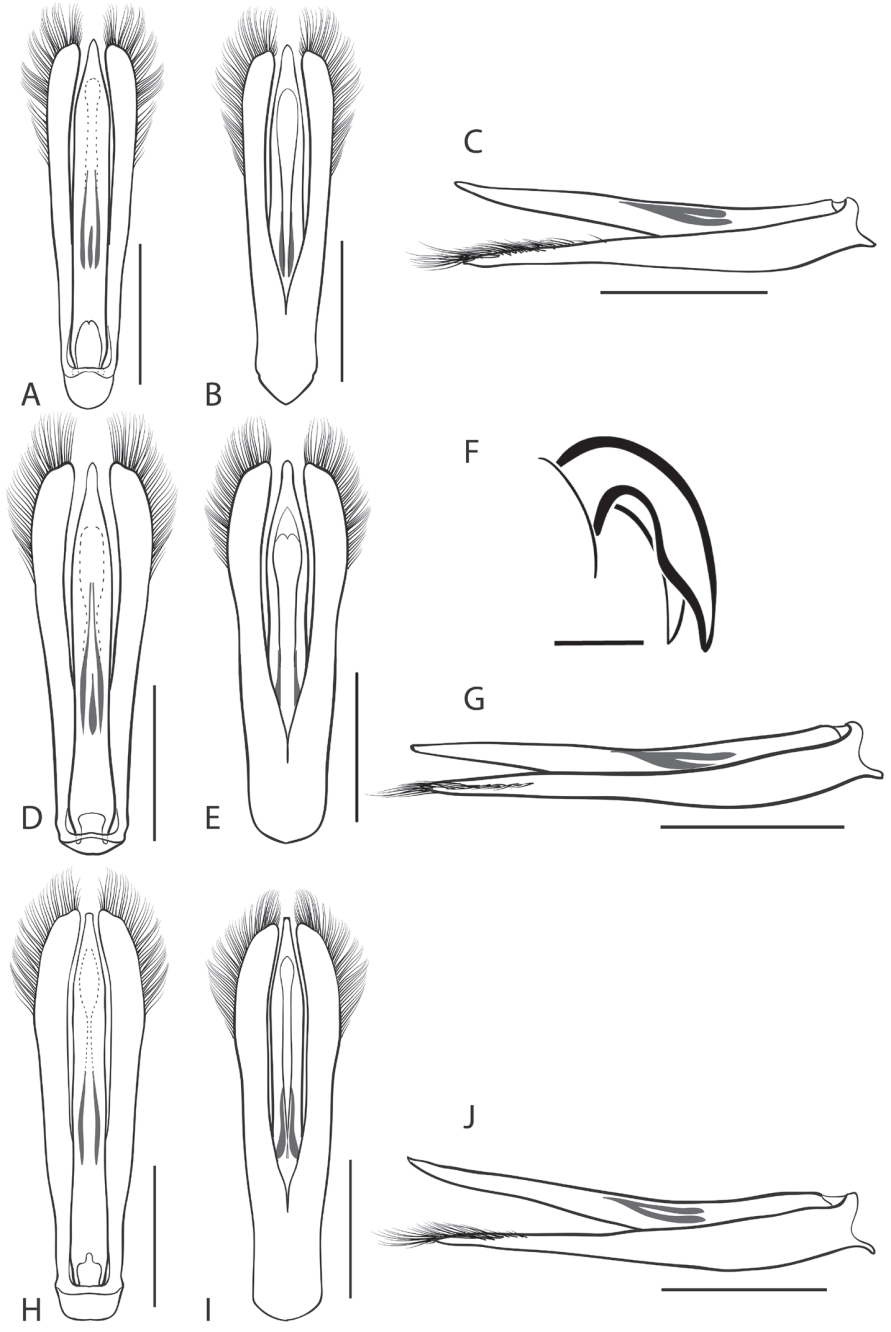
*Dineutus serrulatus analis*. Liberty Co. Florida specimen aedeagus **A** dorsal view **B** ventral view **C** lateral view; Tennessee specimen aedeagus **D** dorsal view **E** ventral view **F** ♂ mesotarsal claws **G** aedeagus lateral view; Kansas specimen aedeagus **H** dorsal view **I** ventral view **J** lateral view. Scale bar for **F** ≈ 0.10 mm all others ≈ 1 mm.

**Figure 39.**
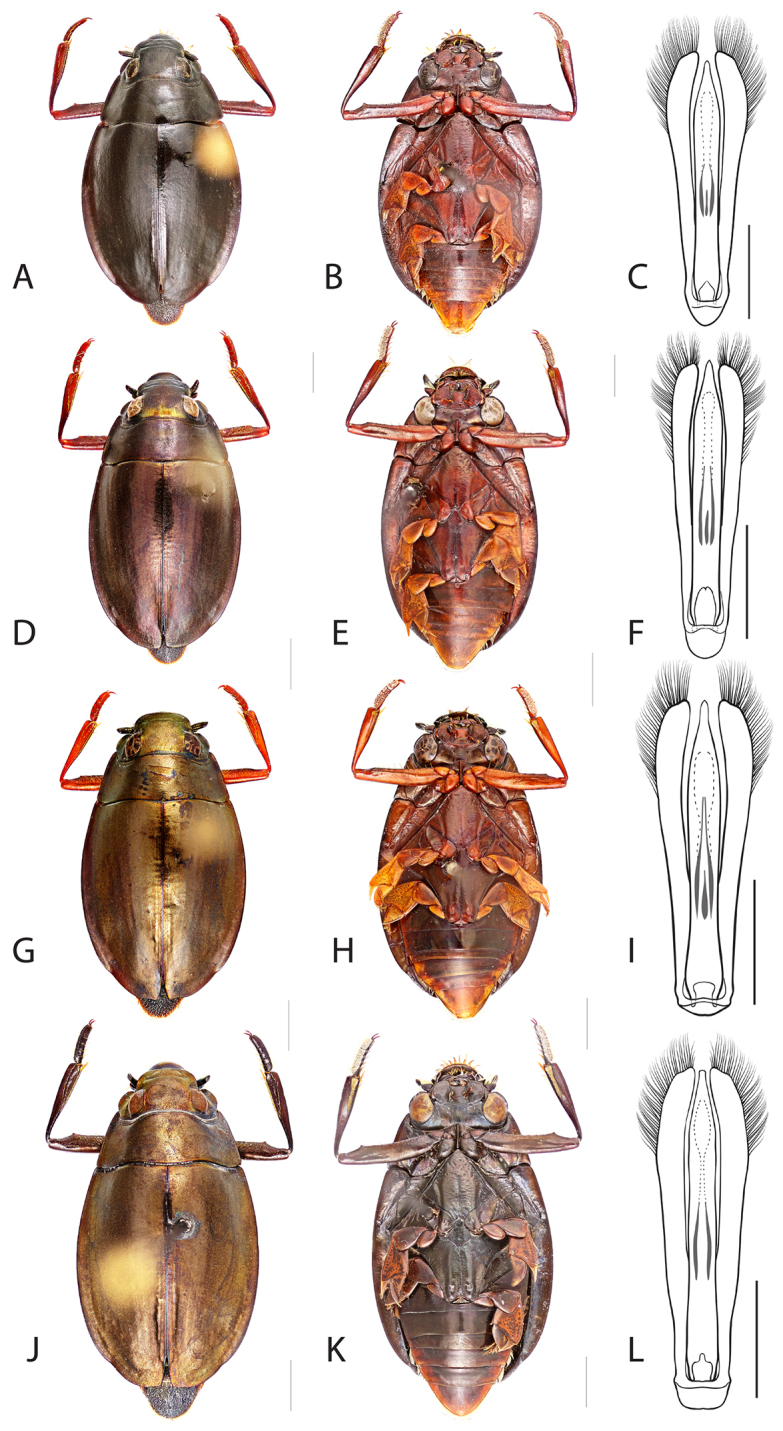
*Dineutus serrulatus* variation in males. *Dineutus serrulatus serrulatus* **A** dorsal habitus **B** ventral habitus **C** aedeagus dorsal view; *Dineutus serrulatus analis* Liberty Co. Florida **D** dorsal habitus **E** ventral habitus **F** aedeagus dorsal view; *Dineutus serrulatus analis* Tennessee **G** dorsal habitus **H** ventral habitus **I** aedeagus dorsal view; *Dineutus serrulatus analis* Kansas **J** dorsal habitus **K** ventral habitus **L** aedeagus. Scale bars for **C, F, I, L** ≈ 1 mm all others ≈ 2 mm.

Figures 37 , 38 , 39 , 51 , 53

Dineutes analis Régimbart 1883: 416, Dineutus (Cyclinus) analis: Ochs 1926: 1377, *Dineutus serrulatus analis*: Wood 1968: 4, *Dineutus analis*: Ciegler et al. 2003: 14.

##### Type locality.

U.S.A., Texas

##### Specimens examined.

41

##### Type material examined.

Lectotype, here designated (1 ♂ pinned, missing right arm) “Texas [white label, handwritten in black ink, handwriting unknown]// MUSEUM PARIS/ COLL MAURICE REGIMBART/ 1908 [blue label with thin black border, typed black ink]// LECTOTYPUS/ P. Brinck designavit 1955. [white label, typed black ink]// TYPE [red label, typed black ink]// LECTOTYPE [red label, typed black ink]//” deposited in the MNHN. Paralectotype (1 ♂ pinned, missing right mesothoracic leg) “Louisiana [white label, typed black ink]// MUSEUM PARIS/ COLL MAURICE REGIMBART/ 1908 [blue label with thin black border, typed black ink]// PARALECTOTYPE [red label, typed black ink]//” (1 ex. MNHN).

##### Material examined.

**U.S.A.:** **Arkansas:** Washington Co., Lake Sequoyah, 7.x.1992, leg. S. Garner (1 ex. MTEC); **Florida:** Bradford Co., 3.ii.1949, leg. B.W. Cooper (1 ex. FSCA); Highlands Co., Highlands Hammock State. Prk., 15.iii.1974, leg. R.E. Beer (1 ex. KSEM); Liberty Co., Yellow Creek SE of Telogia, 7.x.1992, leg. F.N. Young, #3503 (12 ex. FSCA); Suwannee Co., Branford, 31.vii.1930, leg. P.W. Oman (1 ex. KSEM); **Kansas:** Elk Co., Longton, 1 mi S, 1 mi E, Elk River, “T31S”, 2.viii.1977, leg. S. Hamilton, SEMC 1057036 (1 ex. KSEM); Labette Co., Altamont, 5 mi E, Labette Creek, 22.vi.1974, SEMC 1056921, 1056919 (2 ex. KSEM); Montgomery Co., Caney, 25.vi.1991, leg. D. Miller, (1 ex. MTEC); Montgomery Co., Havana, ca. 2 mi N, Coon Creek, 24.vii.1974, leg. T. Edmonds, SEMC 1057032 (1 ex. KSEM); Montgomery Co., Drum Creek, US-160, 23.vii1974, leg. T. Edmonds, SEMC 1057034 (1 ex. KSEM) Sedwick Co., Goddard, 2.5 mi N, 5 mi E, creek, 17.vii.1975, leg. S. Matthies, SEMC 1057030 (1 ex. KSEM); Wilson Co., Altoon, 3.4 mi S, 3.5 mi E Chetopa Creek, “T29S R17E sec 31 NW 1/4”, 5.viii.1977, leg. S. Hamilton, T. Oldham, SEMC 1057019-1057020 (2 ex. KSEM); Wilson Co., Roper, 0.25 mi W, Buffalo Creek, “T27S R15E sec 35, 5.viii.1977, leg. S. Hamilton, T. Oldham, SEMC 1056998 (1 ex. KSEM); **Mississippi:** Hancock Co., Devil’s Swamp, 7.v.1965, leg. H.R. Hepburn (3 ex. KSEM); Hancock Co., Asley, 8.iii.1966, leg. H.R. Hepburn, (1 ex. KSEM); **Tennessee:** McNairy Co., 8 mi S.W. Ramer, 6.viii.1975 (4 ex. FSCA); **Texas:** Montgomery Co., The Woodlands, 10-18.vi.1977, leg. J.E. Wappes (1 ex. FSCA); Victoria Co., Victoria, Musang Creek, 8.ii.1932, leg. L.D. Tuthill (4 ex. KSEM).

##### Diagnosis.

Male (Fig. 37C–D): Size: 9.9–11.4 mm. Body form elongate oval; elytral apices flatly rounded, often with sutural angle produced to a point, apicolateral sinuation present, with sinuation shallow, serration and irregularities present apically near sutural production, elytral apices weakly deflexed, reticulation of pronotal and elytral discs strongly impressed, producing a bronzy appearance; elytral striae faintly evident, most evident on the medial portion of the elytral disc; profemora with sub-apicoventral tooth; protibiae subsinuate; mesotarsal claws (Fig. 38F) small and of similar size, with ventral margin with a more or less developed smooth denticle; venter lightly or darkly colored, coloration usually red but ranging to very dark red or black, with the mesothoracic and metathoracic legs usually lighter in coloration, as well as apex of abdomen; Aedeagus (Fig. 38A–C, D, E, G, H–J) with median lobe in dorsal view as long as parameres, highly parallel sided, narrowed in apical 1/4, apex flatly to regularly rounded, in lateral view median lobe weakly curved dorsally after basal 1/3, parameres in apical 1/3 weakly laterally expanded, apically obliquely flatly rounded to truncate.

Female (Fig. 37A–B): Size: 10.0–11.4 mm. Body form elongate oval; elytral apices flatly rounded, sutural angle often produced to a point, apicolateral sinuation shallow, serration present apically near sutural production, elytral apices weakly deflexed, reticulation of pronotal and elytral discs strongly impressed, producing a bronzy appearance; elytral striae faintly evident, most evident on the medial portion of the elytral disc; profemora without sub-apicoventral tooth; protibiae club shaped, with lateral margin flatly round; venter lightly or darkly colored, coloration usually red but ranging to very dark red or black, with the mesothoracic and metathoracic legs usually lighter in coloration, as well as apex of abdomen.

##### Differential diagnosis.

*Dineutus serrulatus analis* is unique among all other North American *Dineutus* species in belong elongate oval and attenuated anteriorly, with elytral apices having apicolateral sinuations in both sexes, flatly rounded elytral apices, often with the sutural angle produced to a point, and with serration present, males with the profemora with a sub-apicoventral tooth, and in the form of the male aedeagus. The species most similar to *Dineutus serrulatus analis* is *Dineutus productus*. *Dineutus serrulatus analis* can be separated from *Dineutus productus* by the differential diagnosis given for *Dineutus productus*.

Distinguishing between *Dineutus serrulatus analis* and *Dineutus serrulatus serrulatus* can primarily be done using the differences in the key listed above.

##### Distribution

**(Fig. 53C).** Southeastern United States (Ciegler et al. 2003; Epler 2010; Régimbart 1882; Roberts 1895; Wood 1962; 1968).

##### Habitat.

Lotic species found in small streams usually below 500 feet in elevation (Wood 1968). For a more in depth description of habitat see (Gustafson et al. 2014).

##### Discussion.

Of the two subspecies *Dineutus serrulatus analis* has the wider of the two ranges (Wood 1968). *Dineutus serrulatus serrulatus* is primarily distributed in Florida and to the east in the Carolinas (Young 1954; Sanderson 1982). In northern Florida where the two subspecies meet specimens show intermediate morphology. Specimens examined in this study from Liberty County, Florida, (Fig. 39D [FSCA]) showed intermediate dorsal coloration being polished black medially, but laterally bronzy green and the sperm-groove of the aedeagus (Fig. 38B) is intermediate between the narrowed sperm-groove of northern populations of *Dineutus serrulatus analis* and the more broad sperm-groove of *Dineutus serrulatus serrulatus* from southern Florida. These specimens from Liberty County (FSCA) also show unique variation in the parameres (Fig. 38A), in that they are much more narrow and parallel sided in their apical half, not exhibiting the lateral expansions and arc seen in the parameres of other populations. However, the median lobe is identical in shape to that of *Dineutus serrulatus serrulatus*. While this variation in the past may have been enough to qualify as sub-specific differences, given how highly variable this species is (Fig. 39), no formal name will be applied at this time. Making the situation even more difficult, *Dineutus serrulatus analis* from Texas (Fig. 38D, E [KSEM]) have the aedeagus and sperm-groove identical to those of *Dineutus serrulatus serrulatus* (Fig. 41A, B). Specimens from southeastern Kansas (KSEM) were notable for having a bronzy dorsal surface, but a very dark reddish brown venter (Fig. 39J, K). The profemoral subapicoventral tooth is also quite large for a member of *Dineutus serrulatus analis*. Future genetic work may shed better light on the significance of this variation.

##### Type designation.

Régimbart (1882) in the original description describes the species from both Louisiana and Texas. In the MNHN there are two specimens from the Régimbart Collection, one from Texas and one from Louisiana. Here we formerly designate the specimen from Texas (Fig. 51A) as the lectotype and the specimen from Louisiana as the paralectotype.

#### Dineutus serrulatus serrulatus

Taxon classificationAnimaliaColeopteraGyrinidae

LeConte, 1868

**Figure 40.**
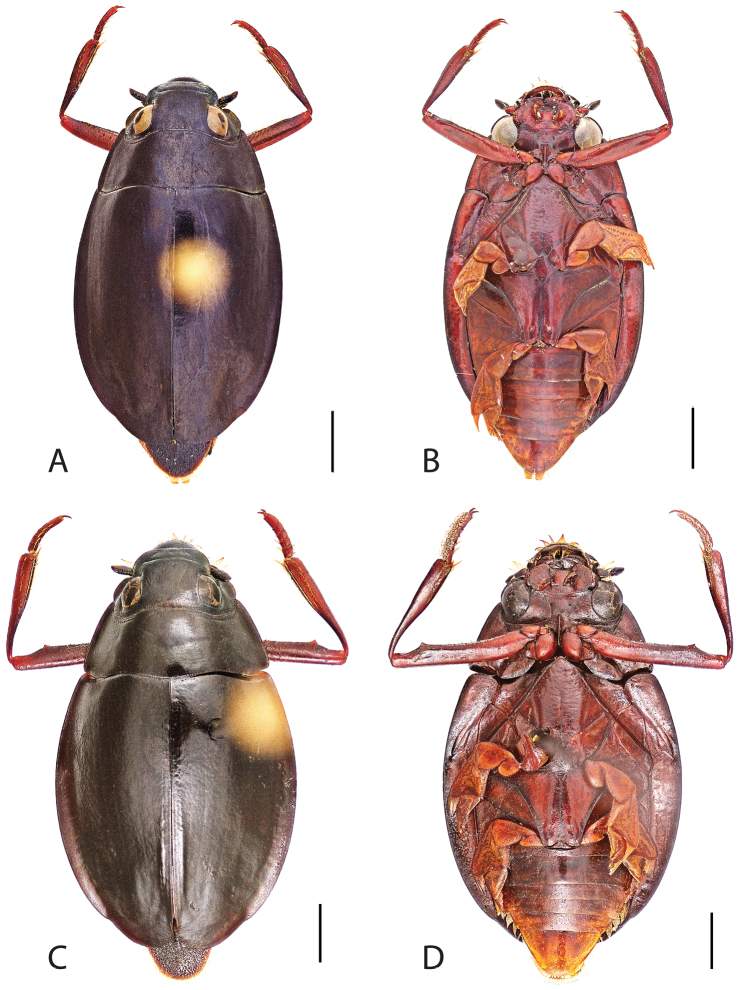
*Dineutus serrulatus serrulatus*. **A** ♀ dorsal habitus **B** ♀ ventral habitus **C** ♂ dorsal habitus **D** ♂ ventral habitus. All scale bars ≈ 2 mm.

**Figure 41.**
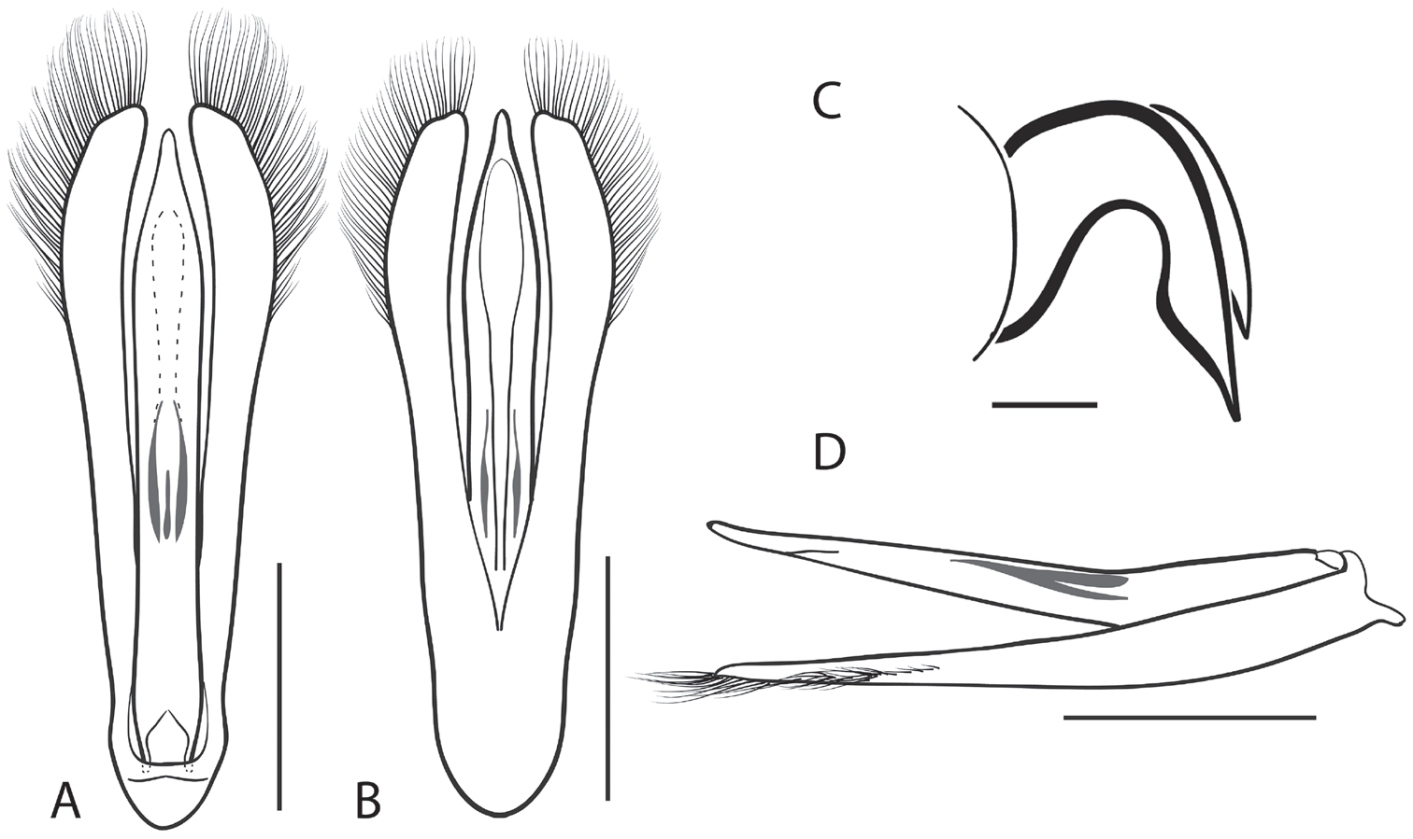
*Dineutus serrulatus serrulatus*. **A** aedeagus dorsal view **B** aedeagus ventral view **C** ♂ mesotarsal claws **D** aedeagus lateral view. Scale bar for **C** ≈ 0.10 mm all others ≈ 1 mm.

Figures 39 , 40 , 41 , 53

Dineutus serrulatus LeConte 1868: 366, Dineutus (Cyclinus) serrulatus: Ochs 1926a: 137. *Dineutus serrulatus serrulatus*: Wood 1968: 4, *Dineutus serrulatus*: Ciegler et al. 2003: 16.

##### Type locality.

U.S.A., “middle states” (which according to MCZ type database could be Maryland, Delaware, New York, New Jersey, Pennsylvania, Connecticut, Rhode Island), but according to Wood (1968) this subspecies is found only in Alabama, Florida, and Georgia. See distribution for more information.

##### Specimens examined.

117

##### Type material examined.

Lectotype (designated by Wood 1968: 3) (♂ pinned) “[pink disc]// Type/ 6094 [red label, Type in typed black ink, 6094 handwritten black ink]// Dineutus serrulatus Lec. [white label, hand written, handwriting appears to be LeConte’s]//” deposited in MCZ.

##### Material examined.

**U.S.A.:** **Florida:** Alachua Co., 10.ii.1949, leg. S.B. Mansell (1 ex. FSCA); same as previous except: 29.iv.1950, leg. T.G. Stewart (1 ex. FSCA); Hatchet Creek, 25.v.1985, leg. D.W. Johnson, water (1 ex. FSCA); Hogtown Creek, 28.vi.1976, leg. J.B. Heppner (6 ex. FSCA); Gainesville, 14.iii.1963, leg. R.E. Woodruff, in blacklight trap (1 ex. FSCA); Bradford Co., 3.ii.1949, leg. B.W. Cooper, (2 ex. FSCA); same as previous except: 16.vii.1934, leg. J.D. Beamer (1 ex. KSEM); Columbia Co., O’Leno State Park,12.ii.1966, leg. F.W. Mead (17 ex. FSCA); same as previous except: 11.xii.1954, leg. C.N. Patton (1 ex. FSCA); Dade Co., iii.1954, leg. L.N. Bell, (1 ex. FSCA); Cutler, 26.xi.1960, leg. D.R. Paulson, (3 ex. FSCA); Canal at Pinecrest, 29.xii.1982, leg. F.N. Young, #2977 (1 ex. FSCA); Hernando Co., Weekiwachee Spring, “Sta.4”, 3.vi.1953, leg. W.C. Sloan, (19 ex. FSCA); same as previous except: 3.vi.1954 (18 ex. FSCA); Highlands Co., Archbold Biol. Sta., 7.vi.1975, leg. L.L. Lampert (4 ex. FSCA); same as previous except: 2.iv.1979, leg. L.L. Lampert Jr. (2 ex. FSCA); Highlands Hammock State. Prk., 3.v.1974, leg. J.B. Heppner, at (UV) blacklight (9 ex. FSCA); same as previous except: 15.iii.1974, leg. R.E. Beer (1 ex. KSEM); Hillsborough Co., Morris Bridge Rd., 5.vii.1975, leg. S. Janisch (1 ex. FSCA); same as previous except: 4.vii.1977, leg. Boyd (1 ex. FSCA); “U.S.F Golf Course”, 21.v.1975, leg. “R.H” (1 ex. FSCA); “U.S.F. Campus”, 26.vi.1972 (1 ex. FSCA); same a previous except: 8.v.1979, leg. “S.T.” (1 ex. FSCA); Levy Co., 6.v.1950, leg. S.R. Young, (2 ex. FSCA); Okaloosa Co., 3mi S. of Holt Log Lake Bridge, 4.x.1966, leg. P.A. Thomas (9 ex. FSCA); Pasco Co., Crystal Springs, vii.1972 (1 ex. FSCA); same a previous except: 5.i.1975, leg. M. Lopez (1 ex. FSCA); Crystal Springs, Hill City, 5.i.1975, leg. J. Ward (1 ex. FSCA); Crystal Springs, “Hillshore”, 5.i.1975, leg. Diemer, (1 ex. FSCA); Polk Co., Mulberry, 24.vii.1972 (1 ex. FSCA); Seminole Co., 5.iv.1941, leg. M.J. Westfall Jr. (1 ex. FSCA); Taylor Co., Esconfina R. at US 98, 31.v.1987, leg. F.N. Young, #3189 (5 ex. FSCA); Volusia Co., Daytona Beach, 18.iv.1960, leg. R.E. Woodruff (1 ex. FSCA).

##### Diagnosis.

Male (Fig. 40C–D): Size: 9.9–11.7 mm. Body form broadly elongate oval; elytral apices flatly rounded, rarely with sutural angle produced to a point, apicolateral sinuation strongly present, serration and irregularities present apically near sutural production, elytral apices weakly deflexed, reticulation of pronotal fine, producing a polished appearance; elytral striae faintly evident, most evident on the medial portion of the elytral disc; profemora with large sub-apicoventral tooth; protibiae subsinuate to weakly-subsinuate; mesotarsal claws (Fig. 41C) small and of similar size, with ventral margin with a more less developed smooth denticle; venter lightly colored, red in coloration for its entirety, with the mesothoracic and metathoracic legs usually lighter in coloration, as well as apex of abdomen; Aedeagus (Fig. 41A, B, D) with median lobe in dorsal view as long as parameres, highly parallel sided, narrowed in apical 1/4, apex flatly to regularly rounded, in lateral view median lobe weakly curved dorsally after basal 1/3, in ventral view sperm-groove broad, parameres in apical 1/3 weakly laterally expanded, apically obliquely flatly rounded to truncate.

Female (Fig. 40A–B): Size: 9.6–11.3 mm. Body form elongate oval; elytral apices flatly rounded to flatly rounded toward apical production, sutural angle often produced to a point, apicolateral sinuation shallow, serration present apically near sutural production, elytral apices weakly deflexed, reticulation of pronotal fine, producing a polished appearance; elytral striae faintly evident, most evident on the medial portion of the elytral disc, profemora without sub-apicoventral tooth; protibiae club shaped, with lateral margin flatly round; venter lightly colored, red in coloration for its entirety, with the mesothoracic and metathoracic legs usually lighter in coloration, as well as apex of abdomen.

##### Differential diagnosis.

*Dineutus serrulatus serrulatus* is unique among all other North American *Dineutus* in being elongate oval and attenuated anteriorly, having the elytral apices flatly rounded with serration present apically near the suture, both sexes with apicolateral sinuation present, being deeply sinuate in the males, males with a large and highly acute sub-apicoventral profemoral tooth, and in the form of the aedeagus. The species most similar to *Dineutus serrulatus serrulatus* is *Dineutus carolinus*. Both *Dineutus serrulatus serrulatus* and *Dineutus carolinus* have the elytral apices with serrations and or irregularities present apically. *Dineutus serrulatus serrulatus* of both sexes can be distinguished fairly easily from *Dineutus carolinus* in the color of the venter. The venter of *Dineutus serrulatus serrulatus* is more lightly colored (Fig. 40B, D), varying from light orange red to a dark orange red, while that of *Dineutus carolinus* is darkly colored, dark reddish brown to black (Fig. 10B, D). The body form between the two species also differs in *Dineutus serrulatus serrulatus* is more attenuated anteriorly, while *Dineutus carolinus* is more regularly elongate oval in body form

Males of *Dineutus serrulatus serrulatus* can be further distinguished from *Dineutus carolinus* in having a strong apicolateral sinuation present in the elytra. Males of *Dineutus carolinus* do not have apicolateral sinuation present. The profemoral sub-apicoventral tooth will also separate the species in that *Dineutus serrulatus serrulatus* has a very large acute tooth, while that of *Dineutus carolinus* is much smaller. The mesotarsal claws are also very different between these two species, in *Dineutus serrulatus serrulatus* the ventral margin has a denticle (Fig. 41C), while in *Dineutus carolinus* the ventral-margins are shallowly rounded and without a denticle (Fig. 11C). The male aedeagus will allow unambiguous separation of the two species. In *Dineutus serrulatus serrulatus* the median lobe of the aedeagus (Fig. 41A) is highly parallel sided, being only narrowed in the apical 1/4 while *Dineutus carolinus* the aedeagus is gradually tapered from base to apex (Fig. 11A). The parameres differ drastically between the species. In *Dineutus serrulatus serrulatus* the parameres are much more narrow and are obliquely truncate to obliquely flatly rounded, while in *Dineutus carolinus* the parameres are thick and broad and are apically flatly rounded, not obliquely angled.

Females of *Dineutus serrulatus serrulatus* are more difficult to distinguish from females of *Dineutus carolinus* as both have an apicolateral sinuation present in the elytra. But the venter coloration and body form differences should allow separation of these two species.

*Dineutus serrulatus serrulatus* can be distinguished from *Dineutus serrulatus analis* by the characters given in the key.

##### Distribution

**(Fig. 53C).** Mostly known from extreme southeastern United States, primarily from Florida (Epler 2010; Folkerts 1978; Régimbart 1882; Sanderson 1982; Wood 1968; Young 1954).

##### Habitat.

*Dineutus serrulatus serrulatus* is a lotic species (Young 1954; Wood 1968) and appears to occupy small streams below 500 feet in elevation (Wood 1968). In Florida *Dineutus serrulatus serrulatus* is commonly found in small, rather swift, sand bottomed streams of the uplands throughout the northern and central peninsular region, as well as in the western uplands (Young 1954). Young (1954) suggests that *Dineutus serrulatus serrulatus* is more rarely found in the swifter streams of the flatwoods. Young (1954) also notes that *Dineutus serrulatus serrulatus* is rarely found in the same habitat as *Dineutus angustus* in the peninsular uplands of Florida, and attributes this to *Dineutus serrulatus serrulatus* being found in slightly more acidic streams.

##### Discussion.

It should be pointed out that *Dineutus serrulatus serrulatus* has the more narrower of the two subspecies ranges, occupying the most southeastern portion of the species range (Fig. 53C).

#### Dineutus solitarius

Taxon classificationAnimaliaColeopteraGyrinidae

Aubé, 1838

**Figure 42.**
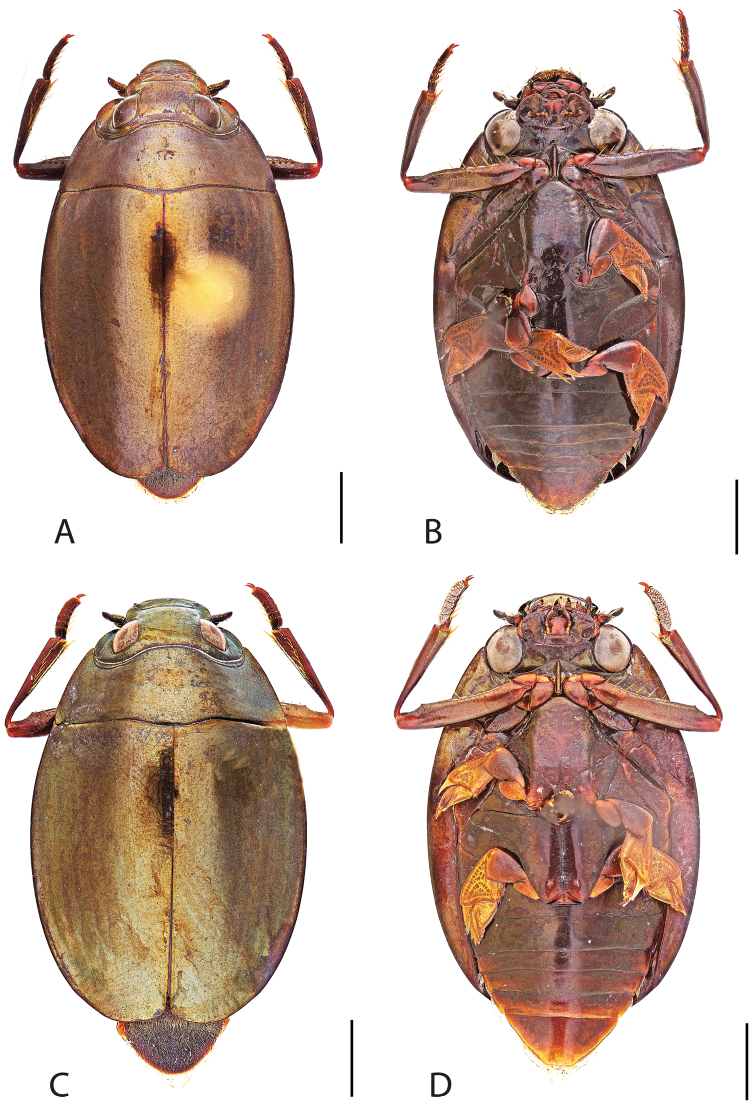
*Dineutus solitarius*. **A** ♀ dorsal habitus **B** ♀ ventral habitus **C** ♂ dorsal habitus **D** ♂ ventral habitus. All scale bars ≈ 2 mm.

**Figure 43.**
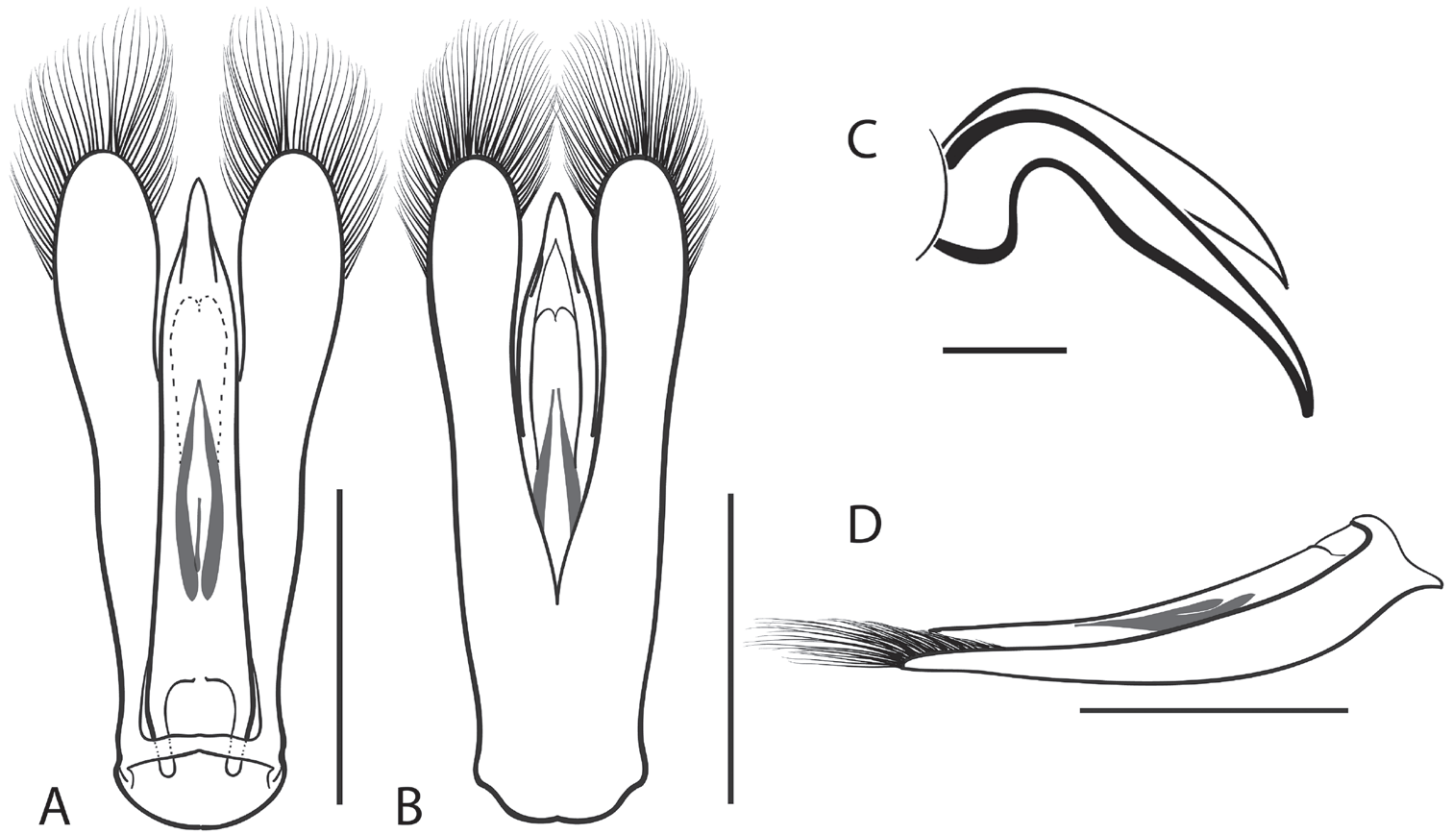
*Dineutus solitarius*. **A** aedeagus dorsal view **B** aedeagus ventral view **C** ♂ mesotarsal claws **D** aedeagus lateral view. Scale bar for **C** ≈ 0.10 mm all others ≈ 1 mm.

Figures 42 , 43 , 46 , 51 , 54

Dineutes solitarius Aubé 1838: 780, Dineutus (Cyclinus) solitarius: Hatch 1925b: 137, *Dineutus solitarius*: Leech 1940: 74, Dineutus (Cyclinus) solitarius: Leech 1948: 422, *Dineutus solitarius*: Arce-Pérez and Roughley 1999: 84.

##### Type locality.

Mexico, Veracruz

##### Specimens examined.

87

##### Type material examined.

Lectotype, here designated (1 ♂ male pinned) “MUSEUM PARIS/ VERA-CRUZ/ 1833 [beige label, typed black ink]// green disc [underneath is handwritten Veracruz/ 1883// in black ink]// *solitarius* [beige label, handwritten in black ink, handwriting appears to be Aubé’s]// TYPE [white label, typed red ink]// LECTOTYPE [red label, typed black ink]//” deposited in the MNHN.

##### Material examined.

**COSTA RICA:** **Guanacaste:** Santa Rosa N.P.,10°50.35'N, 85°37.07'W, 300 m, 6.vi.2008, leg. E. Nearns, I. Swift (2 ex. MSBA); 0.25km S Santa Rosa N.P., roadside pool, 15.vi.2003, leg. W.D. Shepard, EMEC 204684-204687; EMEC 204675 (5 ex. EMEC); La Pacifica nr Canas, 8.vi.1983, leg. J.E. Wappes, (1 ex. FSCA). **EL SALVADOR:** **La Libertad:** Hacienda Capolinas, 5 km NW Quezaltepeque, 455 m, 21.xii.1964, leg. M.E. Irwin (1 ex. UCRC). **GUATEMALA:** **Jalapa:** 4-7 km E. Jalapa, 12.vi.1991, leg. J.E. Wappes (1 ex. FSCA). **HONDURAS:** **Comayagua:** Malootal Minas de Oro, v.1932, leg. J.B. Edwards (3 ex. KSEM); **Francisco Morazán:** 4.5 km S.E. El Zamorano, 25.iv.1993, leg. I. Stange & R. Miller (1 ex. FSCA); **La Paz:** La Paz, 21.vii.1978, leg. V. Diaz, EMEC 204672 (1 ex. EMEC). **MEXICO:** **Chiapas:** 2.7mi W Colonia, Lazaro Cardenas, 6.viii.1965, leg. J.D. McCarty, EMEC 654699; EMEC 204761; EMEC 204769; EMEC 204777; EMEC 204801; EMEC 204807-204809 (8 ex. EMEC); 20 mi W of Cintalapa, 31.xii.1955, leg. J.C. Schaffner (2 ex. FSCA); **Guerrero:** Rincon, “kil.-256 S. MexCity”, 31.x.1936, leg. H.D. Thomas (1 ex. KSEM); **Jalisco:** UNAM Biol. Sta. Chamela, 61 m, 9.viii.1982, leg. C.W. & L. O’Brien & G. Wibmer, at light (1 ex. FSCA); Est. Biol. Chamela, at lites, 13-22.vii.1992, leg. J. Chemsak, EMEC 204753; EMEC 204881; EMEC 204897; EMEC 204925 (4 ex. EMEC); 20 mi N Puerto Vallerta, “200”, 29.viii.1971, leg. J. Cicero (1 ex. FSCA); **Nayarit:** 7 mi. N Tetitlan, 14.vi.1962, leg. D.H. Janzen, EMEC 204772-204773; EMEC 204786; EMEC 204800; EMEC 204805-204806; EMEC 204857-204858; EMEC 204917-204920 (11 ex. EMEC); El Pichon, 25.vi.1963 (1 ex. FSCA); Jesus Maria, 26.vi.1955, leg. B. Malkin, EMEC 204804 (1 ex. EMEC); Sierra Zapotan, 1300 m, iii.1943, leg. E. Paredes (3 ex. UCRC); 24 mi SE Tepic, 1045 m, 22.vi.1968, leg. A.R. Hardy, L. Espinosa, J.P. Abrayaya (2 ex. UCRC); 20.3 mi W Compostela, 60 m, 19.vi.1967, leg. A.R. Hardy (1 ex. UCRC); **Nuevo León:** 10km N Linares, mercury vapor lamp, 430 m, 23.iii.1991, leg. R. Brooks, R. Leschen, Coll. No. 58 (1 ex. KSEM); **Oaxaca:** 5 mi N La Ventosa, 4.vii.1970, leg. R.E. Beer & party (3 ex. KSEM); 80km N of Arriga,10.vi.1971, leg. S.R. & L.M. Steinhauser (2 ex. FSCA); **Quintana Roo:** 10.9km S Playa del Carmen,1.vii.1990, leg. M.C. Thomas, (1 ex. FSCA); Isla de Cozumel, SW side 2mi W Cedral, 8.x.1993, leg. C.B. Barr & W.D. Shepard, EMEC 654700 (1 ex. EMEC); **San Luis Potosí:** El Salto Falls, 15.vi.1956, leg. R.E. Beer & party (5 ex. KSEM); El Salto, 19.vi.1953, Univ. Kans. Mex. Expedition (2 ex. KSEM); same as previous except: 488 m, 24.viii.1954 (1 ex. KSEM); same as previous except: 381 m, 4.ix.1962, leg. Ordway & Marston, at light (1 ex. KSEM); **Sinaloa:** Culiaoan, 6 mi S, Black & White lights, 6.viii.1964, leg. J.A. Chemsak & J. Powell, EMEC 204803 (1 ex. EMEC); 5 mi N Mazatlan, at light, 11.x.1975, leg. J. Powell, J. Chemsak, T. Friedlander, EMEC 204778 (1 ex. EMEC); 15 mi SE Mazatlan, 27.vii.1973, leg. J. Chemsak, E.G. Linsleys & A.E. Michelbacher, EMEC 204779 (1 ex. EMEC); **Veracruz:** 15mi NW of Acayucan, 18.vi.1958, leg. J.C. Schaffner, (1 ex. FSCA); Palma Sola, “255 Pastizal”, 23.viii.1973, leg. G. Halffter & P. Reyes, Blacklight trap (1 ex. FSCA); 6mi SE Rinconada, 21.vi.1962, leg. D.H. Janzen, EMEC 204921 (1 ex. EMEC). **NICARAGUA:** **Rivas:** E of Lago de Apanás, 13°12.77'N, 86°58.06'W, 966 m, 12.vi.2005, leg. W.D. Shepard, EMEC 204676-204683 (8 ex. EMEC); **Jinotega:** roadside pool, Lago de Apanás area, N Jinotega, 13°12.8'N, 85°58.1'W, 966 m, 12.vi.2005, leg. C.B. Barr, EMEC 204673-204674 (2 ex. EMEC). **U.S.A.:** **California:** Riverside Co., Mecca, 15.viii.1924, EMEC 654698 (1 ex. EMEC); **Texas:** “Pinto”, 7.vii.1938, leg. D. W. Craik (1 ex. KSEM).

##### Diagnosis.

Male (Fig. 42C–D): Size: 9.2–10.4 mm. Body form broadly oval; elytral apices regularly broadly rounded, with serrations and irregularities absent apically, elytra reticulation normally coarse and well impressed laterally, medially being replaced by fine microreticulation, elytral disc medially often with fine and weakly impressed punctures, striae very faintly present, most evident medially on elytral disc; profemora with small sub-apicoventral tooth atop profemoral carina; protibiae weakly subsinuate to club-shaped; mesotarsal claws (Fig. 43C) with ventral margin straight, with weak medial expansion; venter darkly colored, usually black to very dark reddish brown, mesothoracic and metathoracic legs usually lighter in coloration, as well as apex of abdomen; Aedeagus (Fig. 43A, B, D) median lobe in dorsal view just shorter than parameres, mildly parallel sided, weakly angled towards apex, acuminate in apical 1/5, apical median lobe angled towards acumination, lateral margins of acumination angled toward apex, apex very shortly rounded producing a strong point, in lateral view median lobe evenly shallowly curved dorsally, ventrally median lobe with diamond shaped sperm-groove, parameres in dorsal view weakly expanded laterally at apical 1/3, narrowly rounded apically.

Female (Fig. 42A–B): Size: 9.1–10.2 mm. Body form regularly oval; elytral apices regularly broadly rounded, with serrations and irregularities absent apically, apicolateral sinuation absent, elytra reticulation course and well impressed laterally, medially being replaced by fine microreticulation, elytral disc medially often with fine and weakly impressed punctures, striae very faintly present, most evident medially on elytral disc; profemora without sub-apicoventral tooth; protibiae club-shaped; venter darkly colored, usually black to very dark brown, mesothoracic and metathoracic legs usually lighter in coloration, as well as apex of abdomen.

##### Differential diagnosis.

*Dineutus solitarius* is unique among all other North American *Dineutus* in being smaller in size (9.1–10.4 mm), with a broadly oval body form (Fig. 42C), having the elytral apices broadly rounded without serrations and/or irregularities, males with the profemoral sub-apical ventral tooth small and atop a carina, and by the form of the aedeagus (Fig. 43A). The species most similar to *Dineutus solitarius* are *Dineutus carolinus* and *Dineutus emarginatus*. Both sexes of *Dineutus solitarius* can be distinguished from *Dineutus carolinus* in having the elytral apices broadly rounded without apical serrations and/or irregularities, as opposed to having the elytral apices narrowly rounded with serrations and/or irregularities present. The body form of *Dineutus solitarius* is much more regularly oval as opposed to being more elongate overall in *Dineutus carolinus*. *Dineutus solitarius* lacks a defined lateral marginal depression as seen in *Dineutus carolinus* as a result of being more dorsoventrally convex.

Of the two species similar to *Dineutus solitarius*, *Dineutus emarginatus* is more similar, in that both of these species have the elytral apices fairly broadly rounded and without apical serrations and/or irregularities present. Furthermore, the aedeagi of these two species are somewhat similar both being acuminate. In general both sexes of *Dineutus solitarius* can be distinguished from *Dineutus emarginatus* in being much more regularly rounded in body form than *Dineutus emarginatus*, whose body form is more elongate oval. The pronotal shape of the two species differs fairly noticeably, in *Dineutus solitarius* the pronotum has the lateral borders much more obtusely angled posteriorly to anteriorly, and the posterior margin of the pronotum flatly meets the posterolateral corners of the pronotum, while in *Dineutus emarginatus* the pronotum has the lateral borders more straightly angled posteriorly to anteriorly with the posterior margin being more sinuate.

Males of *Dineutus solitarius* can further be removed from *Dineutus emarginatus* in having a small profemoral sub-apicoventral tooth atop a carina, while in *Dineutus emarginatus* the tooth is much more large and triangular. The aedeagi of these two species are similar in that both have an acuminate median lobe, however the general shape of the median lobe of the two aedeagi can be separated without difficulty. The median lob of *Dineutus solitarius* (Fig. 43A) is much more parallel sided, being weakly narrowed towards the acumination and the acumination itself having its margins angled towards the apex, which is very narrowly rounded giving it a very pointed feel. In *Dineutus emarginatus* the median lobe (Fig. 20A) is weakly constricted medially giving the lateral margins a slight sinuation, the apical margins of the median lobe are rounded towards the acumination, with the acumination having its lateral margins fairly straight, and the apex relatively more broadly rounded, giving it a more rounded pointed shape, as opposed to narrowly pointed as in *Dineutus solitarius*.

The females of *Dineutus solitarius* differ primarily in the general differences between the two species.

##### Distribution

**(Fig. 54B).** From extreme southern United States, Texas (“Pinto Texas” 1938 [KSEM]) and California (Leech 1940; Leech 1948), throughout Mexico (Ochs 1949) and Central America to western Costa Rica. The first author visited the only known California locality, the city of Mecca, in Riverside County, in southwestern California, in the summer of 2012, in an attempt to recollect this species. The area had changed quite a bit with much of the duck ponds in the area having dried up and an increased agricultural presence was evident.

##### Habitat.

This species appears to be lotic. Several locality labels list “pools in stream”. In La Selva Negra, Nicaragua, specimens were collected from a pool within a stream by the second author.

##### Discussion.

It should be noted that *Dineutus solitarius* is primarily a Mexican and Central American species, only just barely reaching the United States. Historical records indicate it was at one point found in California (Leech 1940) and Texas.

There is some noticeable variation in the elytral sculpturing among populations of *Dineutus solitarius*. Specimens from Sierra de Zapotan, Nayarit, Mexico (UCRC) have the punctures of the elytra much more shallowly and weakly impressed, making them much larger and “dimply” in appearance (Fig. 46B). This is most noticeable medially on the elytra discs near the sutural region, where the strong lateral reticulation is replaced by the fine mesh microreticulation. Interestingly a similar situation is seen in specimens from the same area of *Dineutus sublineatus* (Fig. 46A). These specimens with the dimply punctation make it apparent that the fine weakly impressed punctation seen normally medially on the elytral discs is also present laterally, but is obscured by the strong reticulation of that area, and is visible medially and suturally due to the fine microreticulation of that area. Specimens from Costa Rica (MSBA) also show variation in elytral sculpturing, having the reticulation of the elytra composed of much finer meshes. The lateral meshes are still stronger but not nearly as coarse as in other populations. Specimens of *Dineutus solitarius* from Oaxaca, Mexico, near Arriba, (FSCA) show variation in body form. The outline of the body is much more broadly oval in these specimens. There is also a much greater dorsoventral convexity in the scutellar region, especially evident in lateral view. It should be noted that although there are these variations among populations, the aedeagi of males from each of these populations are not noticeably different or variable in any significant manner.

##### Type designation.

Similar to the situation with *Dineutes metallicus* Aubé, the only specimen with a date and Aubé’s handwriting for the determination label was selected as the lectotype (Fig. 51C). The other specimens in the MNHN collection lacked dates and/or Aubé’s handwriting, therefore we did not designate paralectotypes.

#### Dineutus sublineatus

Taxon classificationAnimaliaColeopteraGyrinidae

(Chevrolat, 1834)

**Figure 44.**
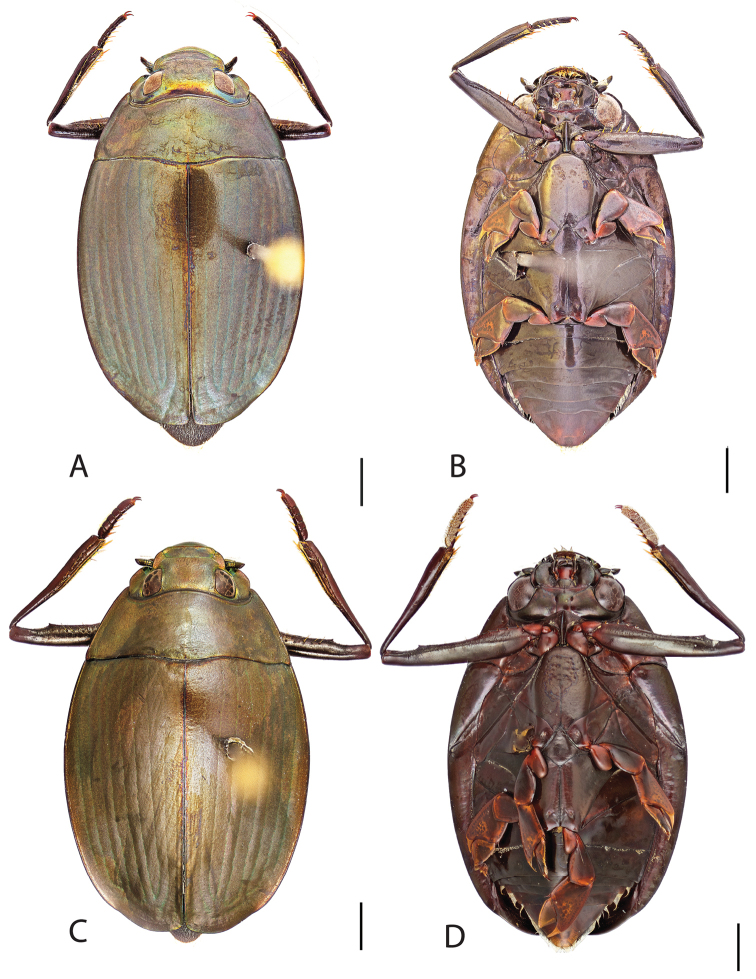
*Dineutus sublineatus*. **A** ♀ dorsal habitus **B** ♀ ventral habitus **C** ♂ dorsal habitus **D** ♂ ventral habitus. All scale bars ≈ 2 mm.

**Figure 45.**
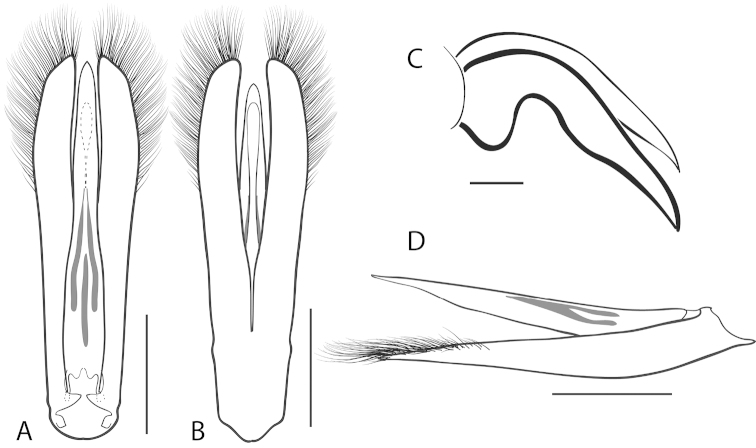
*Dineutus sublineatus*. **A** aedeagus dorsal view **B** aedeagus ventral view **C** ♂ mesotarsal claws **D** aedeagus lateral view. Scale bar for **C** ≈ 0.10 mm all others ≈ 1 mm.

**Figure 46.**
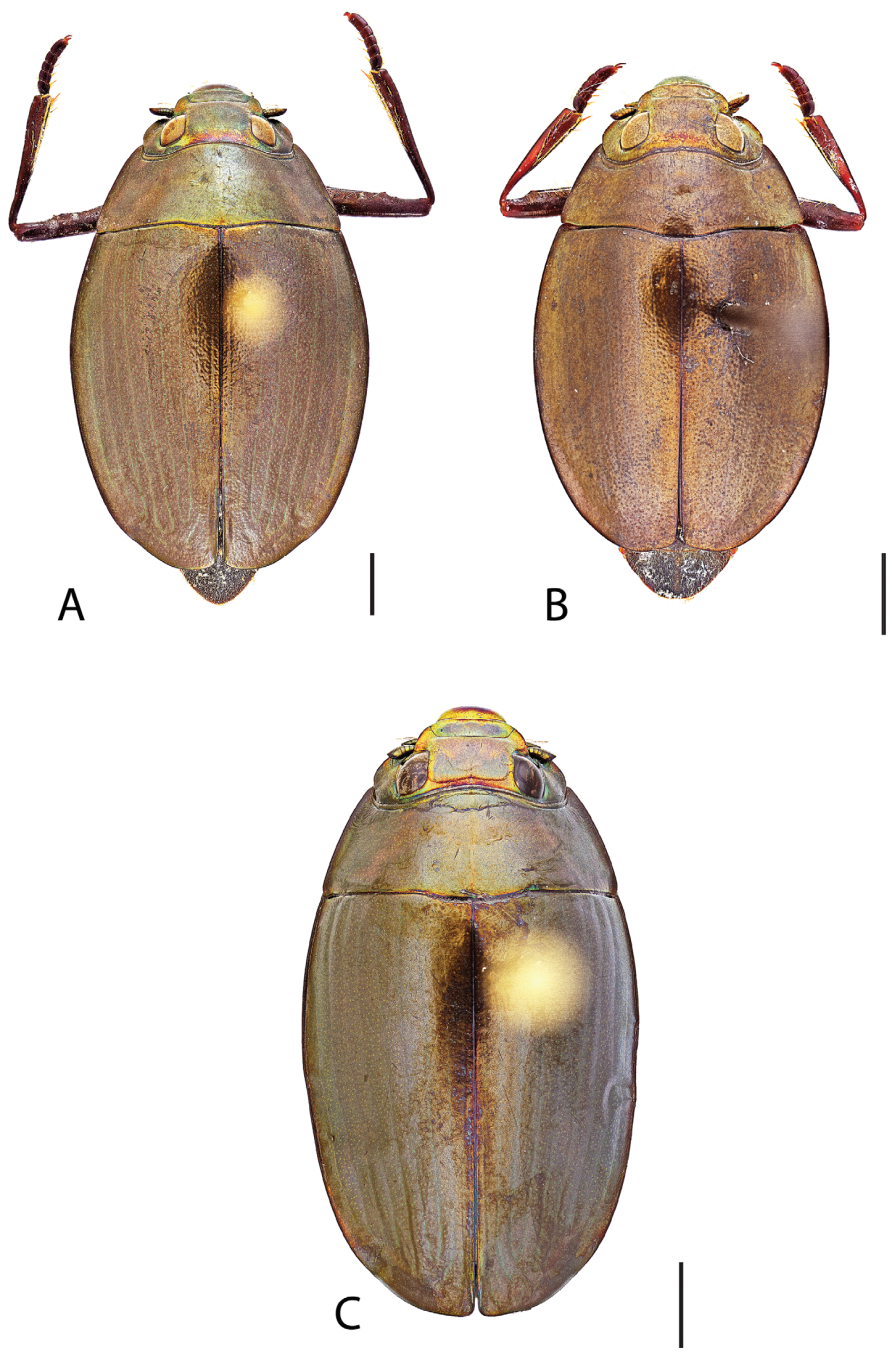
Variation in specimens from Sierra de Zapotan, Narayit, Mexico. **A** ♂ *Dineutus sublineatus* dorsal habitus **B** ♂ *Dineutus solitarius* male dorsal habitus. ♀ *Dineutus sublineatus* from Salama Guatemala **C** dorsal habitus. All scale bars ≈ 2 mm.

Figures 44 , 45 , 46 , 54

Gyrinus sublineatus Chevrolat 1834: 3, *Dineutes sublineatus*: Brullé 1835: 240, *Dineutes integer* LeConte 1854: 221 [synonymy by LeConte 1861], Dineutus (Dineutus) sublineatus: Ochs 1926: 138, Dineutus (Protodineutus) sublineatus: Guignot 1950: 126, *Dineutus sublineatus*: Blackwelder 1944: 81, Dineutus (Cyclinus) sublineatus: Brinck 1955: 106, *Dineutus sublineatus*: Arce-Pérez and Roughley 1999: 84.

##### Type locality.

Mexico, Bocadelmonte.

##### Specimens examined.

**326**

##### Type material examined.

*Dineutus sublineatus*: not examined. The first author was unable to locate the syntype of *Dineutus sublineatus* Chevrolat after searching the MNHN with the assistance of A. Mantilleri.

*Dineutus integer*: syntype (♀ pinned) “Type/ 6092 [red label, Type typed in black ink, 6092 handwritten in black ink]// Dineutus integer/ Webb Lec./ Copper Mines [white label, handwritten in black ink, handwriting appears to be LeConte’s]// sublineatus 2 [white label, handwritten in black ink, handwriting unknown]//” deposited in MCZ.

##### Material examined.

**EL SALVADOR:** **Chalatenango:** San Jose del Sacare, 15.iii.1927, leg. R.A. Stirton (2 ex. KSEM). **GUATEMALA:** **Baja Verapaz:** 15 km N Salama, 5.vi.1991, leg. J.E. Wappes (1 ex. FSCA). **HONDURAS:** **Comayagua:** Malootal Minas de Oro, v.1932, leg. J.B. Edwards (1 ex. KSEM). **MEXICO:** **Aguascalientes:** Sierra Fria ca. 40 mi SW Rinconde Romos, 2438 m, 11.iii.1953, leg. I.J. Centrall, #55 (2 ex. FSCA); **Baja California Sur:** Bahía Concepción, “Lanito”, 15 m, 16.iv.1968, leg. M.E. Irwin (1 ex. UCRC); Sierra Laguna, 17 air mi ENE Todos Santos, 1829 m, 12-18.xii.1979 EMEC 204906-204907 (2 ex. EMEC); Sierra Laguna, 17 mi ENE Todos Santos, 12-18.xii.1979, leg. J. Doyen, W. Tschinkel, EMEC 204770-204771; EMEC 204793-204799; EMC 204811-204815; EMEC 204817-204823; EMEC 204827-204849; EMEC 204860 (47 ex. EMEC); Arroya Posa ca. 10 mi W Loreto, 183 m, 6.vi.1978, leg. C.M. Murvosh & R.K. Allen, EMEC 654702-654710 (9 ex. EMEC); 9 mi WSW Loreto, Arroyo Las Parras, 207 m, 10.vii.2004, leg. W.D. Shepard, EMEC 204602-204619 (18 ex. EMEC); 9 mi SW Loreto, Arroyo Las Parras, 250 m, 20.vii.2004, leg. W.D. Shepard, EMEC 204620-204644 (25 ex. EMEC); 0.6mi S San Javier, 387 m, 20.vii.2004, leg. W.D. Shepard, EMEC 204668-204669 (2 ex. EMEC); Ram di Naran Rd., 10-28k W Rt 1, 28-31.viii.1994, leg. J.E. Wappes (1 ex. FSCA); 4.3 km E La Burrera, 550 m, 11-14.x.1978, leg. Dezier & Westcott, canyon-stream (1 ex. FSCA); **Chihuahua:** 3 mi NW Chihuahua, 20.viii.1952, leg. J.D. Lattin, S. Weitsman, EMEC 204861 (1 ex. EMEC); 15 mi E Cuauhtemoc, Black & White lights, 2012 m, 11.vii.1964, leg. J.A. Chemsak & J. Powell, EMEC 204916 (1 ex. EMEC); 13 mi E Cuauhtamoc, Black & White lights, 2012 m, 11.vii.1964, leg. J.A. Chemsak & J. Powell, EMEC 204825 (1 ex. EMEC); Hidalgo, 25 mi W Del Parral, Black & White lights, 2073 m, 15.vii.1964, leg. J.A. Chemsak & J. Powell, EMEC 204904 (1 ex. EMEC); Hidalgo, 25 mi W Parral, Black & White lights, 2073 m, 15.vii.1964, leg. J.A. Chemsak & J. Powell, EMEC 204824 (1 ex. EMEC); Chihuahua, 1524 m, 4.ix.1963, leg. H.V. Weems Jr., collected at light (1 ex. FSCA); **Chiapas:** El Zapotal, 2 mi S Tuxtla Gutierrez, 15.vii.1956, leg. J.W. MacSwain, D.D. Linsdale, EMEC 204888; EMEC 204892 (2 ex. EMEC); El Chorreodero, 600 m, 15.v.1991, leg. J.D. McCarty, EMEC 204645 (1 ex. EMEC); Simojovel, 1-16.viii.1958, leg. J.A. Chemsak, EMEC 204759; EMEC 204909 (2 ex. EMEC); **Coahuila:** 1 mi SE Saltillo, at blacklight, 23-26.ix.1976, leg. J.A. Chemsak & J.A. Powell, EMEC 204792 (1 ex. EMEC); **Durango:** Nombre de Dios, 25.vi.1952, leg. E.E. Gilbert, C.D. MacNeil, EMEC 204859; EMEC 204911-204915 (5 ex. EMEC); 6.3 mi N Nombre de Dios, 1829 m, 25.vi.1952, leg. R.F. Smith, EMEC 204910 (1 ex. EMEC); **Guerrero:** Mochitlan, Acahuizotla, Bosque tropical bajo caducifolio, 750 m, 11.vii.1986, leg. L. Delgado (1 ex. FSCA); Zumpango del Rio, 2.xi.1943, leg. E.K. Waering (1 ex. FSCA); **Jalisco:** Puerto Los Mazos, 9mi SW Autlan, 27.viii.1970, leg. M.S. & J.S. Wesbauer, EMEC 204776; EMEC 204780-204785 (7 ex. EMEC); 6 mi W Chapala, 30.vi.1963, leg. J. Doyen, EMEC 204790; EMEC 204787-204788 (3 ex. EMEC); Rio de Tepospisaloya, ca. 6 km N Union de Tula, 9.2 km W Tacotán, 20°03.86'N, 104°20.91'W, 381 m, 10.i.2005, leg. C.B. Barr, EMEC 654701 (1 ex. EMEC); **México:** NE of Temescaltepec, Canubi a Chichotla, 2027 m, 23.iv.2004, leg. W.D. Shepard, EMEC 204600-204601 (2 ex. EMEC); **Michoacán:** 26.7 km N La Huacana, 18.vii.1989, leg. R. Brooks, A. Roig, (1 ex. KSEM); **Morelos:** Alpuyeca, 3.vii.1951, leg. P.D. Hurd, EMEC 204757 (1 ex. EMEC); **Nuevo León:** 15 mi W Linares, 27.viii.1969, leg. J. Haddock, J.T. Doyen, EMEC 204774-204775 (2 ex. EMEC); 4 mi W of El Cercado, 6.vi.1951, leg. P.D. Hurd, EMEC 204758 (1 ex. EMEC); 4 mi S of Monterrey nr Siesta Motel, 6.vii.1963, leg. R.H. Arnett Jr., E.R. VanTassell, Lot No. V-56 (1 ex. FSCA); same as previous except: 7.vii.1963, Lot No. V-58 (8 ex. FSCA); 3.9 km NE Iturbide, ex: stream, 1410 m, 24.iii.1991, leg. Brooks, Leschen, #62 (1 ex. KSEM); 37 km SW Linares, 4.8 km S on Bosque Esquela Rd., ex:pool, 1545 m, 20.iii.1991, leg. Brooks, Leschen, #13 (1 ex. KSEM); 10 mi W of Cola del Caballo Falls, 24.xii.1972, leg. J.C. Schuster (2 ex. FSCA); **Nayarit:** 7mi N Tetitlan, 14.vi.1962, leg. D.H. Janzen, EMEC 204746-204751; EMEC 204754-204755; EMEC 204767; EMEC 204850-204852; EMEC 204854-204855; EMEC 204862-204865; EMEC 204869-204880; EMEC 204882-204883; EMEC 204885-204886; EMEC 204898; EMEC 204922-204924 (38 ex. EMEC); La Mesa de Nayar, 19.vii.1955, leg. B. Malkin, EMEC 204867-204868; EMEC 204887; EMEC 204889-204891; EMEC 204893; EMEC 204895-204896; EMEC 204899-204905; EMEC 204914 (16 ex. EMEC); Sierra de Zapotan, iii.1943, leg. E. Paredes, Pool in stream (3 ex. FSCA); Sierra Zapotan, 1300 m, iii.1943, leg. E. Paredes (2 ex. UCRC); 24 mi SE Tepic, 1045 m, 22.vi.1968, leg. A.R. Hardy, L. Espinosa, J.P. Abrayaya (1 ex. UCRC); **Oaxaca:** 4 mi W Tehuantepec, 21.vii.1952, leg. E.E. Gilbert, C.D. MacNeil, EMEC 204763-204766 (4 ex. EMEC); Oaxaca, ix.1957 (1 ex. FSCA); same as previous except: 28.ix.1957 (1 ex. FSCA); same as previous except: 29.ix.1957 (4 ex. FSCA); 33 mi W of Tehuantepec, 762-914 m, 22.viii.1963, leg. H.V. Weems Jr., in pool in mt. stream (12 ex. FSCA); **Puebla:** 11 mi SE Acatlan, 10.vii.1952, leg. E.E. Gilbert, C.D. MacNeil, EMEC 204760; EMEC 204762 (2 ex. EMEC); 45 mi N Acatan, 30.vii.1963, leg. J. Doyen, EMEC 204789; EMEC 204791 (2 ex. EMEC); 3 mi NW Petlalcingo, 1402 m, 29.viii.1972, leg. Byers & Thornhill (1 ex. KSEM); **Sinaloa:** 21 mi E Ville Union, Black & White lights, 91 m, 25.vii.1964, leg. J.A. Chemsak & J. Powell, EMEC 204816 (1 ex. EMEC); 36 mi N of Mazatlan, 18.xi.1955, leg. J.C. Schaffner (1 ex. FSCA); **Sonora:** 7 mi W Alamos, Blk- & White lights, 8.viii.1964, leg. J.A. Chemsak, J. Powell, EMEC 204810; EMEC 204826 (2 ex. EMEC); 5 km N Alamos, 6.viii.1998, leg. M.S. Caterino, EMEC 204768 (1 ex. EMEC); 7 mi SE Alamos, 27.xi.1970, leg. K. Stephan (1 ex. FSCA); nr Guicochi, 1768 m, 25.i.1972, leg. V. Roth (1 ex. FSCA); **Veracruz:** Laguna Verde, 30.iii.1975, leg. C. Halffter, P. Reyes, BLT (2 ex. FSCA). **U.S.A.:** **Arizona:** Cochise Co., SW Reg. Sta., 5 mi SW Portal, 4.viii.1961, leg. J.M. Linsley, EMEC 204884 (1 ex. EMEC);1 mi up CarrCyn R., 9.vii.1972, leg. J.M. Cicero (2 ex. FSCA); 6 mi E Bisbee, 17.vii.1972, leg. J.M. Cicero, (1 ex. FSCA); Skeleton Canyon, 24.viii.1962, leg. D. Weems (1 ex. FSCA); Skeleton Canyon, 1372-1524 m, 24.viii.1962, leg. H.V. Weems Jr. (1 ex. FSCA); Cave Creek Canyon, 11.vii.1984, UVL (1 ex. FSCA); Coronado Ntl. For., Idlewild cmpgd, 18.v.1990, leg. R.S. Miller (1 ex. MTEC); Huachuca Mtns, Bear Cr., rock pools in strm, 31°22'24"N, 110°21'40"W, 26.iv.2000, leg. K.B. Miller, #2000-12 (1 ex. MSBA); Rucker Canyon, pools in strbed, 25.iv.2000, leg. K.B. Miller (1 ex. MSBA); Hidalgo Co., Skeleton Canyon, Peloncillo Mts., 1372-1524 m, 25.viii.1962, leg. H.V. Weems Jr., in stream (1 ex. FSCA); Pima Co., Tucson, v.1958, leg. L. Lenando (2 ex. MTEC); Tucson, 2.viii.1970, leg. K. Stephan (1 ex. FSCA); Tucson, Sabino Canyon, 29.v.1958, leg. L. Lenando (1 ex. MTEC); Mouth of Madera Canyon, 17.vi.1967, leg. B. Streit (7 ex. FSCA); Madera Canyon, 22.ix.1977, leg. R.S. Miller (1 ex. MTEC); Catalina Mts., 4.viii.1930, leg. L.K. Gloyd, 145 (4 ex. FSCA); Sabino Canyon, 22.viii.1968, leg. F. Hovore, 489-490 (2 ex. FSCA); Box Spring Canyon, 8.viii.1986, leg. R.S. Miller (1 ex. MTEC); same as previous except: 9.viii.1986 (1 ex. MTEC); same as previous except: 10.viii.1986 (1 ex. MTEC); Sta. Rita Exp. Range, 04.viii.1988, leg. S. Lajeunesse (1 ex. MTEC) same as previous except: leg. M.M. Hooten (1 ex. MTEC); Coronado Ntl. For., 6.ix.1938, leg. C.L. Hubbs family, 102b (1 ex. FSCA); Santa Catalina Mtns., Molino Canyon, 3.iii.1968 (5 ex. FSCA); same as previous except: 30.iii.1969 (1 ex. FSCA); Santa Cruz Co., Peña Blanca, 24.viii.1977, leg. R.S. Miller (2 ex. MTEC); same as previous except: 20.viii.1971, leg. J. Cicero (2 ex. FSCA); same as previous except: 16.vii.9172 (1 ex. FSCA); Peña Blanca Lake, 10.x.1990, leg. W.B. Warner (2 ex. FSCA); Peña Blanca Lake, 20.vii.1982, leg. G.H. Nelson, Ultraviolet light (2 ex. FSCA); Madera Canyon,15.vii.1972, leg. J. Cicero (2 ex. FSCA); Sycamore Canyon, 16.iv.1950, leg. R.R. Miller & H.E. Winn, M-50-40 (1 ex. FSCA); Mt. Hopkins, 27.vii.1992, leg. J.&M. Huether (1 ex. FSCA); Spring fed trib. to Peña Blanca Canyon, 17.iv.1950, leg. R.R. Miller & H.E. Winn, M50-41 (1 ex. FSCA); Santa Rita Mts., Gardner Canyon, 22.vi.1975, leg. K. Stephan (2 ex. FSCA); **New Mexico:** Hidalgo Co., Peloncillo Mtns., Clanton Canyon, pool in wash, 20.ix.2009, leg. A.B. Johnson et. al. (3 ex. MSBA); **Texas:** “Big Bend”, vii.1960, leg. D. Thornton (3 ex. FSCA); John Davis Co., Valentine, 13.vii.1927, leg. L.A. Anderson (2 ex. KSEM); Kerr Co., Kerrville, 2.vii.1938, leg. I. Norris (2 ex. FSCA).

##### Diagnosis.

Male (Fig. 44C–D): Size: 12.3–15.5 mm. Body form elongate broadly oval; elytral apices broadly or flatly rounded/subtruncate, with serrations and irregularities absent apically, apicolateral sinuation weakly present, each elytron with all 9 elytral striae well developed, elytral stria 1 and 2 often obscured suturally and near the scutellar region by dense microreticulation, often accompanied by fine weakly impressed punctures; profemora with highly acute sub-apicoventral tooth; protibiae club-shaped; mesotarsal claws similar in size with very weak denticle (Fig. 45C) venter darkly colored, usually black to dark reddish brown, mesothoracic and metathoracic legs usually lighter in coloration, as well as apex of abdomen; Aedeagus (Fig. 45A, B, D) median lobe in dorsal view nearly as long as parameres, narrow, becoming attenuated apically, apex very narrowly rounded, in lateral view median lobe becoming highly narrowed ventrally in apical 1/3, shallowly curved dorsally after basal 1/3, ventrally median lobe with parallel sided sperm-groove, parameres in dorsal view very weakly expanded laterally at apical 1/3, angled apically.

Female (Fig. 44A–B): Size: 12.6–14.0 mm. Body form regularly oval; elytral apices broadly or flatly rounded/subtruncate, with serrations and irregularities absent apically, apicolateral sinuation usually weakly present, each elytron with all 9 elytral striae well developed, elytral stria 1 and 2 often obscured suturally and near the scutellar region by dense microreticulation, often accompanied by fine weakly impressed punctures; profemora without sub-apicoventral tooth; protibiae club-shaped; venter darkly colored, usually black to very dark brown, mesothoracic and metathoracic legs usually lighter in coloration, as well as apex of abdomen, abdominal sternite VII with medial expansion of posterior margin.

##### Differential diagnosis.

*Dineutus sublineatus* is unique among all North American *Dineutus* in being large in size, with a more or less regularly elongate oval body form, with all 9 elytral striae strongly evident, males with a small well developed and highly acute profemoral sub-apicoventral tooth, the form of the aedeagus (Fig. 45A), and females with abdominal sternite VII (Fig. 44B) with a medial expansion of the posterior margin. This species is highly unique in having the elytral striae well developed and highly evident. All other species have the elytral striae primarily faintly evident, or apparent but not strongly developed. All 9 elytral striae are evident, and often for most their length. Striae 1 and 2 can be obscured near the scutellar region as well as the near the sutural region of the elytra, by the microreticulation of these areas, which is often accompanied by fine weakly impressed punctures. An even more unique character of this species is the medial expansion of the posterior margin of abdominal sternite VII in the female. This is unique among the females of all Ne World *Dinuetus*. This species is similar to other Mexican and Central American species in coloration, being dorsally olive green with light violet reflections laterally on the elytra, and with the venter being very darkly colored black to reddish brown. However, *Dineutus sublineatus* unlike the other large Mexican and Central American species has the elytral apices regularly to flatly rounded/subtruncate, as opposed to being truly truncate.

##### Distribution

**(Fig. 54C).** Extreme southwestern U.S., throughout most of Mexico and central Central America to Nicaragua (Blackwelder 1944; Leech 1948; Ochs 1949; Régimbart 1882; 1907; Roberts 1895; Wood 1962).

##### Habitat.

Lotic and lentic. In Oaxaca, Mexico, 43 mi w of Tehuantepec *Dineutus sublineatus* was collected in pools in mountain streams at 2500–3000 ft in elevation (FSCA). Other label data also indicate pools in steams. In the United States the second author has collected this species in pools in dry streambeds within canyons in southern Arizona.

##### Discussion.

There is dimorphism in body form among the sexes of *Dineutus sublineatus*. Females tend to be more evenly elongate regularly oval, while males have a greater dorsoventral convexity in the scutellar region (especially evident in lateral view) resulting in a steepness in the humeral region of the elytra and the posterolateral region of the pronotum. Males of *Dineutus sublineatus* also have the lateral margin, just posterior to the humeral region of the elytra, slightly expanded. As mentioned earlier females also have a medial exspansion of abominal sternite VII, not seen the males.

This species exhibits a large amount of variation throughout its range, with noticeable variation in body form. A series of specimens from near Tehuanatepec, Oaxaca, Mexico, has the females noticeably smaller than the males, and much more parallel sided in appearance, as well as both sexes with the elytral apices very flatly rounded, nearly truncate in appearance. A single female specimen from Salama, Guatemala (Fig. 46C) (UCRC), was observed with a highly parallel sided body form, with the elytral apices seemingly produced, and very flatly rounded. This specimen at first glance seemed like a different species, but the specimen possesses the well-developed elytral striae and abdominal sternite VII with a medial expansion, currently characteristic of only *Dineutus sublineatus*. Without a male to unambiguously place the Guatemalan specimen as a different species, we considered it a variation of *Dineutus sublineatus*, as it possessed the diagnostic females character of the medial expansion of abdominal sternite VII, thus far unique to *Dineutus sublineatus*.

Specimens from Siera de Zapotan, Narayit, Mexico (Fig. 46A) (UCRC), show variation in the punctation of the elytra, being composed of large shallowly impressed punctures, giving the elytra a dimply appearance. This situation is also seen in specimens from this locality in *Dineutus solitarius* (Fig. 46B). Other variation in the sculpturing of the elytra can be seen in several different populations. Some populations have the cells of the reticulation of the pronotal disc, as well as the elytral disc near the scutellar and sutural region, noticeably reflective, creating a bright metallic green color.

Even though there is a great deal of variability in these species in terms of body form, elytral apices, and elytral sculpturing, several characters are very stable. These include the strong development of the elytral striae in both sexes, in males the form of the aedeagus, and in females abomdinal sternite VII possessing a medial expansion. There is very minimal varation in the form of the male aedeagus, which is the most diagnostic character for species recognition within *Dineutus*.

#### Dineutus truncatus

Taxon classificationAnimaliaColeopteraGyrinidae

Sharp, 1873

**Figure 47.**
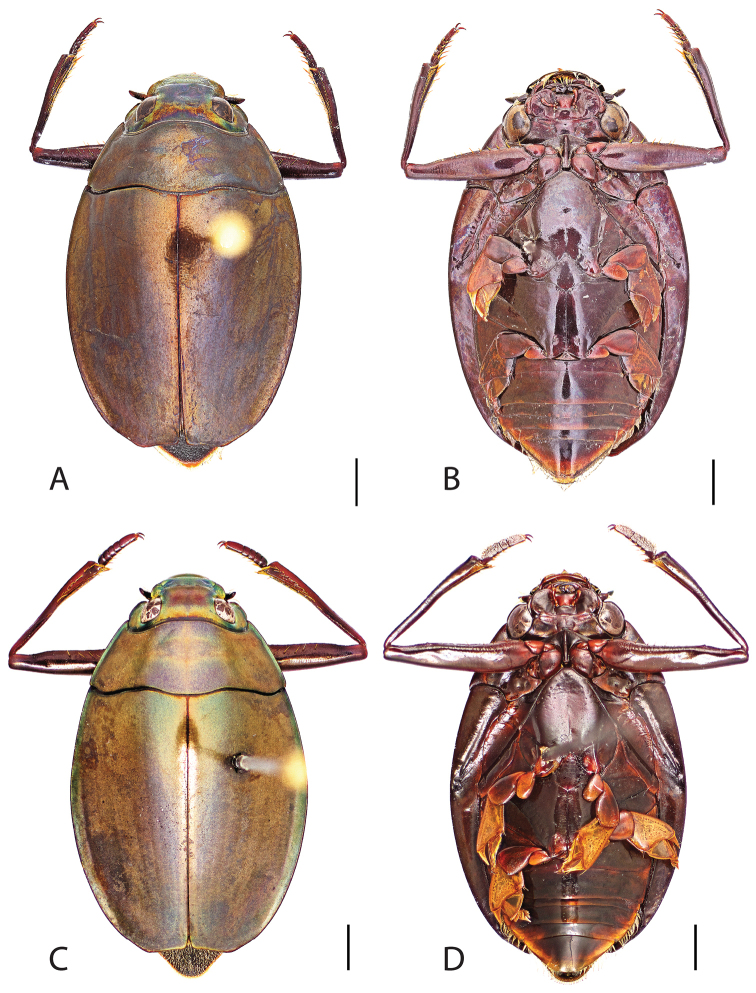
*Dineutus truncatus*. **A** ♀ dorsal habitus **B** ♀ ventral habitus **C** ♂ dorsal habitus **D** ♂ ventral habitus. All scale bars ≈ 2 mm.

**Figure 48.**
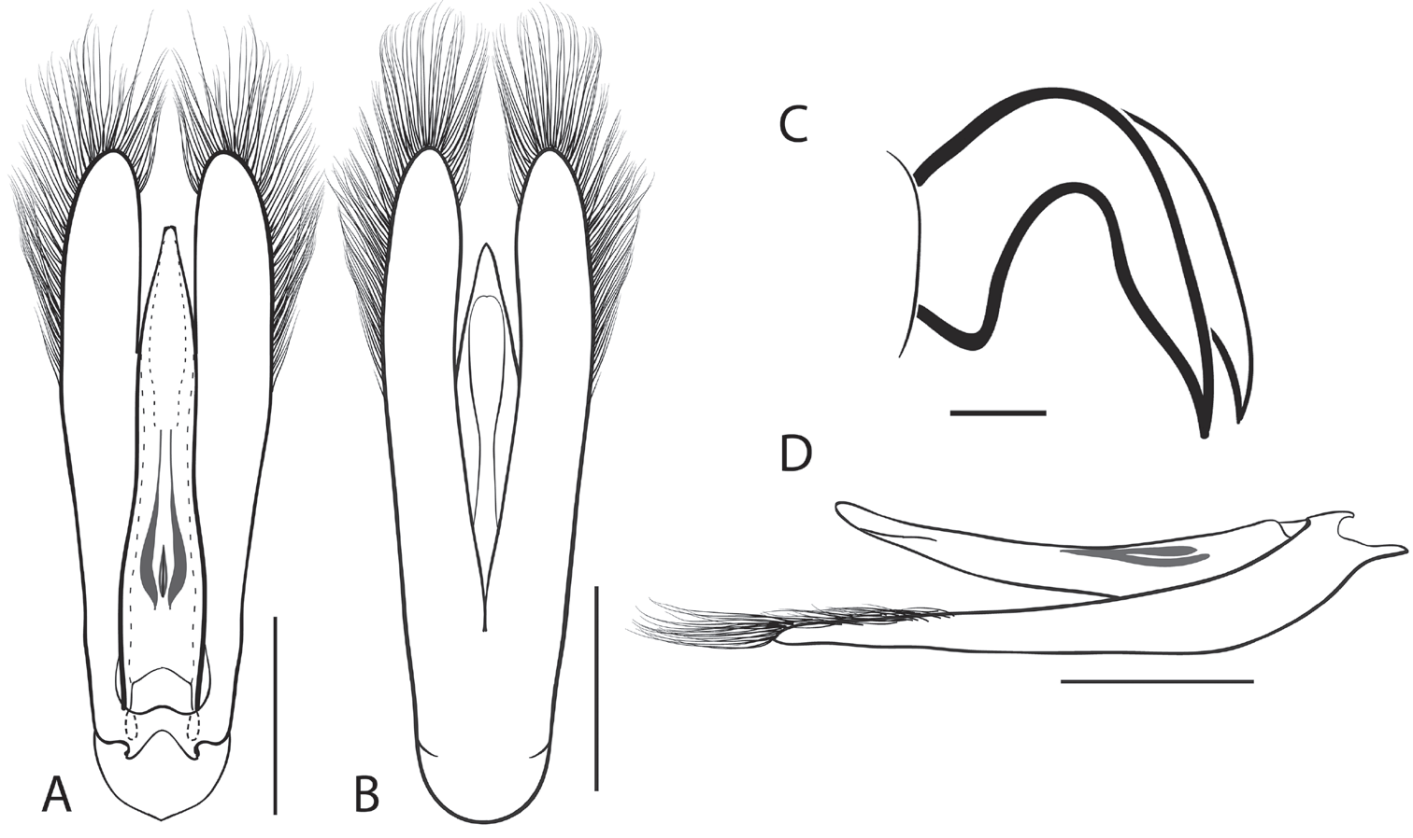
*Dineutus truncatus*. **A** aedeagus dorsal view **B** aedeagus ventral view **C** ♂ mesotarsal claws **D** aedeagus lateral view. Scale bar for **C** ≈ 0.10 mm all others ≈ 1 mm.

Figures 30 , 47 , 48 , 54

Dineutes truncatus Sharp 1873: 54, *Dineutus truncatus*: Ochs 1924: 1, Dineutus (Dineutus) truncatus: Ochs 1926a: 138, *Dineutus truncatus*: Blackwelder 1944: 81, Dineutus (Dineutus) truncatus: Ochs 1949: 288, Dineutus (Cyclinus) truncatus: Brinck 1955: 106, *Dineutus truncatus*: Arce-Pérez and Roughley 1999: 84.

##### Type locality.

Chontales, Nicaragua, BMNH

##### Specimens examined.

34

##### Type material.

Not examined.

##### Material examined.

**COSTA RICA:** **Alajuela:** Zapote Upala, nr. Bijaqua, 20.x.1973, leg. F. Cordera EMEC 204671 (1 ex. EMEC); same as previous except: 10.ix.1973, leg. R. Ortiz, EMEC 204908 (1 ex. EMEC); **Guanacaste:** Rincon de la Vieja N.P., Quebrada Cataracta, 15.vi.2003, leg. W.D. Shepard, EMEC 204646-204667 (22 ex. EMEC); **Puntarenas:** Las Cruces Bio. Station, water tank, 1158 m, 19.vi.2003, leg. A.E.Z. Short, (2 ex. KSEM); Monte Verde, Rio Guacimal, 1420 m, 15.v.1989, leg. J. Ashe, R. Leschen, R. Brooks, Snow Entomol. Mus. Costa Rica Exped. #198 (1 ex. KSEM); 6 km S Sta. Elena, 6-7.vi.1983, leg. J.E. Wappes (1 ex. FSCA). **PANAMA:** **Ngäbe-Buglé:** nr. Soloy, 8°36.554'N, 82°07.814'W, 7.vi.2009, leg. Nearns, Lord, small stream (6 ex. MSBA).

##### Diagnosis.

Male (Fig. 47C–D): Size: 13.5–15.4 mm. Body form regularly oval; elytral apices truncate, lateral corner of truncation roundly angled, fine serration present apically, apicolateral margin without sinuation, elytral striae primarily indistinct, dense microreticulation covering entirety of elytra and pronotum, producing a polished metal feel, elytra often with violet iridescence; ulimate protarsus (Fig. 30C) ca. 2× as long as wide; protibiae club-shaped; profemora with small acute sub-apicoventral tooth; mesotarsal claws (Fig. 48C) without ventral margin expanded into weak denticle; venter darkly colored, usually black to dark reddish brown, mesothoracic and metathoracic legs usually lighter in coloration, as well as apex of abdomen; Aedeagus (Fig. 48A, B, D) median lobe in dorsal view nearly evenly attenuated basally to apically, more strongly narrowed in apical 1/3, apex very shortly rounded, not acuminate, in lateral view median lobe ventrally weakly sinuate in apical 1/3, noticeably dorsally curved, ventrally median lobe with parallel sided sperm-groove, parameres as wide as base of median lobe apically, weakly laterally expanded in apical 1/3.

Female (Fig. 47A–B): Size: 13.3–15.1 mm. Body form regularly oval; elytral apices truncate, lateral corner of truncation roundly angled, fine serration present apically, apicolateral margin without sinuation, elytral striae primarily indistinct, visible upon close examination, dense microreticulation covering entirety of elytra and pronotum, producing a polished metal feel, elytra often with violet iridescence; ulimate protarsus ca. 2× as long as wide; protibiae club-shaped; profemora without sub-apicoventral tooth; venter darkly colored, usually black to dark reddish brown, mesothoracic and metathoracic legs usually lighter in coloration, as well as apex of abdomen.

##### Differential diagnosis.

*Dineutus truncatus* is unique among all other New World *Dineutus* in having the elytral apices truncate, with fine serration present apically, males with the ultimate protarsomeres (Fig. 30C) ca. 2× as long as wide, profemora with a small sub-apicoventral tooth present, mesotarsal claws with ventral margins not expanded into a weak denticle (Fig. 48C), and in the form of the male aedeagus (Fig. 48A). The species most similar to *Dineutus truncatus* is *Dineutus mexicanus* (originally described as a subspecies of *Dineutus truncatus* by Ochs [1925a]). Both species have their elytral apices truncate, however only *Dineutus truncatus* has the elytral apices with fine serration present, those of *Dineutus mexicanus* have the serration much more blunt and thick and/or reduced, in some specimens down to only irregularities of the elytral apices. There is a general difference between the species in the dorsoventral convexity of the body form in lateral view. In general, *Dineutus truncatus* tends to be noticeably more humped in the scutellar region with the posterior length of the elytra more dorsoventral depressed, while in *Dineutus mexicanus* the posterior length of the elytra is less dorsoventrally depressed, giving *Dineutus mexicanus* a generally less dorsoventrally convex lateral profile, without as much of a pronounced hump in the scutellar region.

Males of *Dineutus truncatus* can further be separated from males of *Dineutus mexicanus* by several characters. In *Dineutus truncatus* the elytral striae, while present, are nearly indistinct and are weakly impressed, while in *Dineutus mexicanus* the striae are evident just posterior to the middle of the elytra, disappearing laterally and before the elytral apices. The protarsi (Fig. 30) have several characters separating *Dineutus truncatus* from *Dineutus mexicanus*. The size of the ultimate protarsomere in *Dineutus truncatus* (Fig. 30C) is ca. 2× as long as wide, while in *Dineutus mexicanus* (Fig. 30A) the ulimate protarsomere is less than 2× as long as wide. The shape of the lateral margins of the protarsal tarsomeres 2–5 also differs among the species. In *Dineutus truncatus*, in anterior view, the tarsomeres are laterally flatly rounded (Fig. 30C), this differs strongly from a specimen of uncertain species assignment from Oaxaca (see specimens of uncertain placement below) in which the lateral margins of the protarsi in anterior view are roundly angled (Fig. 30B), but are similar to those of *Dineutus mexicanus* (Fig. 30A). The mesotarsal claws can also assist in separating the males of *Dineutus truncatus* (Fig. 48C) from *Dineutus mexicanus* (Fig. 29C), in that the ventral-margins lack an expansion forming a weak denticle. By far the most reliable way to separate the males of these species is by the form of the aedeagus. In *Dineutus truncatus* (Fig. 48A) the median lobe of the aedeagus is nearly evenly tapered basally to apically, with the apex narrowly rounded and appearing highly pointed, as opposed to the being acuminate as in *Dineutus mexicanus* (Fig. 29A).

Females of the species are primarily separated by the general differences listed between the species for elytral differences and body form differences. The size of the ultimate protarsomere in females shows similar ratios to the males being ca. 2× as long as wide in *Dineutus truncatus*, while in *Dineutus mexicanus* the ulimate protarsomere is less than 2× as long as wide.

##### Distribution

**(Fig. 54D).** Known only from Central America from Nicaragua to western Panama (Ochs 1949; Sharp 1882).

##### Habitat.

Lotic and lentic (Ochs 1949). The authors have collected this species from a medium sized stream with clear water, running through a small agrarian valley in Chiriquí, Panama. In the stream *Dineutus truncatus* was found in areas outside of the rapids where the water slowed and pooled, such as the stream margins or behind large rocks in the stream.

##### Discussion.

This species has the most southern range of any New World *Dineutus* being found only in Central America, as far south as Panama. It is unclear however, the true extent of the range of *Dineutus truncatus*. In Panama, all specimens observed in this study were from the western half of the country near Soloy and Chiriquí, with most references in the literature for the distribution of *Dineutus truncatus* in Panama only mentioning the latter locality (Sharp 1882; Ochs 1949). The north and western limits of this species’ range is even less clear. The type locality is Chontales, Nicaragua, and the species is readily found in Costa Rica, but literature references also suggest the limits may be as far northwest as Guatemala and Mexico (Sharp 1873, 1882; Ochs 1949). It is likely as previously suggest by Ochs (1949) that these data refer to *Dineutus mexicanus*. *Dineutus mexicanus* is superficially very similar to *Dineutus truncatus* in habitus and specimens determined to be *Dineutus mexicanus* in this study included a female specimen from Guatemala, and others from as far southeast as El Salvador. As is often becoming the case in *Dineutus*, some very similar species have overlapping ranges with one having a very limited range of endemism relative to a another more widely ranging species. *Dineutus truncatus* follows this pattern in relation to *Dineutus mexicanus*, seemingly only definitely being found in Nicaragua, Costa Rica, and western Panama. As is the case with other species with narrow ranges *Dineutus truncatus* specimens were relatively few in number, and it is not well represented in many museums.

The authors have collected this species from mountainous Chiriquí, in western Panama, the place where numerous historical references record specimens (Ochs 1949; Sharp 1873). The species is quite common through Costa Rica, which is dominated by central mountain ranges, and its type locality is Western Nicaragua, the mountainous region of the country. These records appear to suggest that *Dineutus truncatus* may be restricted to mountainous regions, and may be a mountain endemic, similar to the situation possibly seen in *Dineutus mexicanus*. It may be that the most southeastern extent of *Dineutus mexicanus*’ range is the Cordillera de Talamanca in Panama, and the northwestern most portion of its range may reflect Nicaraguan mountain ranges.

In Costa Rica where *Dineutus truncatus* is very common it has been given the common name of “mamatetas” (Ochs 1949; Hogue 1993). The literal translation from Spanish appears to mean nipple sucker or nipple suckling. Ochs (1949) translated this name to “dug-sucker” which the second meaning for dug is nipple, in agreement with the translation. The reason for this common name does not appear to be known. Ochs (1949) says that Nevermann believed it to come from superstitious ideas, but does not elaborate, nor does Ochs (1949) provide a citation for Nevermann’s interpretation, for further investigation. An interesting connection to the name mamatetas and the literal translation of nipple sucker, is the practice of some young girls in East Africa to use gyrinids to stimulate breast growth (Kutalek and Kassa 2005). Young girls allow gyrinids to bite their nipples in hopes of stimulating the breasts to grow larger, and interestingly, among the gyrinids in use are common African species of *Dineutus* (Kutalek and Kassa 2005). It is unclear, if there is any similar practice present in Costa Rica, thus resulting in the common name referring to breast suckling.

### Specimens of uncertain placement

#### Dineutus sp. near mexicanus

Taxon classificationAnimaliaColeopteraGyrinidae

##### Material examined.

“Tapanatepec 7 mi [black ink smudge]/ N.E. Oax. Mex [ handwritten in black ink] 48/ 7-9-53 1300 ft. [black handwritten in ink] S// Univ.Kans/ Mex./ Expedition” deposited at the KSEM.

##### Diagnosis.

**Figure 49.**
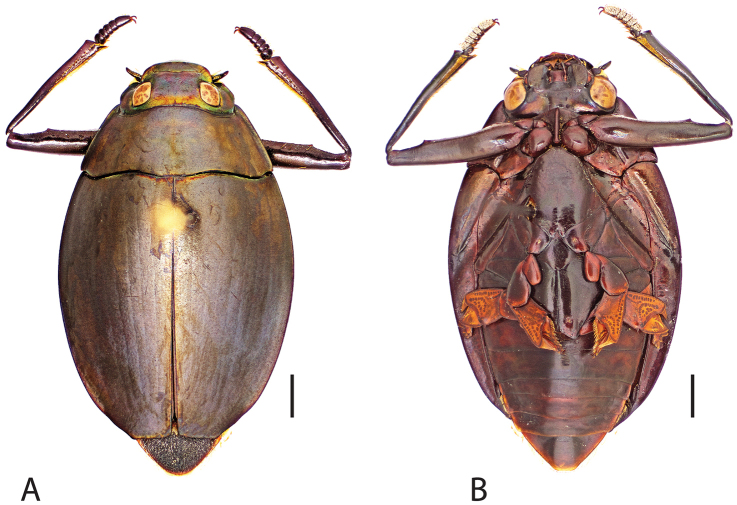
*Dineutus* sp. **A** ♂ dorsal habitus **B** ♂ ventral habitus. All scale bars ≈ 2 mm.

**Figure 50.**
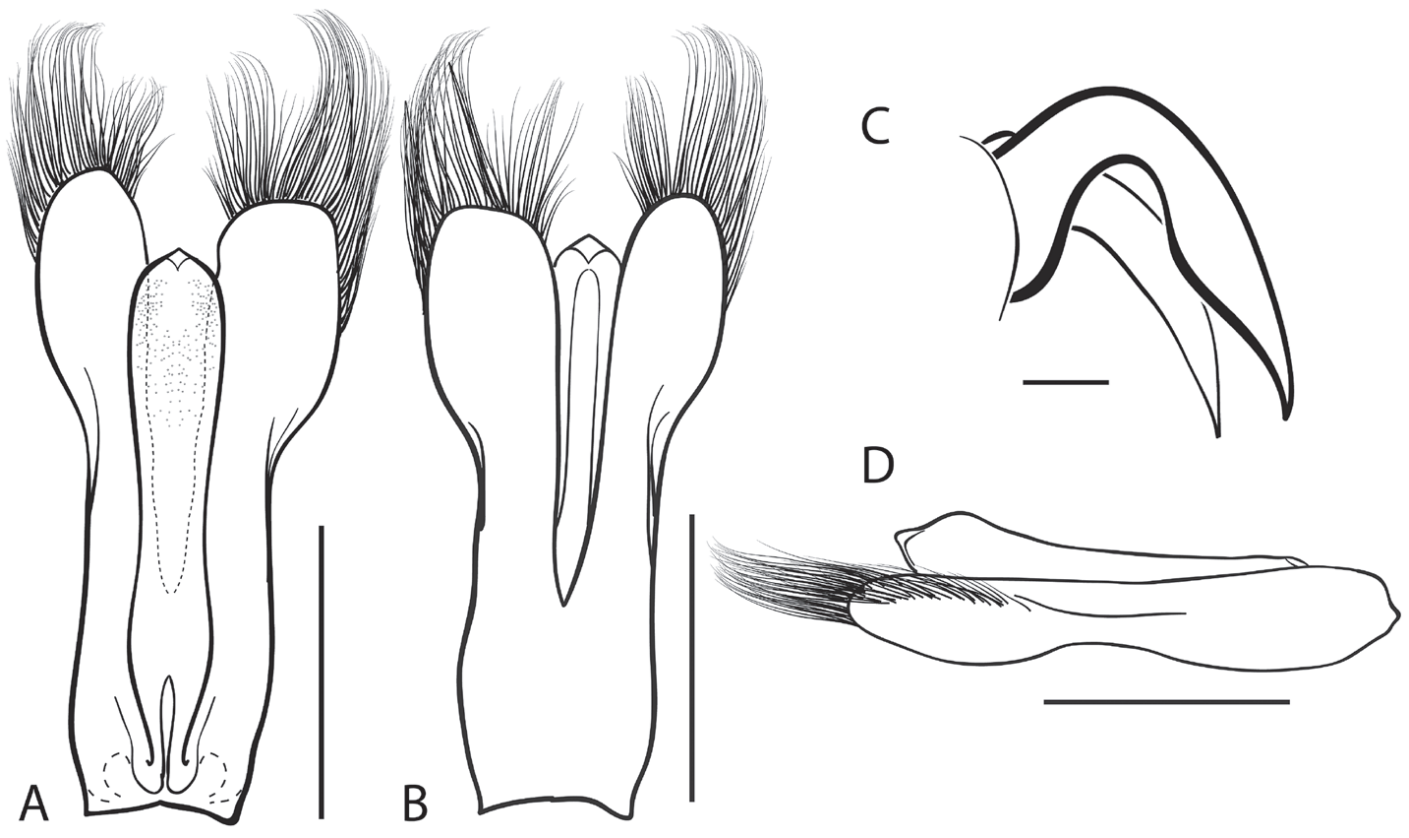
*Dineutus* sp. **A** aedeagus dorsal view **B** aedeagus ventral view **C** ♂ mesotarsal claws **D** aedeagus lateral view. Scale bar for **C** ≈ 0.10 mm all others ≈ 1 mm.

Male (Fig. 49A–B): Size: 17.45 mm. Body form regularly oval; elytral apices truncate, lateral corner of truncation roundly angled, blunt irregularities present apically, apicolateral margin weakly sinuate, elytral striae faint, elytra microreticulation present, apicolaterally with slightly bronzy appearance; ulimate protarsus (Fig. 30B) less than ca. 2× as long as wide, with angled lateral margin after basal 1/3, remaining protarsi with rounded lateral margins; protibiae club-shaped; profemora with small acute sub-apicoventral tooth; anterior mesotarsal claw (Fig. 50C) with ventral margin expanded into weak denticle; venter darkly colored, reddish brown, mesothoracic and metathoracic legs usually lighter in coloration, as well as apex of abdomen; Aedeagus (Figs 50A, B, D) with median lobe in dorsal view parallel sided, weakly laterally expanded in apical 1/4, apex shortly acuminate, ventrally with parallel sided sperm-groove, parameres noticeably laterally expanded in apical 1/3, asymmetrical, in lateral view parameres constricted near mid-length.

##### Differential diagnosis.

This specimen is different from all other New World *Dineutus* species examined in having truncate elytra with the lateral angle rounded, with weak sinuation apicolaterally, and blunt irregularities present, the protarsus (Fig. 30B) has the lateral margin rounded, the ultimate protarsomere angled apically after the basal 1/3, and the form of the aedeagus (Fig. 50A). The aedeagus of this specimen is unique among all known world species of *Dineutus* in having parameres that are medially strongly constricted in lateral view. Externally, however, the specimen is most similar to the North American species *Dineutus truncatus* and *Dineutus mexicanus*. It can be separated from *Dineutus truncatus* by the shape of the elytral truncature and the shape of the male protarsi (Fig. 30). In this specimen the elytral truncature has the lateral angles rounded and the apex with blunt irregularities, instead of the lateral angles of the truncature acute, with fine serration present at the elytral apices as in *Dineutus truncatus*. The length of the ultimate protarsomere is less than 2× the width, whereas in *Dineutus truncatus* the length is approximately 2× the width.

This specimen and *Dineutus mexicanus* have similar elytral truncature, and similar ultimate protarsomere size ratios, however the shape of the protarsus is very different. It (Fig. 30B) has the lateral margin of protarsomeres II–IV broadly rounded, whereas in *Dineutus mexicanus* (Fig. 30A) they are more flatly rounded to subtruncate. The ultimate protarsomere of this specimen is strongly angled apically after the basal 1/3 (Fig. 30B), whereas in *Dineutus mexicanus* it is evenly tapered basally to apically (Fig. 30A). Also, in this specimen the frontoclypeal suture is sinuate medially with a rounded posterior expansion, whereas in *Dineutus mexicanus* the suture is mostly flat and only weakly angled medially. The mesotarsal claws are also slightly different, with those of of this specimen having a slightly more developed denticle.

##### Discussion.

There are several characters that suggest this specimen represents a species distinct from *Dineutus mexicanus* (see the diagnosis and differential diagnosis section above). The aedeagus of this specimen is strange, even among the world *Dineutus* fauna, as discussed above. While the aedeagus does appear to be asymmetrical in the parameres, suggesting a malformation, the rest of the body is highly symmetrical, without any noticeable deformities. Other specimens encountered that were clearly aberrant in some way, had asymmetrical features in the body, usually the prothoracic legs. Given the distinct characters discussed we have included the specimen here without placing it among any of the known species. However, as we only have a single specimen, and given its very odd genitalia, we have decided not to formally describe this species due to lack of additional material. Other *Dineutus* specimens examined from western and north central Oaxaca (IEXA) belonged to *Dineutus mexicanus*. However, this specimen comes from a locality to the south separated by a mountain range, again suggesting it may indeed represent a species other than *Dineutus mexicanus*. Hopefully future collecting near Tapanatepec, Oaxaca, Mexico will allow placement of this enigmatic specimen.

### Checklist of the New World species of *Dineutus*

*Dineutus amazonicus* Hatch, 1930 Arkansas, USA

*Dineutus americanus* (Linnaeus, 1767) Bahamas, Carribean

*Dineutus angustus* LeConte, 1878 SE USA

*Dineutus assimilis* (Kirby, 1837) SE Canada; most of USA

*Dineutus carolinus* LeConte, 1868 E USA, Bahamas

*Dineutus ciliatus* (Forsberg, 1821) E USA

*Dineutus discolor* Aubé, 1838 SE Canada, E USA

*Dineutus emarginatus* (Say, 1825) E USA

*Dineutus hornii* Roberts, 1895 SE Canada, E USA

*Dineutus longimanus* (Olivier, 1791) Carribean

*Dineutus longimanus cubensis* Ochs, 1927 Cuba

*Dineutus longimanus jamaicensis* Ochs, 1938 Jamaica

*Dineutus longimanus longimanus* (Olivier, 1791) Hispaniola

*Dineutus longimanus portoricensis* Ochs, 1924 Puerto Rico

*Dineutus mexicanus* Ochs, 1925 S Mexico to El Salvador

*Dineutus nigrior* Roberts, 1895 SE Canada, E USA

*Dineutus productus* Roberts, 1895 Texas, Kansas, USA and NE Mexico

*Dineutus robertsi* Leng, 1911 SE Appalachian mountains, USA

*Dineutus serrulatus* LeConte, 1868 E USA

*Dineutus serrulatus analis* Régimbart, 1882 E USA

*Dineutus serrulatus serrulatus* LeConte, 1868 SE USA

*Dineutus solitarius* Aubé, 1838 SW USA to W Costa Rica

*Dineutus sublineatus* (Chevrolat, 1834) SW USA to Nicaragua

*Dineutus truncatus* Sharp, 1873 Nicaragua to W Panama

**Figure 51.**
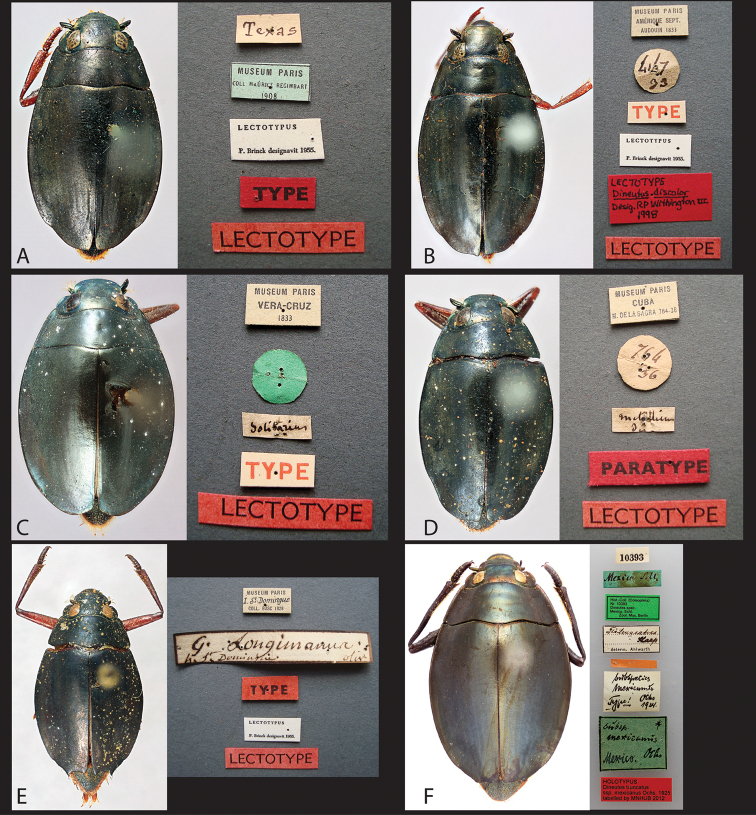
Type specimens **A** *Dineutus analis* Régimbart, 1882 lectotype and labels **B** *Dineutus discolor* Aubé, 1838 lectotype and labels **C** *Dineutus solitarius* Aubé, 1838 lectotype and labels **D** *Dineutus metallicus* Aubé, 1838 lectotype and labels **E** *Gyrinus longimanus* Olivier, 1795 lectotype and labels **F** *Dineutus truncatus mexicanus* Ochs, 1925 holotype and labels.

**Figure 52.**
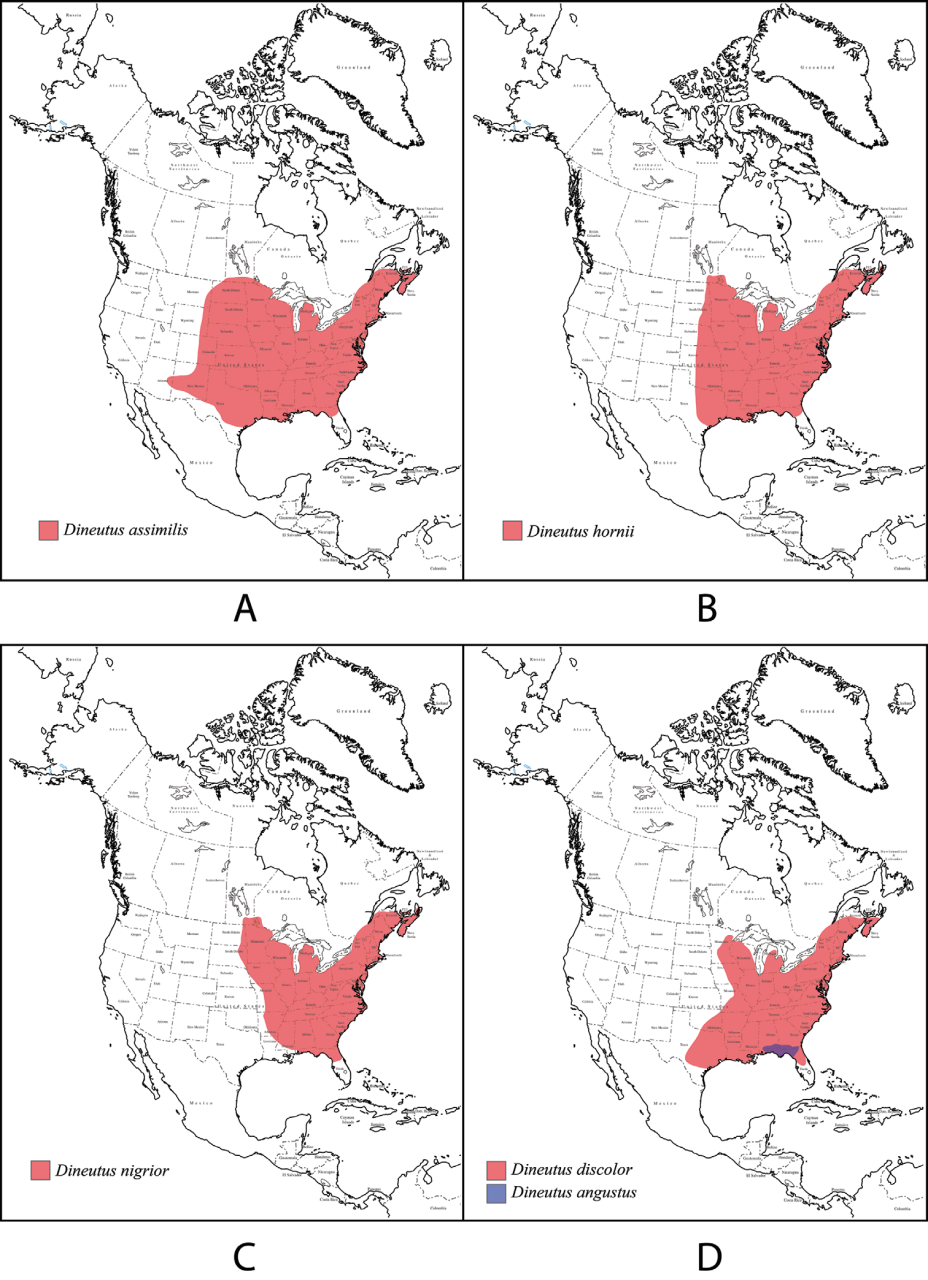
General distribution map. **A** *Dineutus assimilis* **B** *Dineutus hornii* **C** *Dineutus nigrior* **D** *Dineutus discolor* and *Dineutus angustus*.

**Figure 53.**
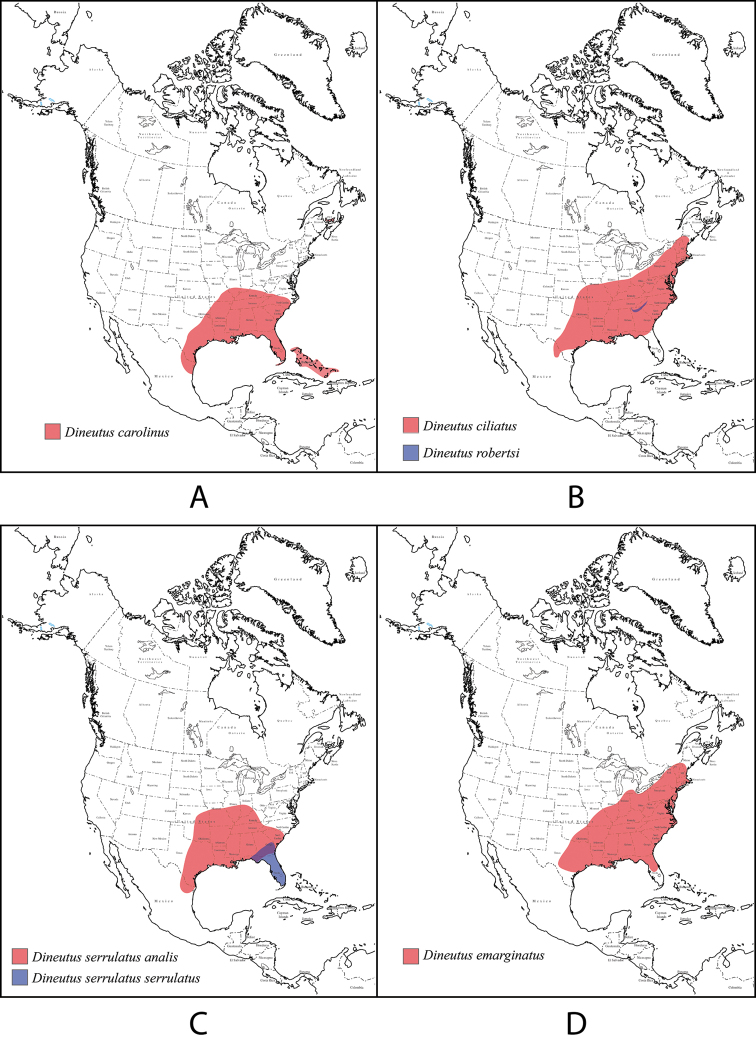
General distribution map. **A** *Dineutus carolinus* **B** *Dineutus ciliatus* and *Dineutus robertsi* **C** *Dineutus serrulatus analis* and *Dineutus serrulatus serrulatus* **D** *Dineutus emarginatus*.

**Figure 54.**
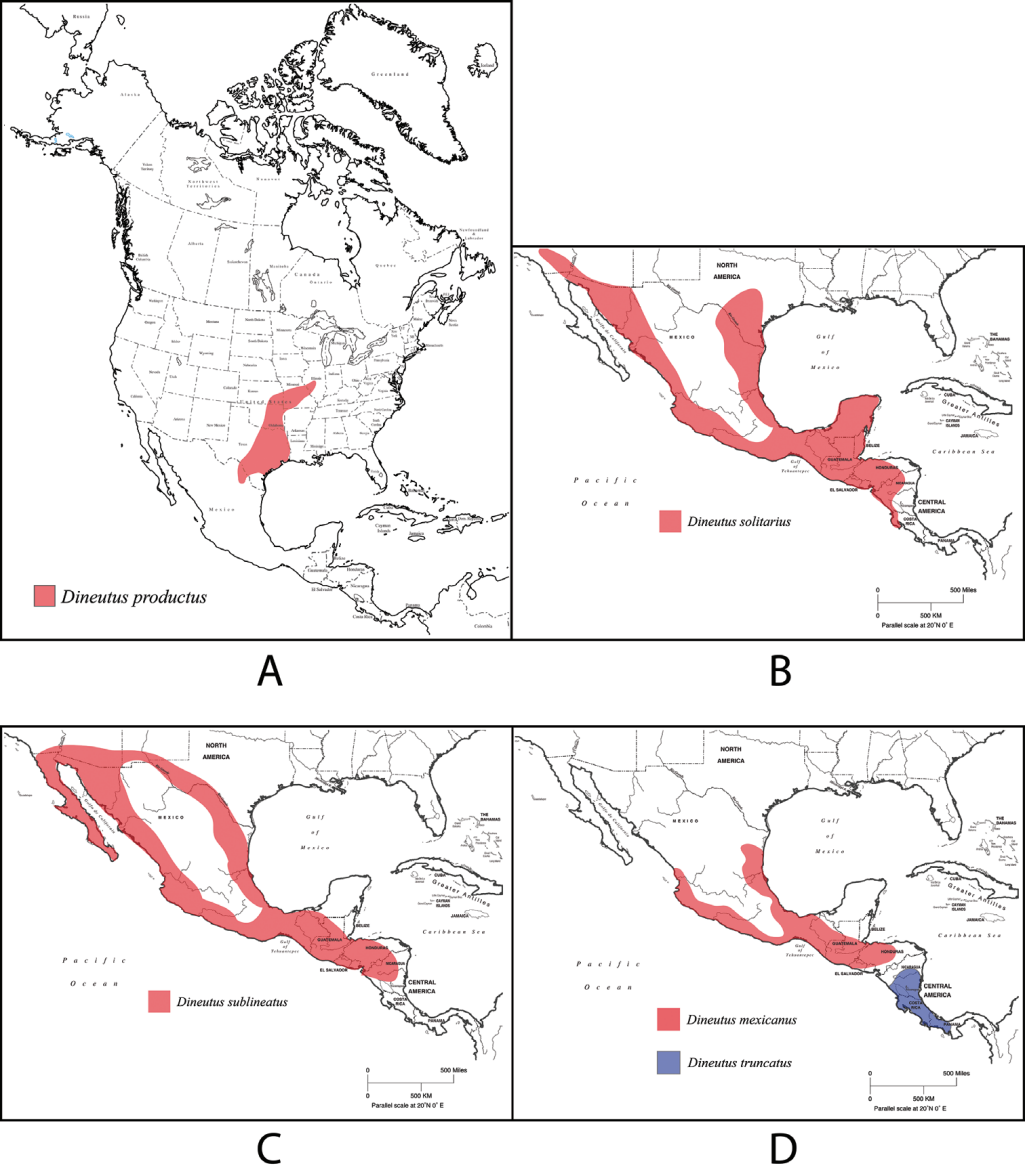
General distribution map. **A** *Dineutus productus* **B** *Dineutus solitarius* **C** *Dineutus sublineatus* **D** *Dineutus mexicanus*, *Dineutus truncatus*.

**Figure 55.**
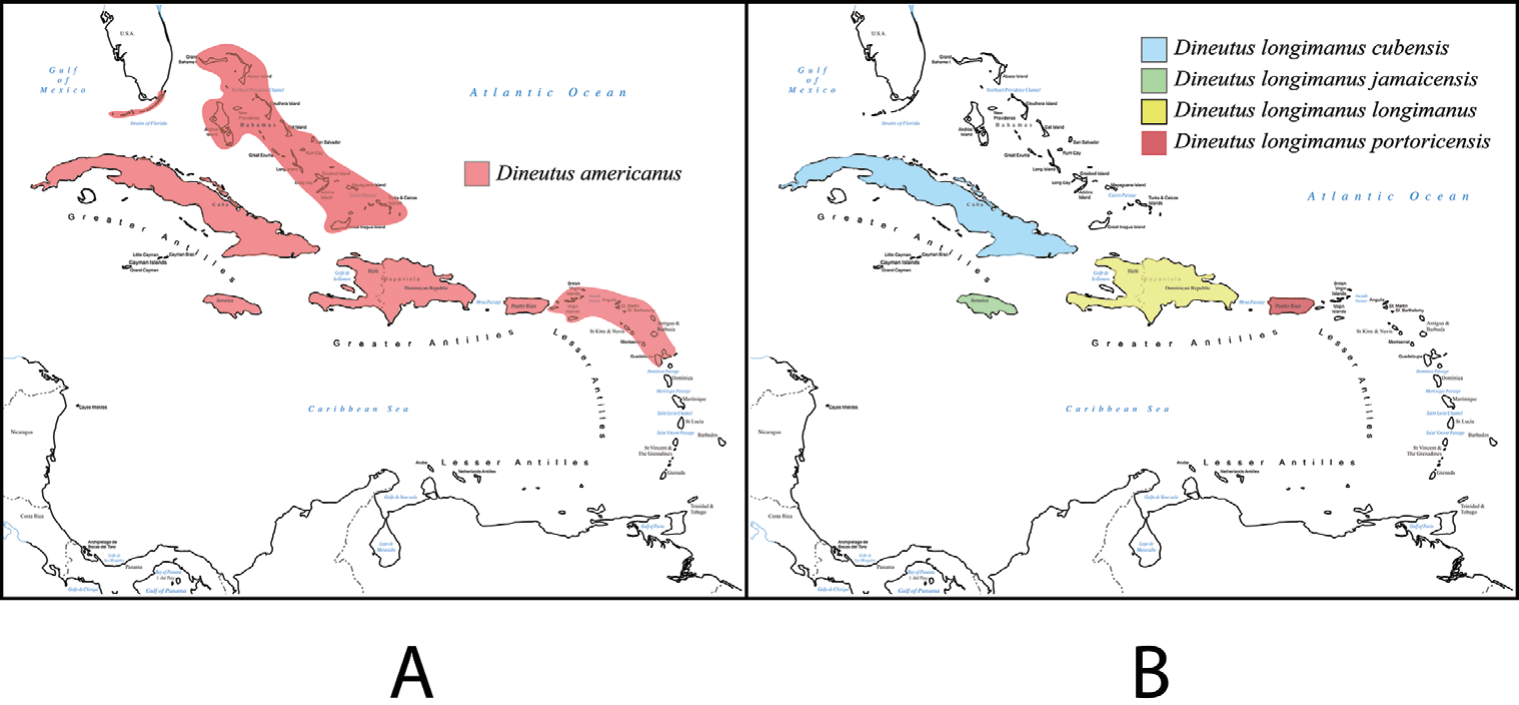
General distribution map. **A** *Dineutus americanus* **B** *Dineutus longimanus cubensis*, *Dineutus longimanus jamaicensis*, *Dineutus longimanus longimanus*, *Dineutus longimanus portoricensis*.

## Supplementary Material

XML Treatment for Dineutus amazonicus

XML Treatment for Dineutus americanus

XML Treatment for Dineutus angustus

XML Treatment for Dineutus assimilis

XML Treatment for Dineutus carolinus

XML Treatment for Dineutus ciliatus

XML Treatment for Dineutus discolor

XML Treatment for Dineutus emarginatus

XML Treatment for Dineutus hornii

XML Treatment for Dineutus longimanus

XML Treatment for Dineutus longimanus cubensis

XML Treatment for Dineutus longimanus jamaicensis

XML Treatment for Dineutus longimanus longimanus

XML Treatment for Dineutus longimanus portoricensis

XML Treatment for Dineutus mexicanus

XML Treatment for Dineutus nigrior

XML Treatment for Dineutus productus

XML Treatment for Dineutus robertsi

XML Treatment for Dineutus serrulatus

XML Treatment for Dineutus serrulatus analis

XML Treatment for Dineutus serrulatus serrulatus

XML Treatment for Dineutus solitarius

XML Treatment for Dineutus sublineatus

XML Treatment for Dineutus truncatus

XML Treatment for Dineutus sp. near mexicanus
